# Sequencing palladium-catalyzed cycloisomerization cascades in a synthesis of the gelsemine core†

**DOI:** 10.1039/d3sc01353g

**Published:** 2023-06-05

**Authors:** Guoduan Liang, Edward A. Anderson

**Affiliations:** a Chemistry Research Laboratory, Department of Chemistry, University of Oxford 12 Mansfield Road Oxford OX1 3TA UK edward.anderson@chem.ox.ac.uk

## Abstract

Transition metal-catalyzed cycloisomerization is a powerful strategy for the construction of cyclic organic molecules, and the use of palladium catalysts can deliver a wide range of monocyclic and bicyclic products. However, applications of cycloisomerizations in complex target synthesis in which more than one cycloisomerization process is deployed in a cascade context are rare. Here we report investigations of the relative rates of two different types of ene-ynamide cycloisomerization that form fused and spirocyclic rings, and use of these results to design a sequence-controlled cascade cycloisomerization that prepares the tetracyclic core of gelsemine in a single step. Crucial to this work was an evaluation of the kinetics of each cycloisomerization in competition experiments, which revealed a key influence of the ynamide electron-withdrawing group on the cycloisomerization reaction.

## Abstract 2

By investigation of the relative rates of Pd-catalyzed ene-ynamide cycloisomerization to form fused and spirocyclic rings, a sequence-controlled cascade has been developed to prepare the tetracyclic core of gelsemine in a single step.

## Graphical abstract

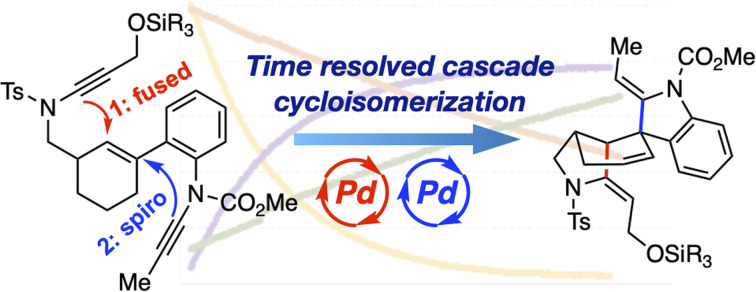

## Introduction

**Scheme 1:**
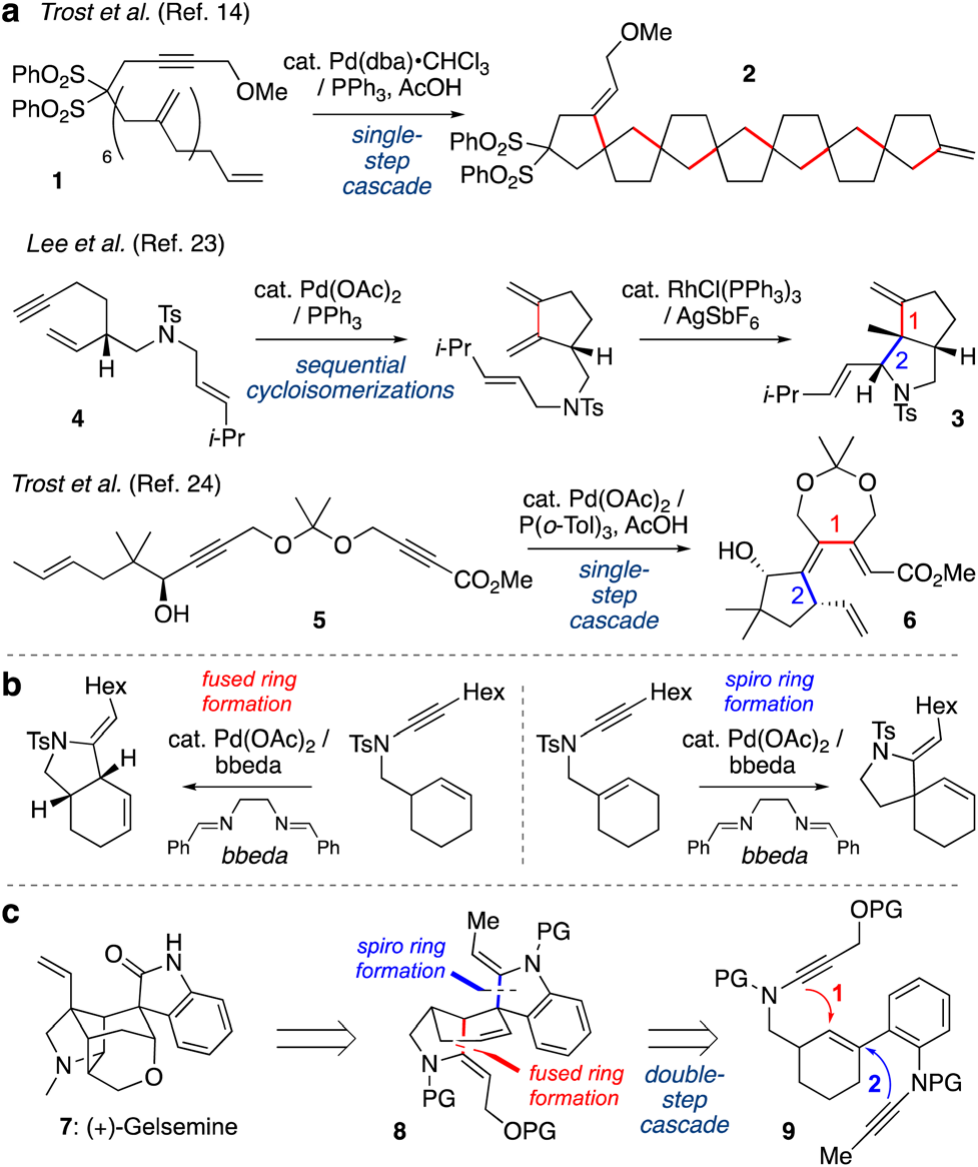
(a) Cascade or sequenced cycloisomerizations. (b) Previous work from our group on palladium-catalyzed ynamide cycloisomerization. (c) This work: time-resolved palladium-catalyzed cascade cycloisomerization towards the synthesis of the gelsemine core.

Transition metal-catalyzed cycloisomerizations have been widely explored as atom-economic processes to form cyclic organic molecules from acyclic polyunsaturated starting materials.^1–4^ Among many metals, palladium catalysts are well-established as versatile promoters of such transformations.^5–12^ When substrates feature multiple unsaturated bonds, elegant cascade processes have been developed whereby intermediate alkyl- or alkenylpalladium species undergo multiple C–C bond forming events in a single step;^13^ one pioneering example from the Trost group is shown in Scheme 1a (1 → 2).^14^ Pd-catalyzed cycloisomerizations have also been widely used in total synthesis,^1,15–22^ however applications of cascade cycloisomerizations are rare in this context. Sequential cycloisomerizations have been reported, such as an elegant approach to dendrobine described by the Chen group in which palladium- and rhodium-catalyzed cycloisomerizations were implemented in consecutive steps to construct the bicyclic pyrrolidine 3 from dienyne 4.^23^ A palladium-catalyzed cycloisomerization cascade was described by Trost *et al.* in which dienyne 5 underwent cyclization to triene 6 in the synthesis of various terpenoid natural products.^24^ However, to our knowledge, no examples of cascade cycloisomerizations have been reported in which a transition metal catalyst operates twice on a reaction substrate, where the order of events is critical for reaction success.

Our group has developed a variety of metal-catalyzed cycloisomerizations of alkenyl ynamides (*e.g.*, Scheme 1b).^25–27^ These compounds are readily accessed by alkynylation of appropriate carbamate and sulfonamide precursors,^28–36^ and undergo Pd-catalyzed cyclizations to azacycles under mild reaction conditions. We further studied the mechanism of these cycloisomerizations, where a series of deuterium-labelling studies demonstrated that the so-called ‘ligand’ bis(benzylidene)ethylenediamine (bbeda) in fact also serves a source of a palladium(ii) hydride species that initiates the catalysis.^37^ Building from this work, we questioned whether we could develop a cycloisomerization cascade in which two discrete cycloisomerizations would be sequenced in a time-resolved manner, thus generating products of greater complexity with higher efficiency.

We were particularly attracted to the challenge of differentiating between fused-ring and spiro-ring formation (as shown in Scheme 1b), as we recognized that a sequence of these reactions could generate the core scaffold of the indole alkaloid gelsemine (7, Scheme 1c). This natural product features a hexacyclic core with seven contiguous stereocenters, including two quaternary carbons, whose intriguing structure has attracted the attention of many chemists.^38–41^ We questioned whether the challenging tetracyclic spirooxindole core of gelsemine could be obtained using a one-pot cascade polycyclization sequence. Specifically, tetracycle 8 might derive from cyclization of bis-ynamide 9 by initial fused-ring formation (Step 1), and then spiro-ring formation (Step 2), ‘walking’ the double bond of the linking cyclohexene around the six-membered ring. To achieve this time-resolved process, an understanding of the relative rates of each process would be critical, as if Step 2 occurs before Step 1 then a totally different skeleton would be formed. We were aware that the relative rates of these processes would likely depend not only on the conformational demands of the cyclizations, and the relative rates of the elementary steps of the cycloisomerization, but also on the nature of the ynamide electron-withdrawing groups, which had not previously been studied. Here we describe the exploration of these factors, and the successful execution of this cascade cycloisomerization, which to our knowledge represents the first example of the sequencing of two independent ring-forming events in palladium-catalyzed cycloisomerization chemistry.

## Results and discussion

**Scheme 2:**
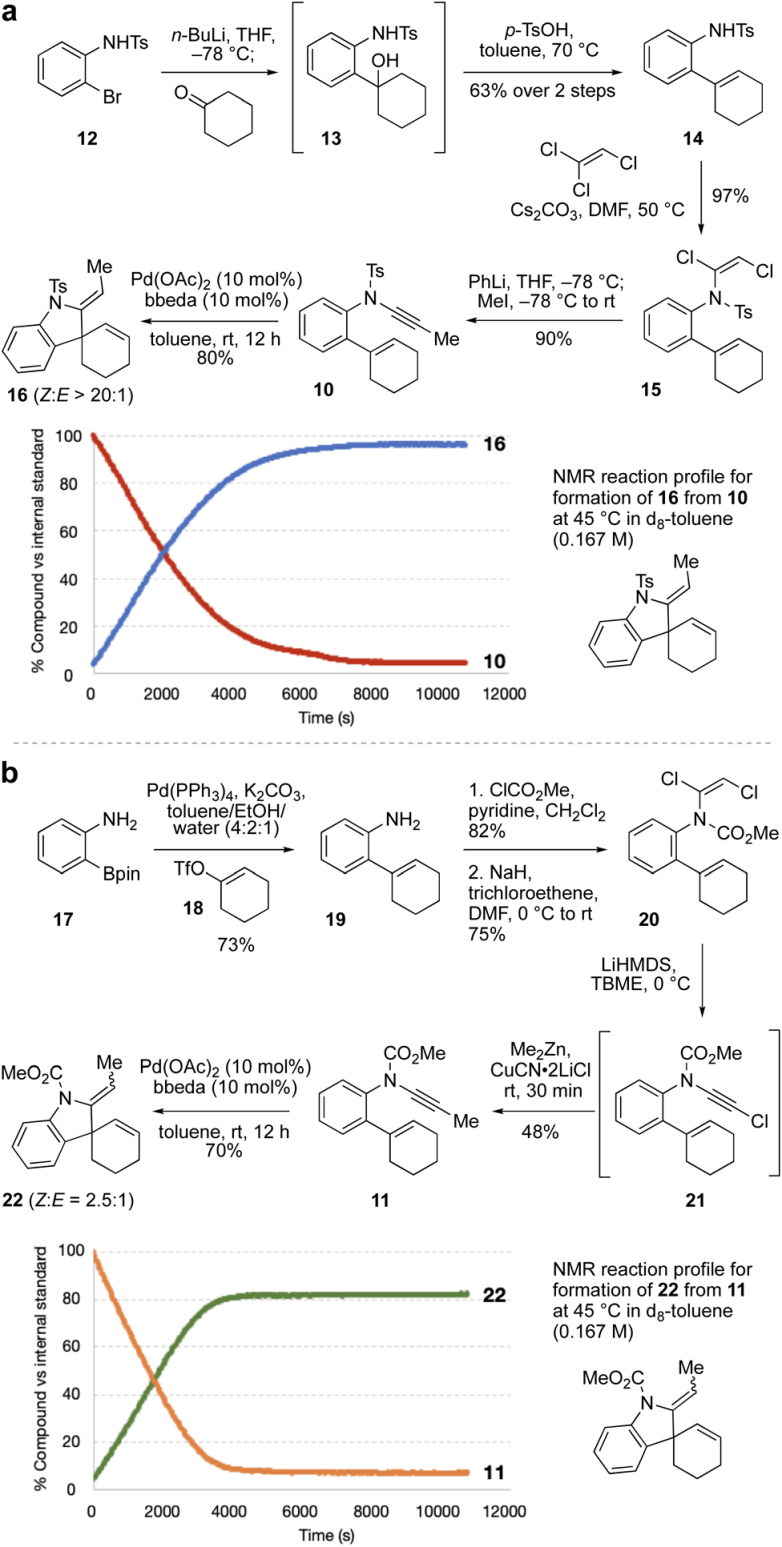
Model spiro-ring synthesis (a), model fused-ring synthesis (b), and reaction kinetics.

As the relative rate of spiro ring formation (Step 2 in Scheme 1c) compared to fused ring formation was expected to be crucial for the success of the cascade, we first synthesized two model ‘spiro’ enynamide substrates 10 and 11 (Scheme 2a and b respectively), differing in the nature of the ynamide electron-withdrawing group, which as noted above could make a significant difference to the rate of cyclization. The synthesis of *N*-aryl ynamides can be challenging, and we selected an approach based on dichloroenamide precursors developed previously by our group.^42,43^ Towards sulfonamide ynamide 10, lithiation of bromide 12 ^44^ followed by addition to cyclohexanone gave tertiary alcohol 13 which, without purification, underwent dehydration to afford alkene 14 (63% over two steps). Treatment of 14 with trichloroethene and Cs_2_CO_3_ gave dichloroenamide 15 (97%), which was converted to *N*-tosyl ynamide 10 on treatment with PhLi, then iodomethane (90%). Subjection of 10 to Pd(OAc)_2_/bbeda (10 mol%) at room temperature for 12 h gave the spiroindoline 16 in 80% yield as a single alkene diastereomer. Other catalyst systems tested (*e.g.* Pd(OAc)_2_/2PPh_3_, or Pd(OAc)_2_ alone) also proceeded efficiently.^45^ An NMR reaction profile obtained at 45 °C revealed that the reaction reached completion after around 2 hours at that temperature.

Synthesis of the equivalent *N*-carbamate ynamide 11 was attempted using a similar route, but proved unsuccessful due to formation of a cyclic carbamate in the first step (by cyclization of the tertiary alcohol onto the *N*-carbamate in the equivalent of intermediate 13).^45^ Instead, Suzuki coupling of boronic ester 17 ^46^ and enol triflate 18 afforded aniline 19 in 73% yield, which was converted to dichloroenamide 20 in two steps (62%). Treatment of 20 with LiHMDS generated an intermediate chloroynamide 21, which underwent *in situ* copper-catalyzed cross-coupling with Me_2_Zn to give ynamide 11 in 48% yield. Pd(OAc)_2_/bbeda-catalyzed cyclization of 11 at room temperature afforded the spirocyclic product 22 in 70% yield, as a 2.5 : 1 (*Z* : *E*) mixture of diastereomers. Interestingly, the two cycloisomerizations (of 10 and 11) appeared to proceed at quite similar reaction rates; an NMR reaction profile obtained at 45 °C revealed the reaction of 11 reached completion after around 1 hour.

**Scheme 3:**
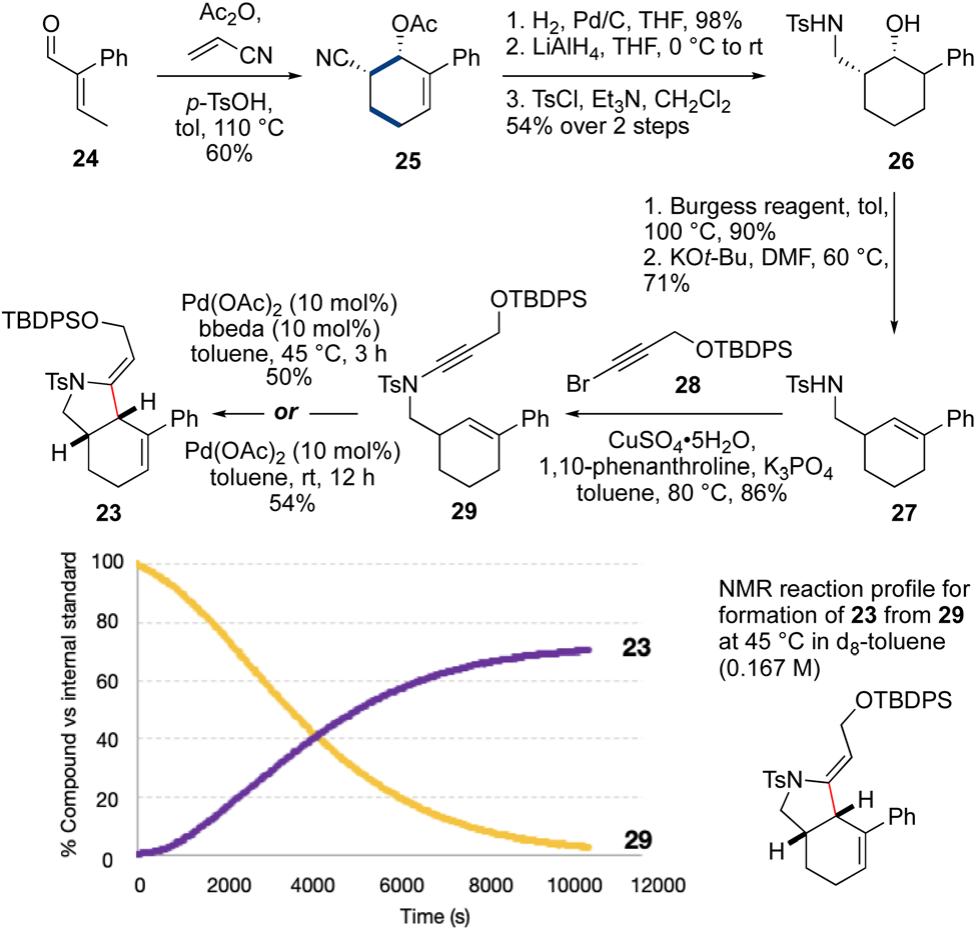
Pd-catalyzed cycloisomerization to fused-ring model 23.

We next targeted a model fused-ring system (23, Scheme 3). An *N*-tosyl electron-withdrawing group was selected due to the efficiency of ynamide formation for aliphatic amides with this group, compared to carbamates. This synthesis commenced with an Ac_2_O-promoted Diels–Alder reaction^47^ between commercially-available aldehyde 24 and acrylonitrile (60%). Reduction of the cyclohexene (H_2_, Pd/C) and the nitrile (LiAlH_4_) followed by tosylation of the resulting amine afforded 26 (53% over three steps). Elimination of the alcohol using the Burgess reagent, and base-promoted isomerization of the alkene^48^ gave 27 exclusively, with the alkene in conjugation with the aromatic ring. Sulfonamide 27 was then coupled^33^ with bromoalkyne 28 to afford ynamide 29 (86%). Cycloisomerization of 29 using Pd(OAc)_2_/bbeda did not proceed at room temperature, but on heating to 45 °C for 3 h gave the desired fused-ring bicyclic product 23 in 50% yield. Surprisingly, use of Pd(OAc)_2_ alone^6^ proved similarly effective, generating 23 in 54% yield. An NMR reaction profile obtained at 45 °C revealed the reaction of 29 reached completion at around 3 hours.

**Fig. 1:**
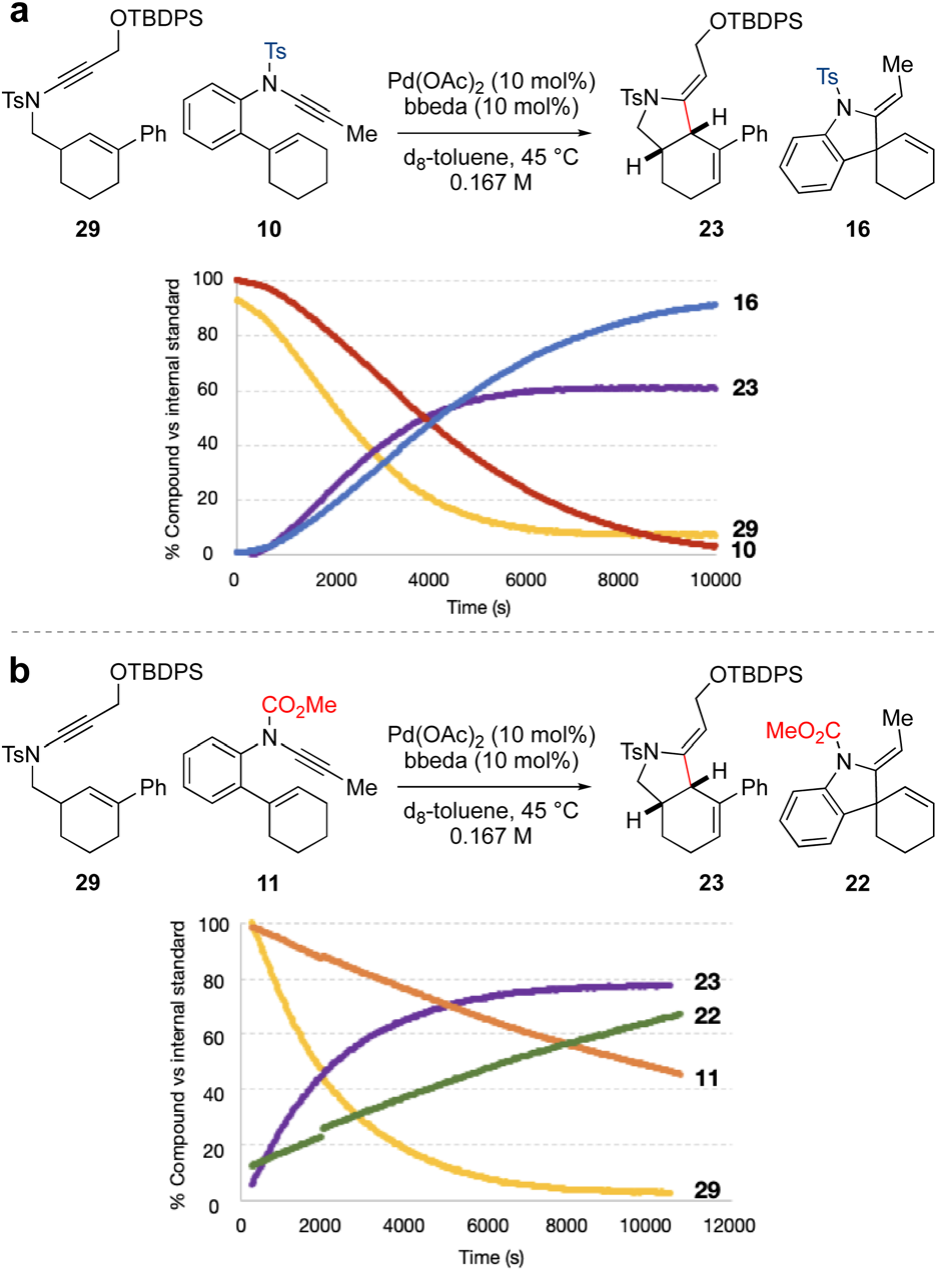
Competition experiments for Pd-catalyzed cycloisomerization of 10 and 11*vs.*29.

The individual NMR timecourse experiments (at 45 °C) suggested that spiro-ring synthesis should be favoured over fused-ring synthesis, which is contrary to our synthetic design. To further explore this, competition experiments were carried out between ynamides 10 and 29, and ynamides 11 and 29, each in a 1 : 1 ratio (Fig. 1). We first compared the reactivity of the two sulfonamide-substituted ynamides 10 and 29 (Fig. 1a). This revealed somewhat similar rates of ynamide consumption and product formation for the two substrates. However, to our surprise, the equivalent competition between *N*-carbamate ynamide 11 and *N*-tosyl ynamide 29 (Fig. 1b) resulted in quite distinct reaction profiles, in which sulfonamide-substituted fused-ring formation outcompeted carbamate-substituted spiro-ring formation, in spite of the significantly higher rate of reaction of the latter when conducted in isolation (compare Schemes 2b and 3). In a competition setting, this appears to suggest preferential complexation of the sulfonamide-substituted ynamide to the palladium(ii) catalyst over the carbamate-substituted ynamide. Cycloisomerization of the former may proceed *via* a low-energy intermediate (*i.e.* a catalyst resting state) that retards the overall observed rate of reaction for both compounds.

**Scheme 4:**
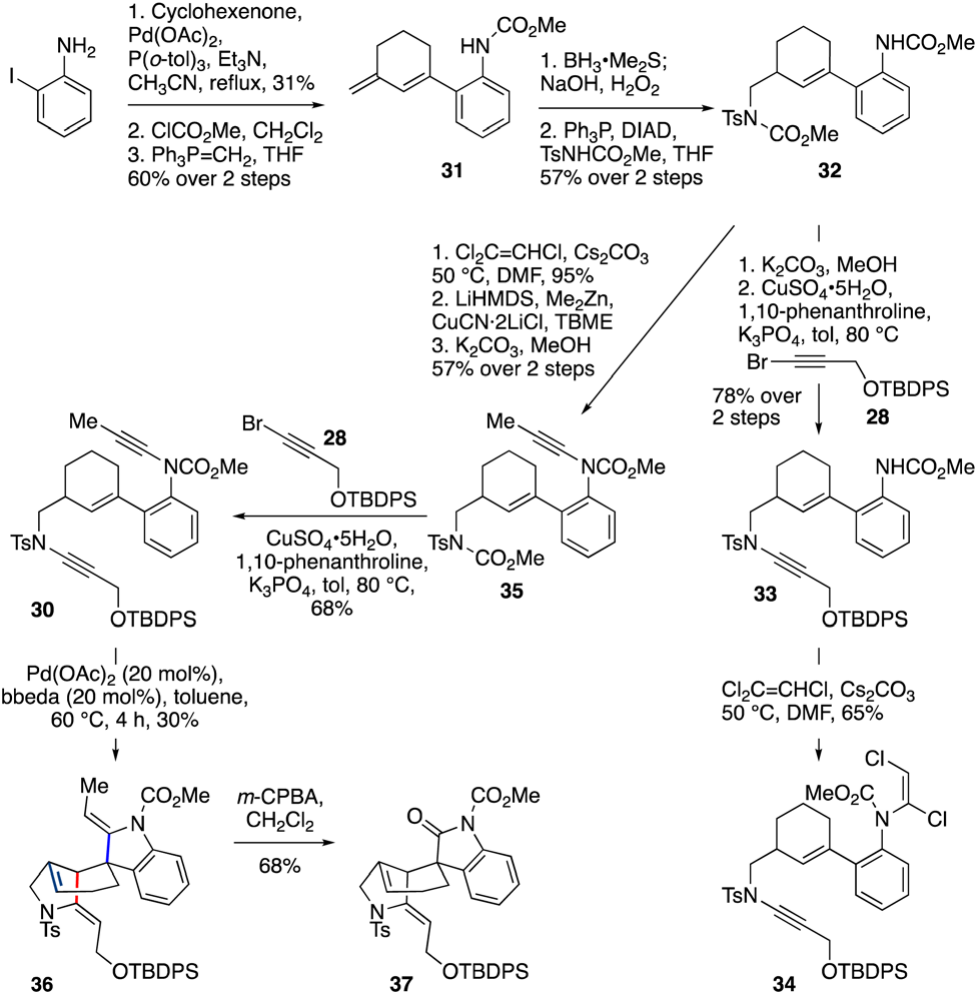
Pd-catalyzed cascade cycloisomerization to the tetracyclic core of gelsemine, 36.

This fortunate finding set the stage for execution of the double cycloisomerization cascade, in which the spiro-ring ynamide would feature a carbamate, and the fused-ring ynamide a sulfonamide (*i.e.*, bis-ynamide 30, Scheme 4). The synthesis of 30 began with a Heck reaction^49^ of 2-iodoaniline with cyclohexenone, which after carbamoylation of the aniline and Wittig olefination of the ketone gave diene 31 (19% yield over three steps). Regioselective hydroboration/oxidation of 31 and subsequent Mitsunobu reaction gave 32 (57% yield over two steps). After selective carbamate deprotection of the sulfonamide-bearing nitrogen, the resulting sulfonamide was coupled^33^ with bromoalkyne 28 to obtain 33 (78% yield over two steps). In preparation for installation of the second ynamide, dichloroenamide 34 was first formed;^42,43^ however, treatment of 34 under ynamide-forming conditions (using LiHMDS) led only to unexpected cleavage of the sulfonamide ynamide,^45^ with no formation of the desired bis-ynamide 30 being observed.

Pleasingly, this obstacle could be overcome by switching the order of ynamide formation. Thus, dichloroenamide synthesis from 32 (95%) was followed by conversion to the methyl ynamide 35*via* elimination (with LiHMDS), copper-catalyzed cross-coupling of the intermediate chloroalkyne with dimethylzinc, and cleavage of the ‘sulfonamide’ carbamate. Coupling of 35 with bromoalkyne 28 successfully afforded bis-ynamide 30. To our delight, subjection of 30 to Pd(OAc)_2_/bbeda-catalyzed cyclization conditions gave tetracyclic compound 36 – the gelsemine core – in 30% yield. Surprisingly, this cascade was accompanied by migration of the double bond of the six membered ring to the (more-substituted) ring junction, presumably by chain-walking of the palladium(ii) hydride species.^50–52^ This yield is comparable with that of the two individual cyclizations (50% and 70% for 29 and 11 respectively); based on observations with the model system 29 we believe the low yield is mainly impacted by substrate degradation during the first fused-ring cycloisomerization. Finally, we demonstrated that differentiation of the two enamides in this product could be achieved on reaction with *m*-CPBA,^53^ which led to selective oxidative cleavage of the indoline enamide double bond, furnishing the spirooxindole 37 in 68% yield, as required in the gelsemine framework.

## Conclusions

In summary, we have demonstrated a time-resolved palladium-catalyzed cycloisomerization cascade, in which sequential fused- and then spiro-ring formation accessed the core of the natural product gelsemine. Critical to this chemistry was an understanding of the relative reactivity of the different ynamides involved, where kinetics studies revealed a preferential (but slower) reaction of a sulfonamide-substituted ynamide over a carbamate-substituted ynamide, perhaps suggesting this reaction may proceed *via* an intermediate resting state that acts as a catalyst reservoir. Application of these findings enabled a 10 step synthesis of the tetracyclic gelsemine core, but perhaps more significantly opens up opportunities for further invention and exploitation of cycloisomerization cascades in synthesis.

## Data availability

All data for experimental procedures and compound characterization are available in the paper and its ESI files.†

## Author contributions

G. L. and E. A. A. conceived the work. G. L. performed the experiments and analysed the results. E. A. A. directed the project. The manuscript was written through contributions of all authors. All authors have given approval to the final version of the manuscript.

## Conflicts of interest

There are no conflicts to declare.

## Supplementary Material

SC-014-D3SC01353G-s001

## References

[cit1] Hu Y., Bai M., Yang Y., Zhou Q. (2017). Org. Chem. Front..

[cit2] Yamamoto Y. (2012). Chem. Rev..

[cit3] Michelet V., Toullec P. Y., Genet J. P. (2008). Angew. Chem., Int. Ed..

[cit4] Aubert C., Buisine O., Malacria M. (2002). Chem. Rev..

[cit5] Trost B. M., Tanoury G. J., Lautens M., Chan C., Macpherson D. T. (1994). J. Am. Chem. Soc..

[cit6] Trost B. M., Romero D. L., Rise F. (1994). J. Am. Chem. Soc..

[cit7] Nugent J., Matoušová E., Banwell M. G., Willis A. C. (2017). J. Org. Chem..

[cit8] Trost B. M. (1990). Acc. Chem. Res..

[cit9] Yadav S., Ramasastry S. S. V. (2020). Chem.–Asian J..

[cit10] Mondal S., Ballav T., Biswas K., Ghosh S., Ganesh V. (2021). Eur. J. Org. Chem..

[cit11] Lanzi M., Cañeque T., Marchiò L., Maggi R., Bigi F., Malacria M., Maestri G. (2018). ACS Catal..

[cit12] Biemolt J., Ruijter E. (2018). Adv. Synth. Catal..

[cit13] Gabriele B., Mancuso R., Veltri L., Ziccarelli I., Della Ca N. (2019). Eur. J. Org. Chem..

[cit14] Trost B. M., Shi Y. (1993). J. Am. Chem. Soc..

[cit15] Gao N., Banwell M. G., Willis A. C. (2017). Org. Lett..

[cit16] Ma X., Gao N., Banwell M. G., Carr P. D., Willis A. C. (2017). J. Org. Chem..

[cit17] Nugent J., Matoušová E., Banwell M. G. (2015). Eur. J. Org. Chem..

[cit18] Zilke L., Hall D. G. (2012). Eur. J. Org. Chem..

[cit19] Peixoto P. A., Severin R., Tseng C.-C., Chen D. Y. K. (2011). Angew. Chem., Int. Ed..

[cit20] Trost B. M., Dong L., Schroeder G. M. (2005). J. Am. Chem. Soc..

[cit21] Trost B. M., Li Y. (1996). J. Am. Chem. Soc..

[cit22] Trost B. M., Hipskind P. A., Chung J. Y. L., Chan C. (1989). Angew. Chem., Int. Ed..

[cit23] Lee Y., Rochette E. M., Kim J., Chen D. Y. K. (2017). Angew. Chem., Int. Ed..

[cit24] Trost B. M., Min C. (2020). Nat. Chem..

[cit25] Walker P. R., Campbell C. D., Suleman A., Carr G., Anderson E. A. (2013). Angew. Chem., Int. Ed..

[cit26] Straker R. N., Peng Q., Mekareeya A., Paton R. S., Anderson E. A. (2016). Nat. Commun..

[cit27] Mansfield S. J., Christensen K. E., Thompson A. L., Ma K., Jones M. W., Mekareeya A., Anderson E. A. (2017). Angew. Chem., Int. Ed..

[cit28] Zhao L., Yang H., Li R., Tao Y., Guo X.-F., Anderson E. A., Whiting A., Wu N. (2021). J. Org. Chem..

[cit29] Andna L., Miesch L. (2019). Org. Biomol. Chem..

[cit30] Zeng X., Tu Y., Zhang Z., You C., Wu J., Ye Z., Zhao J. (2019). J. Org. Chem..

[cit31] Yang Y., Zhang X., Liang Y. (2012). Tetrahedron Lett..

[cit32] Coste A., Karthikeyan G., Couty F., Evano G. (2009). Angew. Chem., Int. Ed..

[cit33] Zhang X., Zhang Y., Huang J., Hsung R. P., Kurtz K. C. M., Oppenheimer J., Petersen M. E., Sagamanova I. K., Shen L., Tracey M. R. (2006). J. Org. Chem..

[cit34] Hirano S., Tanaka R., Urabe H., Sato F. (2004). Org. Lett..

[cit35] Dunetz J. R., Danheiser R. L. (2003). Org. Lett..

[cit36] Witulski B., Stengel T. (1998). Angew. Chem., Int. Ed..

[cit37] Mekareeya A., Walker P. R., Couce-Rios A., Campbell C. D., Steven A., Paton R. S., Anderson E. A. (2017). J. Am. Chem. Soc..

[cit38] Ghosh A., Carter R. G. (2019). Angew. Chem., Int. Ed..

[cit39] Chen X., Duan S., Tao C., Zhai H., Qiu F. G. (2015). Nat. Commun..

[cit40] Zhou X., Xiao T., Iwama Y., Qin Y. (2012). Angew. Chem., Int. Ed..

[cit41] Lin H., Danishefsky S. J. (2003). Angew. Chem., Int. Ed..

[cit42] Mansfield S. J., Smith R. C., Yong J. R. J., Garry O. L., Anderson E. A. (2019). Org. Lett..

[cit43] Mansfield S. J., Campbell C. D., Jones M. W., Anderson E. A. (2015). Chem. Commun..

[cit44] Paleo M. R., Castedo L., Dominguez D. (1993). J. Org. Chem..

[cit45] See the ESI† for details

[cit46] Ortgies S., Breder A. (2015). Org. Lett..

[cit47] Strübing D., von Wangelin A. J., Neumann H., Gördes D., Hübner S., Klaus S., Spannenberg A., Beller M. (2005). Eur. J. Org. Chem..

[cit48] Hassam M., Taher A., Arnott G. E., Green I. R., van Otterlo W. A. L. (2015). Chem. Rev..

[cit49] Reisman S. E., Ready J. M., Weiss M. M., Hasuoka A., Hirata M., Tamaki K., Ovaska T. V., Smith C. J., Wood J. L. (2008). J. Am. Chem. Soc..

[cit50] Sommer H., Juliá-Hernández F., Martin R., Marek I. (2018). ACS Cent. Sci..

[cit51] Kochi T., Hamasaki T., Aoyama Y., Kawasaki J., Kakiuchi F. (2012). J. Am. Chem. Soc..

[cit52] Goj L. A., Widenhoefer R. A. (2001). J. Am. Chem. Soc..

[cit53] Wang Q., Zhang L., Yao J., Qiu G., Li X., Zhou H. (2018). J. Org. Chem..

