# Supplementary material for: Sequencing palladium-catalyzed cycloisomerization cascades in a synthesis of the gelsemine core
Source: Chem Sci. 2023 Jun 5;14(25):6970–4. doi: 10.1039/d3sc01353g (PMC10306092; doi:10.1039/d3sc01353g)
Supplement: SC-014-D3SC01353G-s001 [file SC-014-D3SC01353G-s001.pdf]

Electronic Supplementary Material (ESI) for Chemical Science.
This journal is © The Royal Society of Chemistry 2023

Supporting Information

Sequencing palladium-catalyzed cycloisomerization cascades in a synthesis of the gelsemine core

Guoduan Liang, Edward A. Anderson*

Chemistry Research Laboratory, Department of Chemistry, University of Oxford, 12 Mansfield Road, Oxford OX1 3TA, United Kingdom

Contents

1. General experimental……………………………………….……………...….2
2. Additional experimental results………………………………………………...3
   2.1 Cyclization for model spiro synthesis with different catalyst systems….……….3
   2.2 Optimization of installation of the dichloroethylene moiety…..………...…....3
   2.3 Kinetics studies………………………………………………….………....4
3. Characterization of compounds……………………………………….........…4
4. Reference……………………………………...……..….……........……32
5. NMR spectra of compounds.…………….…………...………….….........…33

1. General experimental

Regents, solvents and reaction conditions: All reactions were performed in oven-dried glassware under an N_2 atmosphere unless stated otherwise. Tetrahydrofuran, dichloromethane, dimethylformamide, dioxane, toluene and triethylamine were obtained anhydrous from solvent dispenser units having been passed through an activated alumina column under nitrogen. Anhydrous methanol and dimethylsulfoxide were obtained commercially and used without further purification. Brine refers to a saturated aqueous solution of NaCl. Heated reactions used an oil bath as the heat source.

NMR Spectra: Proton (^{1}H) and carbon (^{13}C) NMR spectra were recorded on a Bruker AVII500 (500/125 MHz), Bruker DPX400 (400/100 MHz) or Bruker AVF400 (400/100 MHz). Proton and carbon chemical shifts (δ_H, δ_C) are quoted in ppm. ^{1}H NMR spectra were recorded using an internal deuterium lock for the residual protons in $CDCl_3$ (δ 7.26) and C_6D_6 (δ 7.16). ^{13}C NMR Spectra were recorded using an internal deuterium lock using solvents $CDCl_3$ (δ 77.0) and C_6D_6 (δ 128.06). Assignments were made on the basis of chemical shifts, coupling constants, COSY, HSQC, HMBC, nOe, NOESY data and comparison with spectra of related compounds. Resonances are described using the following abbreviations: s (singlet), d (doublet), t (triplet), q (quartet), m (multiplet), br. (broad). Coupling constants (*J*) are given in Hz and are rounded to the nearest 0.1 Hz.

Mass Spectra: Low resolution mass spectra were recorded on a Micromass LCT Premier spectrometer (ESI). High resolution mass spectra were recorded by the Mass Spectrometry service of the Chemistry Research laboratory, University of Oxford, using a Bruker Daltronics microTOF spectrometer (ESI). *m/z* values are reported in Daltons with their percentage abundances and relevant fragment ions in parentheses. High resolution values are calculated to four decimal places from the molecular formula, all found values being within a tolerance of 5 ppm.

Infrared Spectra: Infrared spectra were recorded on a Bruker Tensor 27 Fourier transform spectrometer, as a thin film on NaCl plates or a diamond ATR module. Absorption maxima

Melting Points: Melting points were recorded on a Leica Galen III Compound Microscope and are uncorrected.

Chromatography: TLC was performed on Merck Keiselgel 60 F254 0.2 mm precoated plates and visualized using basic potassium permanganate dip, acidic vanillin dip or ultraviolet light. Retention factors are reported with the solvent system in parentheses. Column chromatography was performed

on Merck Keiselgel 60 SiO_2 (40-63 μm) and the solvent system used is recorded in parentheses.

2. Additional experimental results

2.1 Cyclization for model spiro synthesis with different catalyst systems

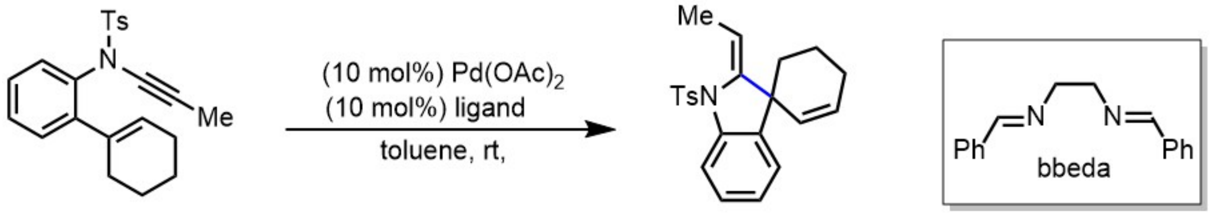

Entry	Ligand	Temperature	Time (h)	Isolated yield (%)
1	bbeda	rt.	12	80
2	Ph_3P	rt.	12	78
3	-	rt.	12	79

2.2 Optimization of synthesis of the dichloroenamide

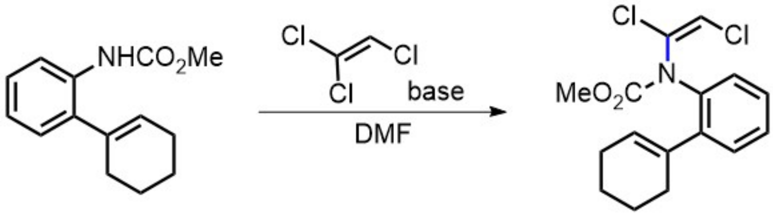

Entry	Base	Temperature	equivalents of $HClC{=}CCl_2$	Conversion (%)[c]
1	1.5 equiv. Cs_2CO_3	50 °C	1	<3
2	1.5 equiv. Cs_2CO_3	50 °C	3	6
3	1.5 equiv. Cs_2CO_3	60 °C	3	6
4[a]	1.5 equiv. NaH	0 °C to rt	1	~50
5[a]	2 equiv. NaH	0 °C to rt	1	~50
6[b]	2 equiv. NaH	0 °C to rt	1.2	100

[a] Deprotonation of carbamate for 30 min before addition of trichloroethylene. [b] Deprotonation of carbamate for 1 h before addition of trichloroethylene. [c] Conversion based on ^{1}H NMR spectrum of the crude reaction mixture.

2.3 Kinetic studies

Procedure: NMR timecourse experiments[1]

Spectrometer set-up: To an NMR tube was added a solution of ynamide (0.067 mmol, 1.0 equiv.) and 1,3,5-trimethoxybenzene (internal standard) in deuterated solvent (0.4 mL, 0.167 M). This 'dummy' sample was placed in the NMR spectrometer, with a probe temperature of 308K, for the purposes of preoptimizing the lock and shim of the reaction sample.

Timecourse experiment: To a nitrogen flushed NMR tube containing ynamide (0.067 mmol, 1.0 equiv.), bbeda (1.6 mg, 6.7 μmol, 0.1 equiv.), $Pd(OAc)_2$ (1.5 mg, 6.7 μmol, 0.1 equiv.) and 1,3,5-trimethoxybenzene (internal standard), was added d_8-toluene (0.4 mL, 0.167 M). The reaction mixture was briefly mixed on a vortex agitator, then rapidly placed in the NMR machine (probe temperature 308 K). Timecourse experiment spectra were collected at 34 s intervals (DS = 0, NS = 1, d1 = 20 s; at this relaxation time, the protons in the substrate and product had been independently confirmed to relax fully); 6s of delay time was added before the initiation of the next acquisition to allow for automated spectrometer processes. This equates to 34 seconds between each acquisition. The spectra were automatically phased, baseline corrected, and integrated (cross-checked against the internal standard peak) using the TOPSPIN multi_integ3 command. The integration values were plotted using Microsoft Excel as a percentage of the total starting material originally present (proportional to concentration).

3. Characterization of compounds

N-(2-Bromophenyl)-4-methylbenzenesulfonamide, 12

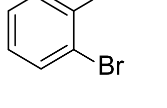

2-Bromoaniline (5.00 g, 29.2 mmol, 1.0 equiv.) was dissolved in CH_2Cl_2 (20 mL). To this stirred solution at room temperature was added *p*-tosylsulfonyl chloride (6.69 g, 35.1 mmol, 1.2 equiv.) and pyridine (7.42 mL, 87.7 mmol, 3.0 equiv.). The mixture was stirred at room temperature for 2 h, then it was diluted with H_2O (5 mL) and extracted with CH_2Cl_2 (3 x 25 mL). The combined organic phases were washed with 1 M HCl, brine, dried over Na_2SO_4, and concentrated. Silica gel flash

chromatography (pentane/EtOAc 5:1) afforded the title compound **12** (8.96 g, 27.5 mmol, 94%) as a yellow oil; **R$_f$** 0.23 (pentane/EtOAc 15:1); **^{1}H NMR** (400 MHz, $CDCl_3$) δ_H 7.58 (1H, d, *J* = 1.5 Hz), 7.57-7.52 (2H, m), 7.31 (1H, dd, *J* = 8.0, 1.4 Hz), 7.20-7.14 (1H, m), 7.11 (2H, d, *J* = 8.0 Hz), 6.88-6.83 (2H, m), 2.27 (3H, s); **^{13}C NMR** (100 MHz, $CDCl_3$) δ_C 144.4, 136.0, 134.9, 132.7, 129.8, 128.7, 127.5, 126.4, 122.7, 115.8, 21.7. Data identical to literature values.[6]

_N_-(2-(1-Hydroxycyclohexyl)phenyl)-4-methylbenzenesulfonamide, 13

4-Methyl-_N_-(2',3',4',5'-tetrahydro-[1,1'-biphenyl]-2-yl)benzenesulfonamide, 14

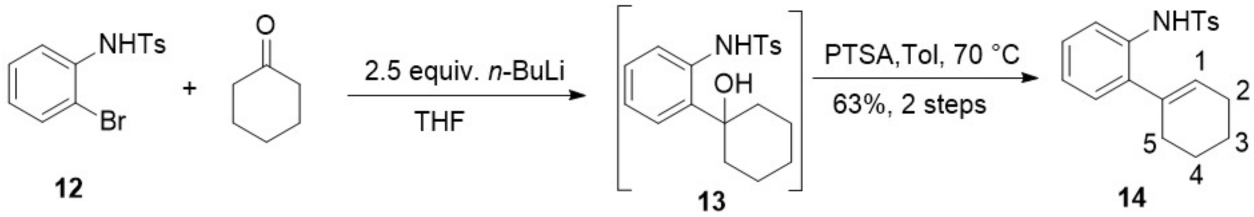

To a stirred solution of **12** (1.00 g, 3.08 mmol, 1.0 equiv.) in THF (8 mL) at -78 °C was added *n*-BuLi (2.5 M in hexane, 3.1 mL, 7.7 mmol, 2.5 equiv.) dropwise. The mixture was stirred for 15 min, then cyclohexanone (0.956 mL, 9.24 mmol, 3 equiv.) was added, and the resulting mixture was allowed to warm to 0 °C and stirred for 1 h. The reaction was then quenched by addition of saturated NH_4Cl solution (2 mL) and diluted with water. The phases were separated and the aqueous layer was extracted with EtOAc (3 × 10 mL). The combined organic phases were dried over Na_2SO_4 and concentrated. A sample of **13** could be obtained for characterisation through purification by flash chromatography (pentane/EtOAc 10:1), as a white solid; **R$_f$** 0.25 (pentane/EtOAc 10:1); **IR** (thin film, ν_{max} / cm^{-1}) 3478, 2927, 2854, 2361, 2340, 1495, 1451, 1333, 1158, 1091, 961, 939, 849, 755, 659; **mp** 140-142 °C; **^{1}H NMR** (400 MHz, $CDCl_3$) δ_H 9.71 (1H, s, N*H*), 7.74 (2H, d, *J* = 8.3 Hz, Ts*H*), 7.56 (1H, dd, *J* = 8.5, 1.3 Hz, Ar*H*), 7.24-7.20 (2H, m, Ts*H*), 7.18-7.14 (2H, m, Ar*H*), 7.03-6.91 (1H, m, Ar*H*), 2.36 (3H, s, TsC*H*$_3$), 1.83-1.76 (2H, m, Cy*H*), 1.73-1.70 (1H, m, Cy*H*), 1.68-1.55 (6H, m, Cy*H*), 1.21 (1H, d, *J* = 4.6 Hz, Cy*H*); **^{13}C NMR** (100 MHz, $CDCl_3$) δ_C 143.6, 137.6, 136.8, 135.3, 129.7, 128.3, 127.3, 127.3, 125.8, 123.5, 120.2, 75.8, 37.1, 25.3, 21.7, 21.6; **HRMS** (ES$^+$) calc. for $C_{19}H_{23}NNaO_3S$ $[M+Na]^+$ 368.1291, found 368.1289.

To a stirred solution of crude **13** (1.63 g, 4.72 mmol, 1.0 equiv.) in toluene (10 mL) was added *p*-toluenesulfonic acid monohydrate (179 mg, 0.94 mmol, 0.2 equiv.), then the mixture was heated

to 70 °C for 2 h. After total conversion, the mixture was cooled to room temperature and quenched with saturated NH_4Cl solution (2 mL). The mixture was diluted with water, the phases were separated and the aqueous layer extracted with EtOAc (3 × 10 mL). The combined organic phases were dried over Na_2SO_4 and concentrated. Silica gel flash chromatography (pentane/EtOAc 20:1) afforded the title compound **14** (636 mg, 1.945 mmol, 63%) as a white solid; **R$_f$** 0.12 (pentane/EtOAc 30:1); **IR** (thin film, ν_{max} / cm^{-1}) 3277, 2856, 2361, 1598, 1490, 1393, 1336, 1166, 1092, 905, 814, 757, 668, 629; **mp** 108-110 °C; **^{1}H NMR** (400 MHz, $CDCl_3$) δ_H 7.64 (1H, dd, *J* = 8.2, 1.2 Hz, Ar*H*), 7.60-7.56 (2H, m, Ts*H*), 7.21-7.17 (3H, m, 2×Ts*H*, Ar*H*), 7.04 (1H, td, *J* = 7.5, 1.2 Hz, Ar*H*), 6.95 (1H, dd, *J* = 7.6, 1.6 Hz, Ar*H*), 5.35 (1H, tt, *J* = 3.7, 1.7 Hz, H1), 2.36 (3H, s, TsC*H*$_3$), 2.12-2.08 (2H, m, H2), 1.72-1.66 (2H, m, H5), 1.61 (4H, t, *J* = 3.2 Hz, H3, H4); **^{13}C NMR** (100 MHz, $CDCl_3$) δ_C 143.9, 136.5, 136.0, 135.3, 133.2, 129.7, 128.8, 128.6, 127.8, 127.3, 124.6, 121.1, 30.2, 25.4, 22.9, 21.8, 21.6; **HRMS** (ES$^+$) calc. for $C_{19}H_{21}NNaO_2S$ [M+Na]$^+$ 350.1185, found 350.1183.

(*E*)-*N*-(1,2-Dichlorovinyl)-4-methyl-*N*-(2',3',4',5'-tetrahydro-[1,1'-biphenyl]-2-yl)benzenesulfonamide, 15

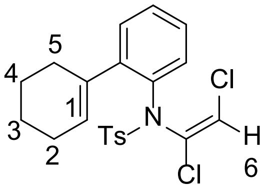

To a solution of **14** (580 mg, 1.77 mmol, 1.0 equiv.) and powdered Cs_2CO_3 (867 mg, 2.66 mmol, 1.5 equiv.) in DMF (4 mL) was added dropwise trichloroethylene (0.48 mL, 5.31 mmol, 3.0 equiv.), and the resulting mixture was stirred at 50 ºC for 2 h. The mixture was then cooled to room temperature and ethyl acetate and water (~2:1, 15 mL) were added. The layers were separated and the aqueous layer extracted with EtOAc (3 × 10 mL). The combined organic phases were dried over Na_2SO_4 and concentrated. Silica gel flash chromatography (pentane/EtOAc 30:1) afforded the title compound **15** (723 mg, 1.717 mmol, 97%) as a white solid; **R$_f$** 0.22 (pentane/EtOAc 50:1); **IR** (thin film, ν_{max} / cm^{-1}) 2929, 2361, 1597, 1485, 1438, 1368, 1289, 1168, 1083, 922, 814, 760, 659; **mp** 78-80 °C; **^{1}H NMR** (400 MHz, $CDCl_3$) δ_H 7.73 (2H, d, *J* = 8.3 Hz, Ts*H*), 7.33-7.28 (3H, m, 2×Ts*H*, Ar*H*), 7.24 (1H, dd, *J* = 8.7, 1.4 Hz, Ar*H*), 7.15 (2H, ddd, *J* = 7.6, 6.9, 1.7 Hz, Ar*H*), 6.32 (1H, s, H6), 5.30 (1H, dt, *J* = 3.8, 1.9 Hz, H1), 2.45 (3H, s, TsC*H*$_3$), 2.19-2.10 (2H, m, H2), 2.05 (2H, dt, *J*

= 6.2, 3.1 Hz, H5), 1.74-1.65 (2H, m, H3), 1.65-1.58 (2H, m, H4); **^{13}C NMR** (100 MHz, $CDCl_3$) δ_C 146.3, 144.9, 136.3, 135.6, 134.5, 131.6, 131.1, 129.6, 129.21, 129.19, 127.8, 126.5, 118.2, 30.2, 29.9, 25.6, 22.9, 21.8, 21.7; **HRMS** (ES^+) calc. for $C_{21}H_{21}Cl_2NNaO_2S$ $[M+Na]^+$ 444.0562, found 444.0562.

4-Methyl-*N*-(prop-1-yn-1-yl)-*N*-(2',3',4',5'-tetrahydro-[1,1'-biphenyl]-2-yl)benzenesulfonamide, 10

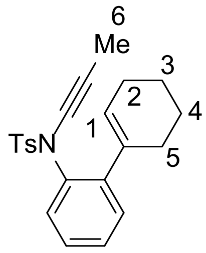

To a stirred solution of dichloroenamide **15** (482 mg, 1.14 mmol, 1.0 equiv.) in anhydrous THF (5 mL) at -78 °C was added PhLi (1.9 M in dibutyl ether, 1.68 mL, 3.20 mmol, 2.8 equiv.) dropwise, and the mixture was stirred for 1 h. After total conversion to the intermediate alkynyllithium (as confirmed by TLC), MeI (0.21 mL, 3.42 mmol, 3.0 equiv.) was added, then the mixture was allowed to warm to room temperature and stirred overnight. The reaction was quenched by addition of saturated NH_4Cl solution (2 mL), then diluted with water, the layers separated, and the aqueous phase extracted with EtOAc (3 × 10 mL). The combined organic phases were dried over Na_2SO_4 and concentrated. Silica gel flash chromatography (pentane/EtOAc 15:1) afforded the title compound **10** (376 mg, 1.03 mmol, 90%) as a yellow solid; **R_f** 0.27 (pentane/EtOAc 30:1); **IR** (thin film, ν_{max} / cm^{-1}) 2926, 2257, 1597, 1484, 1442, 1368, 1306, 1296, 1167, 939, 924, 848, 813, 759, 687, 657; **mp** 129-131 °C; **^{1}H NMR** (400 MHz, $CDCl_3$) δ_H 7.73 (2H, d, *J* = 8.3 Hz, Ts*H*), 7.36-7.31 (2H, m, Ts*H*), 7.28 (1H, dd, *J* = 7.0, 1.3 Hz, Ar*H*), 7.25 (1H, dd, *J* = 7.7, 1.9 Hz, Ar*H*), 7.14 (1H, dd, *J* = 1.9, 0.9 Hz, Ar*H*), 6.91 (1H, dd, *J* = 8.0, 1.3 Hz, Ar*H*), 5.74 (1H, dt, *J* = 3.8, 2.0 Hz, H1), 2.47 (3H, s, TsC*H*3), 2.42-2.31 (2H, m, H5), 2.22-2.13 (2H, m, H2), 1.88 (3H, s, H6), 1.81-1.73 (2H, m, H4), 1.71-1.60 (2H, m, H3); **^{13}C NMR** (100 MHz, $CDCl_3$) δ_C 144.7, 144.5, 136.6, 135.6, 135.0, 130.0, 129.5, 129.0, 128.5, 128.3, 128.0, 127.2, 74.1, 64.6, 29.4, 25.8, 23.2, 22.1, 21.8, 3.5; **HRMS** (ES^+) calc. for $C_{22}H_{23}NNaO_2S$ $[M+Na]^+$ 388.1342, found 388.1337.

(*Z*)-2'-Ethylidene-1'-tosylspiro[cyclohexane-1,3'-indolin]-2-ene, 16

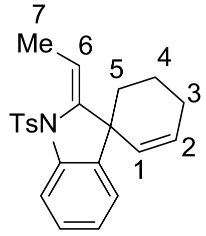

To a solution of ynamide **10** (36.5 mg, 0.1 mmol, 1.0 equiv.) in toluene (2 mL) was added $Pd(OAc)_2$ (2.3 mg, 10 μmol, 0.1 equiv.) and bbeda (2.4 mg, 10 μmol, 0.1 equiv.) and stirred overnight (12 h). The mixture was then concentrated and the product purified by column chromatography (pentane/EtOAc 15:1) to afford **16** (29.2 mg, 0.08 mmol, 80%) as a white solid; **R$_f$** 0.22 (pentane/EtOAc 50:1); **IR** (thin film, ν_{max} / cm^{-1}) 2919, 2361, 1459, 1362, 1168, 1090, 1032, 1013, 931, 919, 813, 755, 706, 660; **mp** 116-118 °C; **^{1}H NMR** (400 MHz, $CDCl_3$) δ_H 7.74 (1H, ddd, *J* = 8.1, 1.0, 0.5 Hz, Ar*H*), 7.52 (2H, d, *J* = 8.3 Hz, Ts*H*), 7.25-7.22 (1H, m, Ar*H*), 7.16-7.12 (2H, m, Ts*H*), 7.08 (1H, td, *J* = 7.5, 1.1 Hz, Ar*H*), 6.96 (1H, ddd, *J* = 7.5, 1.4, 0.5 Hz, Ar*H*), 5.92 (1H, dt, *J* = 9.9, 3.7 Hz, H1), 5.38 (1H, q, *J* = 7.2 Hz, H6), 5.30-5.22 (1H, m, H2), 2.33 (3H, s, TsCH_3), 2.02 (3H, d, *J* = 7.2 Hz, H7), 2.00-1.96 (2H, m, H3), 1.44 (1H, dddt, *J* = 13.7, 10.5, 7.2, 3.3 Hz, H4), 1.33 (1H, ddd, *J* = 10.3, 4.6, 2.1 Hz, H4), 0.71 (1H, ddd, *J* = 13.3, 10.2, 3.0 Hz, H5), 0.51 (1H, ddd, *J* = 13.2, 7.5, 2.3 Hz, H5); **^{13}C NMR** (100 MHz, $CDCl_3$) δ_C 145.7, 144.3, 141.8, 140.9, 135.4, 131.1, 129.6, 129.3, 128.1, 127.8, 125.6, 123.9, 119.9, 118.6, 50.5, 35.8, 24.5, 21.6, 18.0, 15.6; **HRMS** (ES$^+$) calc. for $C_{22}H_{23}NNaO_2S$ $[M+Na]^+$ 388.1342, found 388.1338.

2-(4,4,5,5-Tetramethyl-1,3,2-dioxaborolan-2-yl)aniline, 17

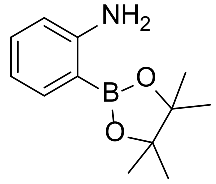

To a stirred mixture of 2-bromoaniline (1.00 g, 5.81 mmol, 1.0 equiv.), Et_3N (3.23 mL, 23.2 mmol, 4.0 equiv.), $Pd(dppf)Cl_2$ (213 mg, 0.29 mmol, 0.05 equiv.) in 1,4-dioxane (25 mL) was added dropwise 4,4,5,5-tetramethyl-1,3,2-dioxaborolane (2.53 mL, 17.4 mmol, 3.0 equiv.). The resultant mixture was heated to 100 °C. After 12 h, the reaction was cooled to room temperature, then quenched with saturated NH_4Cl solution (2 mL). The mixture was diluted with water, then the phases were separated and the aqueous phases extracted with CH_2Cl_2 (2 × 20 mL). The combined

organic phases were dried over Na_2SO_4 and concentrated. Silica gel flash chromatography (pentane/EtOAc 15:1) afforded the title compound **17** (1.07 g, 4.88 mmol, 84%) as a yellow solid; **R_f** 0.23 (pentane/EtOAc 30:1); **^{1}H NMR** (400 MHz, $CDCl_3$) δ_H 7.63 (1H, dd, *J* = 7.4, 1.7 Hz), 7.22 (1H, ddd, *J* = 8.1, 7.2, 1.7 Hz), 6.68 (1H, td, *J* = 7.3, 1.0 Hz), 6.63-6.56 (1H, m), 4.74 (2H, s), 1.35 (12H, s); **^{13}C NMR** (100 MHz, $CDCl_3$) δ_C 153.7, 136.8, 132.8, 116.9, 114.8, 83.5, 24.9. Data identical to literature values.[7]

Cyclohex-1-en-1-yl trifluoromethanesulfonate, 18

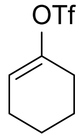

To a stirred solution of cyclohexanone (1.00 g, 10.2 mmol, 1.0 equiv.) in CH_2Cl_2 (30 mL) at 0 °C was added 2,6-di-tert-butyl-4-methylpyridine (2.14 g, 11.2 mmol, 1.1 equiv.) and trifluoromethanesulfonic anhydride (2.56 g, 12.2 mmol, 1.2 equiv.). The reaction mixture was warmed to room temperature, stirred overnight, and then concentrated. Pentane was added, and the solid pyridinium triflate was filtered off (washed with pentane). The combined pentane solutions were washed subsequently with cold HCl (1 M), saturated brine, and then dried over Na_2SO_4 and concentrated. Silica gel flash chromatography (pentane) afforded the title compound **17** (1.41 g, 6.13 mmol, 60%) as a purple oil; **R_f** 0.17 (pentane); **^{1}H NMR** (400 MHz, $CDCl_3$) δ 5.76 (1H, td, *J* = 4.0, 2.0 Hz), 2.31 (2H, tq, *J* = 5.2, 2.1 Hz), 2.18 (2H, dtt, *J* = 6.1, 3.8, 2.0 Hz), 1.84-1.72 (2H, m), 1.66-1.51 (2H, m); **^{13}C NMR** (100 MHz, $CDCl_3$) δ_C 149.5, 118.6 (q, *J* = 320 Hz), 117.1, 27.7, 24.0, 22.8, 21.1. Data identical to literature values.[8,9]

2',3',4',5'-Tetrahydro-[1,1'-biphenyl]-2-amine, 19

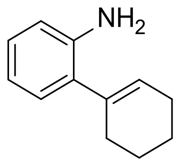

To a degassed solution of **17** (916 mg, 4.18 mmol, 1.1 equiv.), K_2CO_3 (2.10 g, 15.2 mmol, 4.0 equiv.) and $Pd(PPh_3)_4$ (440 mg, 0.38 mmol, 0.1 equiv.) in PhMe/EtOH/H_2O (5:2:1, ~0.05 M, 76 mL) under an argon atmosphere was added triflate **18** (875 mg, 3.8 mmol, 1.0 equiv.), and the resulting mixture was stirred at 100 °C for 10 h. The reaction was cooled to room temperature, then H_2O and

CH_2Cl_2 were added and the phases were separated. The aqueous layer was extracted with CH_2Cl_2 (3 x 40 mL). The combined organic layers were washed with brine, dried over Na_2SO_4 and concentrated. Silica gel flash chromatography (pentane/EtOAc 50:1) afforded the title compound **19** (478 mg, 2.76 mmol, 73%) as a yellow oil; **R$_f$** 0.23 (pentane/EtOAc 30:1); **^{1}H NMR** (400 MHz, $CDCl_3$) δ_H 7.05 (1H, td, *J* = 7.7, 1.6 Hz), 6.99 (1H, dd, *J* = 7.5, 1.5 Hz), 6.81-6.62 (2H, m), 5.77 (1H, tt, *J* = 3.6, 1.7 Hz), 3.77 (2H, s), 2.32-2.23 (2H, m), 2.19 (2H, ddt, *J* = 9.8, 6.2, 3.1 Hz), 1.81-1.76 (2H, m), 1.73-1.67 (2H, m); **^{13}C NMR** (100 MHz, $CDCl_3$) δ_C 143.2, 136.6, 130.6, 128.7, 127.6, 126.9, 118.4, 115.5, 29.5, 25.6, 23.3, 22.3. Data identical to literature values.[9]

Methyl (2',3',4',5'-tetrahydro-[1,1'-biphenyl]-2-yl)carbamate, 20

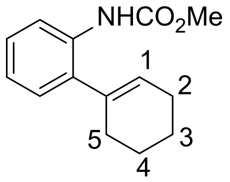

To a stirred solution of **19** (300 mg, 1.73 mmol, 1.0 equiv.) and pyridine (0.35 mL, 4.33 mmol, 2.5 equiv.) in CH_2Cl_2 (3 mL) at 0 °C was added dropwise methyl chloroformate (197 mg, 2.08 mmol, 1.2 equiv.). The mixture was stirred for 1 h, during which time the mixture was allowed to warm to room temperature. The reaction was quenched by addition of water and diluted with CH_2Cl_2. The organic phase was separated and washed with 1 N HCl, saturated $NaHCO_3$, brine, dried over Na_2SO_4 and concentrated. Silica gel flash chromatography (pentane/EtOAc 50:1) afforded the title compound **19** (328 mg, 1.42 mmol, 82%) as a yellow oil; **R$_f$** 0.22 (pentane/EtOAc 50:1); **IR** (thin film, ν_{max} / cm^{-1}) 3410, 2931, 2360,1741, 1584, 1519, 1448, 1304, 1221, 1202, 1067, 1044, 954, 920, 766, 756; **^{1}H NMR** (400 MHz, $CDCl_3$) δ_H 8.04 (1H, d, *J* = 7.4 Hz, N*H*), 7.23 (1H, td, *J* = 7.9, 1.7 Hz, Ar*H*), 7.08-6.99 (2H, m, Ar*H*), 6.94 (1H, s, Ar*H*), 5.77-5.70 (1H, m, H1), 3.77 (3H, s, CO_2CH_3), 2.21 (2H, s, H5), 2.19 (2H, dd, *J* = 6.4, 2.3 Hz, H2), 1.82-1.75 (2H, m, H4), 1.74-1.66 (2H, m, H3); **^{13}C NMR** (100 MHz, $CDCl_3$) δ_C 154.1, 135.8, 134.5, 133.8, 128.6, 128.4, 127.7, 123.0, 119.0, 52.3, 30.1, 25.5, 23.1, 22.1; **HRMS** (ES$^+$) calc. for $C_{12}H_{17}NO_2$ [M+Na]$^+$ 232.1332, found 232.1334.

Methyl (*E*)-(1,2-dichlorovinyl)(2',3',4',5'-tetrahydro-[1,1'-biphenyl]-2-yl)carbamate, 21

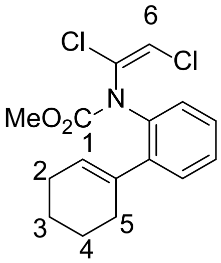

To a stirred suspension of NaH (60 % dispersion in mineral oil, 93 mg, 2.31 mmol, 2.2 equiv.) in anhydrous DMF (2 mL) at 0 °C was added a solution of **20** (242 mg, 1.05 mmol, 1.0 equiv.) in anhydrous DMF (1.5 mL) dropwise. The mixture was allowed to warm to room temperature and stirred for a further 1 h. Trichloroethylene (0.104 mL, 1.16 mmol, 1.1 equiv.) was then added dropwise at room temperature, then the mixture become black and was stirred at room temperature for additional 1 h. The reaction was quenched by addition of saturated NH_4Cl solution (1 mL), then the mixture was diluted with water. The layers were separated and the aqueous layer extracted with EtOAc (3 × 10 mL). The combined organic phases were dried over Na_2SO_4 and concentrated. Silica gel flash chromatography (pentane/EtOAc 50:1) afforded the title compound **21** (255 mg, 0.78 mmol, 75%) as a yellow oil; **R_f** 0.23 (pentane/EtOAc 50:1); **IR** (thin film, ν_{max} / cm^{-1}) 3090, 2929, 1737, 1615, 1520, 1488, 1437, 1309, 1263, 1239, 1195, 1138, 1020, 827, 758; **^{1}H NMR** (400 MHz, $CDCl_3$) δ_H 7.32-7.28 (1H, m, Ar*H*), 7.26 (1H, dd, *J* = 5.5, 1.9 Hz, Ar*H*), 7.23-7.21 (2H, m, Ar*H*), 6.27 (1H, s, H6), 5.82 (1H, dt, *J* = 3.6, 2.0 Hz, H1), 3.79 (3H, s, CO_2CH_3), 2.28 (2H, br, H5), 2.16-2.08 (2H, m, H2), 1.74 (2H, dt, *J* = 10.1, 5.1 Hz, H4), 1.64 (2H, p, *J* = 6.0 Hz, H3); **^{13}C NMR** (100 MHz, $CDCl_3$) δ_C 142.7, 135.2, 135.1, 131.6, 129.8, 128.7, 128.4, 128.2, 127.7, 127.3, 114.2, 54.0, 29.2, 25.6, 23.2, 22.0; **HRMS** (ES^+) calc. for $C_{16}H_{18}Cl_2NO_2$ $[M+H]^+$ 326.0709, found 326.0710.

Methyl prop-1-yn-1-yl(2',3',4',5'-tetrahydro-[1,1'-biphenyl]-2-yl)carbamate, 11

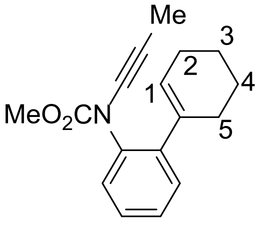

Synthesized according to a literature procedure.[10] To a stirred solution of **21** (205 mg, 0.631 mmol,

1.0 equiv.) in anhydrous TBME (6.3 mL) at 0 °C was added LiHMDS (1.0 M in toluene, 0.76 mL, 0.757 mmol, 1.2 equiv.) dropwise. The reaction stirred at 0 °C for 1 hour until elimination was complete as analysed by TLC. The cooling bath was then removed and the reaction mixture was allowed to warm to room temperature. To the mixture was added CuCN·2LiCl (32 μL of a 1.0 M solution in THF, 32 μmol, 5 mol%), followed by Me_2Zn solution (1.2 M in toluene, 1.58 mL, 1.89 mmol, 3 equiv.) dropwise, and the reaction was stirred for 30 minutes at room temperature, until complete as judged by TLC. The reaction was then quenched by addition of aqueous saturated NH_4Cl (2 mL), the mixture was diluted with water, the layers were separated and the aqueous layer extracted with EtOAc (3 × 10 mL). The combined organic phases were dried over Na_2SO_4 and concentrated. Silica gel flash chromatography (pentane/EtOAc 50:1) afforded the title compound **11** (82 mg, 0.305 mmol, 48%) as a yellow oil; **R_f** 0.21 (pentane/EtOAc 30:1); **IR** (thin film, ν_{max} / cm^{-1}) 2927, 2856, 1737, 1520, 1487, 1313, 1210, 1064, 1039, 927, 844, 757; **^{1}H NMR** (400 MHz, $CDCl_3$) δ_H 7.32-7.27 (2H, m, Ar*H*), 7.27-7.25 (1H, m, Ar*H*), 7.24-7.21 (1H, m, Ar*H*), 5.74 (1H, dt, *J* = 3.8, 2.0 Hz, H1), 3.77 (3H, s, CO_2CH_3), 2.28 (2H, br, H5), 2.16 (2H, app. q, *J* = 4.5 Hz, H2), 1.91 (3H, s, H6), 1.80-1.71 (2H, m, H4), 1.72-1.63 (2H, m, H3); **^{13}C NMR** (100 MHz, $CDCl_3$) δ_C 155.8, 142.2, 137.1, 135.3, 129.4, 128.3, 127.6, 127.5, 127.3, 74.0, 64.4, 54.1, 29.0, 25.7, 23.3, 22.1, 3.5; **HRMS** (ES+) calc. for $C_{17}H_{20}NO_2$ $[M+H]^+$ 270.1489, found 270.1487.

Methyl (*Z*)-2'-ethylidenespiro[cyclohexane-1,3'-indolin]-2-ene-1'-carboxylate, 22a

Methyl (*E*)-2'-ethylidenespiro[cyclohexane-1,3'-indolin]-2-ene-1'-carboxylate, 22b

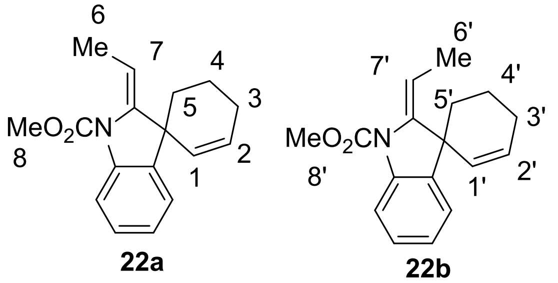

22a **22b**

To a solution of ynamide **11** (27 mg, 0.1 mmol, 1.0 equiv.) in toluene (1 mL) was added $Pd(OAc)_2$ (2.3 mg, 0.01 mmol, 0.1 equiv.) and bbeda (2.4 mg, 0.01 mmol, 0.1 equiv.). and stirred overnight (12 h). The mixture concentrated and the residue was purified by column chromatography (pentane/EtOAc 50:1) to afford **22a and 22b** (19 mg, 0.07 mmol, 70 %) as a white solid; **R_f** 0.17 (pentane/EtOAc 50:1); **IR** (thin film, ν_{max} / cm^{-1}) 3022, 2932, 1711, 1599, 1476, 1463, 1439, 1349,

1314, 1281, 1230, 1199, 1105, 1088, 750, 688, 641; **^{1}H NMR** (400 MHz, $CDCl_3$) δ_H 7.69 (1H, dt, *J* = 8.1, 0.8 Hz, 1H, Ar*H'*), 7.64 (2.5H, dt, *J* = 8.1, 0.8 Hz, Ar*H*), 7.24-7.19 (4H, m, Ar*H* + Ar*H'*), 7.15 (1H, dd, *J* = 7.4, 1.4 Hz, Ar*H'*), 7.11-7.01 (6H, m, Ar*H*+ Ar*H'*), 6.28 (1H, d, *J* = 7.5 Hz, H7'), 6.09 (2.5H, dt, *J* = 10.0, 3.7 Hz, H1), 6.01-5.93 (1H, m, H1'), 5.65 (1H, ddt, *J* = 10.1, 2.8, 1.5 Hz, H2'), 5.46 (2.5H, dtd, *J* = 10.0, 2.1, 0.9 Hz, H2), 5.13 (2.5H, q, H7), 3.91 (3H, s, H8'), 3.88 (7.5H, s, H8), 2.24-2.17 (2H, m, H3'), 2.15-2.10 (5H, m, H3), 2.07-1.98 (1H, m, H5'), 1.94-1.88 (1H, m, H4'), 1.86 (3H, d, *J* = 7.5 Hz, H6'), 1.81-1.68 (9.5H, m, 2 × H4, H5, H5', H4'), 1.66 (7.5H, d, *J* = 7.1 Hz, H6), 1.60-1.54 (2.5H, m, H5); **^{13}C NMR** (100 MHz, $CDCl_3$) δ_C 153.9, 153.4, 145.8, 145.7, 142.1, 139.4, 137.2, 130.8, 130.6, 129.5, 127.8, 127.7, 127.5, 124.9, 124.1, 123.3, 123.1, 116.7, 116.0, 111.8, 108.5, 53.1, 52.8, 49.7, 48.2, 36.2, 34.9, 24.9, 24.3, 18.5, 18.3, 14.5, 12.3; **HRMS** (ES^+) calc. for $C_{17}H_{20}NO_2$ $[M+Na]^+$ 270.1789, found 270.1788.

3-Cyano-2,3,4,5-tetrahydro-[1,1'-biphenyl]-2-yl acetate, 25

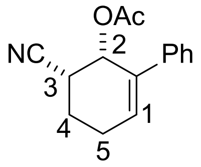

2-Phenyl-but-2-enaldehyde **24** (5.00 g, 34.2 mmol, 1.0 equiv.), acetic anhydride (6.47 mL, 68.4 mmol, 2.0 equiv.), acrylonitrile (18 mL, 273.6 mmol, 8.0 equiv.) and *p*-TsOH.H_2O (195 mg, 1.02 mmol, 0.03 equiv.) were combined in a pressure tube, and toluene (30 mL) was added. The reaction mixture was heated to 110 °C for 1.5 days. The mixture was then cooled, and concentrated. Silica gel flash chromatography (pentane/EtOAc 6:1) afforded the title compound **25** (4.95 g, 20.5 mmol, 60%) as a yellow solid; **R_f** 0.2 (pentane/EtOAc 6:1); **IR** (thin film, ν_{max} / cm^{-1}) 2941, 2244, 1743, 1497, 1447, 1371, 1223, 1100, 1014, 926, 760, 699; **mp** 90-92 °C; **^{1}H NMR** (400 MHz, $CDCl_3$) δ_H 7.30-7.26 (3H, m, Ar*H*), 7.25-7.20 (2H, m, Ar*H*), 6.21 (1H, t, *J* = 4.1 Hz, H1), 6.19 (1H, d, *J* = 4.4 Hz, H2), 3.11 (1H, dt, *J* = 9.9, 4.0 Hz, H3), 2.52-2.42 (1H, m, H5), 2.33-2.24 (1H, m, H5), 2.11-2.03 (2H, m, H4), 1.96 (3H, s, COC*H*$_3$); **^{13}C NMR** (100 MHz, $CDCl_3$) δ_C 170.3, 138.2, 134.9, 129.8, 128.6, 127.9, 126.1, 119.2, 64.7, 32.0, 24.2, 22.0, 20.8; **HRMS** (ES^+) calc. for $C_{15}H_{15}NNaO_2$ $[M+Na]^+$ 264.0995, found 264.0995. The stereochemistry of the product was assigned by analogy to the literature[2].

2-Cyano-6-phenylcyclohexyl acetate, S1

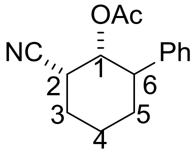

To a solution of **25** (1.00 g, 4.15 mmol, 1.0 equiv.) in THF (10 mL) was added 10% Pd/C (1.73 g, 1.62 mmol, 0.39 equiv.). The mixture was purged with hydrogen using a hydrogen balloon and then stirred under hydrogen atmosphere at room temperature for 1 h, then it was filtered through a short plug of Celite, and the filtrate was concentrated to give **S1** (987 mg, 4.06 mmol, 98%) as yellow oil that required no further purification; $\mathbf{R_f}$ 0.2 (pentane/EtOAc 6:1); **IR** (thin film, ν_{max} / cm^{-1}) 2942, 2868, 2359, 2242, 1747, 1449, 1374, 1223, 1132, 1017, 762,701; **^{1}H NMR** (400 MHz, $CDCl_3$) δ 7.32-7.22 (3H, m, Ar*H*), 7.20-7.16 (2H, m, Ar*H*), 5.59 (1H, s, H1), 2.88 (1H, ddd, *J* = 12.6, 4.4, 2.5 Hz, H2), 2.77 (1H, dt, *J* = 12.9, 3.0 Hz, H6), 2.10-2.01 (4H, m, 2×H2, 1×H4, 1×H5), 1.98 (3H, s, $COCH_3$), 1.77 (1H, dd, *J* = 9.9, 3.1 Hz, H5), 1.48 (1H, dt, *J* = 12.8, 3.4 Hz, H4); **^{13}C NMR** (100 MHz, $CDCl_3$) 169.0, 140.9, 128.5, 127.7, 127.3, 119.7, 69.6, 45.9, 33.7, 24.8 (2C), 24.4, 20.6; **HRMS** (ES^+) calc. for $C_{15}H_{17}NNaO_2$ $[M+Na]^+$ 266.1152, found 266.1150. The stereochemistry at H6 could not be assigned, but is lost in a subsequent step.

***N*-((2-Hydroxy-3-phenylcyclohexyl)methyl)-4-methylbenzenesulfonamide, 26**

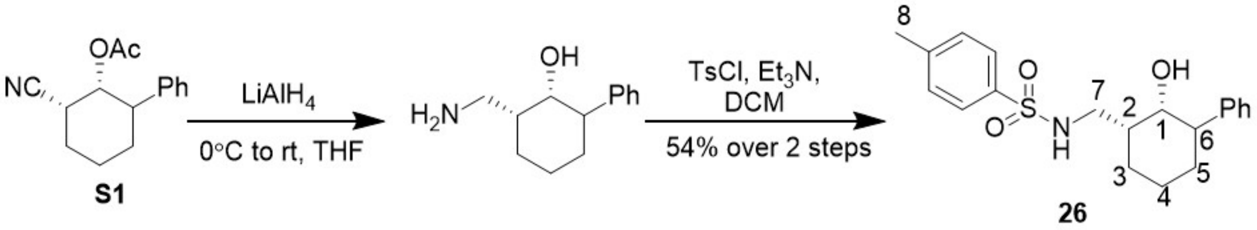

To a solution of **S1** (560 mg, 2.30 mmol, 1.0 equiv.) in anhydrous THF (7 mL) at 0 °C was added lithium aluminium hydride powder (524 mg, 13.8 mmol, 6.0 equiv.) cautiously in three portions, and the reaction was allowed to stir at room temperature overnight. The mixture was then cooled to 0 °C and carefully quenched by the dropwise addition of water (0.53 mL), 15% NaOH (0.53 mL), and water (1.06 mL) under vigorous stirring. The resulting suspension was filtered, and the filtrate was concentrated to furnish the crude amine (479 mg) which was used directly in the next step without further purification.

To a stirred solution of this crude residue (479 mg, 2.33 mmol, 1.0 equiv.) in CH_2Cl_2 (6 mL) at 0 °C

was added Et_3N (0.98 mL, 7.00 mmol, 3.0 equiv.) and *p*-toluenesulfonyl chloride (1.33 g, 7.00 mmol, 3.0 equiv.), and the mixture was allowed to warm to room temperature. After stirring for an additional 1 hour, the reaction was quenched by addition of saturated aqueous NH_4Cl (1 mL). The mixture was diluted with water (3 mL), the organic layer was separated and the aqueous layer was extracted with EtOAc (3×10 mL). The combined organic layers were washed with brine, dried over anhydrous Na_2SO_4 and concentrated. The residue was purified by flash chromatography on silica gel (pentane/EtOAc 10:1→5:1) to give the title compound **26** (457 mg, 1.27 mmol, 54%) as a yellow oil; **R$_f$** 0.23 (pentane/EtOAc 5:1); **IR** (thin film, ν_{max} / cm^{-1}) 3540, 3284, 2928, 2859, 1599, 1496, 1448, 1322, 1157, 1094, 976, 814, 701, 663; **^{1}H NMR** (400 MHz, $CDCl_3$) δ_H 7.74-7.69 (2H, m, Ts*H*), 7.29 (4H, 7.34-7.27, m, 2×Ts*H*, 2×Ar*H*), 7.25-7.18 (3H, m, Ar*H*), 5.15-5.01 (1H, m, N*H*), 3.92 (1H, s, H1), 3.05 (1H, dt, *J* = 12.9, 7.9 Hz, H7), 2.90 (1H, dt, *J* = 12.9, 5.0 Hz, H7), 2.64 (1H, dt, *J* = 13.0, 2.8 Hz, H6), 2.41 (3H, s, H8), 1.98 (1H, td, *J* = 12.7, 3.6 Hz, H5), 1.92-1.85 (1H, m, H4), 1.79 (1H, ddt, *J* = 11.4, 7.7, 3.7 Hz, H2), 1.61 (1H, dd, *J* = 13.1, 3.2 Hz, H5), 1.49-1.40 (2H, m, H3), 1.39-1.32 (1H, m, H4); **^{13}C NMR** (100 MHz, $CDCl_3$) δ_C 143.33, 143.27, 137.3, 129.8, 128.7, 127.9, 127.2, 126.8, 71.7, 48.4, 46.4, 42.0, 25.5, 24.0, 23.4, 21.6; **HRMS** (ES$^+$) calc. for $C_{20}H_{25}NNaO_3S$ [M+Na]$^+$ 382.1447, found 382.1441.

4-Methyl-*N*-((3,4,5,6-tetrahydro-[1,1'-biphenyl]-3-yl)methyl)benzenesulfonamide, S2a[3]
4-Methyl-*N*-((1,4,5,6-tetrahydro-[1,1'-biphenyl]-3-yl)methyl)benzenesulfonamide, S2b

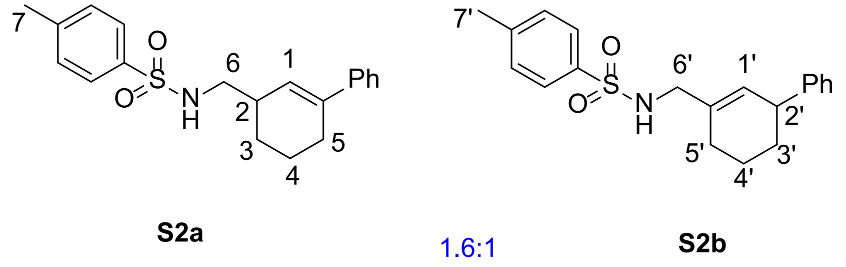

To a solution of **26** (230 mg, 0.64 mmol, 1.0 equiv.) in toluene (5 mL) was added Burgess reagent (183 mg, 0.77 mmol, 1.2 equiv.), then the mixture was heated for 1 h at 70 °C. After cooling to room temperature, the resultant mixture was directly purified using flash column chromatography (pentane/EtOAc 5:1) to give **S2a** and **S2b** (196 mg, 0.57 mmol, 90%) as a yellow oil; **R$_f$** 0.2 (pentane/EtOAc 10:1); **IR** (thin film, ν_{max} / cm^{-1}) 3282, 2925, 2859, 2360, 2342, 1599, 1494, 1446, 1429, 1324, 1158, 1094, 814, 754, 699, 666; **^{1}H NMR** (400 MHz, $CDCl_3$) δ_H 7.83-7.67 (5.2H, m, 2

x Ts*H*+2 x Ts*H'*), 7.32-7.27 (14H, m, 2 x Ts*H*+2 x Ts*H'* +4 x Ar*H'*+3 x Ar*H*), 7.25-7.18 (2.6H, m, Ar*H*+Ar*H'*), 7.11-7.09 (1.6H, m, Ar*H*), 5.81 (1.6H, dd, *J* = 2.2, 1.2 Hz, H1), 5.59 (1H, dd, *J* = 2.9, 1.6 Hz, H1'), 4.60-4.52 (1.6H m, N*H*), 4.51 (1H, d, *J* = 5.9 Hz, N*H'*), 3.53 (2H, dt, *J* = 6.4, 1.4 Hz, H6'), 3.30 (1H, br, H2'), 3.07-2.86 (3.2H, m, H6), 2.48-2.43 (1.6H, m, H2), 2.425 (3H, s, H7), 2.419 (4.8H, s, H7'), 2.37 (3.2H, tdt, *J* = 4.7, 2.8, 1.8 Hz, H5), 2.02-1.96 (2H, m, H5'), 1.94 (1H, td, *J* = 6.4, 4.3 Hz, H3'), 1.91-1.86 (1.6H, m, H4), 1.82-1.77 (1.6H, m, H3), 1.77-1.71 (1H, m, H4'), 1.67-1.59 (1.6H, m, H4), 1.56-1.50 (1H, m, H4'), 1.49-1.42 (1H, m, H3'), 1.37-1.30 (1.6H, m, H3); **^{13}C NMR** (101 MHz, $CDCl_3$) δ_C 146.0, 143.5, 141.9, 139.8, 137.2, 137.1, 134.5, 129.9, 129.8, 128.5, 128.4, 128.4, 128.3, 127.7, 127.3, 127.2, 126.3, 125.3, 125.1, 124.7, 49.7, 48.2, 42.0, 36.3, 34.5, 32.1, 27.6, 26.3, 26.1, 21.7, 21.5, 21.3; **HRMS** (ES^+) calc. for $C_{20}H_{24}NO_2S$ $[M+H]^+$ 342.1522, found 342.1525.

4-Methyl-*N*-((3,4,5,6-tetrahydro-[1,1'-biphenyl]-3-yl)methyl)benzenesulfonamide, 27

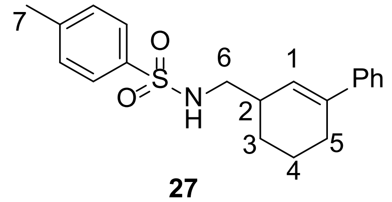

27

Synthesized according to a literature procedure.[4] To a solution of **S2a** and **S2b** (276 mg, 0.81 mmol, 1.0 equiv.) in anhydrous DMF (3 mL) was added KO*t*-Bu (1.0 M in THF, 2.43 mL, 2.43 mmol, 3.0 equiv.), then the mixture was heated for 1 h at 60 °C. After cooling to room temperature, the reaction was quenched by addition of saturated NH_4Cl solution (1 mL). Then H_2O (2 mL) was added to this mixture, the phases were separated and the aqueous phase was extracted with EtOAc (3 × 10 mL). The combined organic phases were dried over Na_2SO_4 and concentrated. Silica gel flash chromatography (pentane/EtOAc (10:1)) afforded the title compound **27** (196 mg, 0.575 mmol, 71%) as yellow oil; **R_f** 0.2 (pentane/EtOAc (10:1); **IR** (thin film, ν_{max} / cm^{-1}) 3282, 2925, 2859, 2360, 2342, 1598, 1494, 1445, 1429, 1323, 1094, 1070, 840, 753, 696, 665; **^{1}H NMR** (400 MHz, $CDCl_3$) δ_H 7.76 (2H, d, *J* = 8.3 Hz, Ts*H*), 7.34-7.27 (6H, m, 2×Ts*H*, 4×Ar*H*), 7.25-7.19 (1H, m, Ar*H*), 5.82-5.81 (m, 1H, H1), 4.64 (1H, s, N*H*), 3.04-2.89 (2H, m, H6), 2.45 (1H, dd, *J* = 6.4, 3.1 Hz, H2), 2.42 (3H, s, $TsCH_3$), 2.40-2.35 (2H, m, H5), 1.91-1.84 (1H, m, H4), 1.80 (1H, dtd, *J* = 12.3, 5.8, 2.6 Hz, H3), 1.68-1.58 (1H, m, H4), 1.40-1.23 (1H, m, H3); **^{13}C NMR** (100 MHz, $CDCl_3$)

δ_C 143.5, 141.9, 139.7, 137.1, 129.8, 128.4, 127.2 (2C), 125.3, 124.7, 48.2, 36.3, 27.6, 26.1, 21.7, 21.5; **HRMS** (ES^+) calc. for $C_{20}H_{23}NNaO_2S$ $[M+Na]^+$ 364.1342, found 364.1336.

((3-Bromoprop-2-yn-1-yl)oxy)(tert-butyl)diphenylsilane, 28

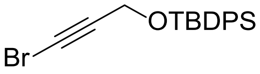

To a stirred solution of propynol (1.00 g, 17.8 mmol, 1.0 equiv.) and imidazole (2.43 g, 35.6 mmol, 2.0 equiv.) in CH_2Cl_2 (50 mL) at 0 °C was added TBDPSCl (6.94 mL, 26.7 mmol, 1.5 equiv.). The mixture was allowed to warm to room temperature, and stirred for 4 h. Water (20 mL) was added to the mixture, which was then extracted with EtOAc (20 mL x 3). The combined organic phases were washed with brine (20 mL) and dried over Na_2SO_4, filtered, and concentrated. The light-yellow oily product was directly used for the next step without further purification.

The crude silyl ether was dissolved in acetone (80 mL) at room temperature. Then NBS (3.81 g, 21.4 mmol, 1.2 equiv.) and $AgNO_3$ (605 mg, 3.56 mmol, 0.2 equiv.) were added. The mixture was stirred, shielded from light, for 6 h. The mixture was then concentrated and the residue was purified by flash column chromatography (pentane) to afford the title compound **28** (5.83 g, 15.7 mmol, 88%); **R_f** 0.3 (pentane); **^{1}H NMR** (400 MHz, $CDCl_3$) δ_H 7.71-7.69 (4H, m, Ar*H*), 7.47-7.38 (6H, m, Ar*H*), 4.33 (2H, s, C*H*$_2$), 1.06 (9H, s, C(C*H*$_3$)$_3$); **^{13}C NMR** (100 MHz, $CDCl_3$) δ_C 135.8, 133.0, 130.0, 127.9, 78.5, 53.6, 45.1, 26.8, 19.3. Data identical to literature values.[5]

***N*-(3-((*tert*-butyldiphenylsilyl)oxy)prop-1-yn-1-yl)-4-methyl-*N*-((3,4,5,6-tetrahydro-[1,1'-biphenyl]-3-yl)methyl)benzenesulfonamide, 29**

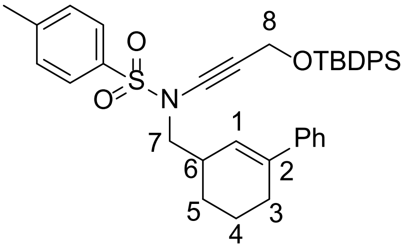

To a stirred solution of **27** (182 mg, 0.53 mmol, 1.0 equiv.) and bromoalkyne **28** (238 mg, 0.64 mmol, 1.2 equiv.) in dry toluene (3 mL) was added $CuSO_4 \cdot 5H_2O$ (27.5 mg, 0.11 mmol, 0.2 equiv.), 1,10-phenanthroline (39.6 mg, 0.22 mmol, 0.4 equiv.) and K_3PO_4 (337 mg, 1.59 mmol, 3.0 equiv.). The mixture was shielded from light, and heated to 75 °C for 12 h (overnight). The reaction was

cooled to room temperature, and filtered through a Celite pad (washed with EtOAc). The filtrate was concentrated and the residue purified by flash column chromatography (pentane/EtOAc, 30:1) to afford **29** (291 mg, 0.46 mmol, 86%); **R$_f$** 0.27 (pentane/EtOAc 30:1); **IR** (thin film, ν_{max} / cm^{-1}) 2929, 2857, 2241, 1597, 1428, 1362, 1168, 1110 , 1070, 998, 822, 701, 610; **^{1}H NMR** (400 MHz, $CDCl_3$) δ_H 7.79 (2H, d, *J* = 8.3 Hz, Ar*H*), 7.75-7.67 (4H, m, Ar*H*), 7.45-7.35 (9H, m, Ar*H*), 7.34-7.27 (4H, m, Ar*H*), 5.92-5.77 (1H, m, H1), 4.50 (2H, s, H8), 3.33-3.08 (2H, m, H7), 2.65 (1H, app. s, H6), 2.58-2.35 (2H, m, H3), 2.43 (3H, s, TsCH_3), 1.94-1.85 (1H, m, H4), 1.81 (1H, dq, *J* = 12.0, 3.3 Hz, H5), 1.69 (1H, ddd, *J* = 12.5, 6.0, 2.6 Hz, H4), 1.38 (1H, ddd, *J* = 8.1, 6.4, 3.9 Hz, H5), 1.06 (9H, s, C(CH_3)$_3$); **^{13}C NMR** (100 MHz, $CDCl_3$) δ_C 144.6, 142.1, 139.2, 135.7, 134.9, 134.8, 133.39, 133.37, 129.9, 129.8, 128.3, 127.83, 127.81, 127.7, 127.1, 125.3, 125.2, 124.4, 78.8, 70.0, 56.0, 53.1, 34.8, 27.7, 26.8, 25.8, 21.8, 21.2, 19.3; **HRMS** (ES$^+$) calc. for $C_{39}H_{43}NNaO_3SSi$ [M+Na]$^+$ 656.2625, found 656.2619.

(3a*S*,7a*S*,*Z*)-1-(2-((*tert*-butyldiphenylsilyl)oxy)ethylidene)-7-phenyl-2-tosyl-2,3,3a,4,5,7a-hexahydro-1*H*-isoindole, 23

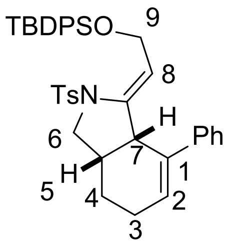

To a solution of **29** (41 mg, 65 µmol, 1.0 equiv.) in dry toluene (1 mL) was added $Pd(OAc)_2$ (1.5 mg, 6.5 µmol, 0.1 equiv.) and stirred overnight (12h). The mixture was concentrated, and the residue was purified by flash column chromatography (pentane/EtOAc 10:1) to afford **1** (22 mg, 35 µmol, 54%); **R$_f$** 0.17 (pentane/EtOAc 15:1); **IR** (thin film, ν_{max} / cm^{-1}) 2929, 2857, 2359, 1685, 1597, 1494, 1428, 1356 , 1166, 1108, 1057, 909, 821, 736, 702, 668; **^{1}H NMR** (400 MHz, $CDCl_3$) δ_H 7.57 (6H, app. dtd, *J* = 7.5, 3.8, 1.8 Hz, Ar*H*), 7.43-7.37 (2H, m, Ar*H*), 7.35-7.28 (9H, m, Ar*H*), 7.09-7.03 (2H, m, Ar*H*), 6.15 (1H, dd, *J* = 4.9, 2.8 Hz, H2), 4.97 (1H, dt, *J* = 8.5, 2.5 Hz, H8), 4.72 (1H, ddd, *J* = 14.4, 8.6, 1.3 Hz, H9), 4.43 (1H, dt, *J* = 14.4, 3.1 Hz, H9), 3.67 (1H, dd, *J* = 11.2, 7.1 Hz, H6), 3.21 (1H, d, *J* = 11.2 Hz, H6), 2.50-2.47 (1H, m, H7), 2.47 (3H, s, TsCH_3), 2.25-2.05 (2H, m, H3), 2.11-2.03 (1H, m, H5), 1.51-1.45 (1H, m, H4), 1.34 (1H, dd, *J* = 11.2, 6.2 Hz, H4), 1.00

(9H, s, C(CH_3)$_3$); **^{13}C NMR** (100 MHz, $CDCl_3$) δ_C 144.0, 140.7, 138.6, 135.7, 135.69, 135.67, 135.6, 134.4, 134.33, 134.31, 133.7, 129.7, 129.52, 129.51, 128.5, 128.1, 128.0, 127.7, 127.6, 127.2, 125.4, 123.5, 62.2, 54.7, 43.0, 33.4, 27.0, 26.0, 24.0, 21.8, 19.4; **HRMS** (ES^+) calc. for $C_{39}H_{43}NNaO_3SSi$ $[M+Na]^+$ 656.2625, found 656.2618.

2'-Amino-5,6-dihydro-[1,1'-biphenyl]-3(4H)-one, S3

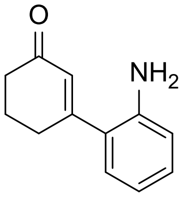

To a mixture of 2-iodoaniline (8.76 g, 40.0 mmol, 1.0 equiv.), tri-*o*-tolyl phosphine (731 mg, 2.4 mmol, 0.06 equiv.) and $Pd(OAc)_2$ (270 mg, 1.2 mmol, 0.03 equiv.) under an N_2 atmosphere was added CH_3CN (60 mL), cyclohex-2-en-1-one (4.26 mL, 44 mmol, 1.1 equiv.) and Et_3N (11.2 mL, 80 mmol, 2.0 equiv.). The reaction was heated to reflux for 60 h, then the solution was cooled to rt, diluted with water (30 mL) and extracted with EtOAc (3 x 50 mL). The organic extracts were washed with brine (100 mL), dried over Na_2SO_4, concentrated and purified by silica gel chromatography (pentane/diethyl ether 1:2) to give **S3** as a green oil (2.37 g, 12.67 mmol, 31%); **R_f** 0.27 (pentane/diethyl ether 1:3); **^{1}H NMR** (400 MHz, $CDCl_3$) δ_H 7.13 (1H, ddd, J = 8.1, 7.3, 1.6 Hz), 7.05 (1H, dd, J = 7.7, 1.6 Hz), 6.76 (1H, td, J = 7.5, 1.1 Hz), 6.71 (1H, dd, J = 8.0, 1.2 Hz), 6.24 (1H, t, J = 1.6 Hz), 3.87 (2H, s), 2.66 (2H, td, J = 6.0, 1.6 Hz), 2.49 (2H, dd, J = 7.4, 6.0 Hz), 2.14 (2H, dq, J = 7.8, 6.2 Hz); **^{13}C NMR** (100 MHz, $CDCl_3$) δ_C 199.7, 161.4, 143.0, 129.9, 128.0, 127.9, 125.7, 118.5, 116.4, 37.4, 30.4, 23.2. Data identical to literature values.[11]

Methyl (5'-methylene-2',3',4',5'-tetrahydro-[1,1'-biphenyl]-2-yl)carbamate, 31

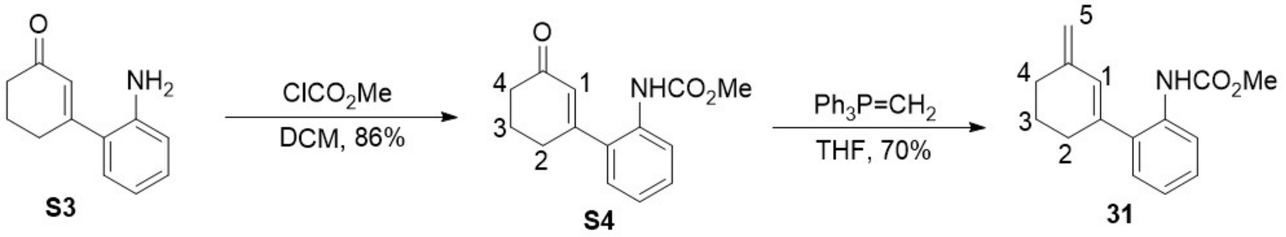

To a stirred solution of **S3** (1.14 g, 6.09 mmol, 1.0 equiv.) and pyridine (1.23 mL, 15.23 mmol, 2.5 equiv.) in CH_2Cl_2 (10 mL) at 0 °C was added dropwise methyl chloroformate (0.57 mL, 7.31 mmol, 1.2 equiv.). The mixture was stirred for 1 h, during which time the reaction was allowed to warm to

ambient temperature. The reaction was then quenched by addition of water and diluted with CH_2Cl_2. The organic layer was separated and washed with 1 N HCl, saturated $NaHCO_3$, saturated brine, dried over Na_2SO_4 and concentrated. Silica gel flash chromatography (pentane/diethyl ether 1:2) afforded **S4** (1.27 g, 5.18 mmol, 86%) as a green oil; **R_f** 0.27 (pentane/diethyl ether 1:3); **IR** (thin film, ν_{max} / cm^{-1}) 3294, 2950, 1728, 1714, 1661, 1612, 1581, 1523, 1447, 1232, 1044, 959, 852, 767, 755; **^{1}H NMR** (400 MHz, $CDCl_3$) δ_H 7.91 (1H, d, *J* = 8.2 Hz, Ar*H*), 7.45-7.31 (1H, m, Ar*H*), 7.21-7.08 (2H, m, Ar*H*), 6.61 (1H, s, N*H*), 6.09 (1H, t, *J* = 1.6 Hz, H1), 3.75 (3H, s, CO_2CH_3), 2.62 (2H, td, *J* = 6.0, 1.6 Hz, H2), 2.51 (2H, dd, *J* = 7.4, 6.0 Hz, H4), 2.20-2.14 (2H, m, H3); **^{13}C NMR** (100 MHz, $CDCl_3$) δ_C 199.1, 160.2, 154.2, 133.7, 129.7, 129.4, 127.5, 124.3, 121.8, 52.7, 37.4, 30.9, 23.2; **HRMS** (ES^+) calc. for $C_{14}H_{16}NO_3$ $[M+H]^+$ 246.1125, found 246.1126.

To a suspension of methyltriphenylphosphonium bromide (3.06 g, 8.58 mmol, 3.0 equiv.) in THF (15 mL) at 0 °C was added KO*t*Bu (963 mg, 8.58 mmol, 3.0 equiv.); a bright yellow color was observed. The mixture was stirred at 0 °C for 30 min. Then compound **S4** (700 mg, 2.86 mmol, 1.0 equiv.) was added and the reaction mixture was allowed to warm to room temperature over 1 h (the solution turned green and then dark brown). Water was added to the mixture and the product was extracted with CH_2Cl_2. The organic phase was dried with Na_2SO_4, filtered and concentrated. The residue was purified by flash chromatography on silica gel (pentane/diethyl ether 20:1) to give the title compound **31** (486 mg, 2.00 mmol, 70%) as a yellow oil; **R_f** 0.18 (pentane/Diethyl Ether 30:1); **IR** (thin film, ν_{max} / cm^{-1}) 3414, 2937, 2362, 2338, 1740, 1582, 1519, 1302, 1222, 1206, 1068, 901, 882,755; ^{1}H NMR (400 MHz, $CDCl_3$) δ_H 8.04 (1H, d, *J* = 8.1 Hz, Ar*H*), 7.27 (1H, ddd, *J* = 8.5, 6.9, 1.8 Hz, Ar*H*), 7.11 (1H, dd, *J* = 7.6, 1.8 Hz, Ar*H*), 7.05 (1H, td, *J* = 7.4, 1.2 Hz, Ar*H*), 6.80 (1H, br, N*H*), 6.21 (1H, t, *J* = 1.7 Hz, H1), 4.95-4.88 (2H, m, H5), 3.77 (3H, s, CO_2CH_3), 2.45 (2H, ddt, *J* = 7.9, 4.7, 1.5 Hz, H4), 2.37-2.31 (2H, m, H2), 1.92-1.85 (2H, m, H3); **^{13}C NMR** (100 MHz, $CDCl_3$) δ_C 154.2, 142.7, 138.5, 134.4, 133.1, 130.6, 128.3, 128.1, 123.3, 119.7, 112.6, 52.4, 30.4, 30.2, 23.4; **HRMS** (ES^+) calc. for $C_{15}H_{18}NO_2$ $[M+H]^+$ 244.1332, found 244.1334.

Methyl (5'-(hydroxymethyl)-2',3',4',5'-tetrahydro-[1,1'-biphenyl]-2-yl)carbamate, S5

Methyl ((2'-((methoxycarbonyl)amino)-3,4,5,6-tetrahydro-[1,1'-biphenyl]-3-yl)methyl)(tosyl)carbamate, 32

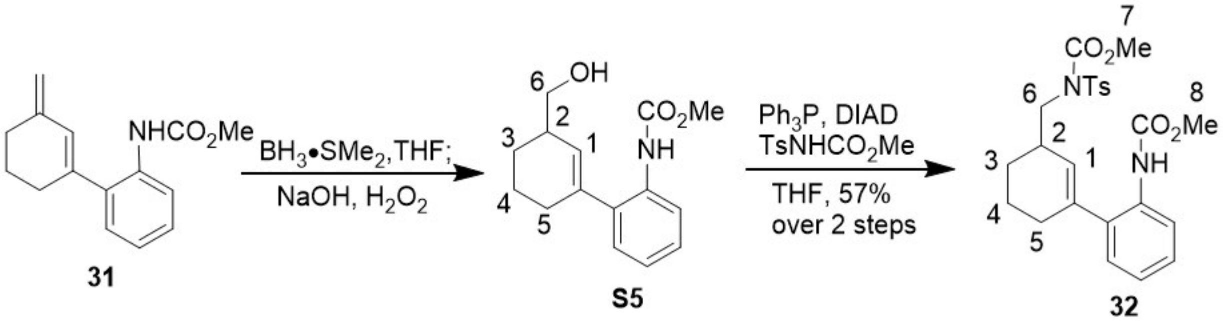

To a stirred solution of **31** (884 mg, 3.64 mmol, 1.0 equiv.) in THF (18 mL) at 0 °C was added a solution of BH_3•SMe_2 (2.19 mL, 2.0 M in THF, 4.37 mmol, 1.2 equiv.). The reaction mixture was allowed to warm to room temperature and stirred for 20 min, monitored by TLC until disappearance of the starting material. It was then cooled to 0 °C, and methanol (14 mL) was added slowly followed by addition of 3.0 M NaOH (14 mL) and 30% H_2O_2 (14 mL). The resulting mixture was stirred at room temperature for 0.5 h, then diluted with water and ether. The organic phase was separated, and the aqueous phase extracted with ether. The combined organic extracts were washed with brine, dried over Na_2SO_4 and concentrated. The residue was used directly in the next step without further purification. A sample of **S5** could be obtained through purification by flash chromatography (pentane/EtOAc 2:1) as a yellow oil; **R_f** 0.23 (pentane/EtOAc 2:1); **IR** (thin film, ν_{max} / cm^{-1}) 3413, 2930, 2863, 1736, 1583, 1521, 1448, 1302, 1224, 1068, 1044, 756; **^{1}H NMR** (400 MHz, $CDCl_3$) δ_H 8.03 (1H, s, Ar*H*), 7.24 (1H, ddd, *J* = 8.5, 7.2, 1.7 Hz, Ar*H*), 7.13 (1H, br, N*H*), 7.08 (1H, dd, *J* = 7.6, 1.8 Hz, Ar*H*), 7.02 (1H, td, *J* = 7.4, 1.2 Hz, Ar*H*), 5.65 (1H, d, *J* = 2.2 Hz, H1), 3.75 (3H, s, CO_2CH_3), 3.66 (2H, dd, *J* = 9.0, 5.5 Hz, H6), 2.49 (1H, app. dq, *J* = 5.7, 2.9 Hz, H2), 2.22-2.19 (2H, m, H5), 1.96-1.90 (1H, m, H4), 1.89-1.85 (1H, m, H3), 1.76-1.71 (1H, m, H4), 1.54-1.48 (1H, m, H3); **HRMS** (ES^+) calc. for $C_{15}H_{19}NaNO_3$ $[M+Na]^+$ 284.1257, found 284.1259.

A solution of triphenylphosphine (1.40 g, 5.33 mmol, 1.5 equiv.), DIAD (1.05 mL, 5.33 mmol, 1.5 equiv.), TsNH(CO_2Me) (976 mg, 4.26 mmol, 1.2 equiv.) in THF (12 mL) at 0 °C was stirred for 30 min, then a solution of residue **S5** (926 mg, 3.55 mmol, 1.0 equiv.) in THF (6 mL) was added. The reaction was allowed to warm to room temperature and stirred for an additional 1 h. The solvent was removed under reduced pressure. The crude product was then purified by flash column chromatography (100% CH_2Cl_2) to afford crude **32** as a yellow oil (this procedure can effectively remove DIAD adduct byproduct which can be witnessed by vanillin stain). A second purification by column chromatography (pentane/EtOAc 4:1) afforded clean product **32** (1.11 g, 2.35 mmol, 57%)

as a yellow oil; **R$_f$** 0.24 (pentane/EtOAc 4:1); **IR** (thin film, ν_{max} / cm^{-1}) 2953, 2361, 1734, 1583, 1520, 1447, 1358, 1230, 1167, 1088, 1068, 766, 673; **^{1}H NMR** (400 MHz, $CDCl_3$) δ_H 8.06-7.94 (1H, m, Ar*H*), 7.88-7.76 (2H, m, Ts*H*), 7.31-7.27 (2H, m, Ts*H*), 7.25-7.22 (1H, m, Ar*H*), 7.08-7.00 (2H, m, Ar*H*), 6.91 (1H, br, N*H*), 5.64-5.59 (1H, m, H1), 3.91-3.85 (2H, m, H6), 3.76 (3H, s, H8), 3.68 (3H, s, H7), 2.86 (1H, ddt, *J* = 7.7, 4.8, 2.7 Hz, H2), 2.42 (3H, s, TsC*H*$_3$), 2.26-2.17 (2H, m, H5), 1.97-1.93 (1H, m, H4), 1.92-1.86 (1H, m, H3), 1.72 (1H, ddt, *J* = 9.9, 7.5, 5.0 Hz, H4), 1.57-1.44 (1H, m, H3); **^{13}C NMR** (100 MHz, $CDCl_3$) δ_C 154.3, 153.3, 144.8, 138.3, 136.6, 134.5, 133.8, 129.5, 128.6, 128.5, 127.9, 123.3, 119.9, 54.0, 52.4, 51.8, 35.7, 30.4, 25.8, 21.8, 21.0; **HRMS** (ES$^+$) calc. for $C_{25}H_{28}N_2O_6S$ [M+H]$^+$ 473.1741, found 473.1743.

Methyl (5'-(((4-methylphenyl)sulfonamido)methyl)-2',3',4',5'-tetrahydro-[1,1'-biphenyl]-2-yl) carbamate, S6

Methyl (5'-(((N-(3-((*tert*-butyldiphenylsilyl)oxy)prop-1-yn-1-yl)-4-methylphenyl)sulfonamido) methyl)-2',3',4',5'-tetrahydro-[1,1'-biphenyl]-2-yl)carbamate, 33

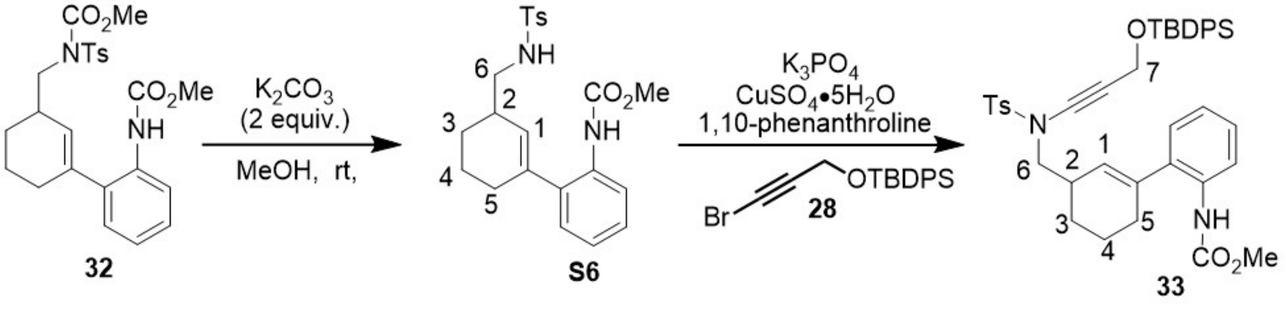

To a stirred suspension of K_2CO_3 (126 mg, 0.912 mmol, 2.0 equiv.) in methanol (3 mL) at 0 °C was added a solution of **32** (215 mg, 0.456 mmol, 1.0 equiv.) in methanol (2 mL). The reaction mixture was allowed to warm to room temperature and stirred for 3 h. The reaction was quenched by addition of saturated aqueous NH_4Cl, then the mixture was extracted with EtOAc (3 × 10 mL). The combined organic phases were dried (Na_2SO_4) and concentrated under reduced pressure to obtain the **S6** as a yellow oil (176 mg, 0.43 mmol, 93%). The residue was used directly for the next step without further purification. Alternatively, an analytical sample of **S6** could be obtained through purification by flash chromatography (pentane/EtOAc 4:1) as a yellow oil; **R$_f$** 0.15 (pentane/EtOAc 4:1); **IR** (thin film, ν_{max} / cm^{-1}) 3284, 2927, 2360, 1733, 1716, 1582, 1520, 1448, 1325 , 1230, 1157, 1068, 814, 757, 663; **^{1}H NMR** (400 MHz, $CDCl_3$) δ_H 7.93 (1H, br, Ar*H*), 7.80-7.70 (2H, m, Ts*H*),

7.30-7.26 (2H, m, Ts*H*), 7.24 (1H, ddd, *J* = 8.6, 5.8, 3.3 Hz, Ar*H*), 7.05-7.00 (2H, m, Ar*H*), 6.80 (1H, br, N*H*), 5.49 (1H, app. q, *J* = 2.1 Hz, H1), 4.86 (1H, t, *J* = 6.4 Hz, TsN*H*), 3.76 (3H, s, CO_2CH_3), 3.02-2.89 (2H, m, H6), 2.45 (1H, tt, *J* = 6.5, 3.3 Hz, H2), 2.41 (3H, s, TsCH_3), 2.23-2.10 (2H, m, H5), 1.90-1.84 (1H, m, H4), 1.84-1.79 (1H, m, H3), 1.70-1.60 (1H, m, H4), 1.43-1.34 (1H, m, H3); **^{13}C NMR** (100 MHz, $CDCl_3$) δ_C 154.3, 143.6, 138.8, 137.0, 134.2, 133.7, 129.8, 128.9, 128.3, 127.9, 127.2, 123.5, 120.0, 52.5, 48.0, 36.0, 30.0, 25.9, 21.6, 21.5; **HRMS** (ES$^+$) calc. for $C_{22}H_{26}N_2NaO_4S$ $[M+Na]^+$ 437.1505, found 437.1703.

To a stirred solution of **S6** (176 mg, 0.43 mmol, 1.0 equiv.) and bromoalkyne **28** (193 mg, 0.52 mmol, 1.2 equiv.) in dry toluene (3 mL) was added $CuSO_4.5H_2O$ (22 mg, 0.086 mmol, 0.2 equiv.), 1,10-phenanthroline (31 mg, 0.17 mmol, 0.4 equiv.) and K_3PO_4 (273 mg, 1.29 mmol, 3.0 equiv.). The mixture was shielded from light, heated at 80 °C and stirred overnight. The mixture was cooled to room temperature, and filtered through a Celite pad (washed with EtOAc). The mixture was concentrated and the residue was purified by flash column chromatography (pentane/EtOAc 10:1) to afford **33** (249 mg, 0.353 mmol, 78%) as a yellow oil; **R_f** 0.28 (pentane/EtOAc 6:1); **IR** (thin film, ν_{max} / cm^{-1}) 3415, 2932, 2858, 2242, 1738, 1583, 1519, 1448, 1363 , 1222, 1111, 1068, 704; **^{1}H NMR** (400 MHz, $CDCl_3$) δ_H 7.99 (1H, d, *J* = 7.5 Hz, Ar*H*), 7.79-7.74 (2H, m, Ts*H*), 7.69 (4H, dq, *J* = 6.8, 1.4 Hz, 2 x Ts*H*, 2 x Ar*H*), 7.46-7.39 (2H, m, Ar*H*), 7.36 (4H, tdd, *J* = 8.1, 5.1, 1.1 Hz, Ar*H*), 7.29-7.23 (3H, m, Ar*H*), 7.03 (2H, d, *J* = 0.8 Hz, Ar*H*), 6.79 (1H, br, N*H*), 5.51-5.41 (1H, m, H1), 4.48 (2H, s, H7), 3.75 (3H, s, CO_2CH_3), 3.22-3.14 (2H, m, H6), 3.20 (dd, *J* = 12.6, 8.4 Hz, 1H), 3.16 (dd, *J* = 12.6, 6.8 Hz, 1H), 2.69-2.57 (1H, m, H2), 2.41 (3H, s, TsCH_3), 2.28-2.15 (2H, m, H5), 1.89-1.84 (1H, m, H4), 1.84-1.80 (1H, m, H3), 1.71-1.64 (1H, m, H4), 1.46-1.36 (1H, m, H3), 1.04 (9H, s, $C(CH_3)_3$); **^{13}C NMR** (100 MHz, $CDCl_3$) δ_C 154.2, 144.7, 138.6, 135.7, 134.7, 134.3, 133.4, 129.90, 129.85, 128.4, 128.3, 128.0, 127.9, 127.8, 127.7, 123.3, 119.7, 78.6, 70.1, 55.8, 53.0, 52.4, 34.4, 34.3, 30.3, 26.8, 25.7, 22.5, 21.8, 21.1, 19.3, 14.2; **HRMS** (ES$^+$) calc. for $C_{41}H_{46}N_2NaO_5SSi$ $[M+Na]^+$ 729.2784, found 729.2784.

Methyl (*E*)-(5'-(((*N*-(3-((*tert*-butyldiphenylsilyl)oxy)prop-1-yn-1-yl)-4-methylphenyl) sulfonamido)methyl)-2',3',4',5'-tetrahydro-[1,1'-biphenyl]-2-yl)(1,2-dichlorovinyl)carbamate, 34

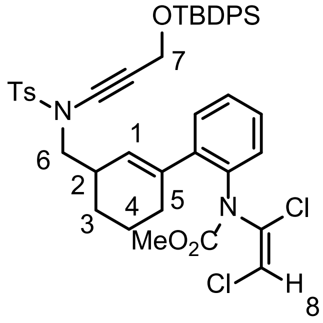

To a solution of **33** (111 mg, 0.157 mmol, 1.0 equiv.), powdered Cs_2CO_3 (769 mg, 2.36 mmol, 15 equiv.) in DMF (3 mL) was added dropwise trichloroethylene (0.042 mL, 0.471 mmol, 3 equiv.), and the resulting mixture stirred at 50 ºC for 1.5 h. The mixture was then cooled to room temperature and ethyl acetate and water (2:1, 15 mL) were added. The layers were separated and the aqueous layer was extracted with EtOAc (3 × 10 mL). The combined organic layers were dried over Na_2SO_4 and concentrated. Silica gel flash chromatography (pentane/EtOAc 15:1) afforded the title compound **34** (84 mg, 0.105 mmol, 67%) as a yellow oil; $\mathbf{R_f}$ 0.27 (pentane/EtOAc 10:1); **IR** (thin film, ν_{max} / cm^{-1}) 3072, 2931, 2858, 2242, 1764, 1440, 1362, 1311, 1169, 1111, 1064, 909, 824, 704, 660; ^{1}H NMR (400 MHz, $CDCl_3$) δ_H 7.76 (2H, d, *J* = 8.0 Hz, Ts*H*), 7.68 (4H, dt, *J* = 8.1, 1.5 Hz, 2 x Ts*H*, 2 x Ar*H*), 7.44-7.40 (2H, m, Ar*H*), 7.36 (4H, ddd, *J* = 8.2, 6.5, 4.3 Hz, Ar*H*), 7.32-7.27 (3H, m, Ar*H*), 7.26-7.17 (3H, m, Ar*H*), 6.27 (1H, s, H8), 5.59 (1H, s, H1), 4.46 (2H, s, H7), 3.78 (3H, s, CO_2CH_3), 3.23-3.08 (2H, m, H6), 2.58 (1H, br, H2), 2.41 (3H, s, TsCH_3), 2.29 (2H, br, H5), 1.85-1.78 (1H, m, H4), 1.77-1.72 (1H, m, H3), 1.68-1.58 (1H, m, H4), 1.42-1.32 (1H, m, H3), 1.04 (9H, s, $C(CH_3)_3$); ^{13}**C NMR** (100 MHz, $CDCl_3$) δ_C 144.6, 141.9, 138.2, 135.7, 135.0, 134.7, 133.37, 133.36, 131.7, 129.9, 129.8, 128.3, 127.84, 127.77, 127.7, 114.6, 78.5, 70.0, 55.5, 54.1, 53.1, 34.4, 29.4, 26.8, 25.3, 21.8, 21.0, 19.3; **HRMS** (ES^+) calc. for $C_{43}H_{46}Cl_2N_2NaO_5SSi$ $[M+H]^+$ 823.2166, found 823.2161.

Methyl (*E*)-(1,2-dichlorovinyl)(5'-(((4-methylphenyl)sulfonamido)methyl)-2',3',4',5'-tetrahydro-[1,1'-biphenyl]-2-yl)carbamate, S7

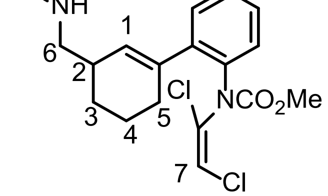

To an oven dried, nitrogen flushed flask equipped with a magnetic stirrer bar and a septum, was

added **34** (128 mg, 0.16 mmol, 1.0 equiv.) and anhydrous TBME (1.6 mL), and the solution was cooled to 0 °C whilst stirring. To this solution was added the LiHMDS (1.0 M in toluene, 0.192 mL, 0.192 mmol, 1.2 equiv.) dropwise, and the reaction stirred at 0 °C for 1 hour, analyzed by TLC. After total conversion, the cooling bath was then removed and the reaction mixture was then allowed to warm to room temperature whilst stirring. Then the mixture was added CuCN·2LiCl (1.0 M solution in THF, 8 µL, 8 µmol, 5 mol%), followed by Me_2Zn solution (1.2 M in toluene, 0.40 mL, 0.48 mmol, 3 equiv.) dropwise, and the reaction was stirred for 30 minutes at room temperature. Reaction completion was then verified by TLC. The reaction was quenched with aqueous saturated NH_4Cl (1 mL), the mixture was dilute with water, the layers were separated and the aqueous layer extracted with EtOAc (3 × 10 mL). The combined organic layers were dried over Na_2SO_4 and concentrated in vacuo. Silica gel flash chromatography (Pentane/EtOAc 10:1) afforded the tittle product **S7** (24 mg, 47 µmol, 30%) as yellow oil; **R$_f$** 0.14 (Pentane/EtOAc 10:1); **IR** (thin film, νmax / cm-1) 3278, 2929, 2859, 2360, 2340, 1734, 1440, 1316, 1159, 1094, 1039, 910, 827, 733, 650; **^{1}H NMR** (500 MHz, $CDCl_3$) δ 7.74 (2H, d, *J* = 7.9 Hz, Ts*H*), 7.31 – 7.26 (4H, m, 2 x Ts*H*, 2 x Ar*H*), 7.25 – 7.16 (2H, m, Ar*H*), 6.32 (1H, s, H7), 5.52 (1H, br, H1), 4.82 (1H, br, N*H*), 3.82 (3H, s, CO_2CH_3), 3.09 – 2.80 (2H, m, H6), 2.42 (1H, br, H2), 2.41 (3H, s, $TsCH_3$), 2.34 – 2.15 (2H, m, H5), 1.87 – 1.80 (1H, m, H4), 1.78 – 1.73 (1H, m, H3), 1.62 (1H, s, H4), 1.50 – 1.38 (1H, m, H3); **^{13}C NMR** (125 MHz, $CDCl_3$) δ_C 153.4, 143.3, 137.2, 134.6, 131.6, 129.8, 129.7, 128.3, 127.8, 127.2, 115.1, 54.4, 48.0, 35.8, 29.0, 25.5, 21.6; **HRMS** (ES+) calc. for $C_{24}H_{26}Cl_2N_2NaO_4S$ $[M+Na]^+$ 531.0883, found 531.0883.

Methyl (*E*)-(1,2-dichlorovinyl)(5'-(((*N*-(methoxy carbonyl)-4-methylphenyl)sulfonamido) methyl)-2',3',4',5'-tetrahydro-[1,1'-biphenyl]-2-yl)carbamate, S8

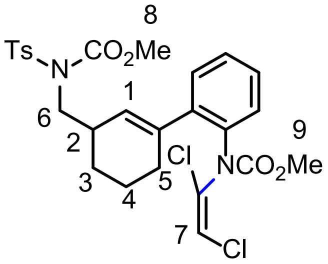

To a solution of **32** (118 mg, 0.25 mmol, 1.0 equiv.) and powdered Cs_2CO_3 (1.22 g, 3.75 mmol, 15 equiv.) in DMF (2.5 mL) was added dropwise trichloroethylene (0.07 mL, 0.75 mmol, 3.0 equiv.),

and the resulting mixture stirred at 50 ºC for 30 min. The mixture was then cooled to room temperature and ethyl acetate and water (2:1, 15 mL) were added. The layers were separated and the aqueous layer extracted with EtOAc (3 × 10 mL). The combined organic layers were dried over Na_2SO_4 and concentrated. Silica gel flash chromatography (pentane/EtOAc 4:1) afforded the title compound **S8** (134 mg, 0.237 mmol, 94%) as a yellow oil; **R_f** 0.36 (pentane/EtOAc 4:1); **IR** (thin film, ν_{max} / cm^{-1}) 3091, 2954, 2931, 2260, 2338, 1734, 1490, 1358, 1311, 1166, 1111, 1088, 909, 827, 761, 732; **^{1}H NMR** (400 MHz, $CDCl_3$) δ_H 7.84-7.78 (2H, m, Ts*H*), 7.33-7.27 (4H, m, 2 x Ts*H*, 2 x Ar*H*), 7.23 (2H, ddd, *J* = 7.7, 3.8, 1.7 Hz, Ar*H*), 6.28 (1H, s, H7), 5.71 (1H, s, H1), 3.88-3.76 (5H, m, 2 x H6, 3 x H9), 3.66 (3H, s, H8), 2.77 (1H, br, H2), 2.43 (3H, s, TsCH_3), 2.31 (2H, br, H5), 1.94-1.88 (1H, m, H4), 1.84 (1H, qd, *J* = 7.6, 3.3 Hz, H3), 1.72-1.63 (1H, m, H4), 1.50-1.42 (1H, m, H3); **^{13}C NMR** (100 MHz, $CDCl_3$) δ_C 153.3, 144.6, 142.0, 137.7, 136.7, 135.0, 131.7, 129.8, 129.5, 128.6, 128.2, 127.7, 114.6, 54.1, 53.9, 51.5, 36.1, 29.4, 25.4, 21.8, 21.2; **HRMS** (ES^+) calc. for $C_{26}H_{29}Cl_2N_2O_6S$ $[M+H]^+$ 567.1118, found 567.1117.

Methyl (5'-(((4-methylphenyl)sulfonamido)methyl)-2',3',4',5'-tetrahydro-[1,1'-biphenyl]-2-yl (prop-1-yn-1-yl)carbamate, 35

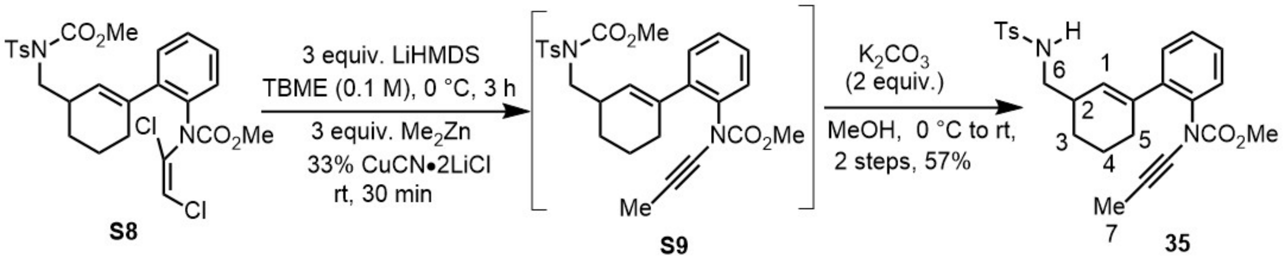

To a stirred solution of **S8** (215 mg, 0.38 mmol, 1.0 equiv.) in anhydrous TBME (3.8 mL) at 0 °C was added LiHMDS (1.0 M in toluene, 1.1 mL, 1.14 mmol, 3.0 equiv.) dropwise, and the reaction stirred at 0 °C for 3 hours. After total conversion of the dichloroenamide to the intermediate chloroynamide, the mixture was allowed to warm to room temperature. CuCN·2LiCl (1.0 M solution in THF, 125 µL, 125 µmol, 33 mol%) was added, followed by Me_2Zn (1.2 M in toluene, 0.95 mL, 1.14 mmol, 3.0 equiv.) dropwise, and the reaction was stirred for 30 minutes at room temperature until complete as judged by TLC. The reaction was quenched by addition of aqueous saturated NH_4Cl (1 mL), then the mixture was diluted with water, the layers were separated and the

aqueous layer extracted with EtOAc (3 × 10 mL). The combined organic phases were dried over Na_2SO_4 and concentrated. The crude product was taken forward to the next step without further purification.

To a stirred mixture of K_2CO_3 (63 mg, 0.454 mmol, 2.0 equiv.) in methanol (0.2 mL) at 0 °C was added a solution of **S9** (116 mg, 0.227 mmol, 1.0 equiv.) in methanol (2 mL). The reaction mixture was warmed to room temperature and stirred for 3 h. The reaction was quenched by addition of saturated aqueous NH_4Cl, and the mixture was extracted with EtOAc (3 × 10 mL). The combined organic phases were dried over Na_2SO_4 and concentrated. Silica gel flash chromatography (pentane/EtOAc 4:1) afforded the title compound **35** (99 mg, 0.22 mmol, 57%) as a yellow oil; $\mathbf{R_f}$ 0.14 (pentane/EtOAc 4:1); **IR** (thin film, νmax / cm-1) 3278, 2926, 2861, 2362, 2339, 1732, 1616, 1442, 1326, 1160, 1063, 1039, 912, 815, 735; **^{1}H NMR** (400 MHz, $CDCl_3$) δ_H 7.77 (2H, d, J = 7.9 Hz, Ts*H*), 7.33-7.26 (5H, m, 2 x Ts*H*, 3 x Ar*H*), 7.22-7.16 (1H, m, Ar*H*), 5.48 (1H, br, H1), 4.95 (1H, br, N*H*), 3.76 (3H, s, CO_2CH_3), 2.94 (2H, s, H6), 2.49-2.43 (1H, m, H2), 2.41 (3H, s, TsCH_3), 2.24 (2H, s, H5), 1.90 (3H, s, H7), 1.82-1.79 (1H, m, H4), 1.78-1.73 (1H, m, H3), 1.66-1.58 (1H, m, H4), 1.41 (1H, br, H3); **^{13}C NMR** (100 MHz, $CDCl_3$) δ_C 155.9, 143.2, 141.3, 137.4, 136.9, 129.7, 128.9, 128.5, 127.9, 127.7, 127.2, 74.2, 65.3, 54.4, 48.2, 35.6, 28.9, 25.6, 21.6, 21.4, 3.4; **HRMS** (ES+) calc. for $C_{25}H_{28}N_2NaO_4S$ $[M+Na]^+$ 475.1662, found 475.1660.

Methyl (5'-(((*N*-(3-((*tert*-butyldiphenylsilyl)oxy)prop-1-yn-1-yl)-4-methylphenyl) sulfonamido)methyl)-2',3',4',5'-tetrahydro-[1,1'-biphenyl]-2-yl)(prop-1-yn-1-yl)carbamate, 30

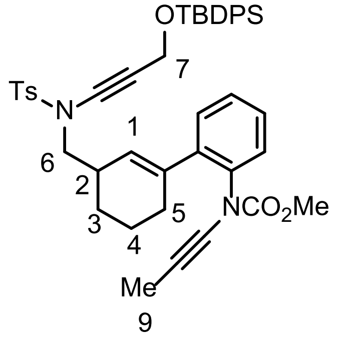

To a stirred solution of **35** (99 mg, 0.22 mmol, 1.0 equiv.) and bromoalkyne **28** (78 mg, 0.26 mmol, 1.2 equiv.) in dry toluene (2 mL) was added $CuSO_4{\cdot}5H_2O$ (11 mg, 0.044 mmol, 0.2 equiv.), 1,10-phenanthroline (16 mg, 0.088 mmol, 0.4 equiv.) and K_3PO_4 (140 mg, 0.66 mmol, 3.0 equiv.). The mixture was shielded from light and heated to 80 °C and stirred overnight. After cooling to

room temperature, the reaction was filtered through a Celite pad (washed with EtOAc). The solvent was evaporated and the residue was purified by flash column chromatography (pentane/EtOAc 8:1) to afford **30** (105 mg, 0.141 mmol, 68%) as a yellow oil; **R_f** 0.26 (pentane/EtOAc 6:1); **IR** (thin film, ν_{max} / cm^{-1}) 3657, 2981, 2884, 2361, 2340, 1734, 1382, 1252, 1153 , 1073, 954, 818; **^{1}H NMR** (500 MHz, $CDCl_3$) δ_H 7.78 (2H, d, *J* = 7.9 Hz, Ts*H*), 7.73-7.65 (4H, m, 2 x Ts*H*, 2 x Ar*H*), 7.46-7.34 (7H, m, Ar*H*), 7.32-7.27 (4H, m, Ar*H*), 7.20 (1H, dd, *J* = 6.9, 2.4 Hz, Ar*H*), 5.48 (1H, s, H1), 4.48 (2H, s, H7), 3.75 (3H, br, CO_2CH_3), 3.25-3.18 (1H, m, H6), 3.12-3.08 (1H, m, H6), 2.60 (1H, br, H2), 2.42 (3H, s, TsCH_3), 2.28 (2H, br s, H5), 1.90 (3H, s, H9), 1.85-1.81 (1H, m, H4), 1.81-1.77 (1H, m, H3), 1.68-1.61 (1H, m, H4), 1.43-1.37 (1H, m, H3), 1.05 (9H, s, $C(CH_3)_3$); **^{13}C NMR** (125 MHz, $CDCl_3$) δ_C 155.7, 144.5, 141.4, 138.2, 137.0, 135.7, 135.69, 134.67, 133.34, 133.32, 129.9, 129.8, 129.3, 128.4, 127.9, 127.8, 127.7, 78.5, 73.8, 70.0, 64.6, 55.5, 54.2, 53.0, 34.3, 29.2, 26.8, 25.4, 21.7, 20.9, 19.3, 3.4; **HRMS** (ES^+) calc. for $C_{44}H_{48}N_2NaO_5SSi$ $[M+Na]^+$ 767.2945, found 767.2941.

Methyl (2*Z*,3*S*,3'*Z*,3a'*S*,7a'*S*)-3'-(2-((*tert*-butyldiphenylsilyl)oxy)ethylidene)-2-ethylidene-2'-tosyl-1',2',3',3a',7',7a'-hexahydrospiro[indoline-3,4'-isoindole]-1-carboxylate, 36

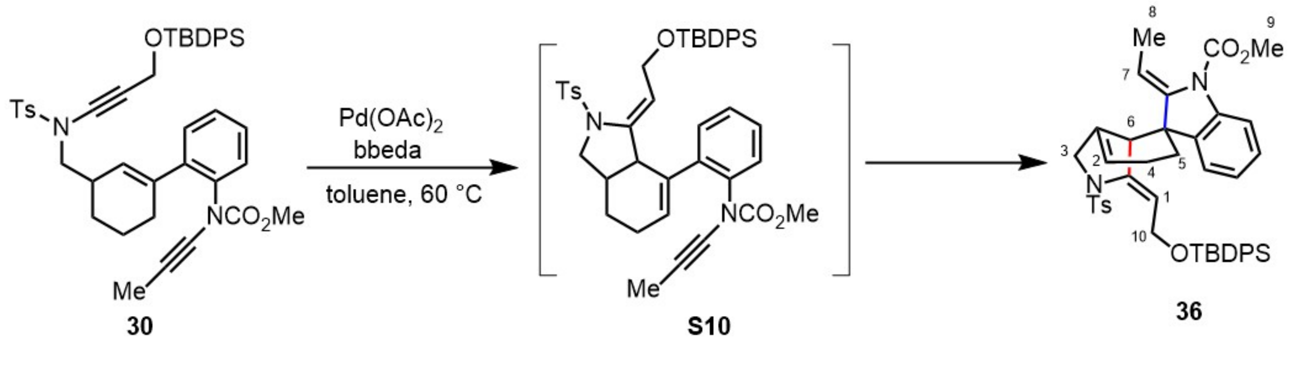

To a solution of bis-ynamide **30** (26 mg, 34.9 µmol, 1.0 equiv.) in dry toluene (1 mL) was added $Pd(OAc)_2$ (1.56 mg, 6.98 µmol, 0.2 equiv.) and bbeda (1.65 mg, 6.98 µmol, 0.2 equiv.). The stirred mixture was heated to 60 °C for 4 h until the starting material was consumed (as judged by TLC). Then the mixture was cooled to room temperature and concentrated. The residue was purified by column chromatography (pentane/EtOAc 8:1) to afford **36** (7.8 mg, 10.5 µmol, 30%) as yellow oil; **R_f** 0.21 (pentane/EtOAc 10:1); **IR** (thin film, ν_{max} / cm^{-1}) 3070, 2951, 2851, 1775, 1714, 1598, 1524, 1476, 1461, 1356, 1167, 1105, 1043, 821, 741, 704, 664; **^{1}H NMR** (500 MHz, $CDCl_3$) δ_H 7.65 (2H, d, *J* = 5.3 Hz, Ts*H*), 7.63-7.61 (4H, m, 2 x Ts*H*, 2 x Ar*H*), 7.42-7.40 (2H, m, 2 x Ar*H*), 7.36 (5H, dd, *J* = 8.0, 6.6 Hz, 2 x Ts*H*, 1 x Ar*H*), 7.23-7.19 (4H, m, Ar*H*), 7.04-7.03 (1H, m, Ar*H*), 5.85 (1H, t, *J* =

6.7 Hz, H1), 5.33 (1H, s, H2), 5.07 (1H, q, J = 7.0 Hz, H7), 4.27-4.25 (2H, m, H10), 3.90-3.88 (1H, m, H6), 3.87 (3H, s, H9), 3.81 (2H, br s, H3), 2.36 (3H, s, TsCH_3), 2.13 (2H, t, J = 6.2 Hz, H4), 1.76-1.74 (1H, m, H5), 1.51-1.48 (1H, m, H5), 1.62 (3H, d, J = 7.0 Hz, H8), 1.00 (9H, s, $C(CH_3)_3$); **^{13}C NMR** (125 MHz, $CDCl_3$) δ_C 153.4, 145.2, 144.1, 142.2, 138.9, 136.9, 135.9, 135.71, 135.0, 133.5, 133.4, 130.5, 129.9, 129.86, 129.81, 128.0, 127.9, 127.7, 124.3, 123.4, 122.6, 116.6, 112.3, 108.5, 60.1, 54.9, 53.1, 52.8, 50.2, 35.8, 27.0, 26.1, 21.7, 19.3, 14.5; **HRMS** (ES^+) calc. for $C_{44}H_{49}N_2O_5SSi$ $[M+H]^+$ 745.3106, found 745.3121.

Methyl (3*S*,3a'*S*,*Z*)-3'-(2-((*tert*-butyldiphenylsilyl)oxy)ethylidene)-2-oxo-2'-tosyl-1',2',3',3a',5',6'-h exahydrospiro[indoline-3,4'-isoindole]-1-carboxylate, 37

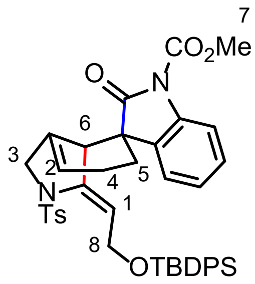

To a stirred solution of double cyclization compound **36** (6 mg, 8.06 μmol, 1.0 equiv.) in DCM (1 mL) was added *m*-CPBA (4.2 mg, 24.2 μmol, 3.0 equiv.). The mixture was stirred 2 h, then the reaction was quenched by saturated $NaHCO_3$ solution. Extracted with DCM twice, the solvent was evaporated to dryness and the residue was purified by flash column chromatography (Pentane/EtOAc 4:1) to afford **37** (4 mg, 5.46 μmol, 68%) as a yellow oil; **R_f** 0.23 (Pentane/EtOAc 4:1); **IR** (thin film, ν_{max} / cm^{-1}) 3657, 2981, 2884, 2361, 2340, 1734, 1382, 1252, 1153 , 1073, 954, 818; **^{1}H NMR** (500 MHz, $CDCl_3$) δ_H 7.66 – 7.59 (6H, m, Ar*H*), 7.42 (2H, dd, J = 7.4, 2.2 Hz, Ar*H*), 7.40 – 7.35 (5H, m, Ar*H*), 7.23 – 7.18 (4H, m, Ar*H*), 7.12 (1H, td, J = 7.6, 1.1 Hz, Ar*H*), 5.78 (1H, dd, J = 7.9, 5.6 Hz, H1), 5.18 (1H, s, H2), 4.33 – 4.26 (1H, m, H8), 4.20 (1H, dd, J = 13.4, 5.6 Hz, H8), 4.00 (3H, s, H7), 3.97 (1H, dd, J = 14.2, 6.3 Hz, H3), 3.80 – 3.74 (1H, m, H6), 3.54 (1H, d, J = 13.5 Hz, H3), 2.34 (3H, s, TsCH_3), 2.15 – 2.07 (2H, m, H4), 1.97-1.95 (1H, m, H5), 1.93-1.91 (1H, m, H5); **^{13}C NMR** (125 MHz, $CDCl_3$) δ_C 167.4, 151.8, 144.2, 138.3, 137.8, 135.7, 135.7, 133.5, 133.3, 129.9, 129.7, 128.6, 128.0, 127.9, 127.89, 125.2, 125.1, 124.3, 122.6, 115.0, 60.1, 54.9, 54.0, 50.2, 33.4, 27.0, 25.5, 21.7, 19.3, 18.2; **HRMS** (ES^+) calc. for $C_{42}H_{45}N_2O_6SSi$ $[M+H]^+$ 733.2762, found 733.2747.

Methyl (2-bromophenyl)carbamate, S11

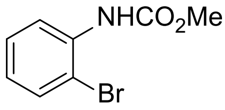

To a solution of 2-bromoaniline (2.00 g, 11.7 mmol, 1.0 equiv.) and pyridine (2.36 mL, 29.25 mmol, 2.5 equiv.) in DCM (10 mL), cooled in ice water bath under Ar, was added dropwise a solution of methyl chloroformate (1.09 mL, 14.04 mmol, 1.2 equiv.). The mixture was stirred overnight, during which time the ice bath warmed up to ambient temperature. The reaction was quenched with water and diluted with DCM. The organic layer was separated and washed with 1N HCl, saturated $NaHCO_3$, saturated brine, dried over Na_2SO_4 and concentrated under reduced pressure to give the title compound **S11** as white oil (2.67 g, 11.66 mol, 99.7%); **R$_f$** 0.3 (pentane/EtOAc 30:1); ^{1}H NMR (400 MHz, $CDCl_3$) δ_H 8.14 (d, J = 8.3 Hz, 1H), 7.54-7.46 (m, 1H), 7.30 (tt, J = 8.3, 1.9 Hz, 1H), 7.14 (s, 1H), 6.97-6.87 (m, 1H), 3.83-3.77 (m, 3H); ^{13}C NMR (100 MHz, $CDCl_3$) δ_C 153.8, 136.0, 132.4, 128.5, 124.4, 120.4, 112.7, 52.7. Data identical to literature values.[12]

Spiro[benzo[*d*][1,3]oxazine-4,1'-cyclohexan]-2(1*H*)-one, S12

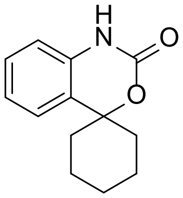

To a dry, argon flushed flask was added **S12** (126 mg, 0.55 mmol, 1.0 equiv.) and anhydrous THF (3 mL) and cooled down to -78 °C whist stirring. Then *n*-BuLi (2.5 M in hexane, 0.55 mL, 1.375 mmol, 2.5 equiv.) was added dropwise and stirred for 15 min. After total conversion to organolithium compound (confirmed by TLC), cyclohexanone (162 mg, 1.65 mmol, 3.0 equiv.) was added, then the mixture was allowed to warm up to 0 °C and stirred for 1 h. Quenching with saturated NH_4Cl solution (2 mL), the mixture was dilute with water, the layers were separated and the aqueous layer extracted with EtOAc (3 × 10 mL). The combined organic layers were dried over Na_2SO_4 and concentrated in vacuo. Silica gel flash chromatography (pentane/EtOAc 5:1) afforded the title compound **S12** (96 mg, 0.442 mmol, 80%); **R$_f$** 0.25 (pentane/EtOAc 5:1); **IR** (thin film, ν_{max} / cm^{-1}) 3243, 3099, 2933, 2861, 1760, 1598, 1502, 1375, 1342, 1263, 1247, 1047, 1021, 935, 752, 667; ^{1}H NMR (400 MHz, $CDCl_3$) δ_H 9.29 (1H, s, N*H*), 7.21 (1H, td, J = 7.7, 1.4 Hz, Ar*H*),

7.14 (1H, d, *J* = 7.2 Hz, Ar*H*), 7.04 (1H, td, *J* = 7.6, 1.1 Hz, Ar*H*), 6.92-6.87 (1H, m, Ar*H*), 2.19 (2H, d, *J* = 13.3 Hz, Cy*H*), 1.96 (2H, qt, *J* = 13.1, 3.3 Hz, Cy*H*), 1.82 (1H, d, *J* = 13.2 Hz, Cy*H*), 1.76 (2H, dd, *J* = 13.5, 4.1 Hz, Cy*H*), 1.68 (2H, td, *J* = 13.1, 3.7 Hz, Cy*H*), 1.31 (1H, qt, *J* = 13.1, 3.7 Hz, Cy*H*); ^{13}C NMR (100 MHz, $CDCl_3$) δ_C 153.2, 134.4, 128.8, 126.7, 123.5, 123.3, 114.8, 83.8, 35.9, 25.1, 21.0; **HRMS** (ES^+) calc. for $C_{13}H_{16}NO_2$ $[M+H]^+$ 218.1176, found 218.1175.

4. Reference

1. A. Mekareeya, P. R. Walker, A. Couce-Rios, C. D. Campbell, A. Steven, R. S. Paton, E. A. Anderson, *J. Am. Chem. Soc.*, 2017, **139**, 10104−10114.
2. D. Strübing, A. Wangelin, H. Neumann, D. Gördes, S. Hübner, S. Klaus, A. Spannenberg, M. Beller, *Eur. J. Org. Chem.*, 2005, **2005**, 107−113.
3. Z. Lu, X. Zhang, Z. Guo, Y. Chen, T. Mu, A. Li, *J. Am. Chem. Soc.*, 2018, **140**, 9211–9218.
4. X. Lu, J. Li, J. Wang, S. Wang, Y. Li, Y. Zhu, R. Zhou, W. Ma, *RSC Adv.*, 2018, **8**, 41561–41565.
5. N. Zheng, Y. Chang, L. Zhang, J. Gong, Z. Yang, *Chem. Asian J.*, 2016, **11**, 371–375.
6. T. W. Liwosz, S. R. Chemler, *Chem. Eur. J.*, 2013, **19**, 12771–1277.
7. C. Kong, T. G. Driver, *Org. Lett.*, 2015, **17**, 802–805.
8. F. Gelat, J. Lohier, A. Gaumont, S. Perrio, *Adv. Synth. Catal.*, 2015, **357**, 2011–2016.
9. S. Ortgies, A. Breder, *Org. Lett.*, 2015, **17**, 2748–2751.
10. S. J. Mansfield, R. C. Smith, J. R. J. Yong, J. Yong, O. L. Garry, E. A. Anderson, *Org. Lett.*, 2019, **21**, 2918–2922.
11. S. E. Reisman, J. M. Ready, M. M. Weiss, A. Hasuoka, M. Hirata, K. Tamaki, T. V. Ovaska, C. J. Smith, J. L. Wood, *J. Am. Chem. Soc.*, 2008, **130**, 2087–2100.
12. P. Mampuys, H. Neumann, S. Sergeyev, R. V. A. Orru, H. Jiao, A Spannenberg, B. U. W. Maes, M. Beller, *ACS Catal.*, 2017, **7**, 5549–5556.

5. NMR spectra of compounds

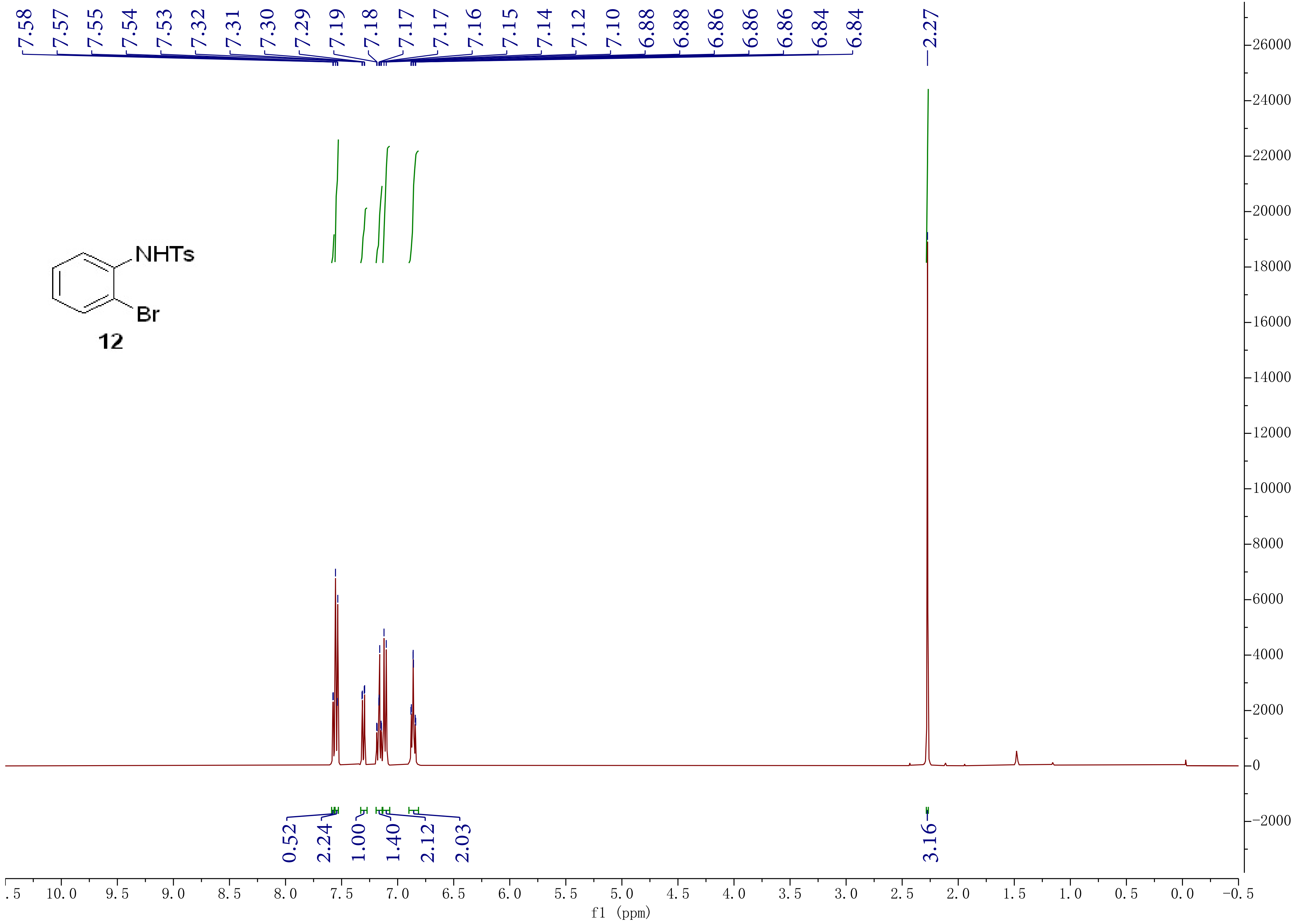

7.58
7.57
7.55
7.54
7.53
7.32
7.31
7.30
7.29
7.19
7.18
7.17
7.17
7.16
7.15
7.14
7.12
7.10
6.88
6.88
6.86
6.86
6.86
6.84
6.84
2.27
NHTs
Br
12
0.52
2.24
1.00
1.40
2.12
2.03
3.16
f1 (ppm)

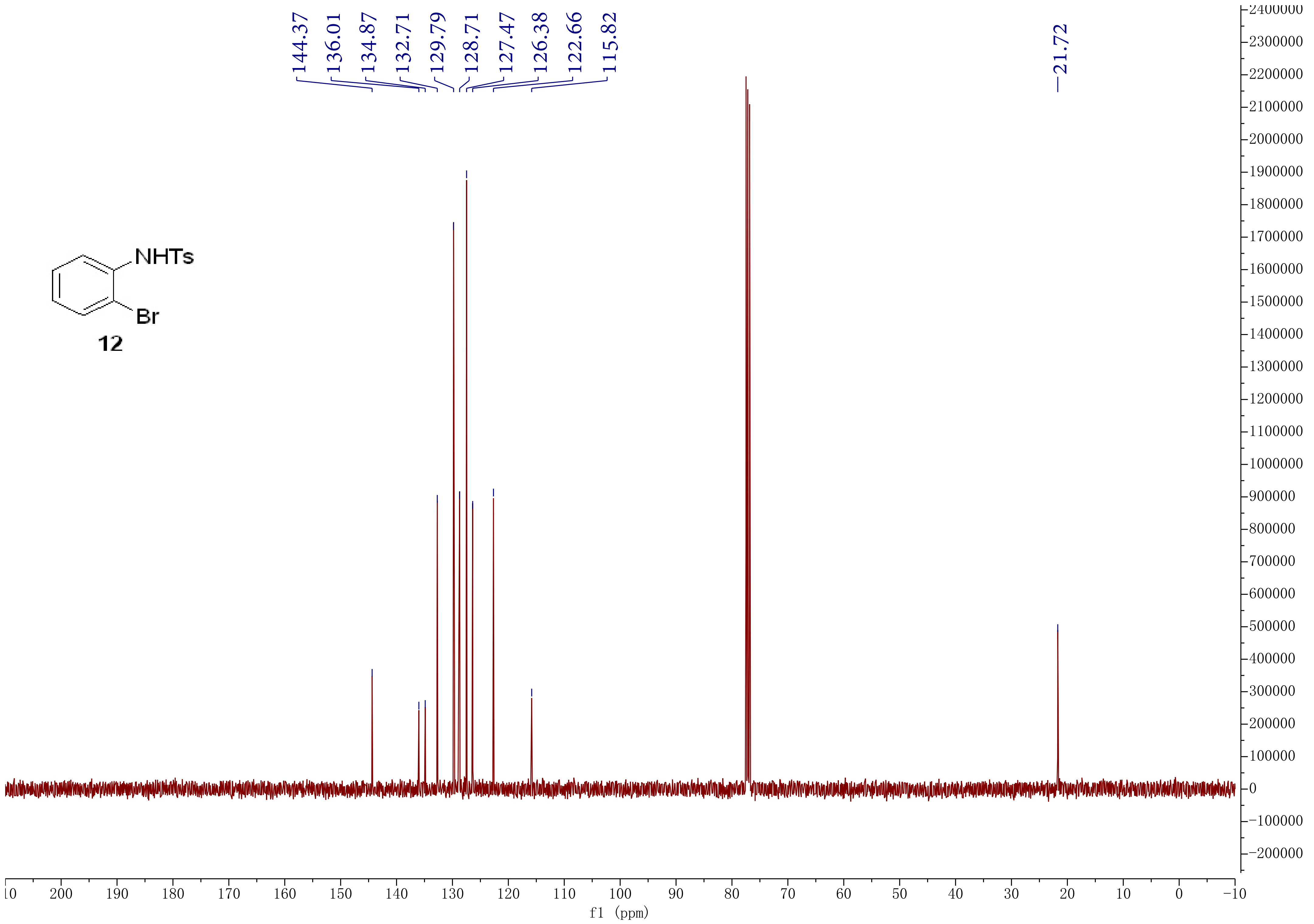

NHTs
Br
144.37
136.01
134.87
132.71
129.79
128.71
127.47
126.38
122.66
115.82
21.72
f1 (ppm)

12

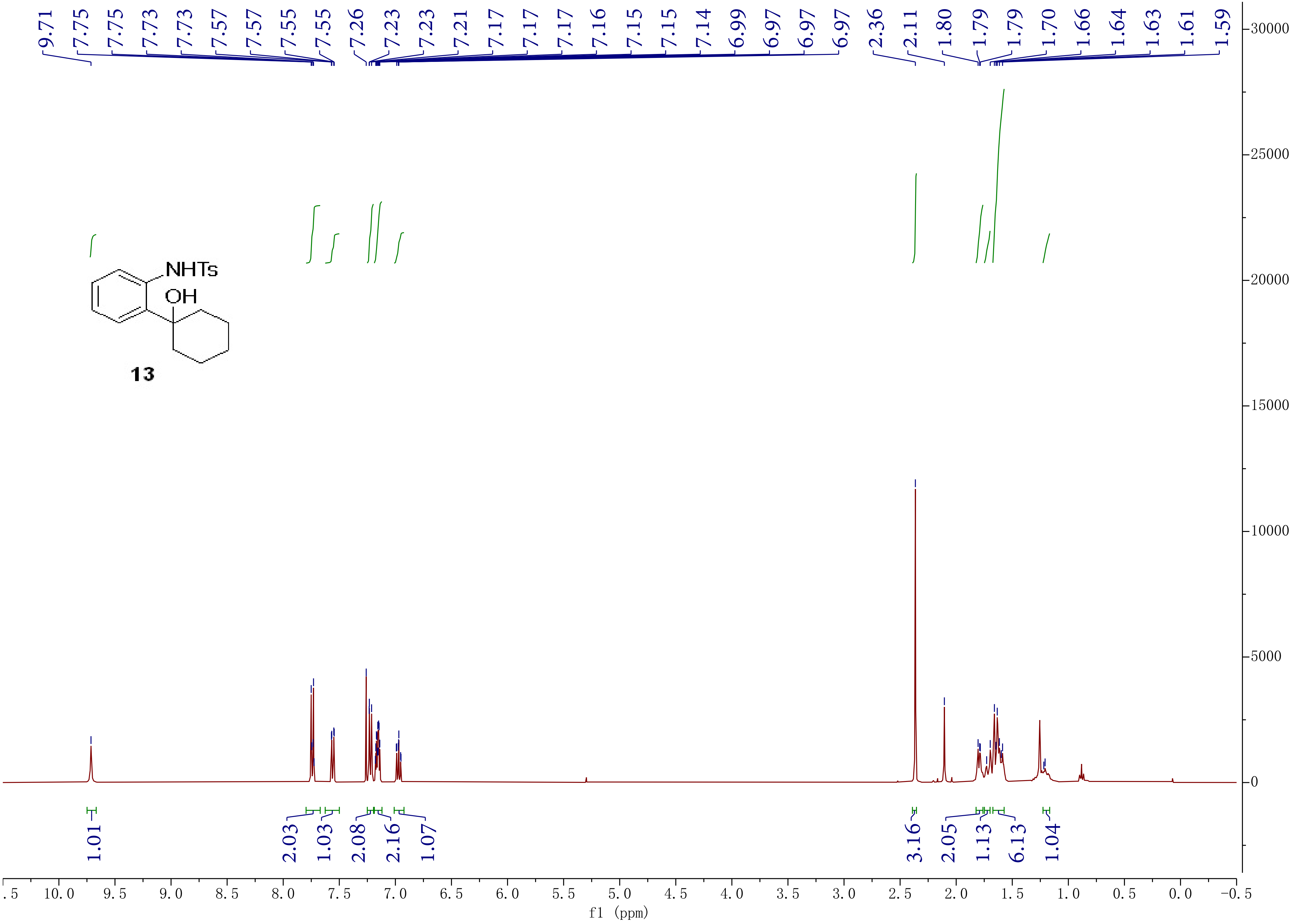

9.71
7.75
7.75
7.73
7.73
7.57
7.57
7.55
7.55
7.26
7.23
7.23
7.21
7.17
7.17
7.17
7.16
7.15
7.15
7.14
6.99
6.97
6.97
6.97
2.36
2.11
1.80
1.79
1.79
1.70
1.66
1.64
1.63
1.61
1.59
1.01
2.03
1.03
2.08
2.16
1.07
3.16
2.05
1.13
6.13
1.04
f1 (ppm)
NHTs
OH

13

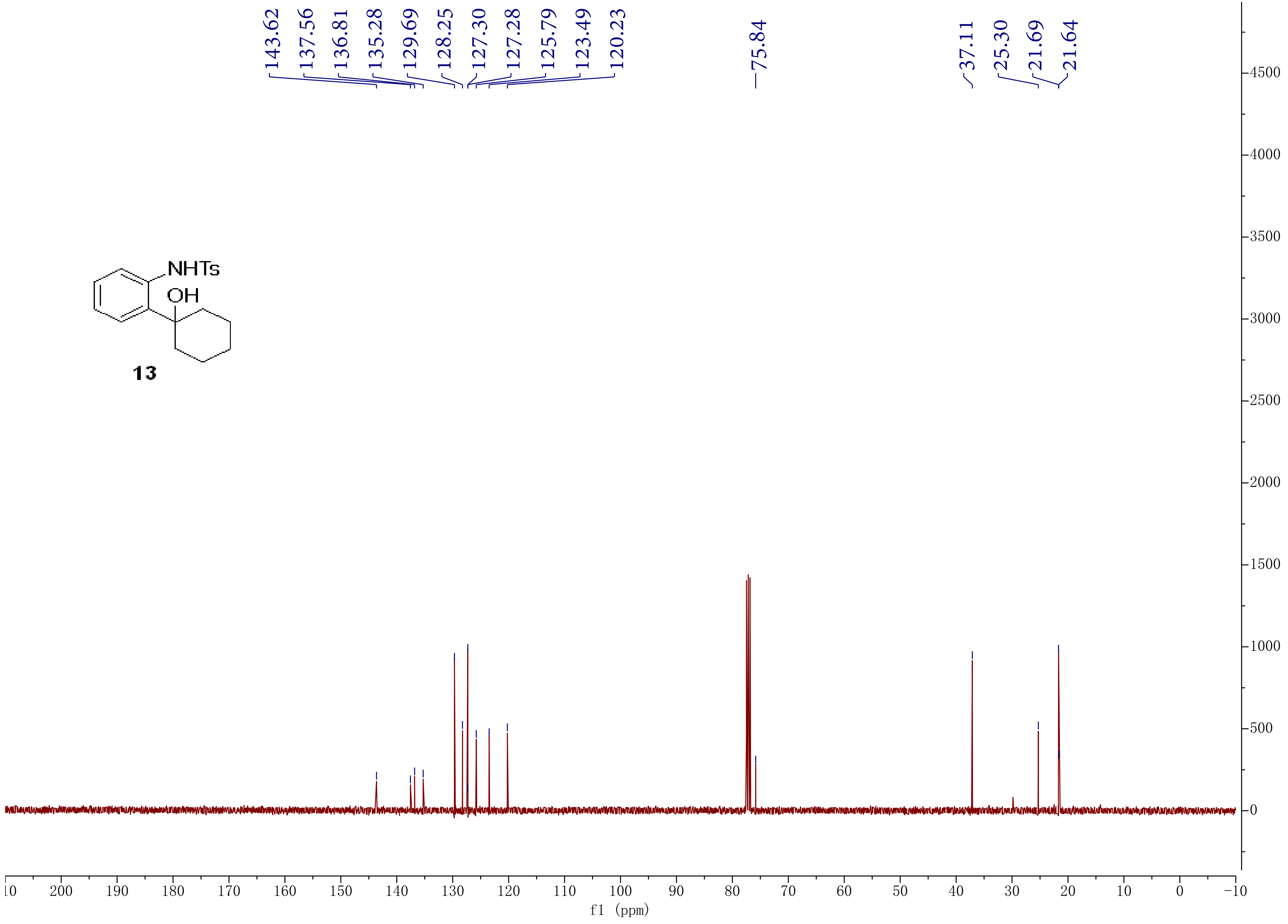
143.62
137.56
136.81
135.28
129.69
128.25
127.30
127.28
125.79
123.49
120.23
75.84
37.11
25.30
21.69
21.64
NHTs
OH
13
f1 (ppm)

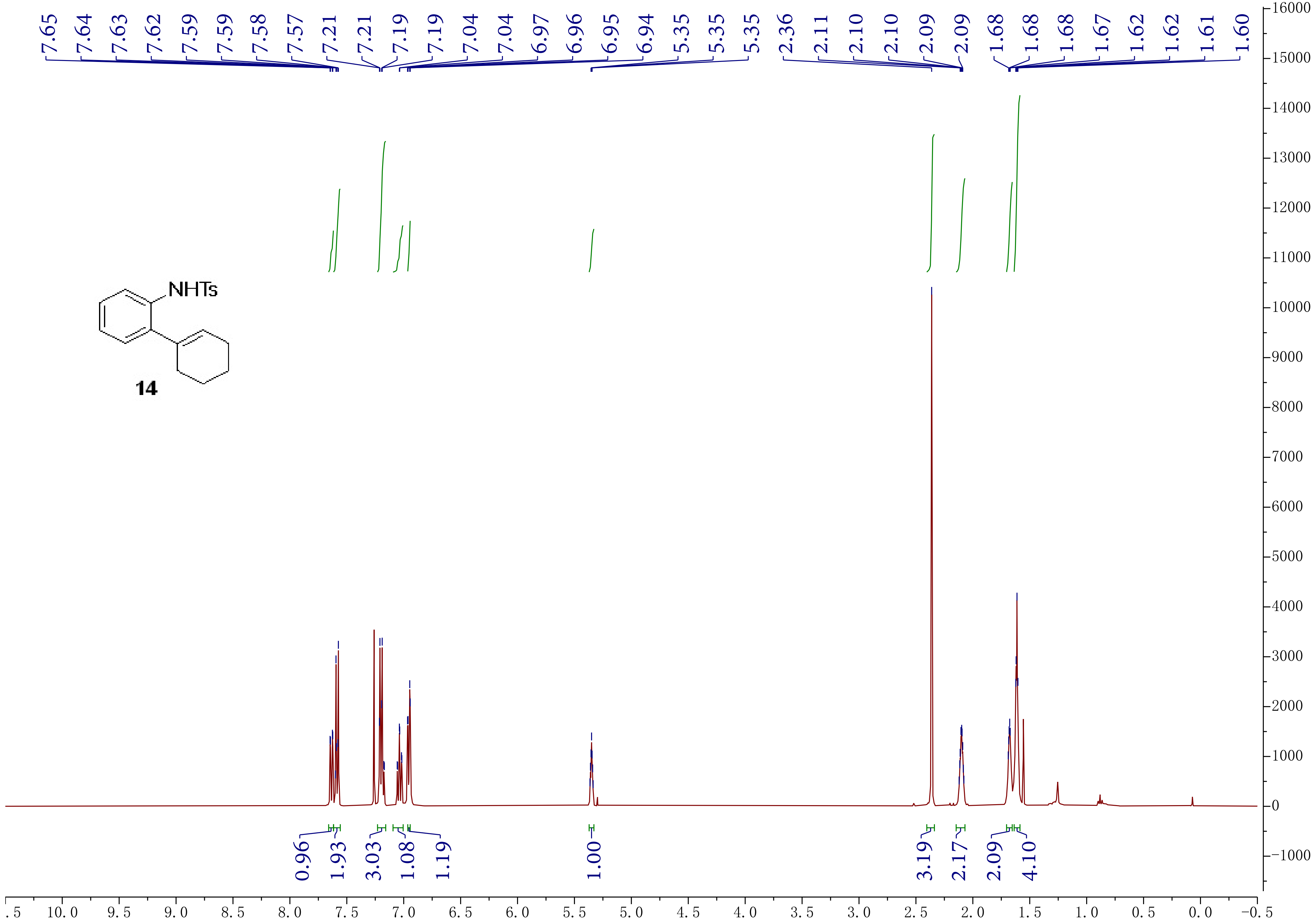
7.65 7.64 7.63 7.62 7.59 7.59 7.58 7.57 7.21 7.21 7.19 7.19 7.04 7.04 6.97 6.96 6.95 6.94 5.35 5.35 5.35 2.36 2.11 2.10 2.10 2.09 2.09 1.68 1.68 1.68 1.67 1.62 1.62 1.61 1.60
NHTs
14
0.96 1.93 3.03 1.08 1.19 1.00 3.19 2.17 2.09 4.10
16000 15000 14000 13000 12000 11000 10000 9000 8000 7000 6000 5000 4000 3000 2000 1000 0 −1000
.5 10.0 9.5 9.0 8.5 8.0 7.5 7.0 6.5 6.0 5.5 5.0 4.5 4.0 3.5 3.0 2.5 2.0 1.5 1.0 0.5 0.0 −0.5

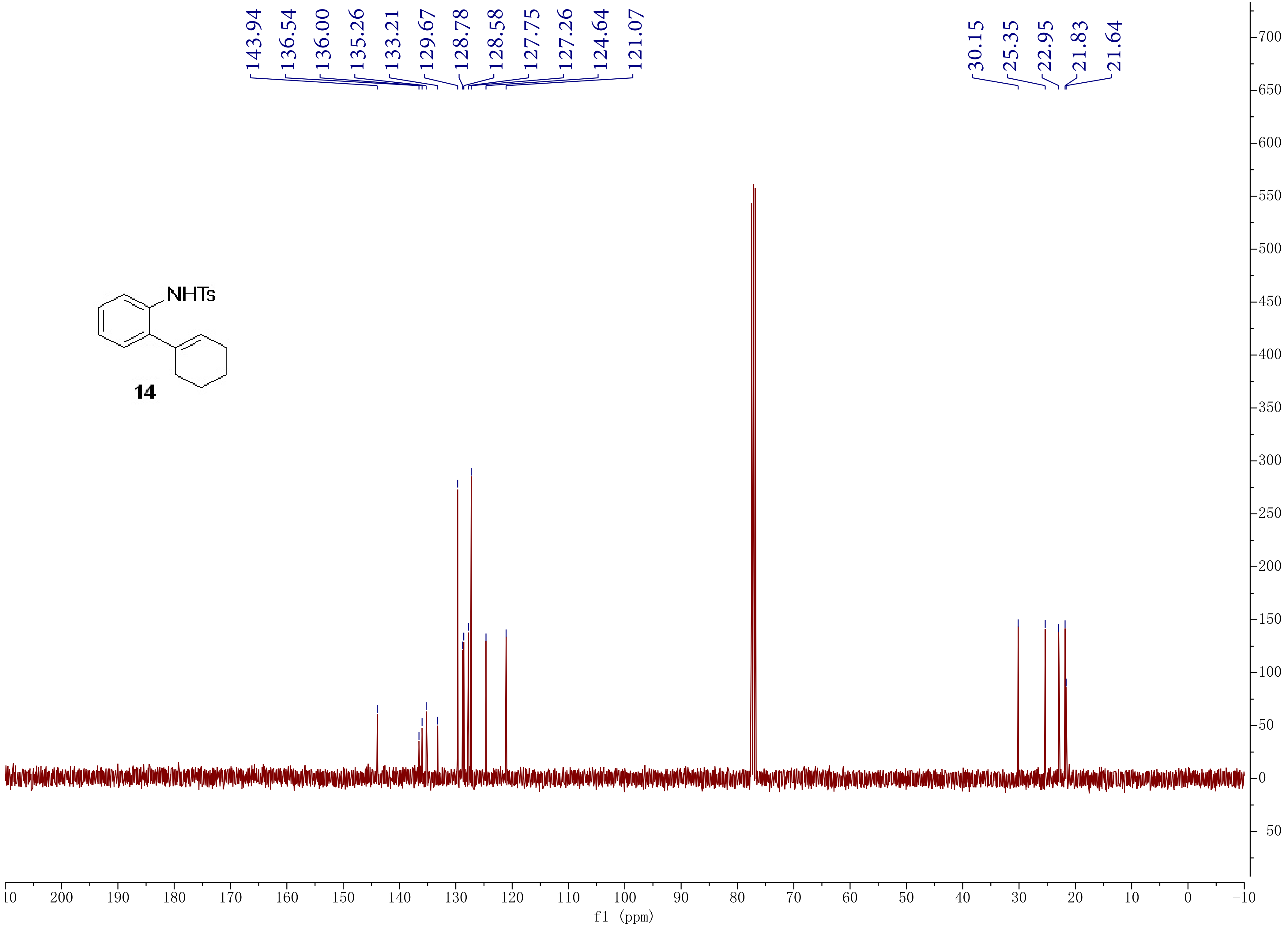

143.94
136.54
136.00
135.26
133.21
129.67
128.78
128.58
127.75
127.26
124.64
121.07
30.15
25.35
22.95
21.83
21.64
NHTs
14
f1 (ppm)

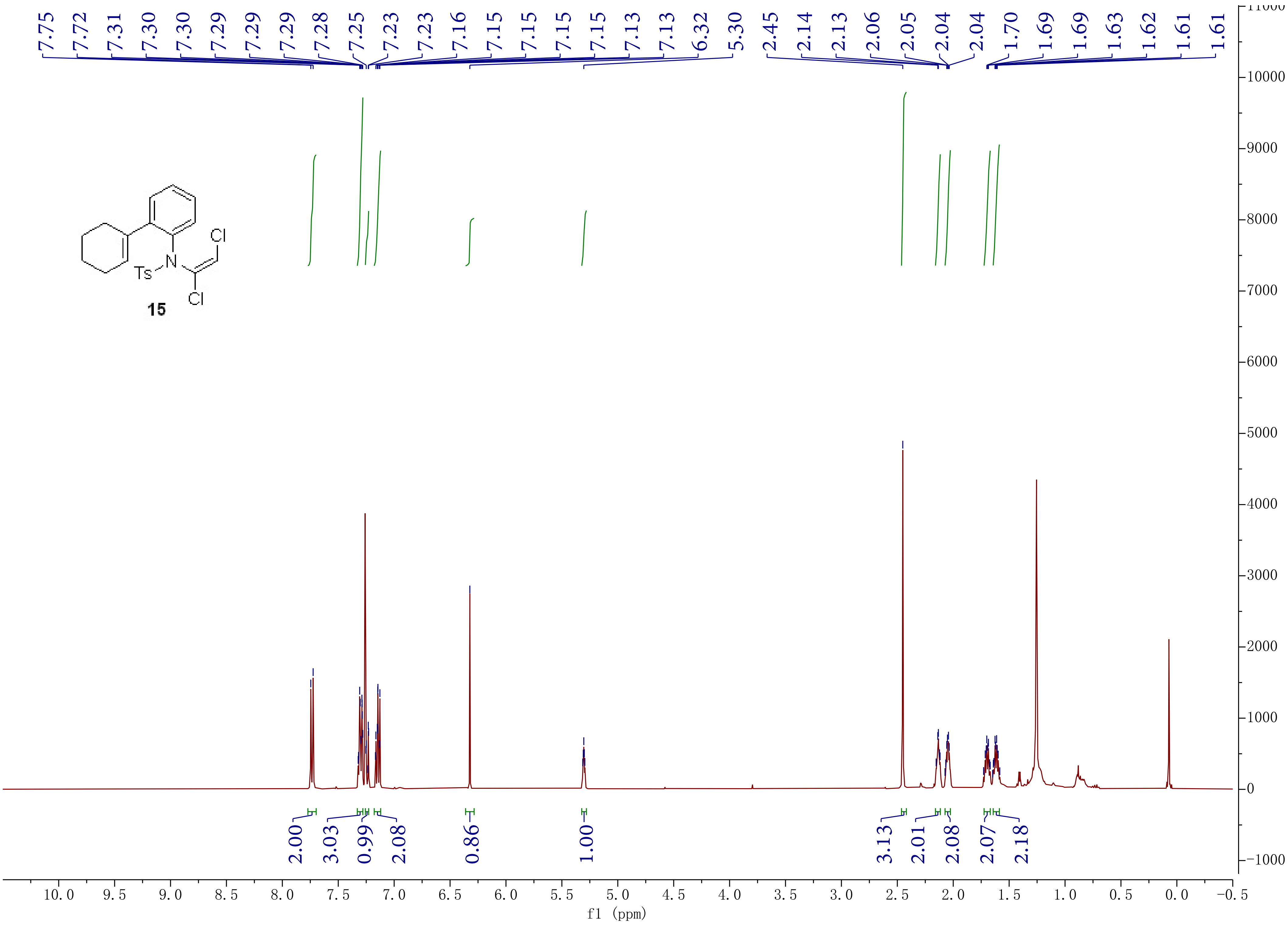

7.75 7.72 7.31 7.30 7.30 7.29 7.29 7.29 7.28 7.25 7.23 7.23 7.16 7.15 7.15 7.15 7.15 7.13 7.13 6.32 5.30 2.45 2.14 2.13 2.06 2.05 2.04 2.04 1.70 1.69 1.69 1.63 1.62 1.61 1.61
Ts
N
Cl
Cl
15
2.00 3.03 0.99 2.08 0.86 1.00 3.13 2.01 2.08 2.07 2.18
10.0 9.5 9.0 8.5 8.0 7.5 7.0 6.5 6.0 5.5 5.0 4.5 4.0 3.5 3.0 2.5 2.0 1.5 1.0 0.5 0.0 -0.5
f1 (ppm)
10000 9000 8000 7000 6000 5000 4000 3000 2000 1000 0 -1000

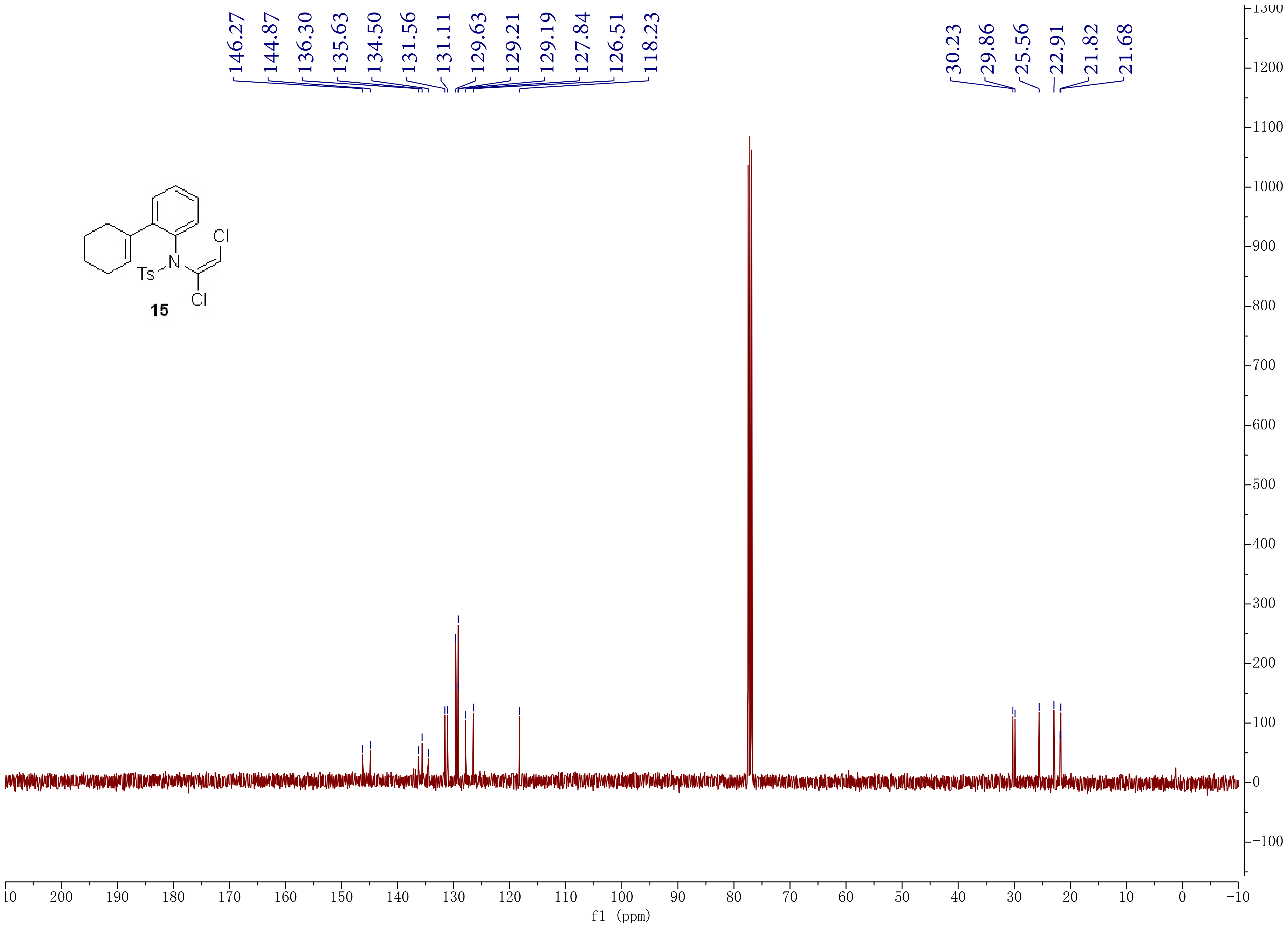
146.27
144.87
136.30
135.63
134.50
131.56
131.11
129.63
129.21
129.19
127.84
126.51
118.23
30.23
29.86
25.56
22.91
21.82
21.68
Cl
Ts
N
Cl
15
f1 (ppm)

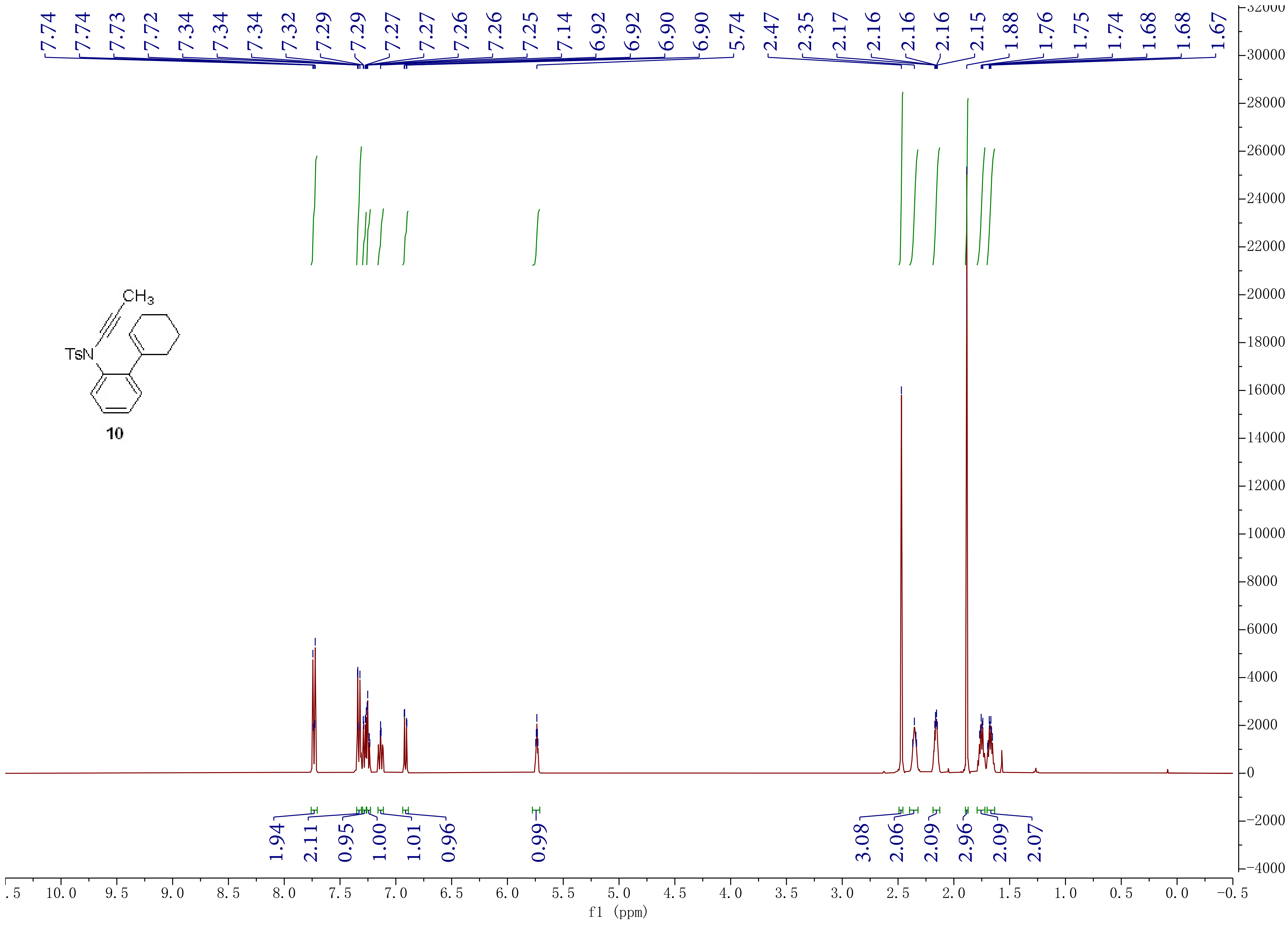

7.74 7.74 7.73 7.72 7.34 7.34 7.34 7.32 7.29 7.29 7.27 7.27 7.26 7.26 7.25 7.14 6.92 6.92 6.90 6.90 5.74 2.47 2.35 2.17 2.16 2.16 2.16 2.15 1.88 1.76 1.75 1.74 1.68 1.68 1.67
CH3
TsN
10
1.94 2.11 0.95 1.00 1.01 0.96 0.99 3.08 2.06 2.09 2.96 2.09 2.07
f1 (ppm)

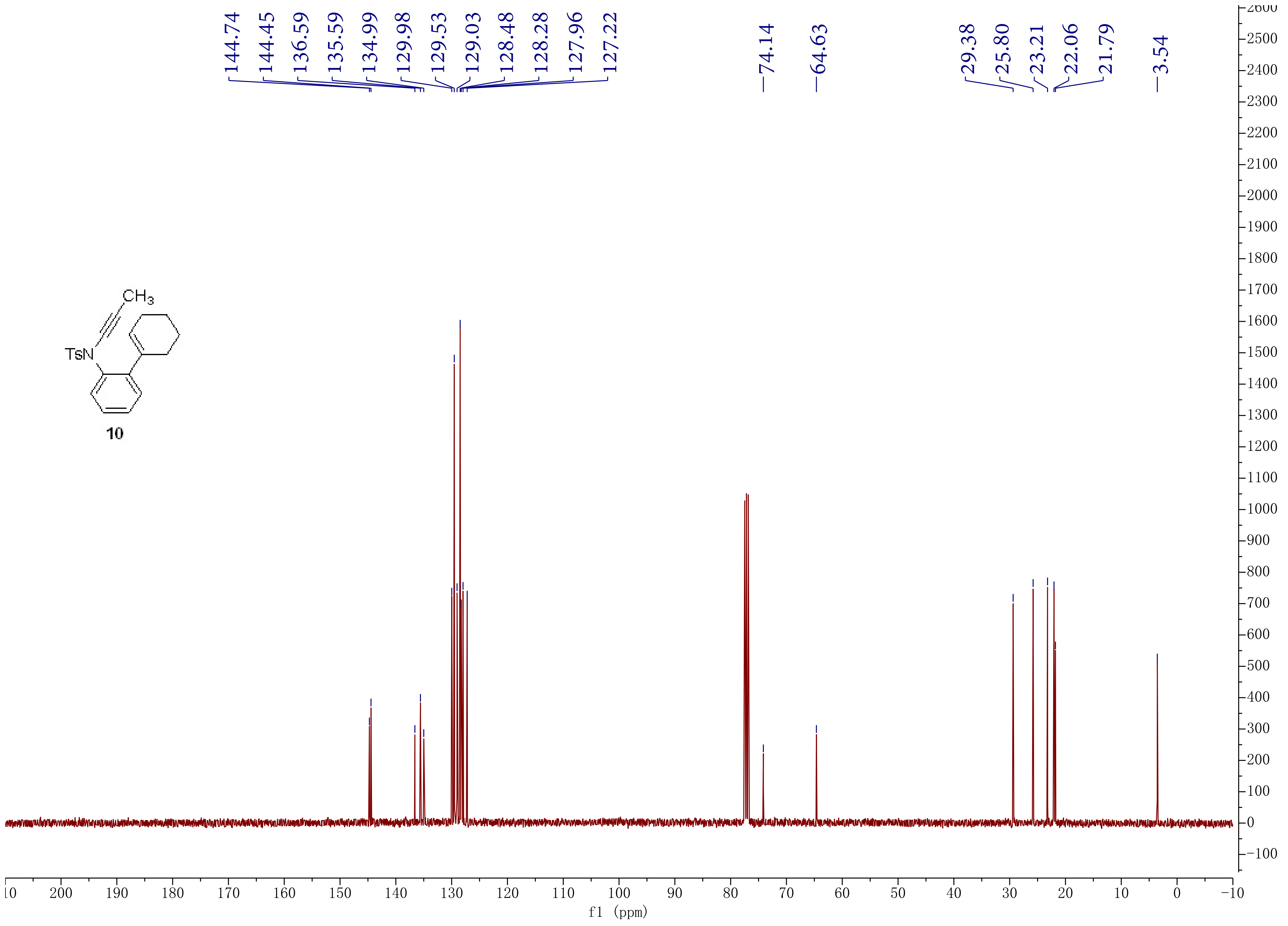
144.74
144.45
136.59
135.59
134.99
129.98
129.53
129.03
128.48
128.28
127.96
127.22
74.14
64.63
29.38
25.80
23.21
22.06
21.79
3.54
CH3
TsN
10
f1 (ppm)

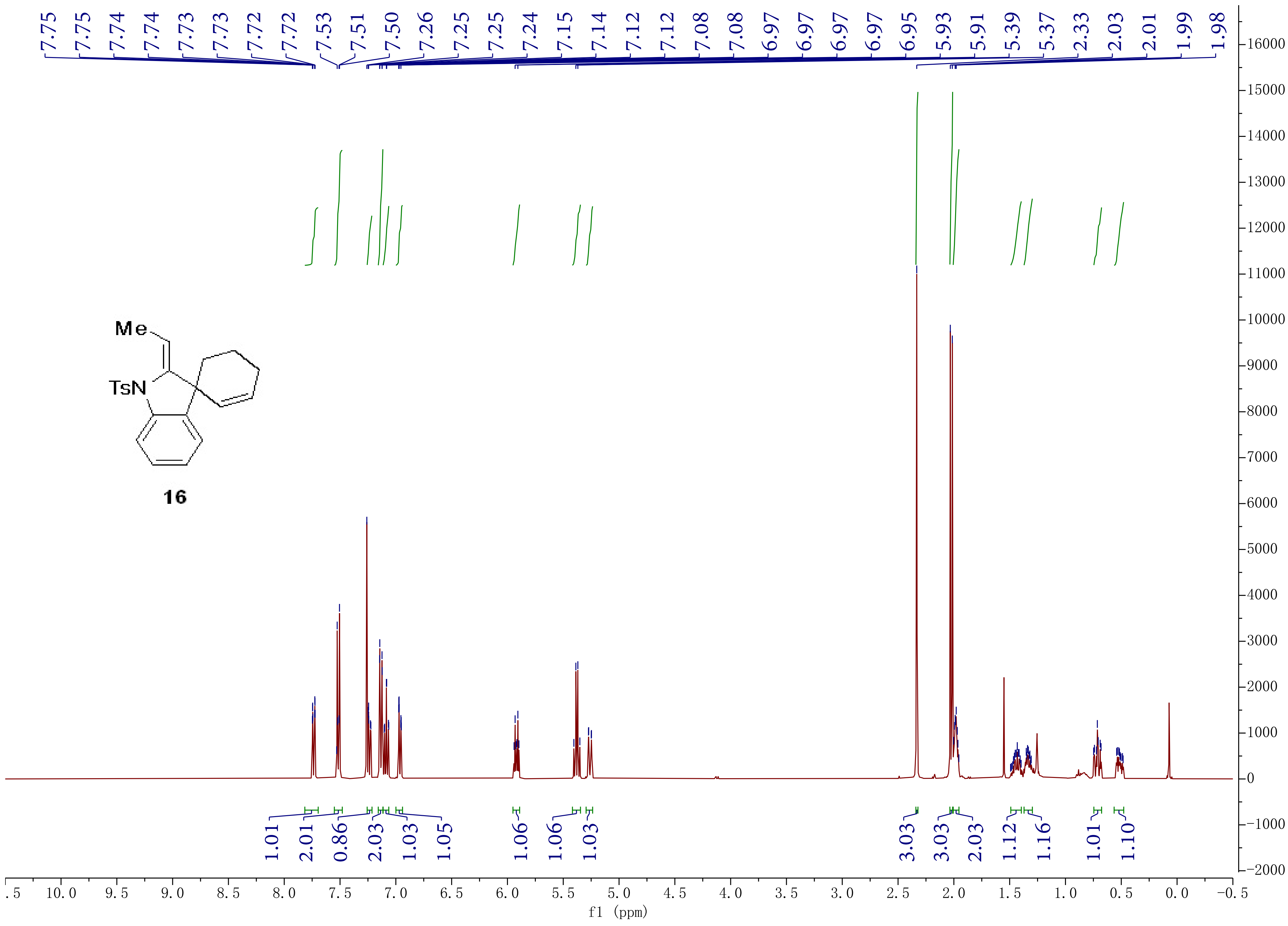

7.75
7.75
7.74
7.74
7.73
7.73
7.72
7.72
7.53
7.51
7.50
7.26
7.25
7.25
7.24
7.15
7.14
7.12
7.12
7.08
7.08
6.97
6.97
6.97
6.97
6.95
5.93
5.91
5.39
5.37
2.33
2.03
2.01
1.99
1.98
Me
TsN
16
1.01
2.01
0.86
2.03
1.03
1.05
1.06
1.06
1.03
3.03
3.03
2.03
1.12
1.16
1.01
1.10
f1 (ppm)

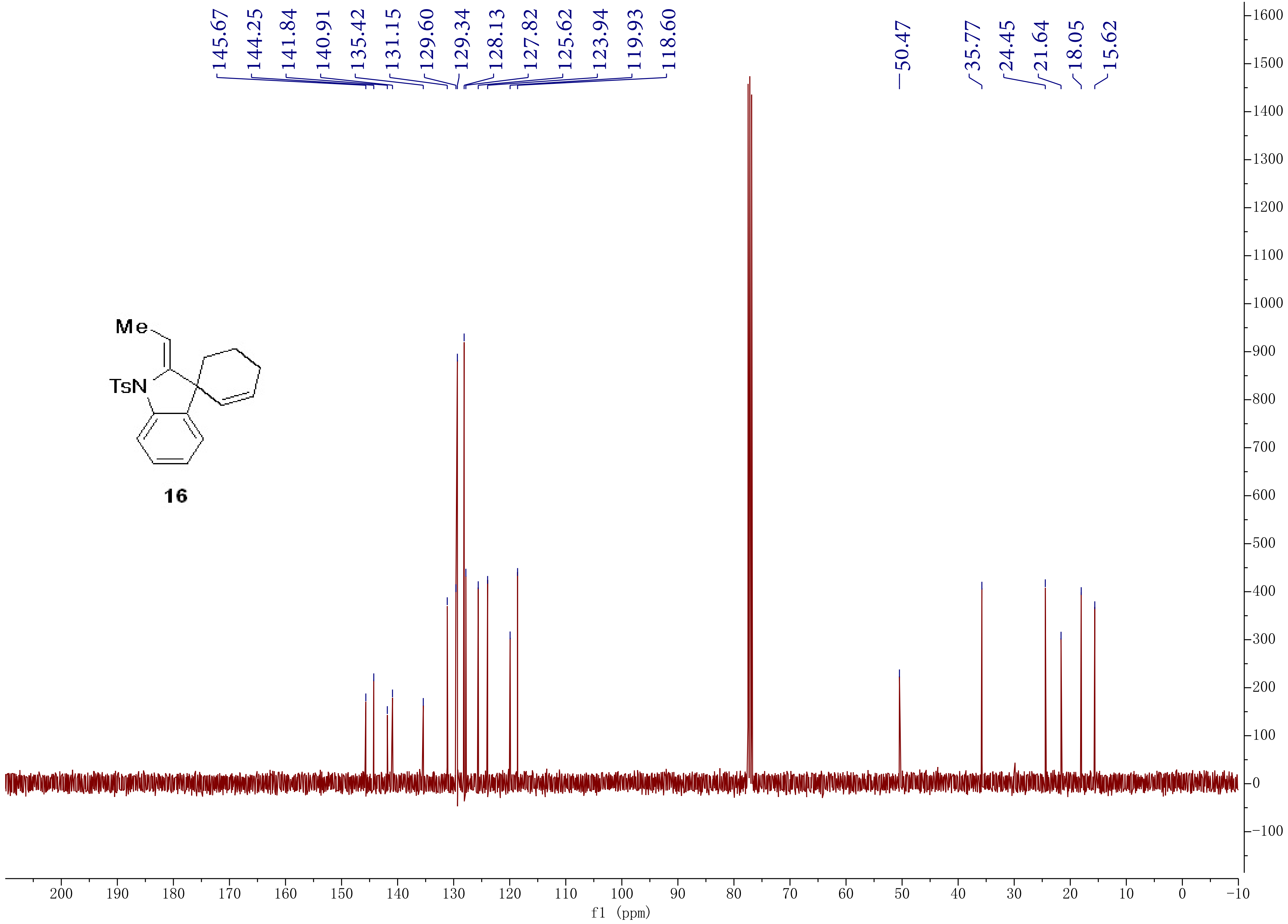
145.67
144.25
141.84
140.91
135.42
131.15
129.60
129.34
128.13
127.82
125.62
123.94
119.93
118.60
50.47
35.77
24.45
21.64
18.05
15.62
f1 (ppm)
Me
TsN

16

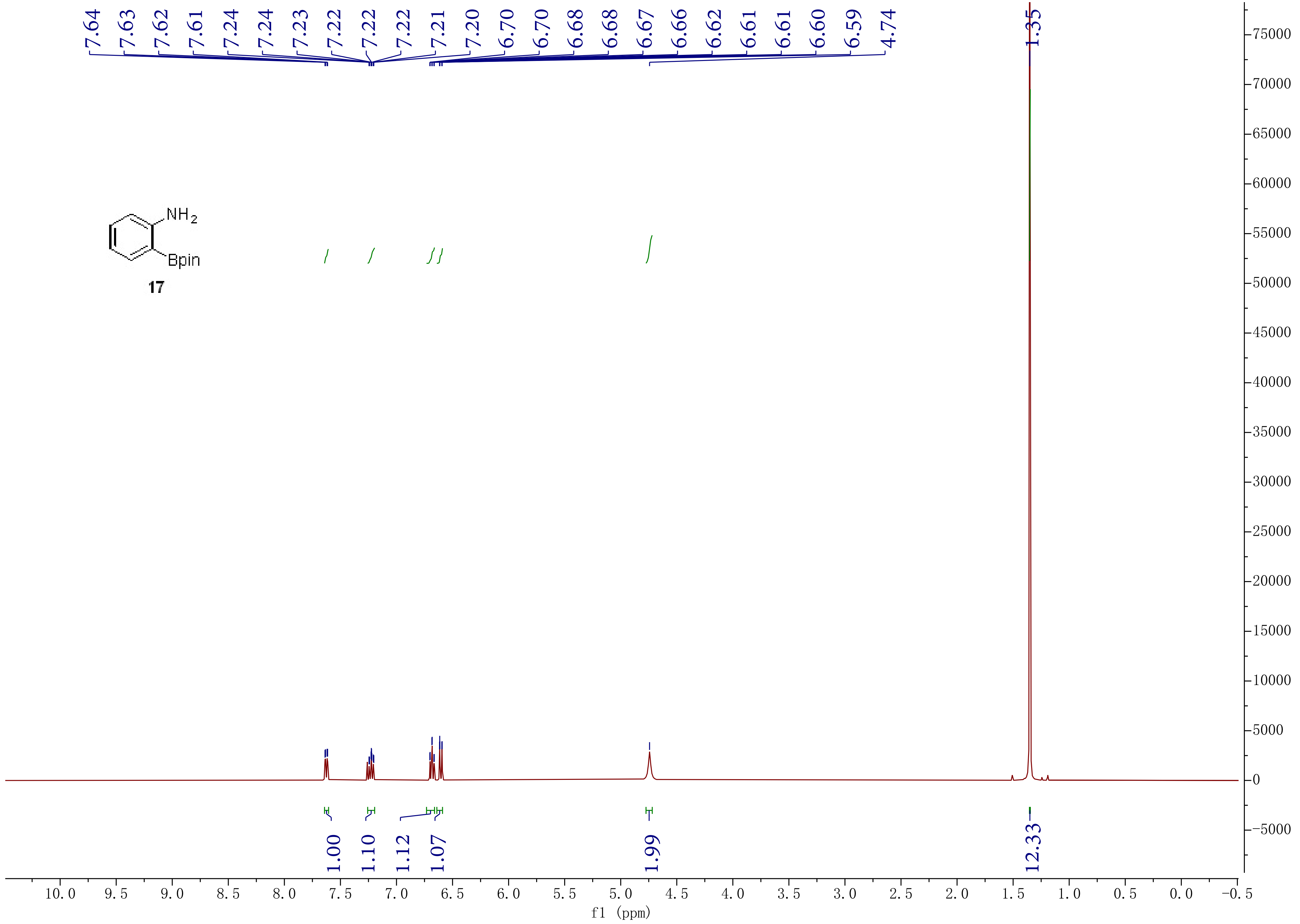
7.64 7.63 7.62 7.61 7.24 7.24 7.23 7.22 7.22 7.22 7.21 7.20 6.70 6.70 6.68 6.68 6.67 6.66 6.62 6.61 6.61 6.60 6.59 4.74
1.35
NH2
Bpin
17
1.00 1.10 1.12 1.07 1.99 12.33
10.0 9.5 9.0 8.5 8.0 7.5 7.0 6.5 6.0 5.5 5.0 4.5 4.0 3.5 3.0 2.5 2.0 1.5 1.0 0.5 0.0 -0.5
f1 (ppm)
75000 70000 65000 60000 55000 50000 45000 40000 35000 30000 25000 20000 15000 10000 5000 0 -5000

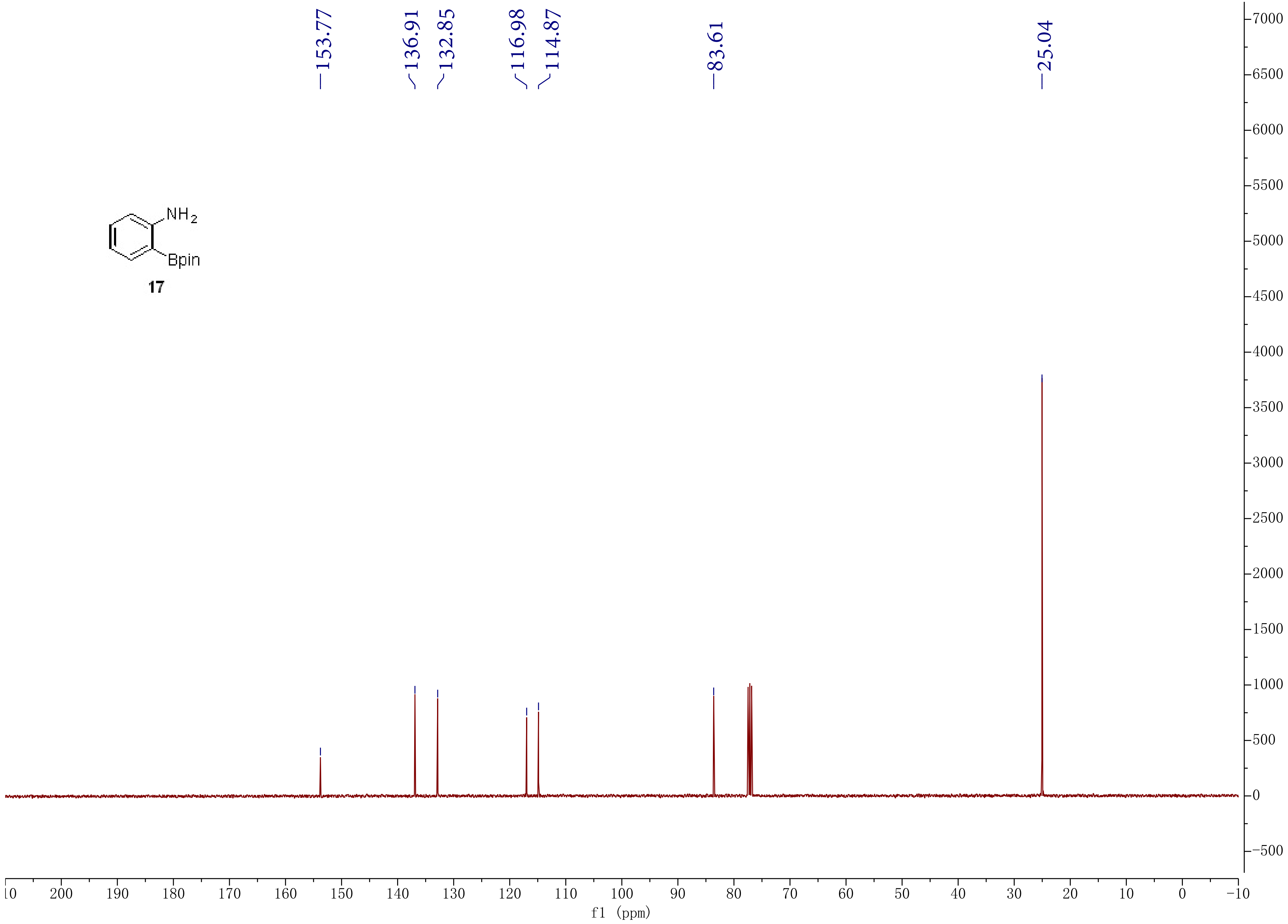
153.77
136.91
132.85
116.98
114.87
83.61
25.04
NH2
Bpin
17
7000
6500
6000
5500
5000
4500
4000
3500
3000
2500
2000
1500
1000
500
0
−500
10
200
190
180
170
160
150
140
130
120
110
100
90
80
70
60
50
40
30
20
10
0
−10
f1 (ppm)

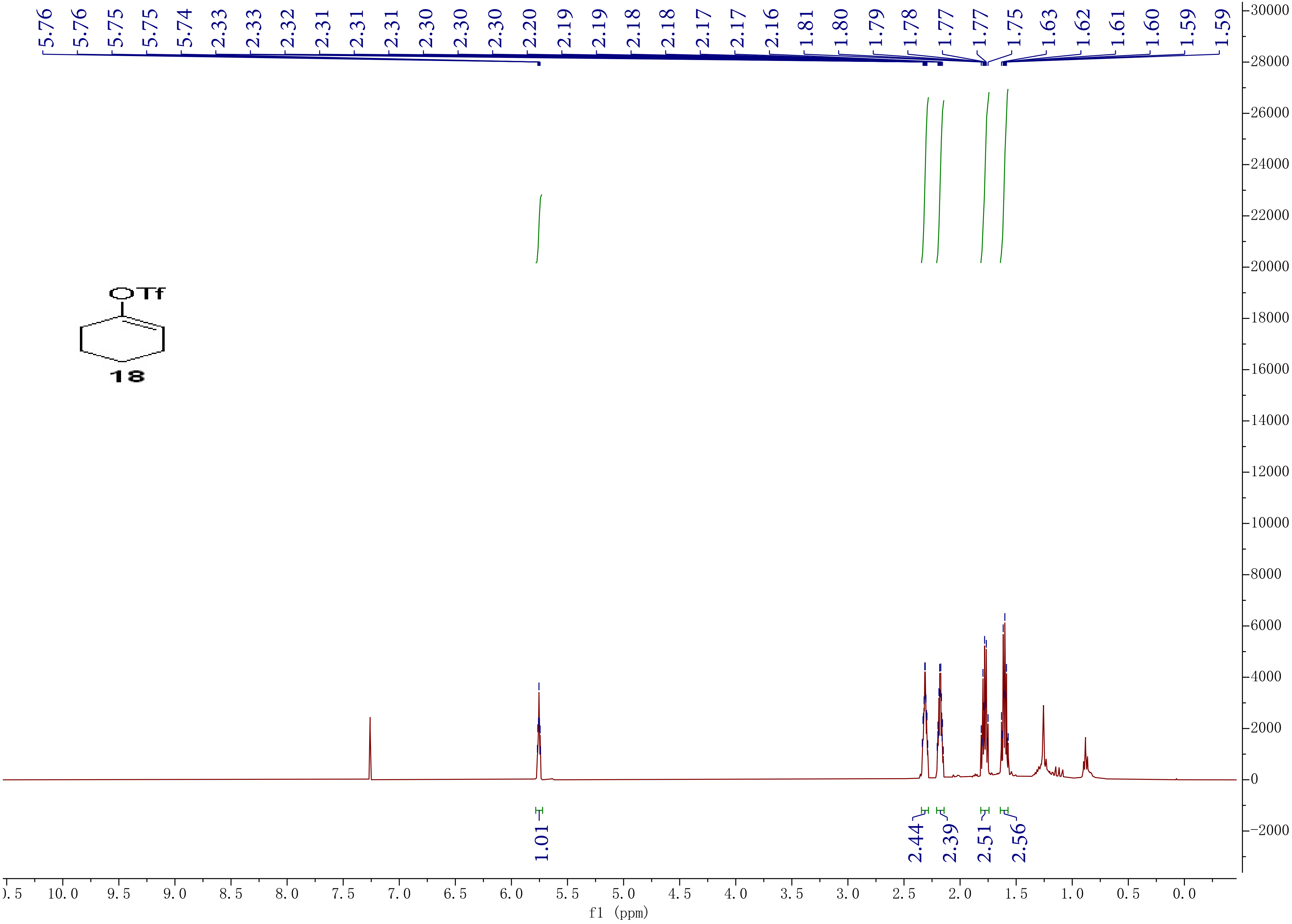
5.76 5.76 5.75 5.75 5.74 2.33 2.33 2.32 2.31 2.31 2.31 2.30 2.30 2.30 2.20 2.19 2.19 2.18 2.18 2.17 2.17 2.16 1.81 1.80 1.79 1.78 1.77 1.77 1.75 1.63 1.62 1.61 1.60 1.59 1.59
OTf
18
1.01
2.44
2.39
2.51
2.56
30000 28000 26000 24000 22000 20000 18000 16000 14000 12000 10000 8000 6000 4000 2000 0 −2000
10.0 9.5 9.0 8.5 8.0 7.5 7.0 6.5 6.0 5.5 5.0 4.5 4.0 3.5 3.0 2.5 2.0 1.5 1.0 0.5 0.0
f1 (ppm)

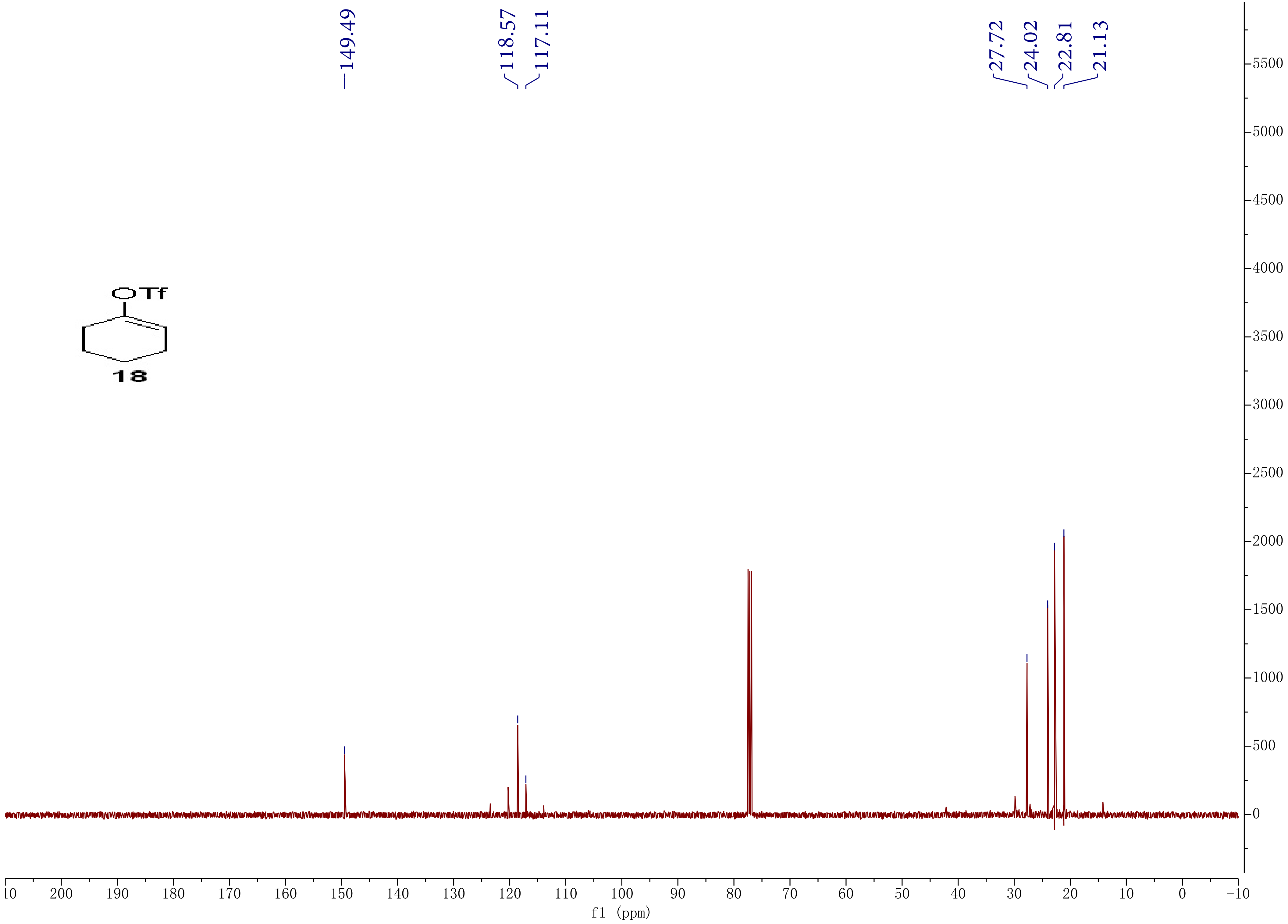
149.49
118.57
117.11
27.72
24.02
22.81
21.13
OTf
18
f1 (ppm)

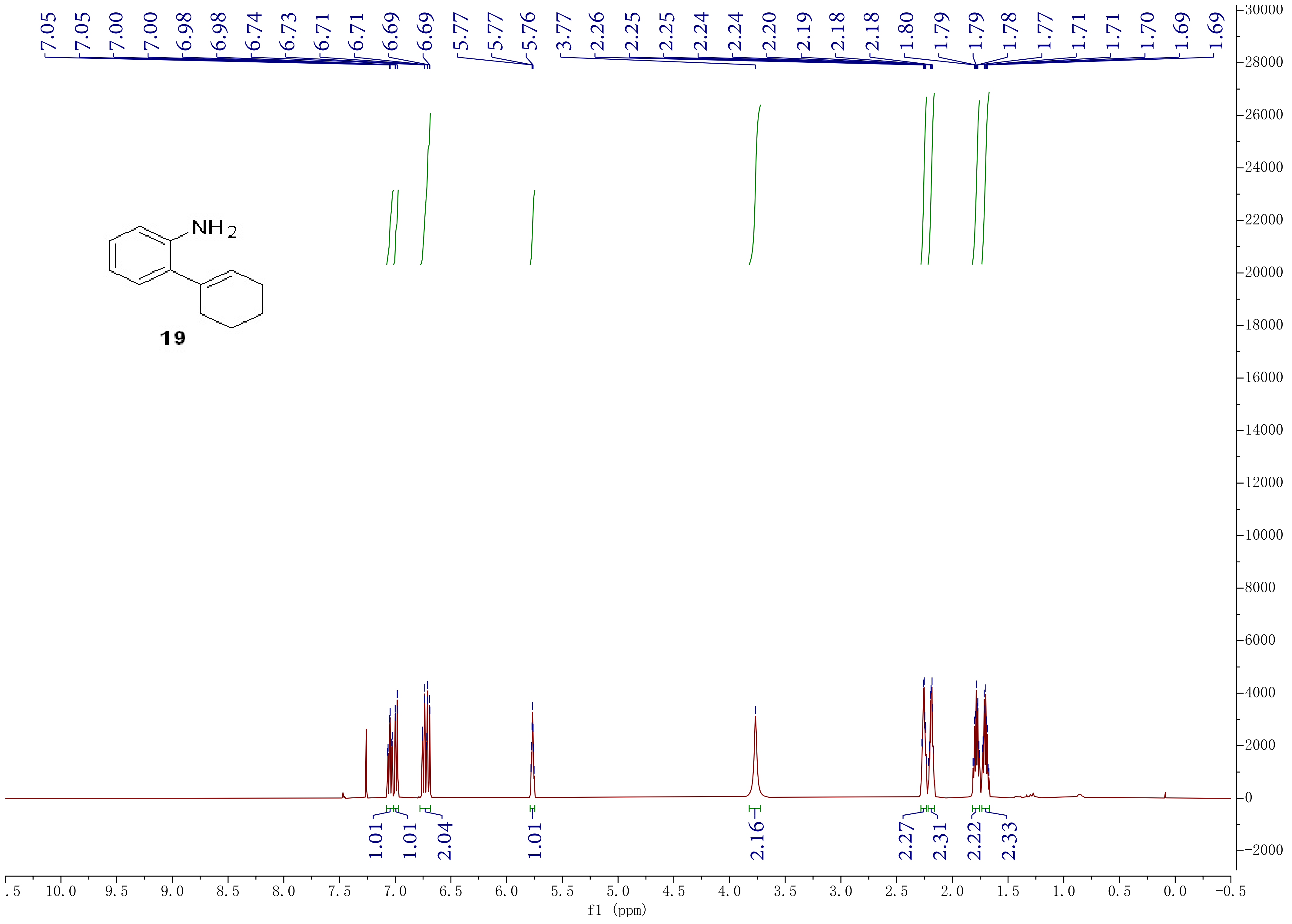
7.05 7.05 7.00 7.00 6.98 6.98 6.74 6.73 6.71 6.71 6.69 6.69 5.77 5.77 5.76 3.77 2.26 2.25 2.25 2.24 2.24 2.20 2.19 2.18 2.18 1.80 1.79 1.79 1.78 1.77 1.71 1.71 1.70 1.69 1.69
NH2
19
1.01 1.01 2.04 1.01 2.16 2.27 2.31 2.22 2.33
f1 (ppm)

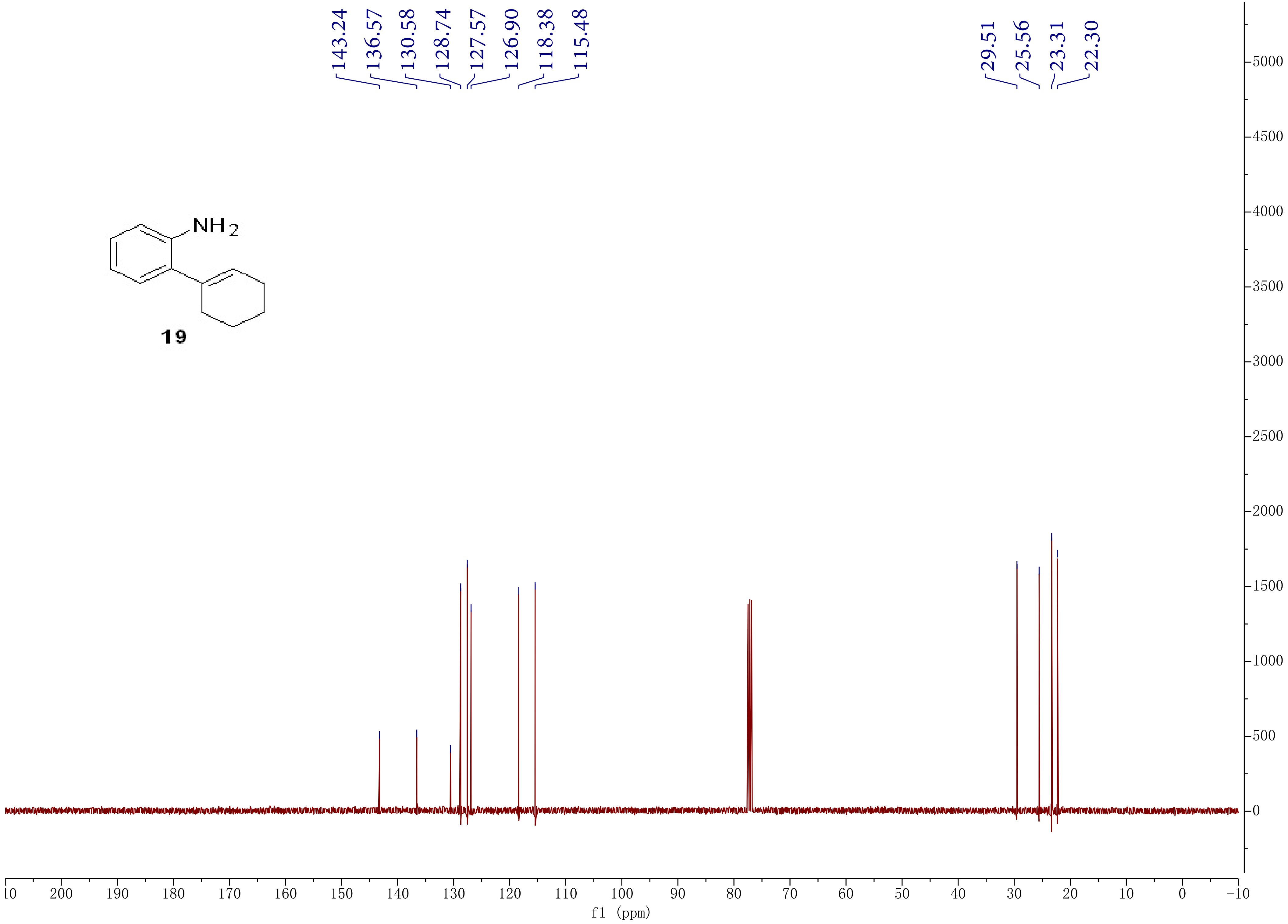
143.24
136.57
130.58
128.74
127.57
126.90
118.38
115.48
29.51
25.56
23.31
22.30
NH2
19
f1 (ppm)

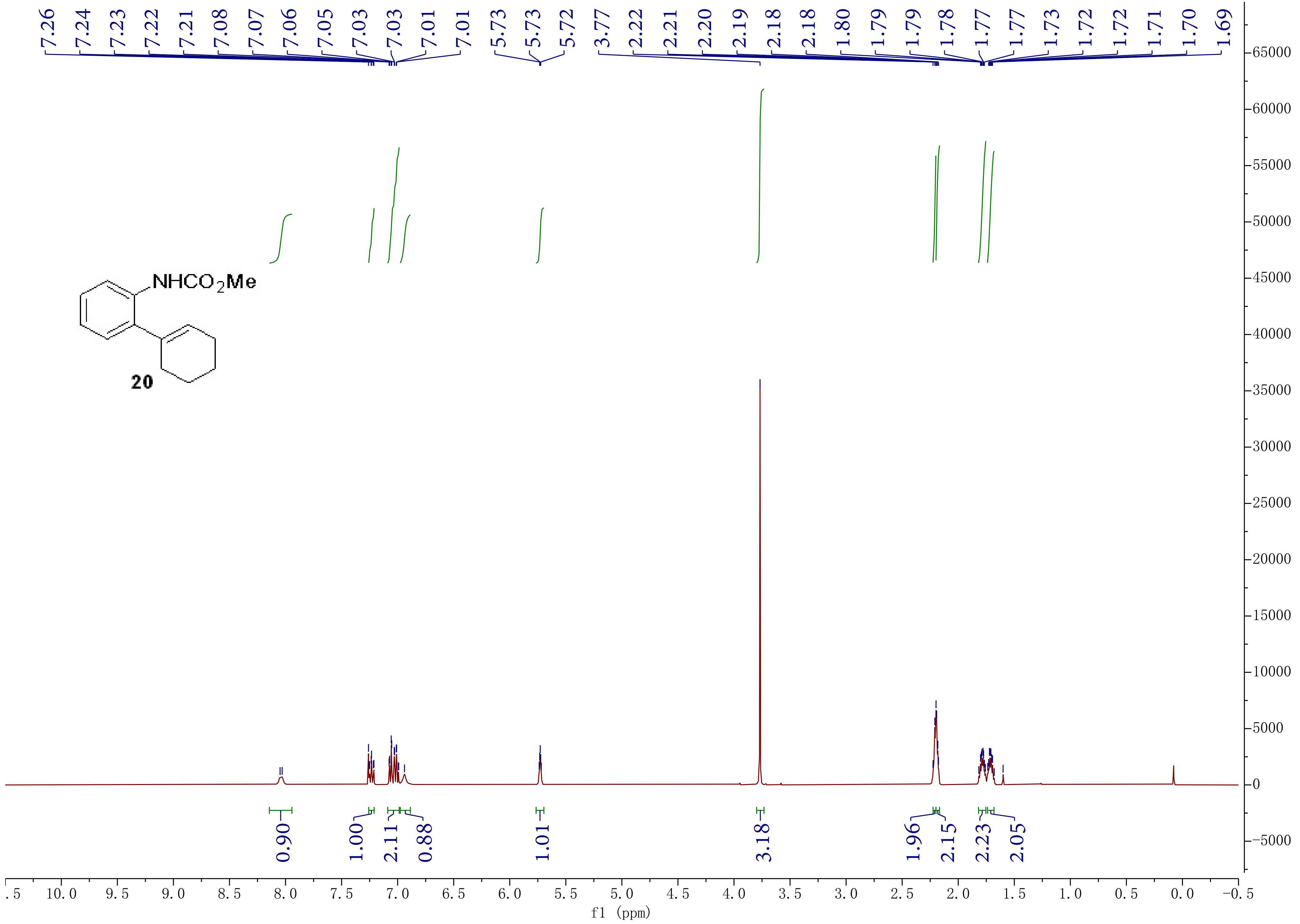
7.26
7.24
7.23
7.22
7.21
7.08
7.07
7.06
7.05
7.03
7.03
7.01
7.01
5.73
5.73
5.72
3.77
2.22
2.21
2.20
2.19
2.18
2.18
1.80
1.79
1.79
1.78
1.77
1.77
1.73
1.72
1.72
1.71
1.70
1.69
NHCO2Me
20
0.90
1.00
2.11
0.88
1.01
3.18
1.96
2.15
2.23
2.05
f1 (ppm)

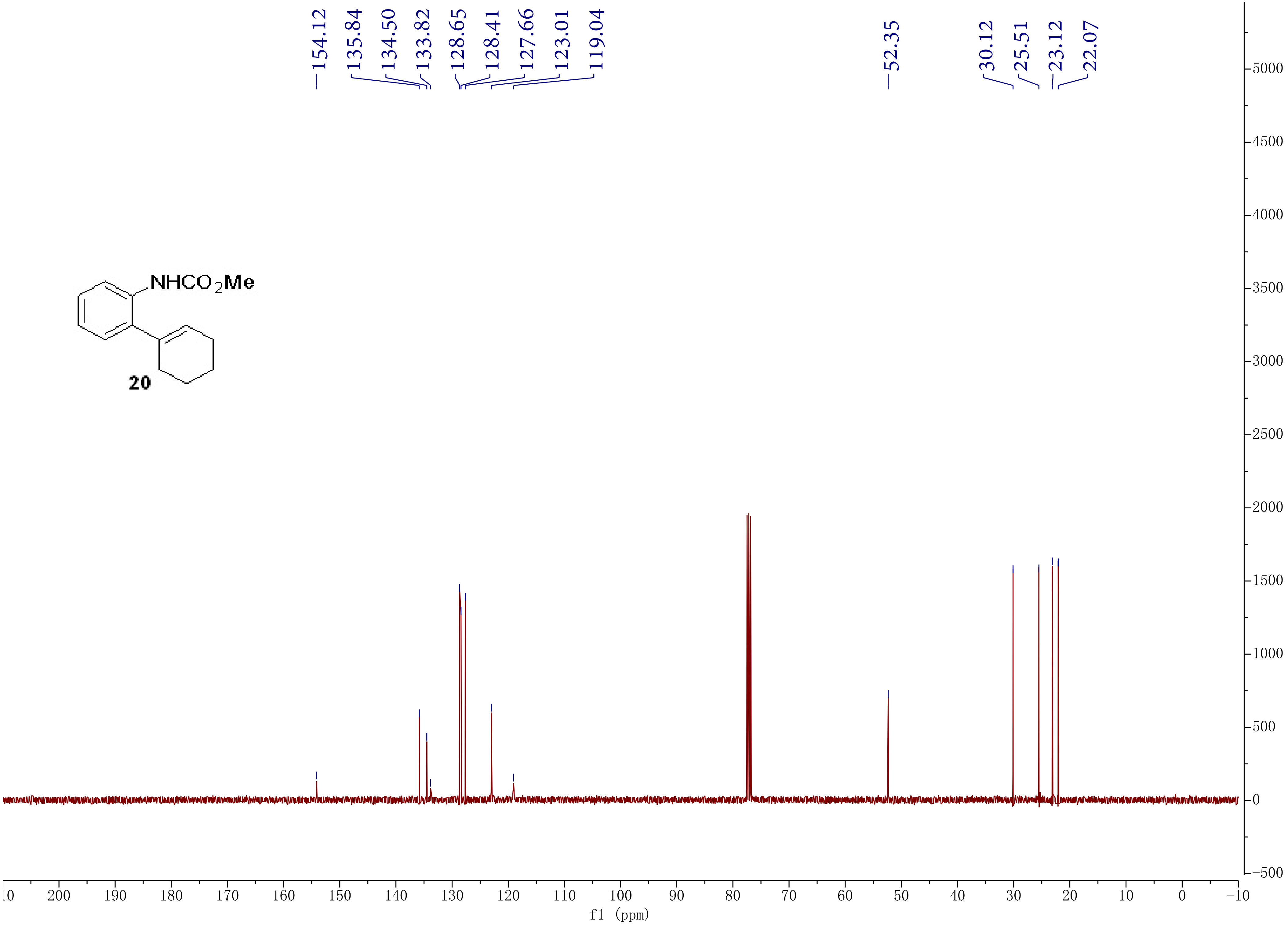
NHCO2Me
20
154.12
135.84
134.50
133.82
128.65
128.41
127.66
123.01
119.04
52.35
30.12
25.51
23.12
22.07
5000
4500
4000
3500
3000
2500
2000
1500
1000
500
0
−500
200
190
180
170
160
150
140
130
120
110
100
90
80
70
60
50
40
30
20
10
0
−10
f1 (ppm)

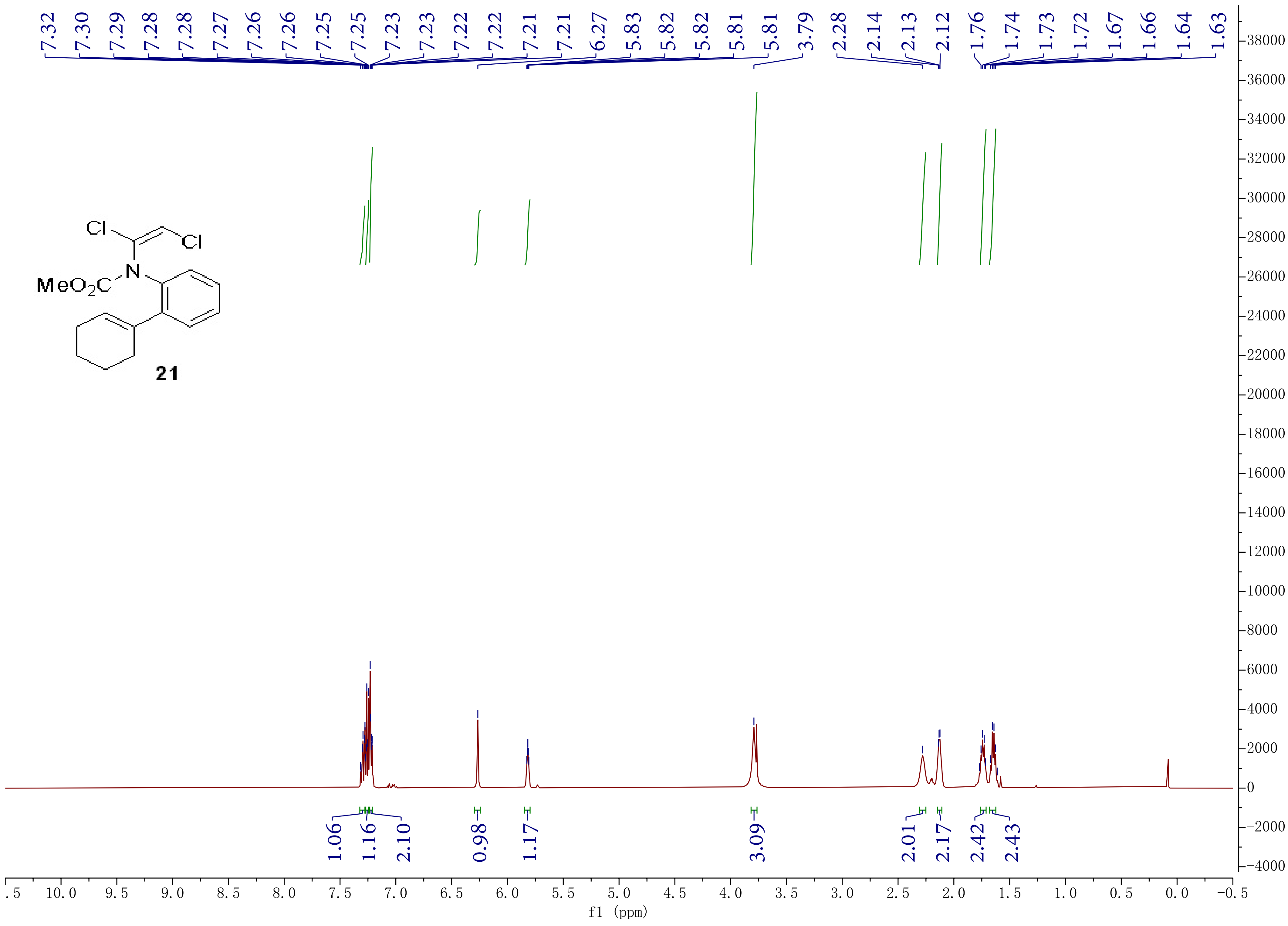
7.32 7.30 7.29 7.28 7.28 7.27 7.26 7.26 7.25 7.25 7.23 7.23 7.22 7.22 7.21 7.21 6.27 5.83 5.82 5.82 5.81 5.81 3.79 2.28 2.14 2.13 2.12 1.76 1.74 1.73 1.72 1.67 1.66 1.64 1.63
Cl
Cl
N
MeO2C
21
1.06 1.16 2.10 0.98 1.17 3.09 2.01 2.17 2.42 2.43
f1 (ppm)

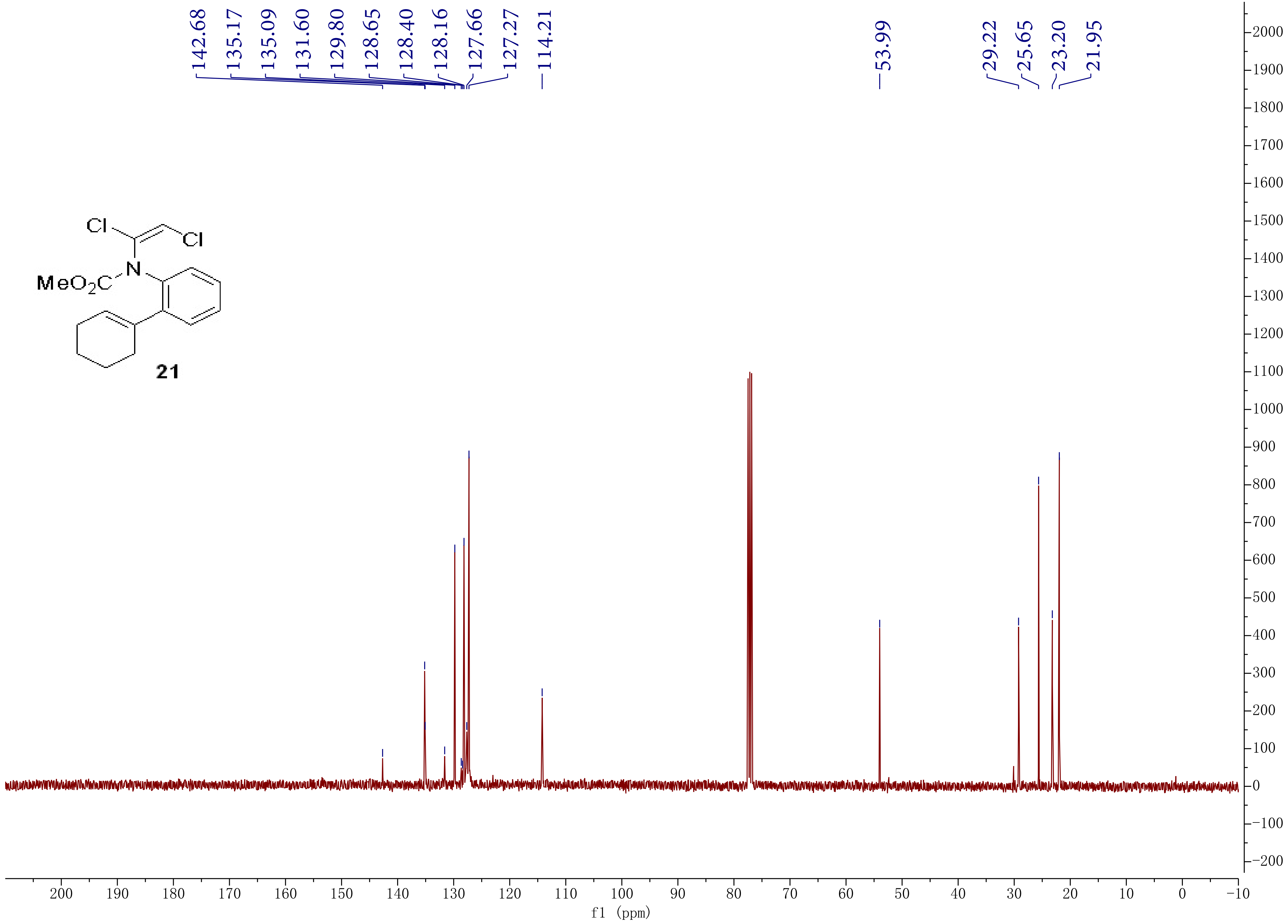
142.68
135.17
135.09
131.60
129.80
128.65
128.40
128.16
127.66
127.27
114.21
53.99
29.22
25.65
23.20
21.95
Cl
Cl
MeO2C
N
f1 (ppm)
21

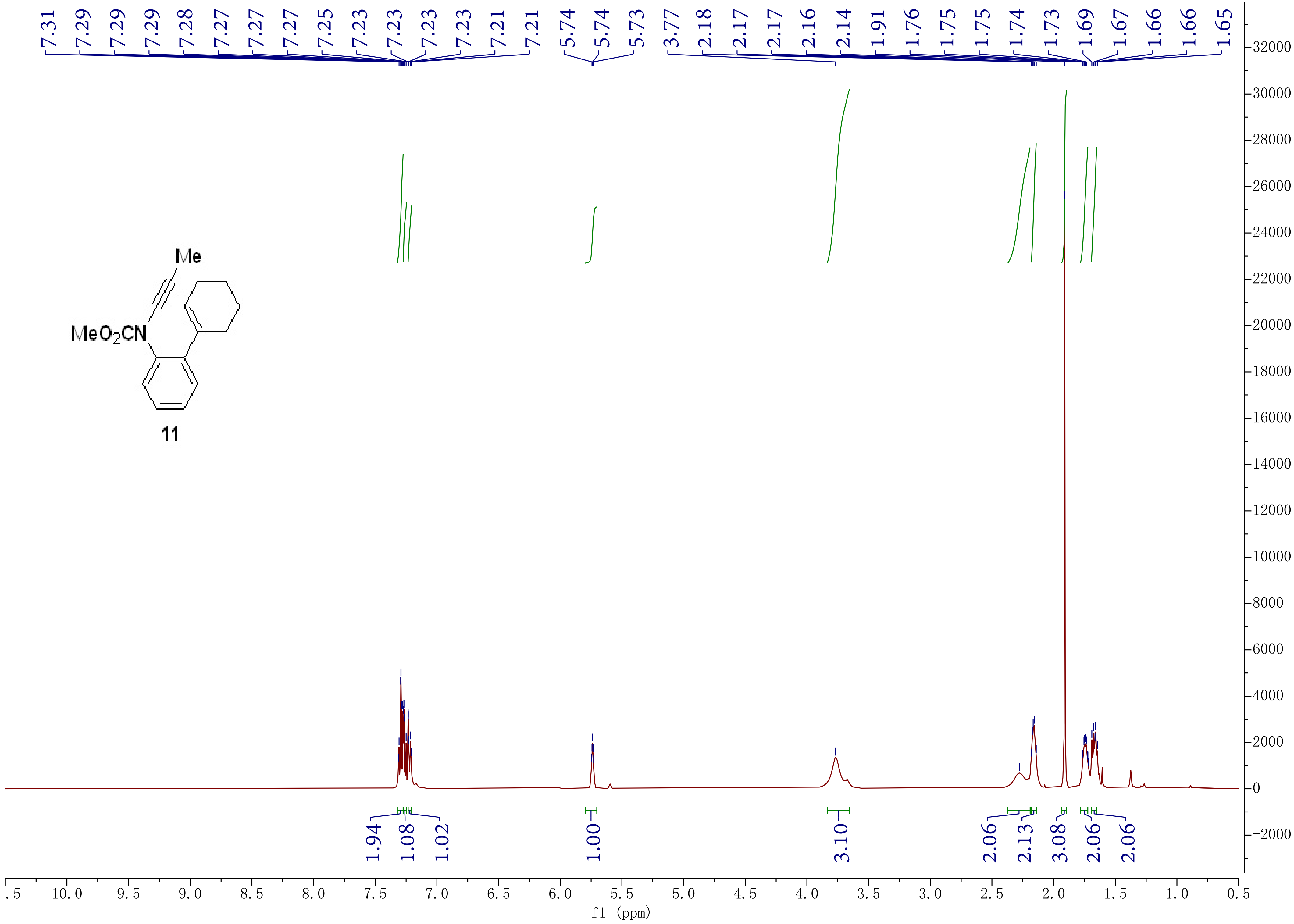

7.31
7.29
7.29
7.29
7.28
7.27
7.27
7.27
7.25
7.23
7.23
7.23
7.23
7.21
7.21
5.74
5.74
5.73
3.77
2.18
2.17
2.17
2.16
2.14
1.91
1.76
1.75
1.75
1.74
1.73
1.69
1.67
1.66
1.66
1.65
Me
MeO_2CN
11
1.94
1.08
1.02
1.00
3.10
2.06
2.13
3.08
2.06
2.06
f1 (ppm)

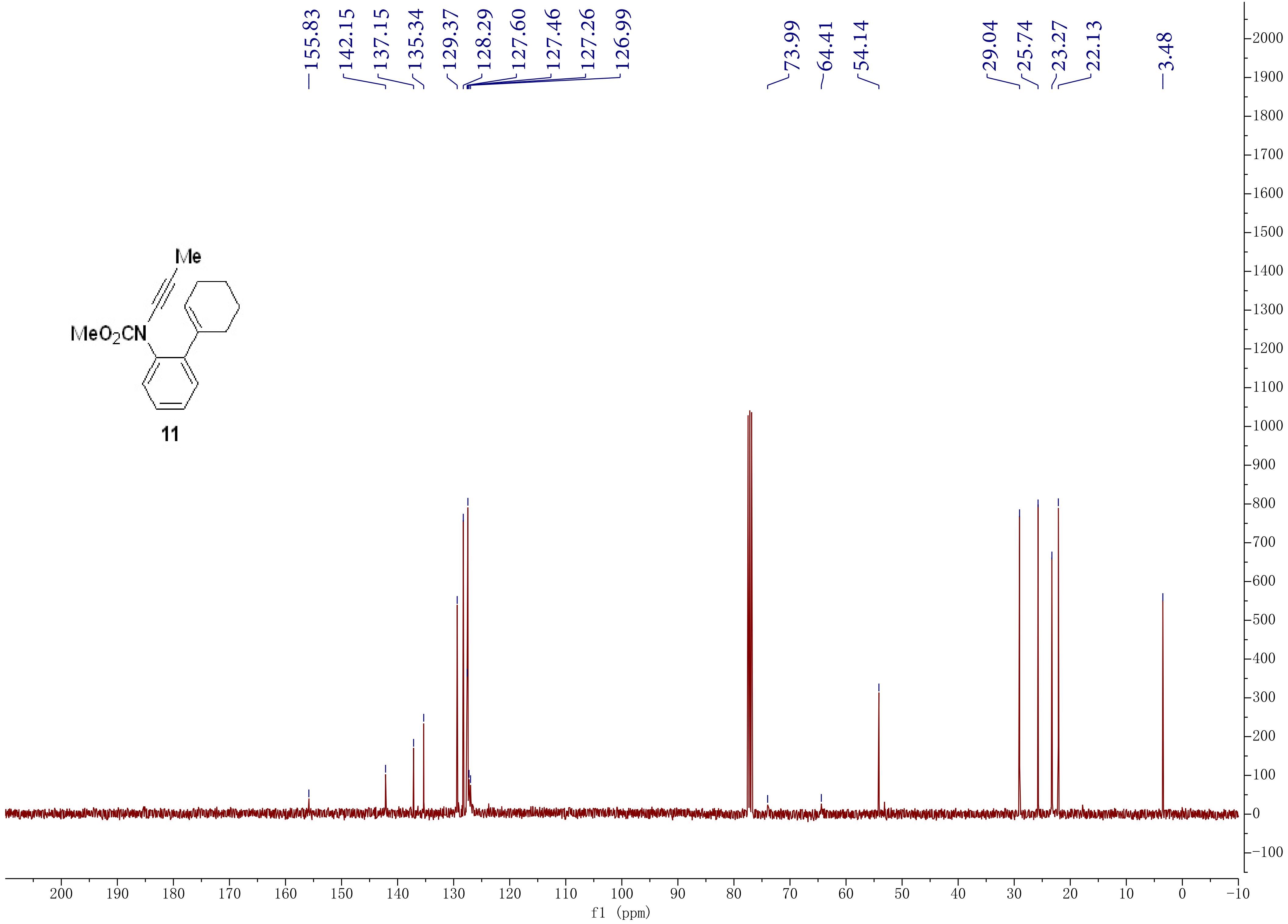
155.83
142.15
137.15
135.34
129.37
128.29
127.60
127.46
127.26
126.99
73.99
64.41
54.14
29.04
25.74
23.27
22.13
3.48
Me
MeO2CN
11
f1 (ppm)

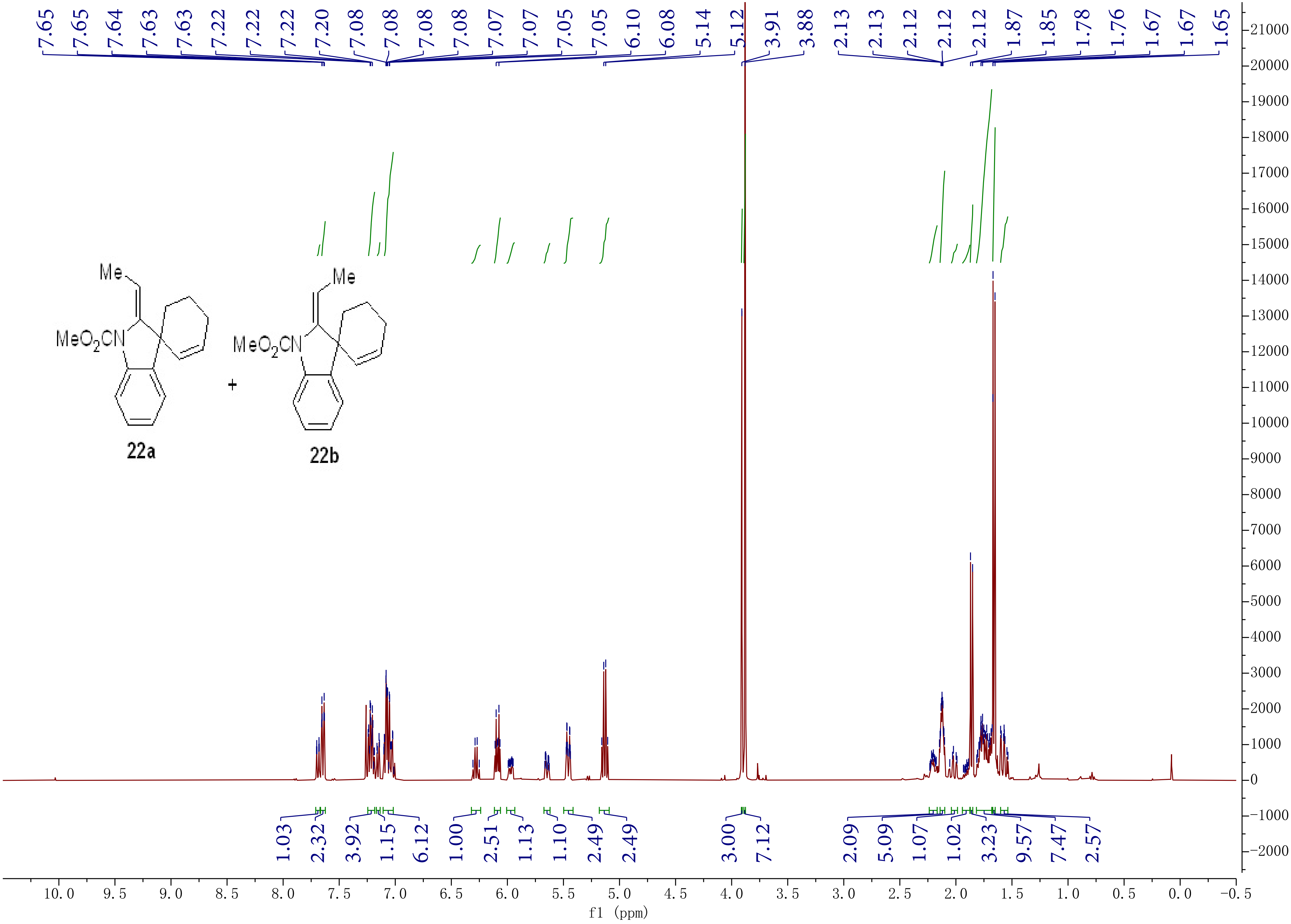
7.65
7.65
7.64
7.63
7.63
7.22
7.22
7.22
7.20
7.08
7.08
7.08
7.08
7.07
7.07
7.05
7.05
6.10
6.08
5.14
5.12
3.91
3.88
2.13
2.13
2.12
2.12
2.12
1.87
1.85
1.78
1.76
1.67
1.67
1.65
Me
MeO2CN
22a
+
22b
1.03
2.32
3.92
1.15
6.12
1.00
2.51
1.13
1.10
2.49
2.49
3.00
7.12
2.09
5.09
1.07
1.02
3.23
9.57
7.47
2.57
f1 (ppm)

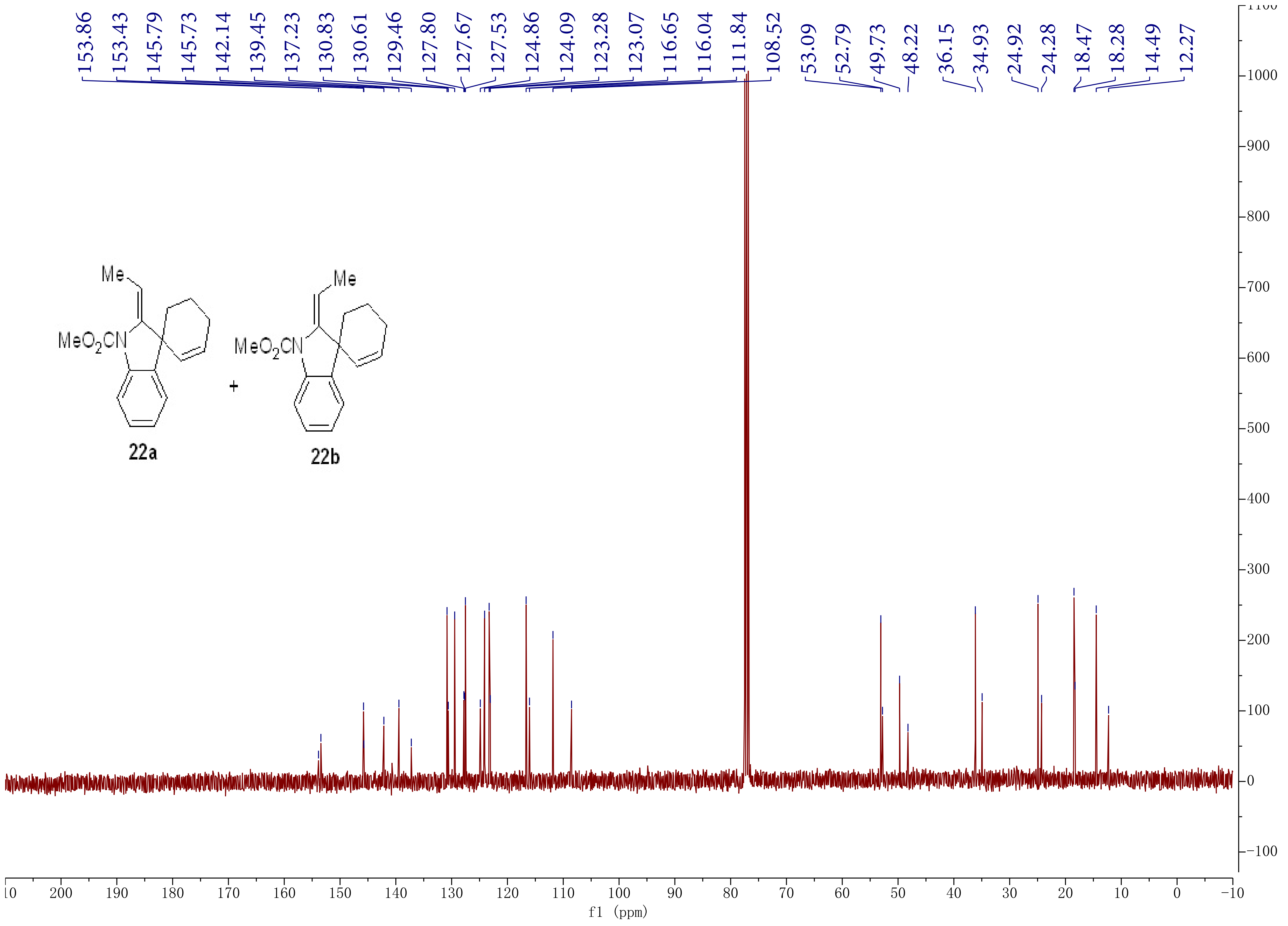
153.86
153.43
145.79
145.73
142.14
139.45
137.23
130.83
130.61
129.46
127.80
127.67
127.53
124.86
124.09
123.28
123.07
116.65
116.04
111.84
108.52
53.09
52.79
49.73
48.22
36.15
34.93
24.92
24.28
18.47
18.28
14.49
12.27
Me
MeO_2CN
22a
+
22b
f1 (ppm)

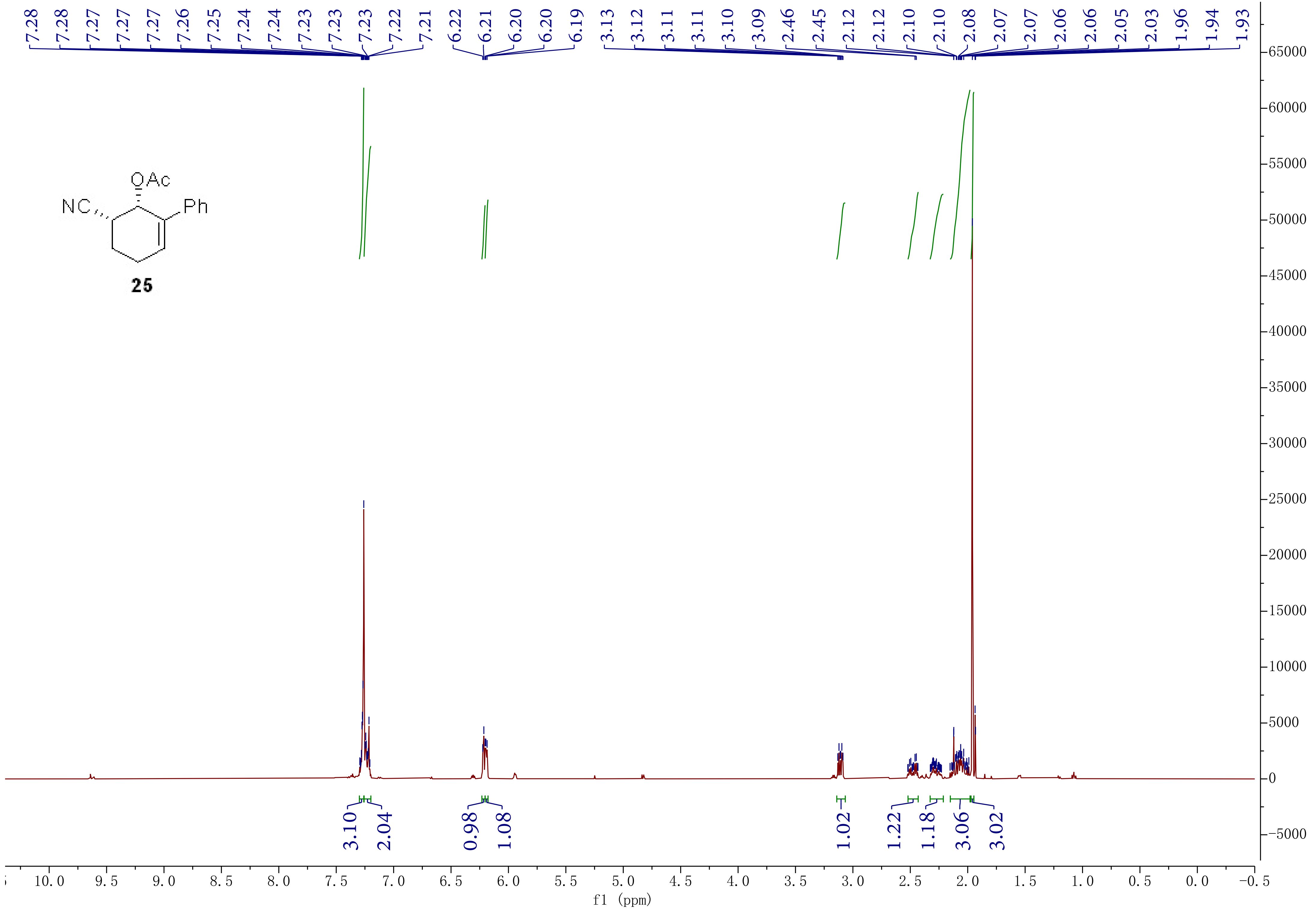

OAc
NC
Ph
25
7.28
7.28
7.27
7.27
7.27
7.26
7.25
7.24
7.24
7.23
7.23
7.23
7.22
7.21
6.22
6.21
6.20
6.20
6.19
3.13
3.12
3.11
3.11
3.10
3.09
2.46
2.45
2.12
2.12
2.10
2.10
2.08
2.07
2.07
2.06
2.06
2.05
2.03
1.96
1.94
1.93
3.10
2.04
0.98
1.08
1.02
1.22
1.18
3.06
3.02
f1 (ppm)

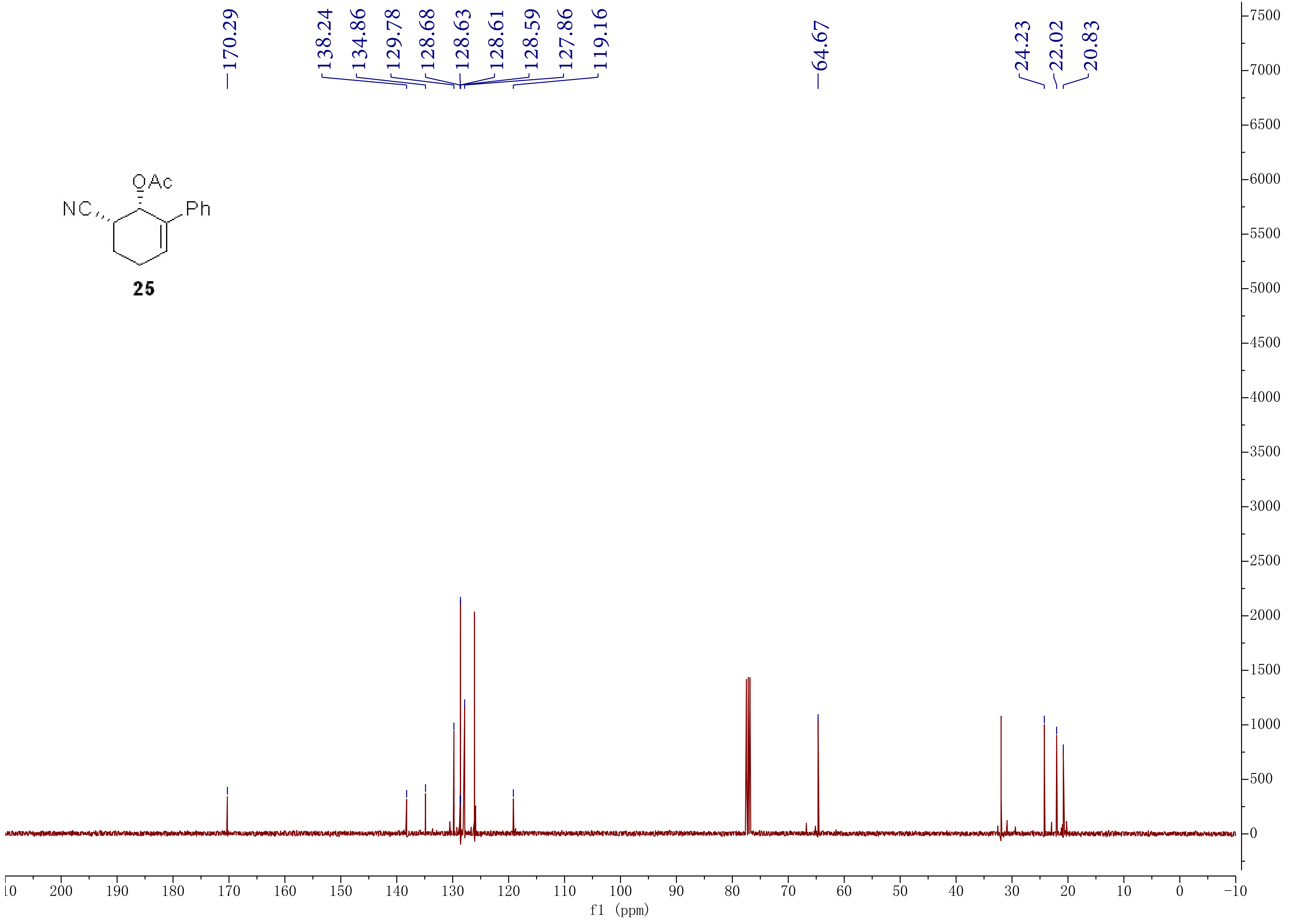

170.29
138.24
134.86
129.78
128.68
128.63
128.61
128.59
127.86
119.16
64.67
24.23
22.02
20.83
OAc
NC
Ph
25
f1 (ppm)

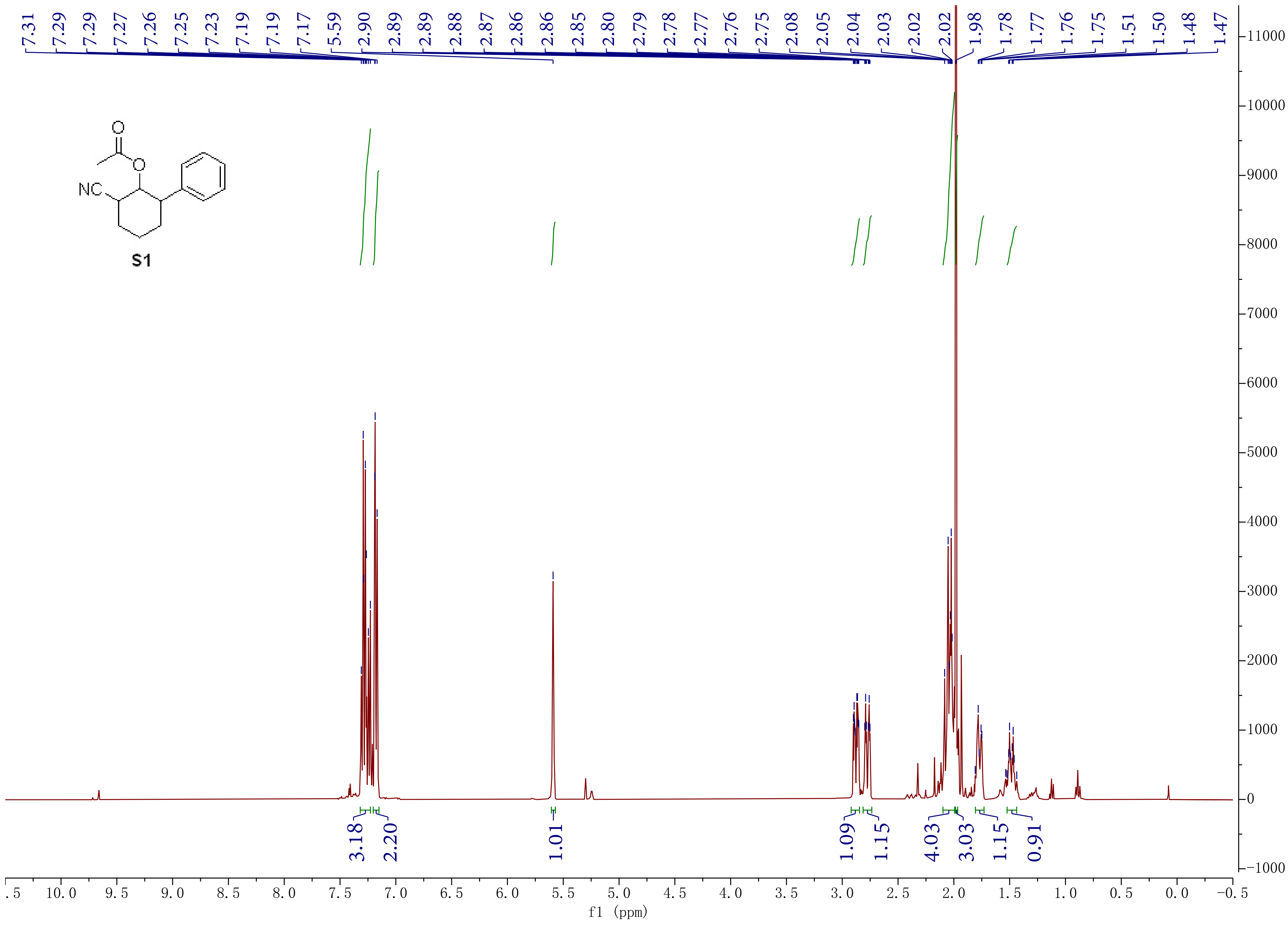

7.31 7.29 7.29 7.27 7.26 7.25 7.23 7.19 7.19 7.17 5.59 2.90 2.89 2.89 2.88 2.87 2.86 2.86 2.85 2.80 2.79 2.78 2.77 2.76 2.75 2.08 2.05 2.04 2.03 2.02 2.02 1.98 1.78 1.77 1.76 1.75 1.51 1.50 1.48 1.47
NC
O
S1
3.18 2.20 1.01 1.09 1.15 4.03 3.03 1.15 0.91
f1 (ppm)

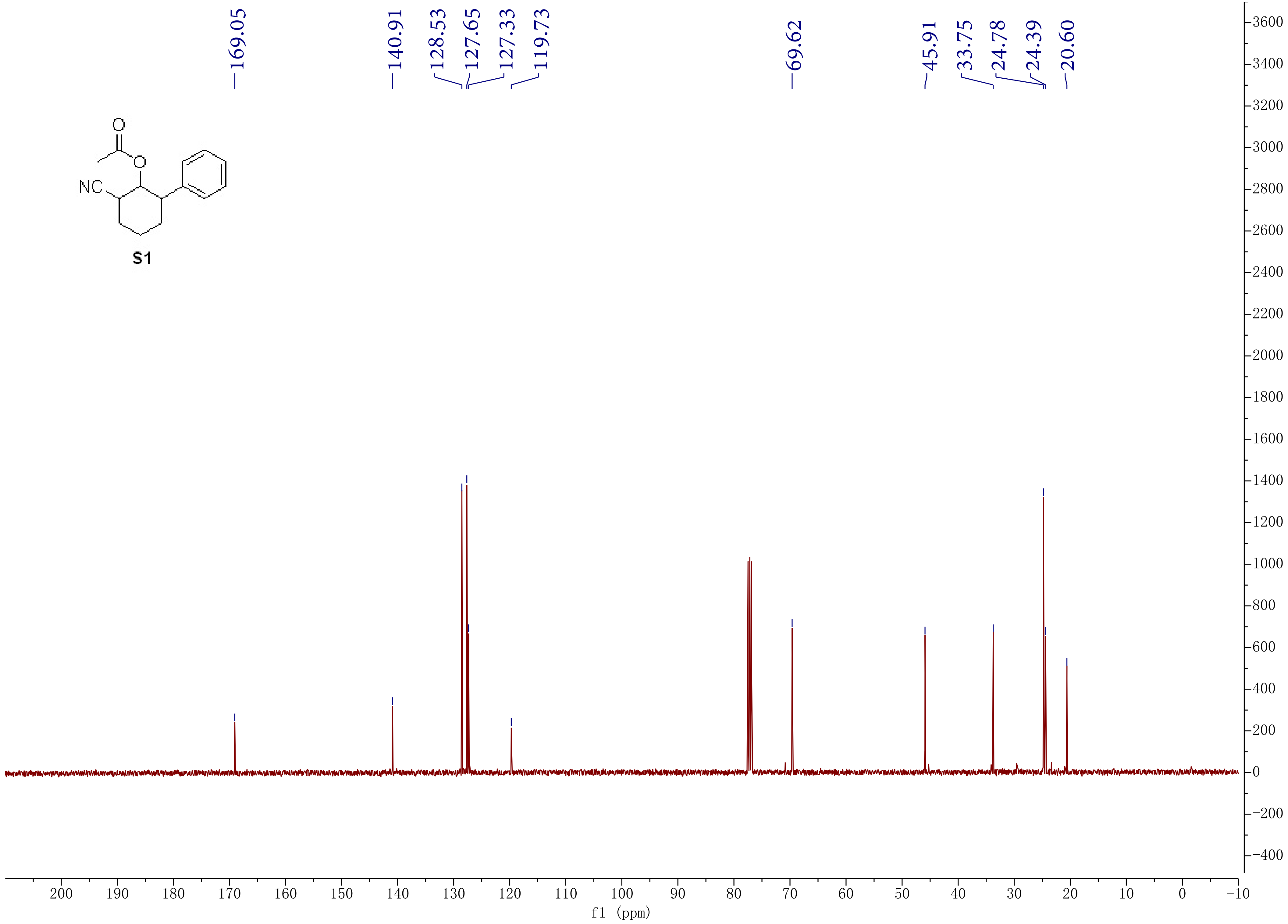
169.05
140.91
128.53
127.65
127.33
119.73
69.62
45.91
33.75
24.78
24.39
20.60
NC
O
S1
f1 (ppm)

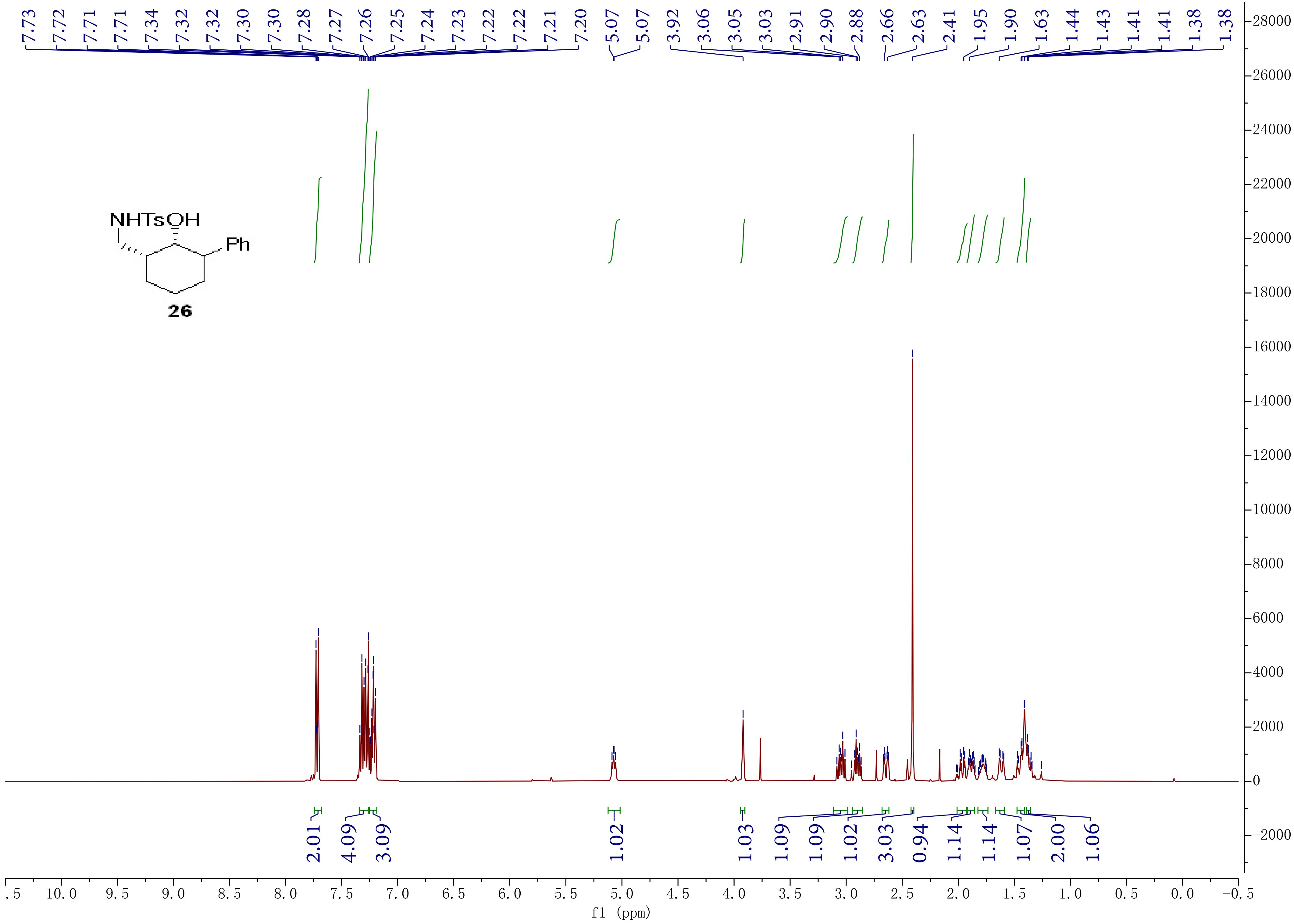
7.73 7.72 7.71 7.71 7.34 7.32 7.32 7.30 7.30 7.28 7.27 7.26 7.25 7.24 7.23 7.22 7.22 7.21 7.20 5.07 5.07 3.92 3.06 3.05 3.03 2.91 2.90 2.88 2.66 2.63 2.41 1.95 1.90 1.63 1.44 1.43 1.41 1.41 1.38 1.38
NHTs OH
Ph
26
2.01 4.09 3.09 1.02 1.03 1.09 1.09 1.02 3.03 0.94 1.14 1.14 1.07 2.00 1.06
f1 (ppm)

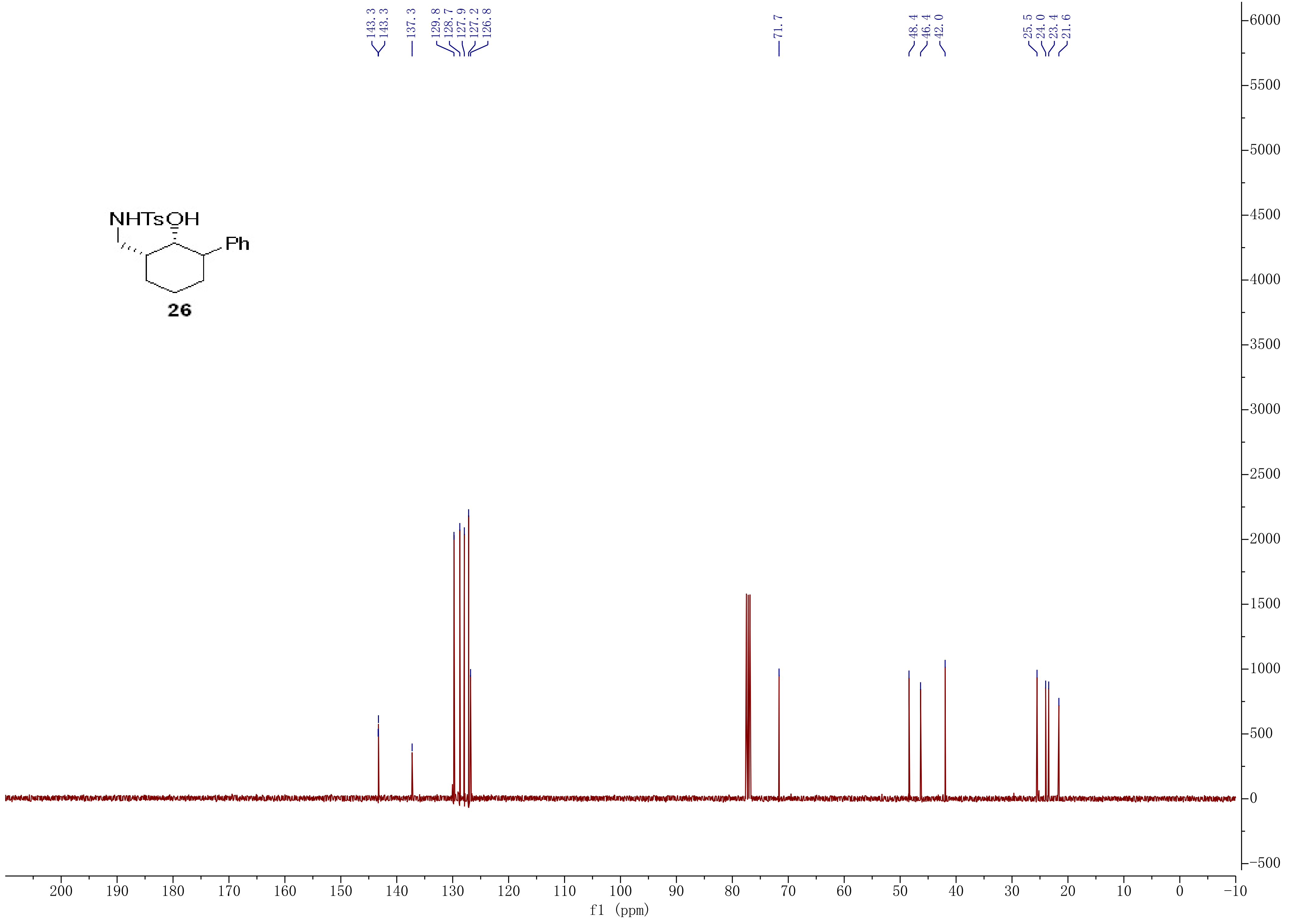
143.3
143.3
137.3
129.8
128.7
127.9
127.2
126.8
71.7
48.4
46.4
42.0
25.5
24.0
23.4
21.6
NHTs
OH
Ph
26
f1 (ppm)

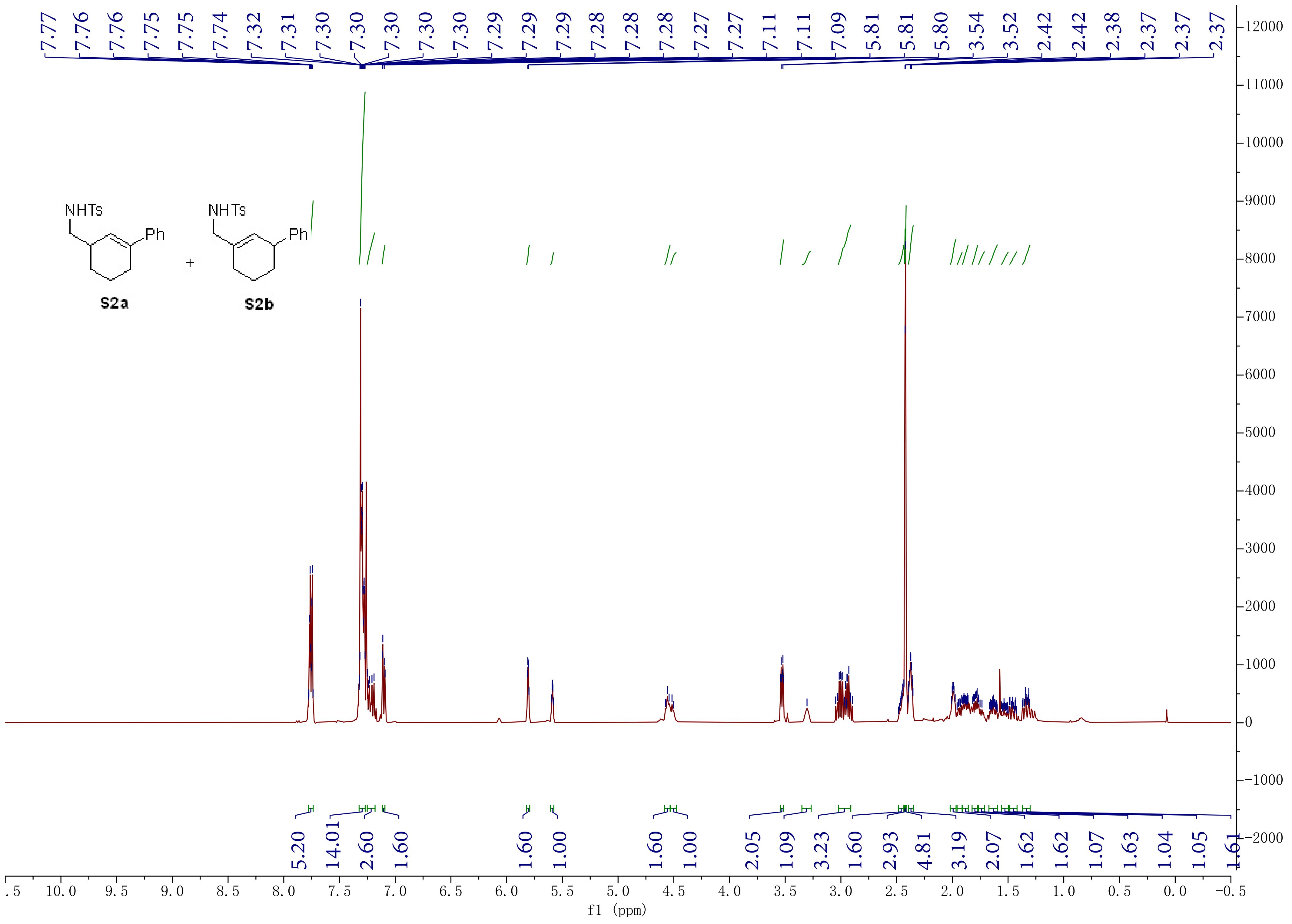
NHTs
Ph
+
NHTs
Ph
S2a
S2b
f1 (ppm)

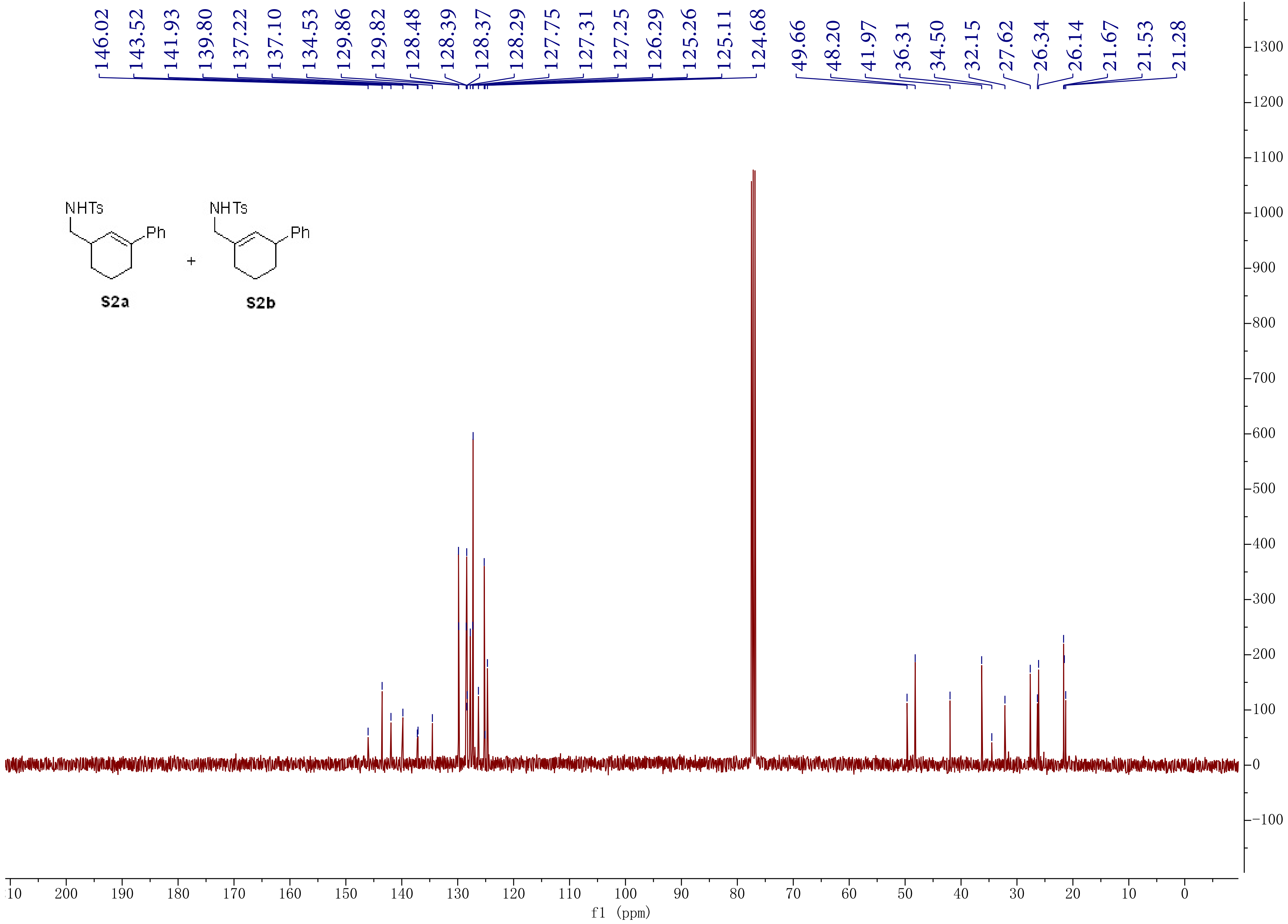
146.02
143.52
141.93
139.80
137.22
137.10
134.53
129.86
129.82
128.48
128.39
128.37
128.29
127.75
127.31
127.25
126.29
125.26
125.11
124.68
49.66
48.20
41.97
36.31
34.50
32.15
27.62
26.34
26.14
21.67
21.53
21.28
NHTs
Ph
+
S2a
S2b
f1 (ppm)

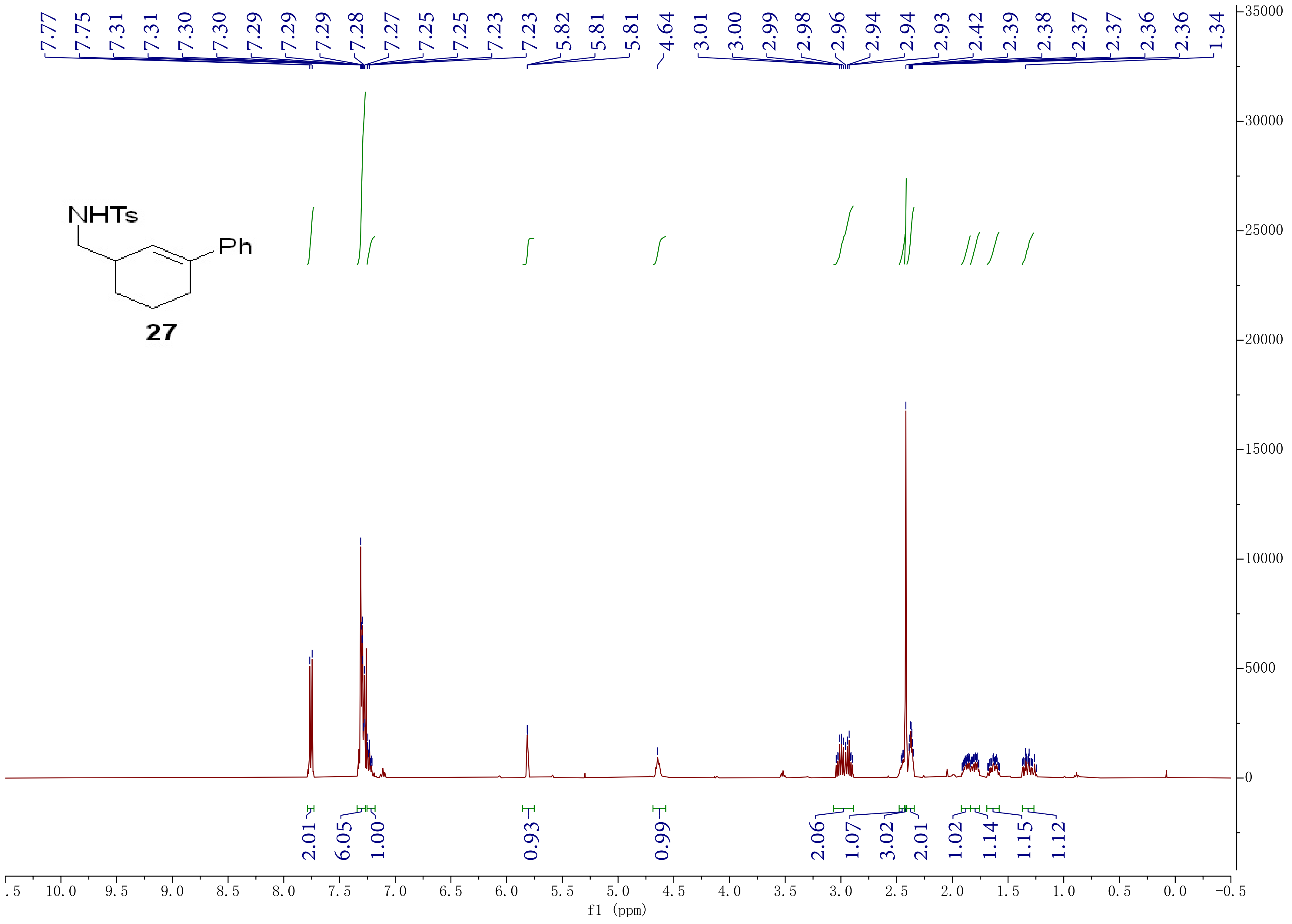
NHTs
Ph
27
7.77
7.75
7.31
7.31
7.30
7.30
7.29
7.29
7.29
7.28
7.27
7.25
7.25
7.23
7.23
5.82
5.81
5.81
4.64
3.01
3.00
2.99
2.98
2.96
2.94
2.94
2.93
2.42
2.39
2.38
2.37
2.37
2.36
2.36
1.34
2.01
6.05
1.00
0.93
0.99
2.06
1.07
3.02
2.01
1.02
1.14
1.15
1.12
f1 (ppm)

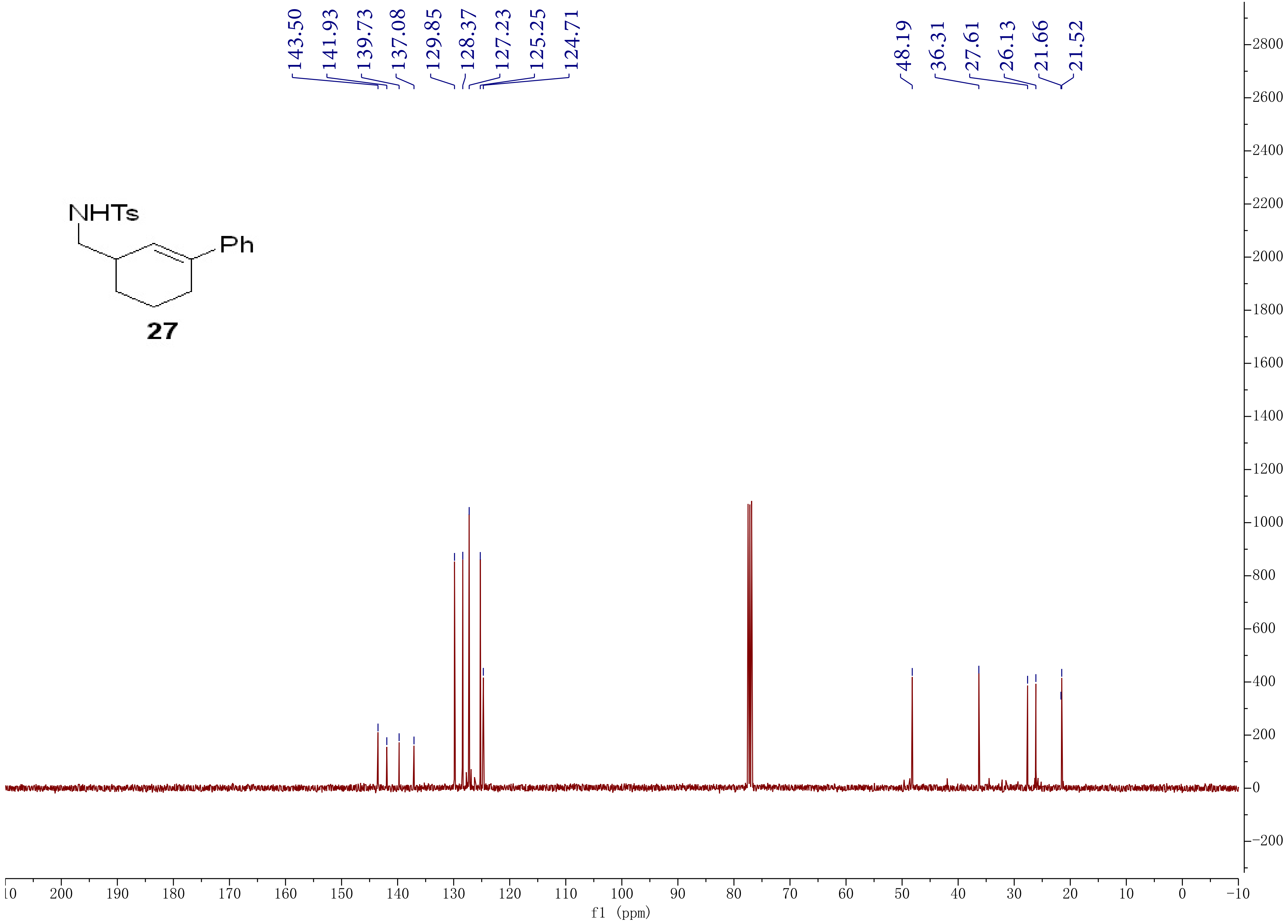
NHTs
Ph
143.50
141.93
139.73
137.08
129.85
128.37
127.23
125.25
124.71
48.19
36.31
27.61
26.13
21.66
21.52
2800
2600
2400
2200
2000
1800
1600
1400
1200
1000
800
600
400
200
0
−200
200
190
180
170
160
150
140
130
120
110
100
90
80
70
60
50
40
30
20
10
0
−10
f1 (ppm)

27

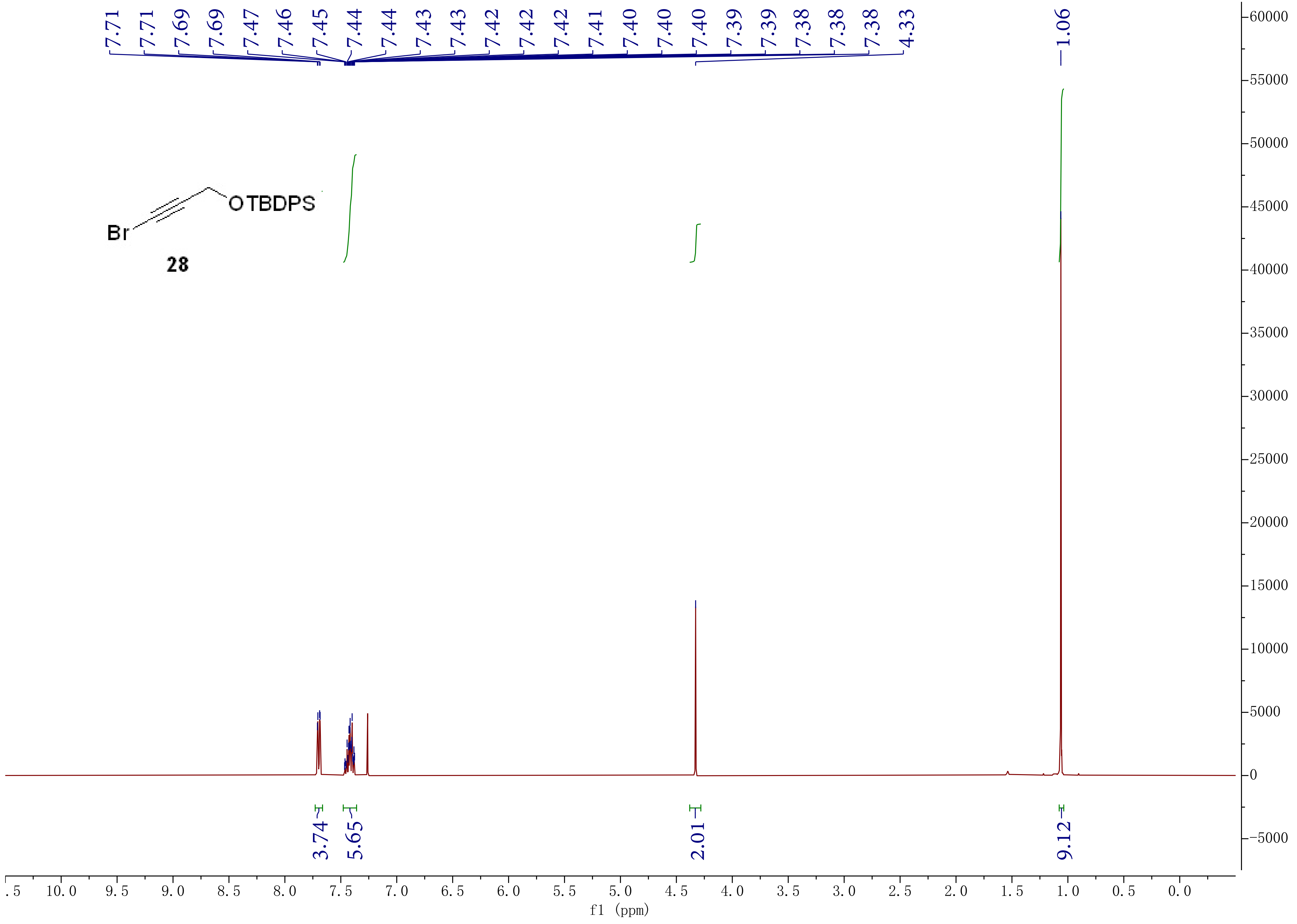

7.71 7.71 7.69 7.69 7.47 7.46 7.45 7.44 7.44 7.43 7.43 7.42 7.42 7.42 7.41 7.40 7.40 7.40 7.39 7.39 7.38 7.38 7.38 4.33
1.06
Br
OTBDPS
28
3.74
5.65
2.01
9.12
f1 (ppm)

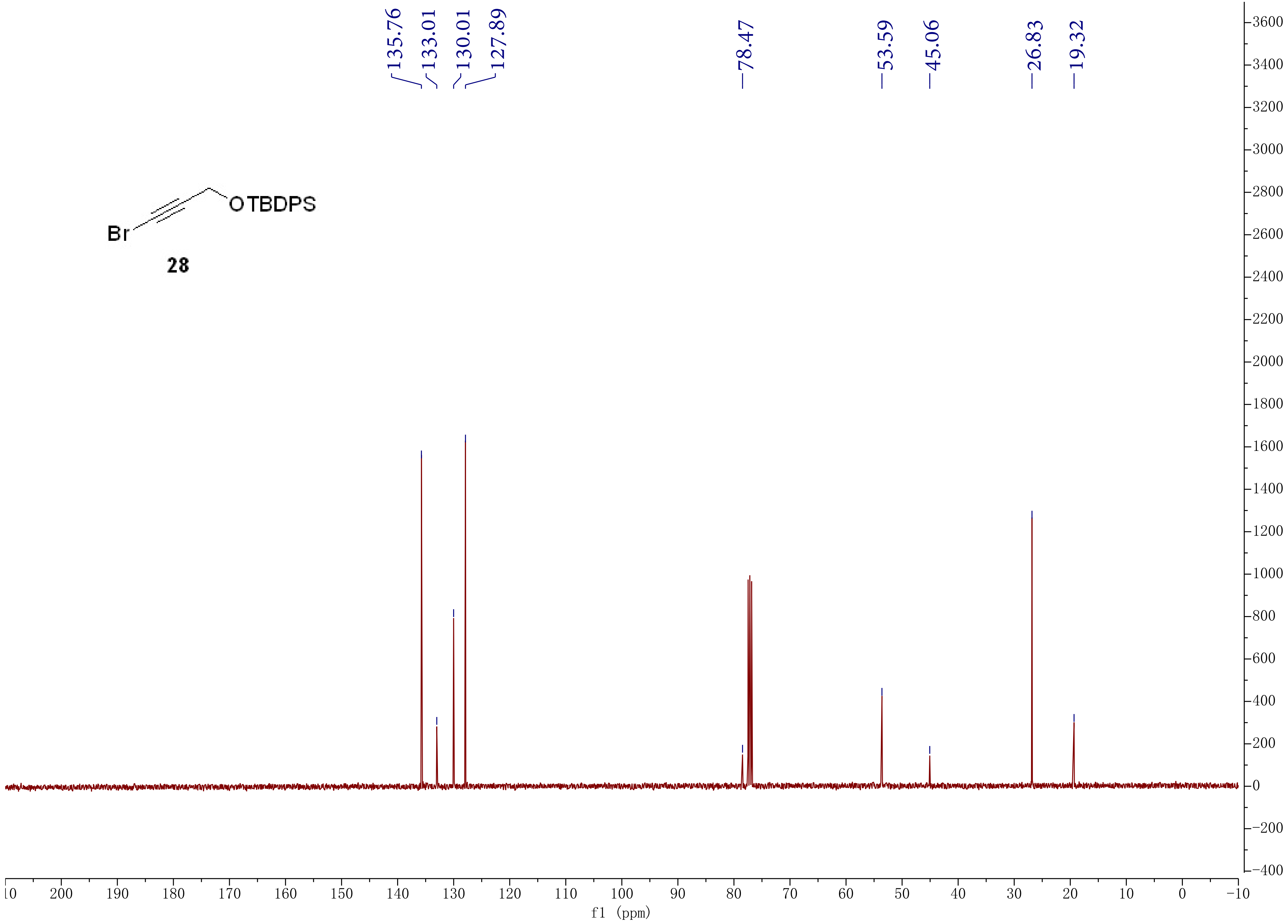
135.76
133.01
130.01
127.89
78.47
53.59
45.06
26.83
19.32
Br
OTBDPS
28
f1 (ppm)

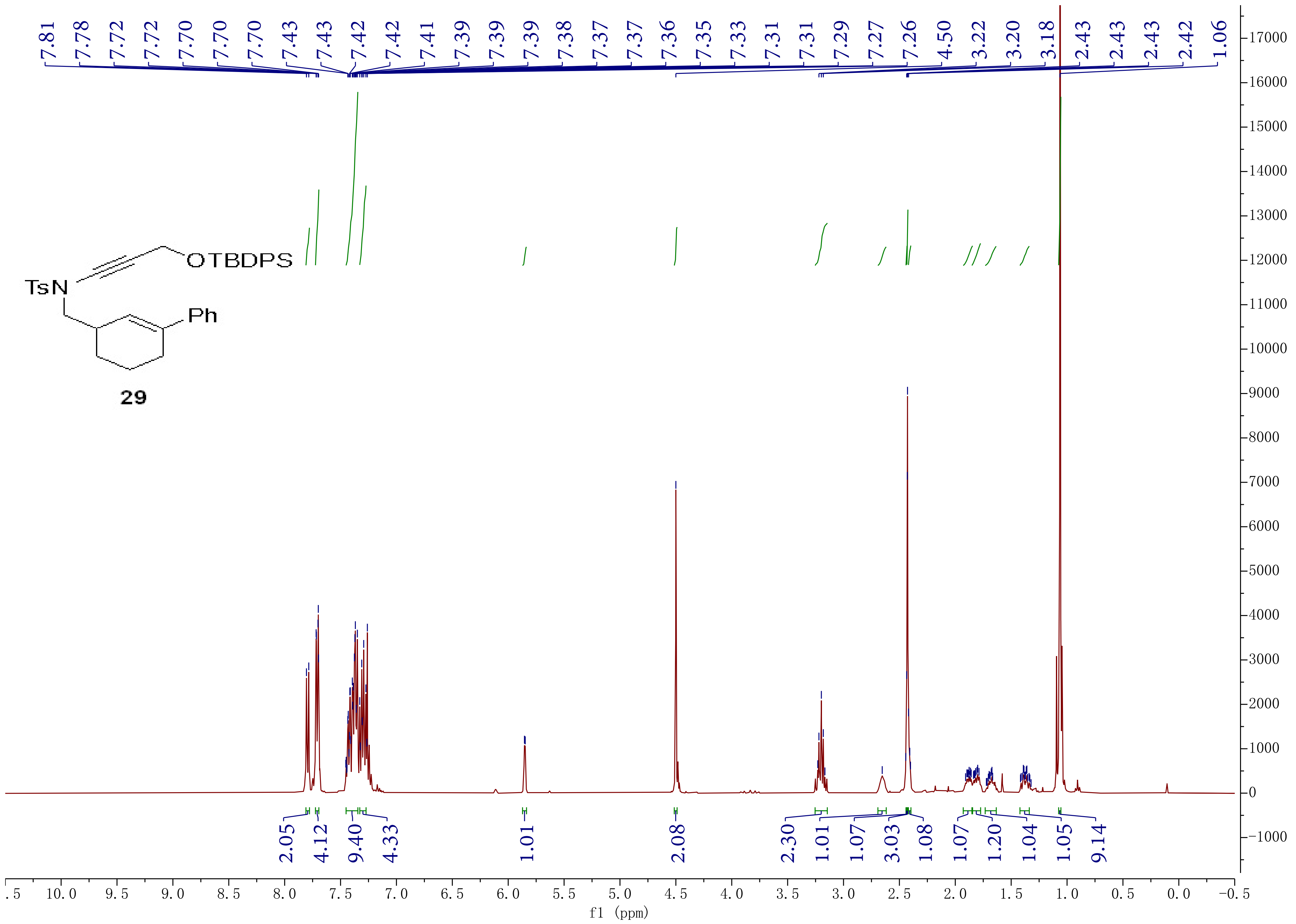
7.81 7.78 7.72 7.72 7.70 7.70 7.70 7.43 7.43 7.42 7.42 7.41 7.39 7.39 7.39 7.38 7.37 7.37 7.36 7.35 7.33 7.31 7.31 7.29 7.27 7.26 4.50 3.22 3.20 3.18 2.43 2.43 2.43 2.42 1.06
OTBDPS
TsN
Ph
29
2.05 4.12 9.40 4.33 1.01 2.08 2.30 1.01 1.07 3.03 1.08 1.07 1.20 1.04 1.05 9.14
17000 16000 15000 14000 13000 12000 11000 10000 9000 8000 7000 6000 5000 4000 3000 2000 1000 0 −1000
.5 10.0 9.5 9.0 8.5 8.0 7.5 7.0 6.5 6.0 5.5 5.0 4.5 4.0 3.5 3.0 2.5 2.0 1.5 1.0 0.5 0.0 −0.5
f1 (ppm)

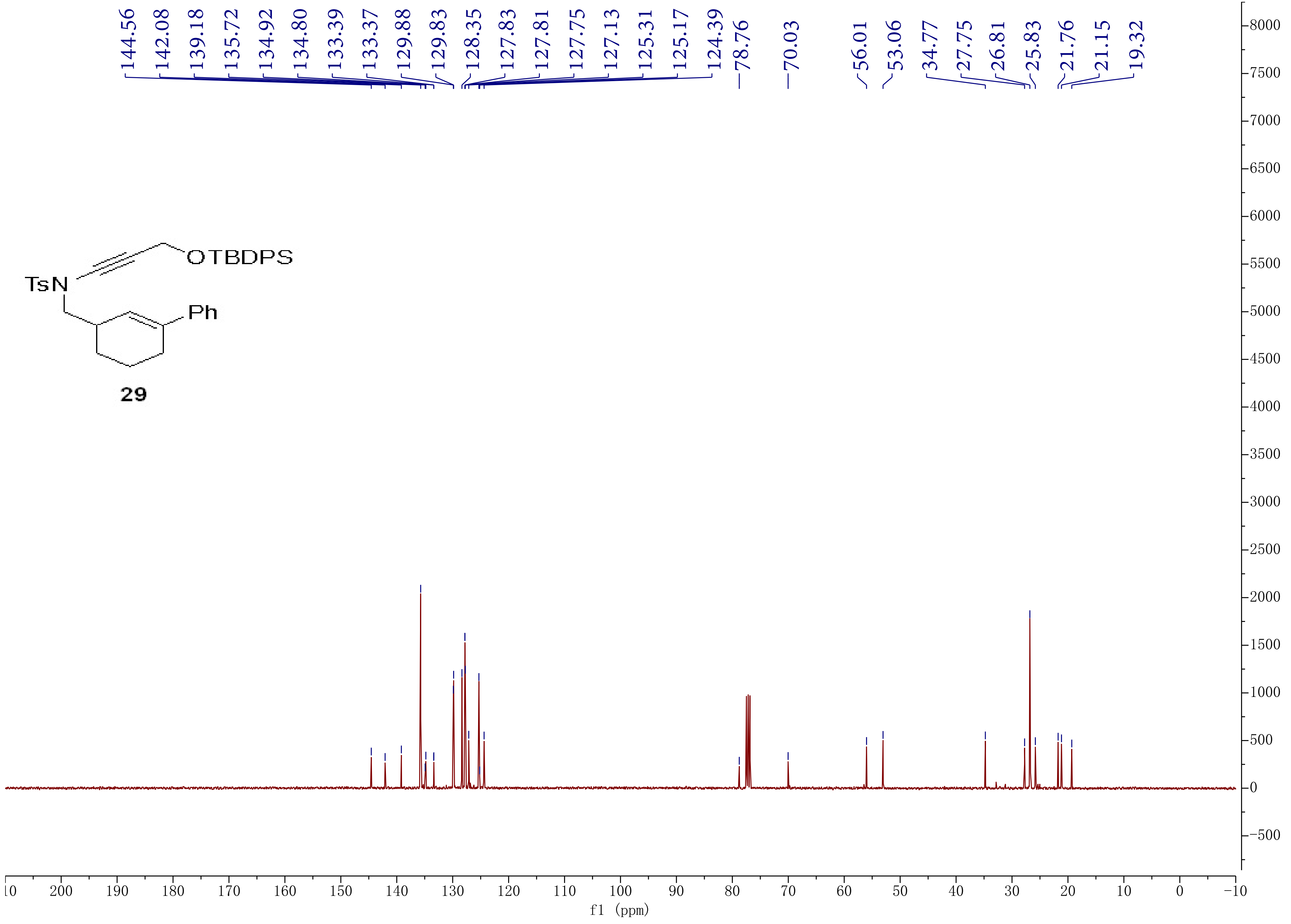
144.56
142.08
139.18
135.72
134.92
134.80
133.39
133.37
129.88
129.83
128.35
127.83
127.81
127.75
127.13
125.31
125.17
124.39
78.76
70.03
56.01
53.06
34.77
27.75
26.81
25.83
21.76
21.15
19.32
OTBDPS
TsN
Ph
29
f1 (ppm)

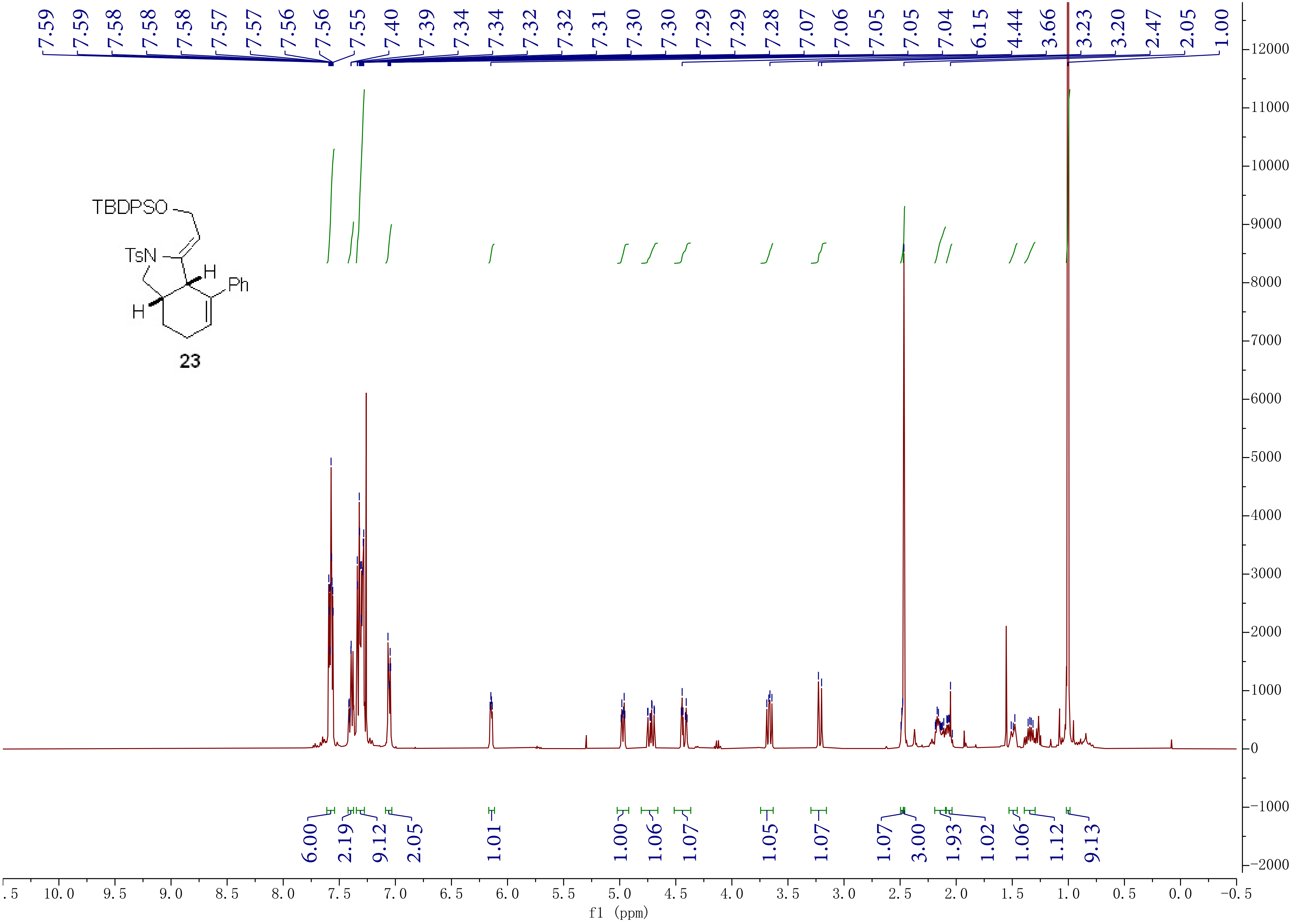

7.59
7.59
7.58
7.58
7.58
7.57
7.57
7.56
7.56
7.55
7.40
7.39
7.34
7.34
7.32
7.32
7.31
7.30
7.30
7.29
7.29
7.28
7.07
7.06
7.05
7.05
7.04
6.15
4.44
3.66
3.23
3.20
2.47
2.05
1.00
TBDPSO
TsN
H
Ph
H
23
6.00
2.19
9.12
2.05
1.01
1.00
1.06
1.07
1.05
1.07
1.07
3.00
1.93
1.02
1.06
1.12
9.13
f1 (ppm)

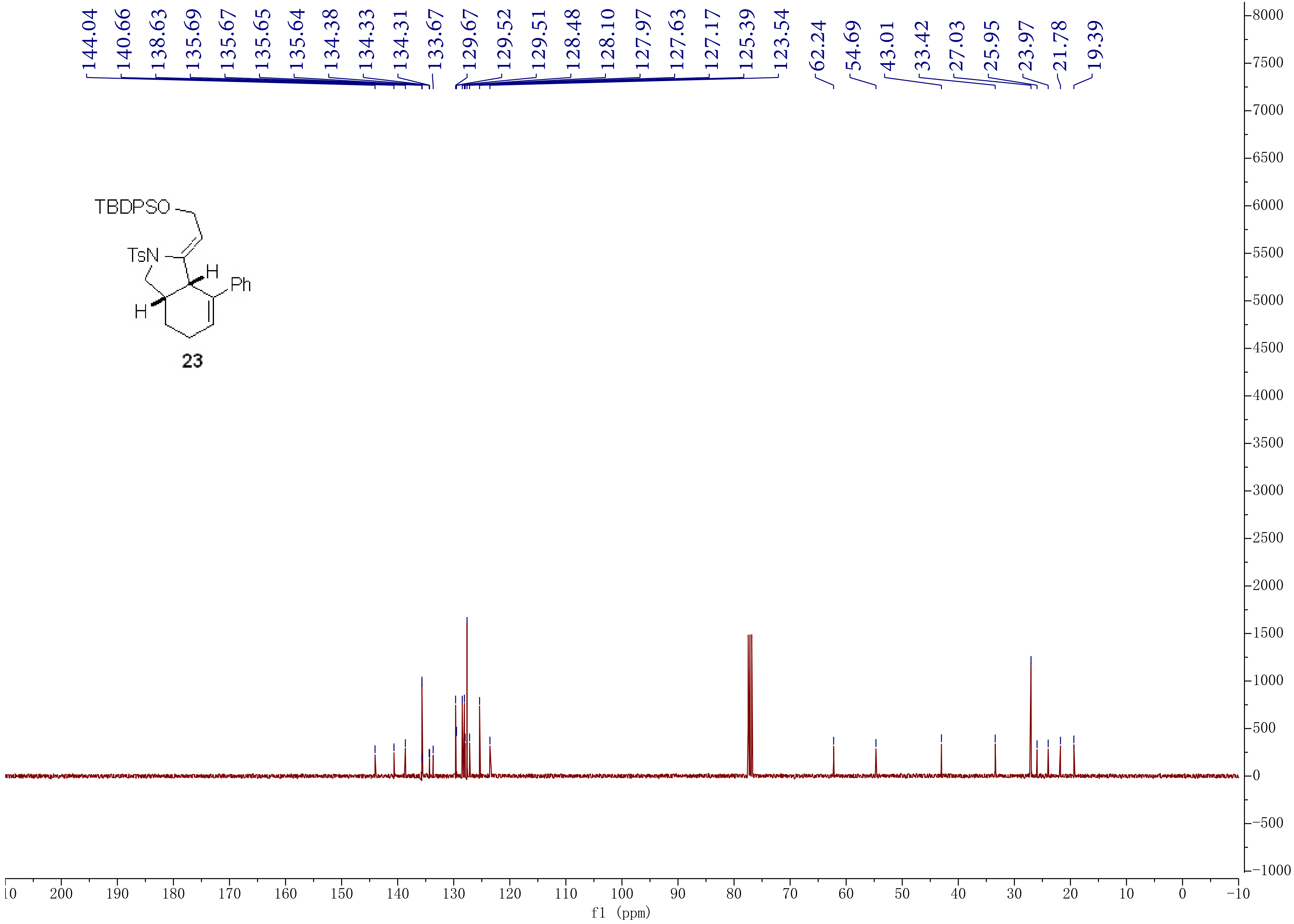

144.04
140.66
138.63
135.69
135.67
135.65
135.64
134.38
134.33
134.31
133.67
129.67
129.52
129.51
128.48
128.10
127.97
127.63
127.17
125.39
123.54
62.24
54.69
43.01
33.42
27.03
25.95
23.97
21.78
19.39
TBDPSO
TsN
H
Ph
H
23
f1 (ppm)

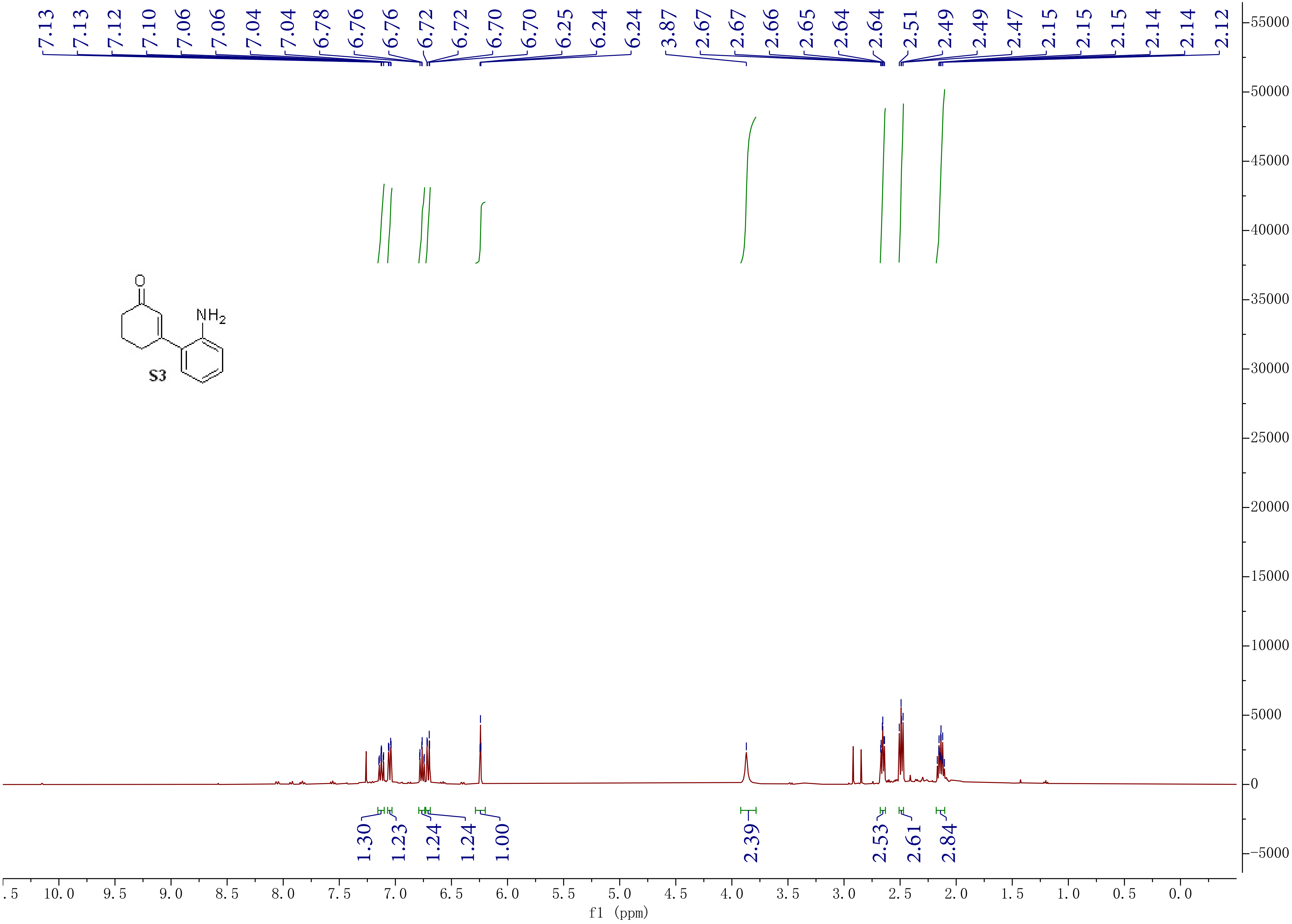
S3

7.13
7.13
7.12
7.10
7.06
7.06
7.04
7.04
6.78
6.76
6.76
6.72
6.72
6.70
6.70
6.25
6.24
6.24
3.87
2.67
2.67
2.66
2.65
2.64
2.64
2.51
2.49
2.49
2.47
2.15
2.15
2.15
2.14
2.14
2.12
1.30
1.23
1.24
1.24
1.00
2.39
2.53
2.61
2.84
f1 (ppm)

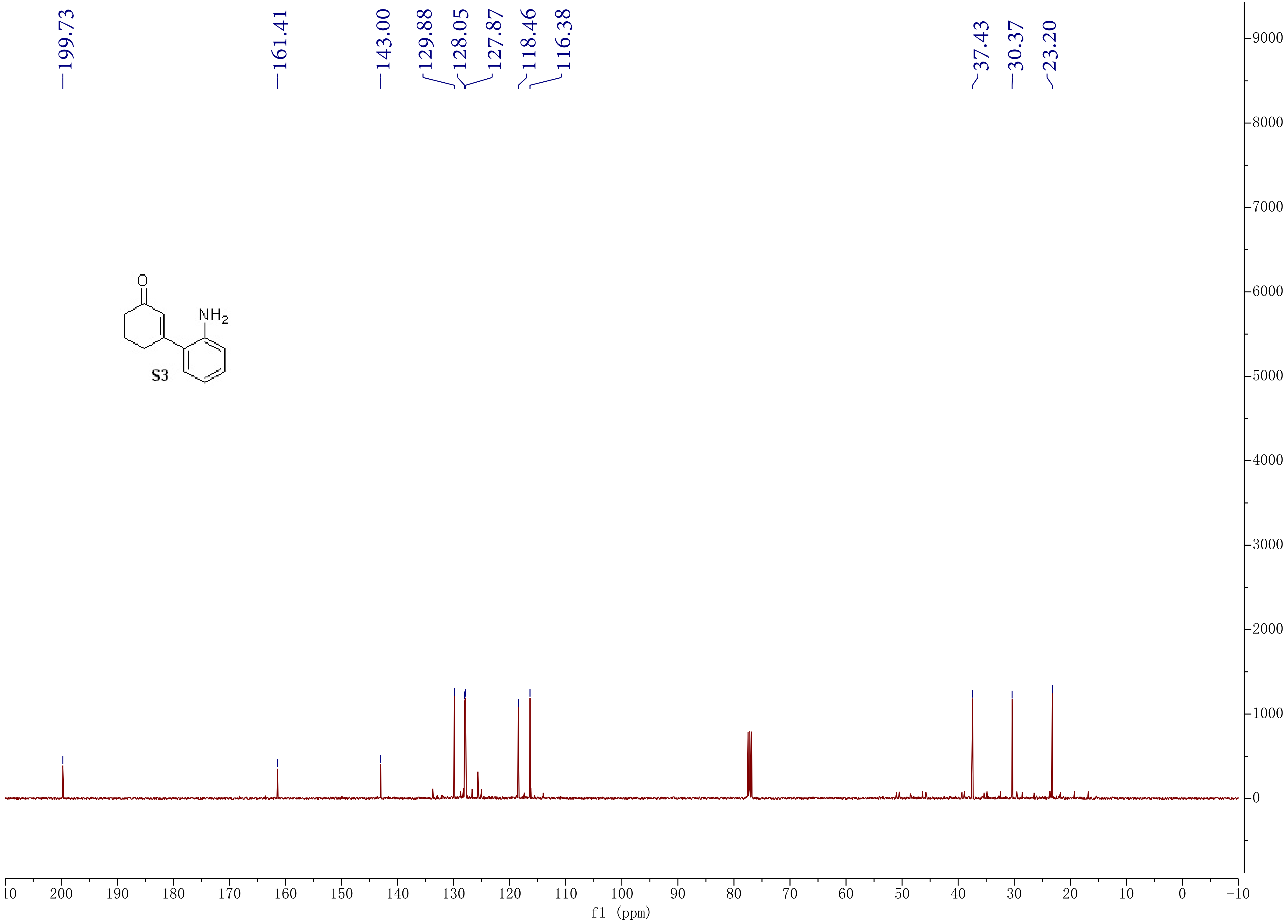

199.73
161.41
143.00
129.88
128.05
127.87
118.46
116.38
37.43
30.37
23.20
O
NH2
S3
9000
8000
7000
6000
5000
4000
3000
2000
1000
0
210
200
190
180
170
160
150
140
130
120
110
100
90
80
70
60
50
40
30
20
10
0
−10
f1 (ppm)

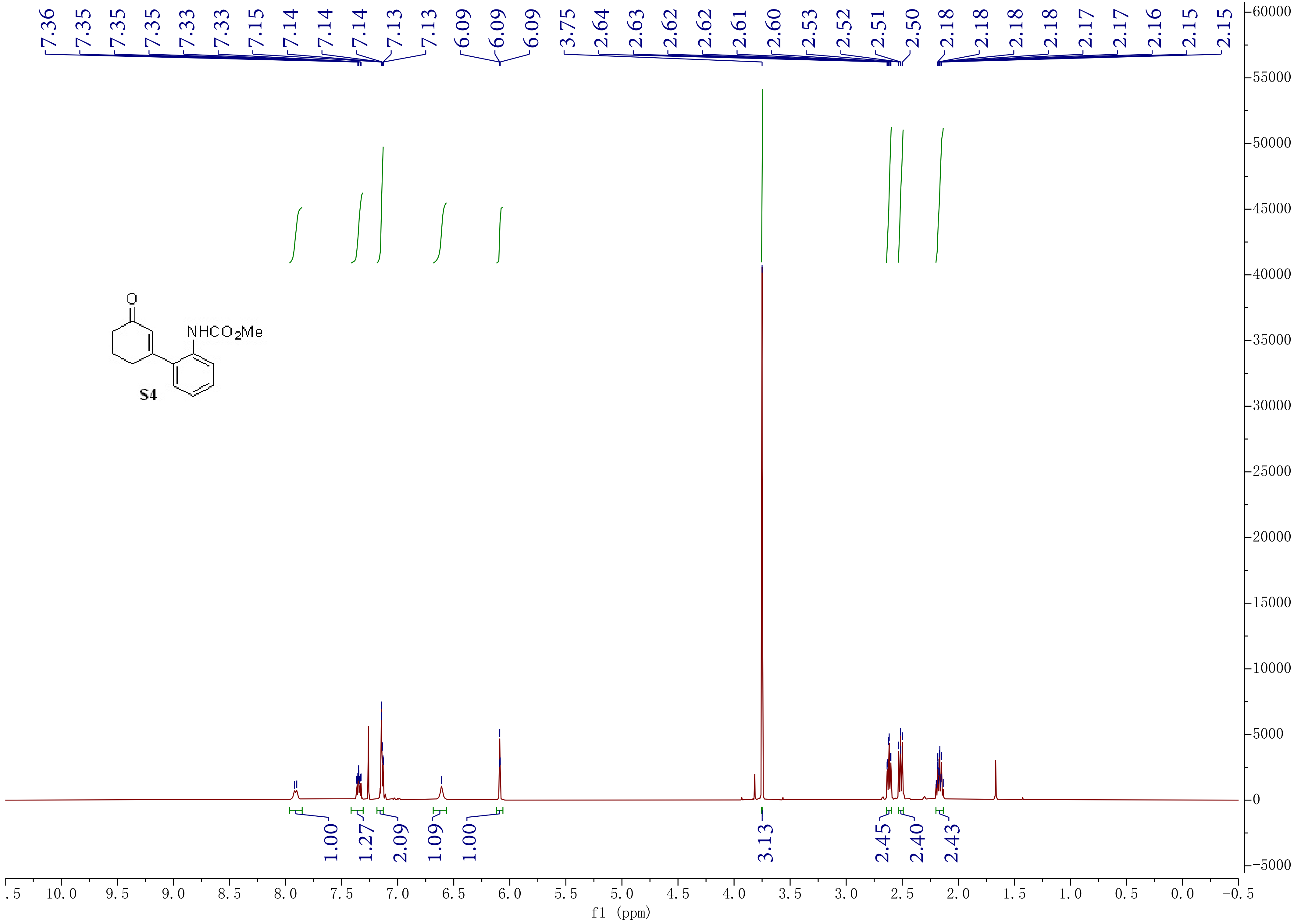
7.36 7.35 7.35 7.35 7.33 7.33 7.15 7.14 7.14 7.14 7.13 7.13 6.09 6.09 6.09 3.75 2.64 2.63 2.62 2.62 2.61 2.60 2.53 2.52 2.51 2.50 2.18 2.18 2.18 2.18 2.17 2.17 2.16 2.15 2.15
NHCO2Me
O
S4
1.00 1.27 2.09 1.09 1.00 3.13 2.45 2.40 2.43
60000 55000 50000 45000 40000 35000 30000 25000 20000 15000 10000 5000 0 −5000
.5 10.0 9.5 9.0 8.5 8.0 7.5 7.0 6.5 6.0 5.5 5.0 4.5 4.0 3.5 3.0 2.5 2.0 1.5 1.0 0.5 0.0 −0.5
f1 (ppm)

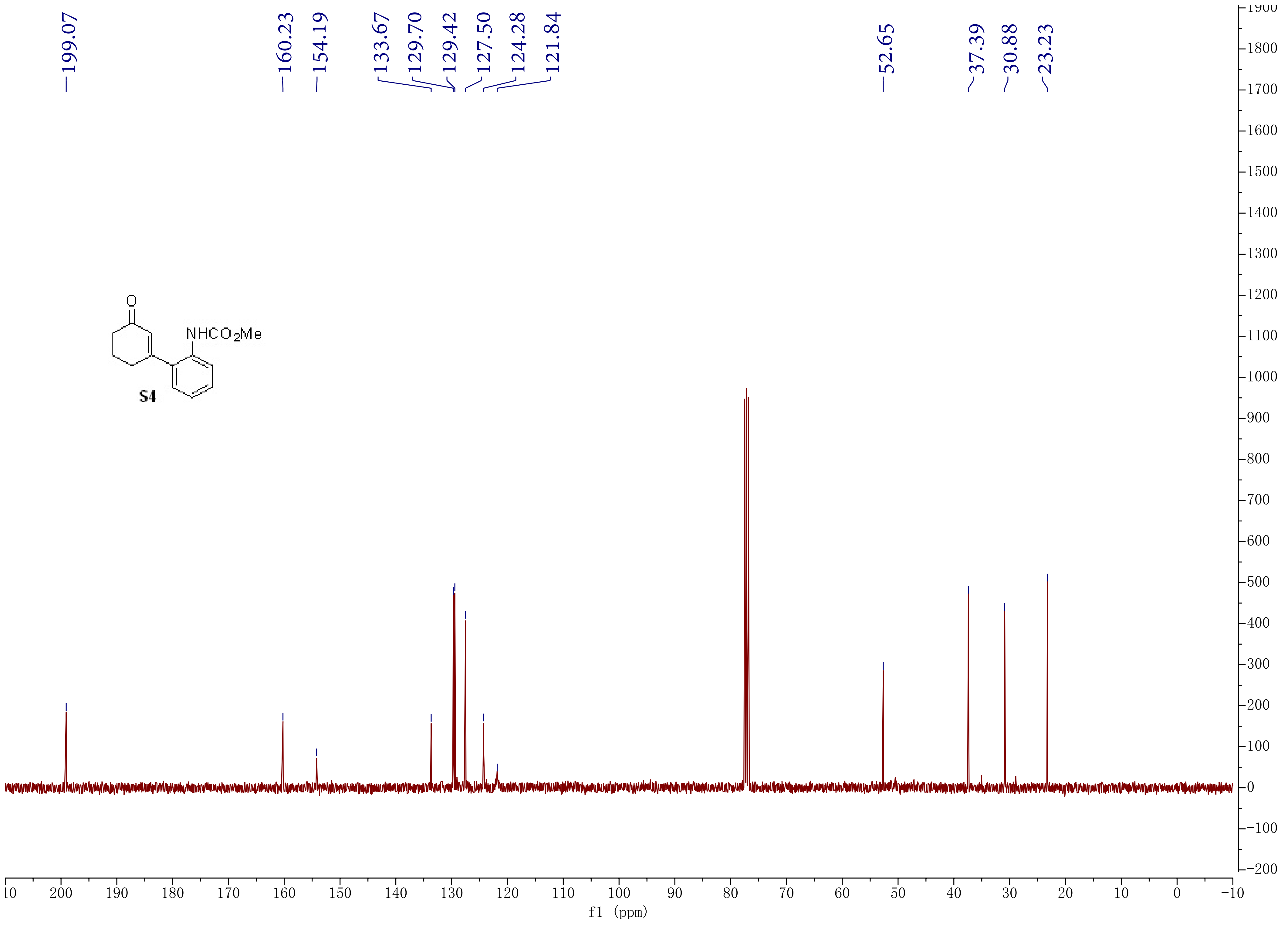
199.07
160.23
154.19
133.67
129.70
129.42
127.50
124.28
121.84
52.65
37.39
30.88
23.23
O
NHCO2Me
S4
f1 (ppm)

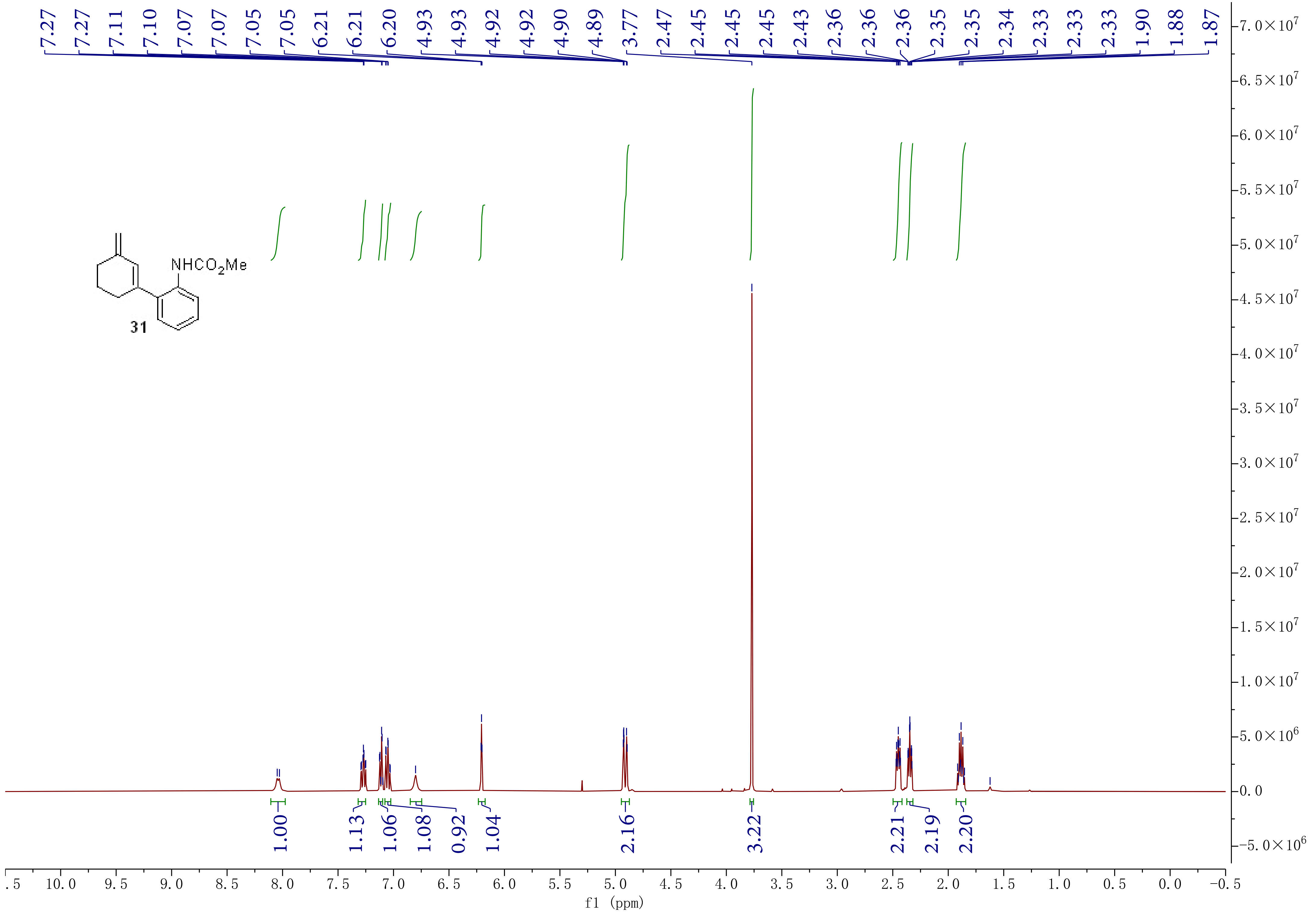

7.27 7.27 7.11 7.10 7.07 7.07 7.05 7.05 6.21 6.21 6.20 4.93 4.93 4.92 4.92 4.90 4.89 3.77 2.47 2.45 2.45 2.45 2.43 2.36 2.36 2.36 2.35 2.35 2.34 2.33 2.33 2.33 1.90 1.88 1.87
NHCO2Me
31
1.00 1.13 1.06 1.08 0.92 1.04 2.16 3.22 2.21 2.19 2.20
f1 (ppm)

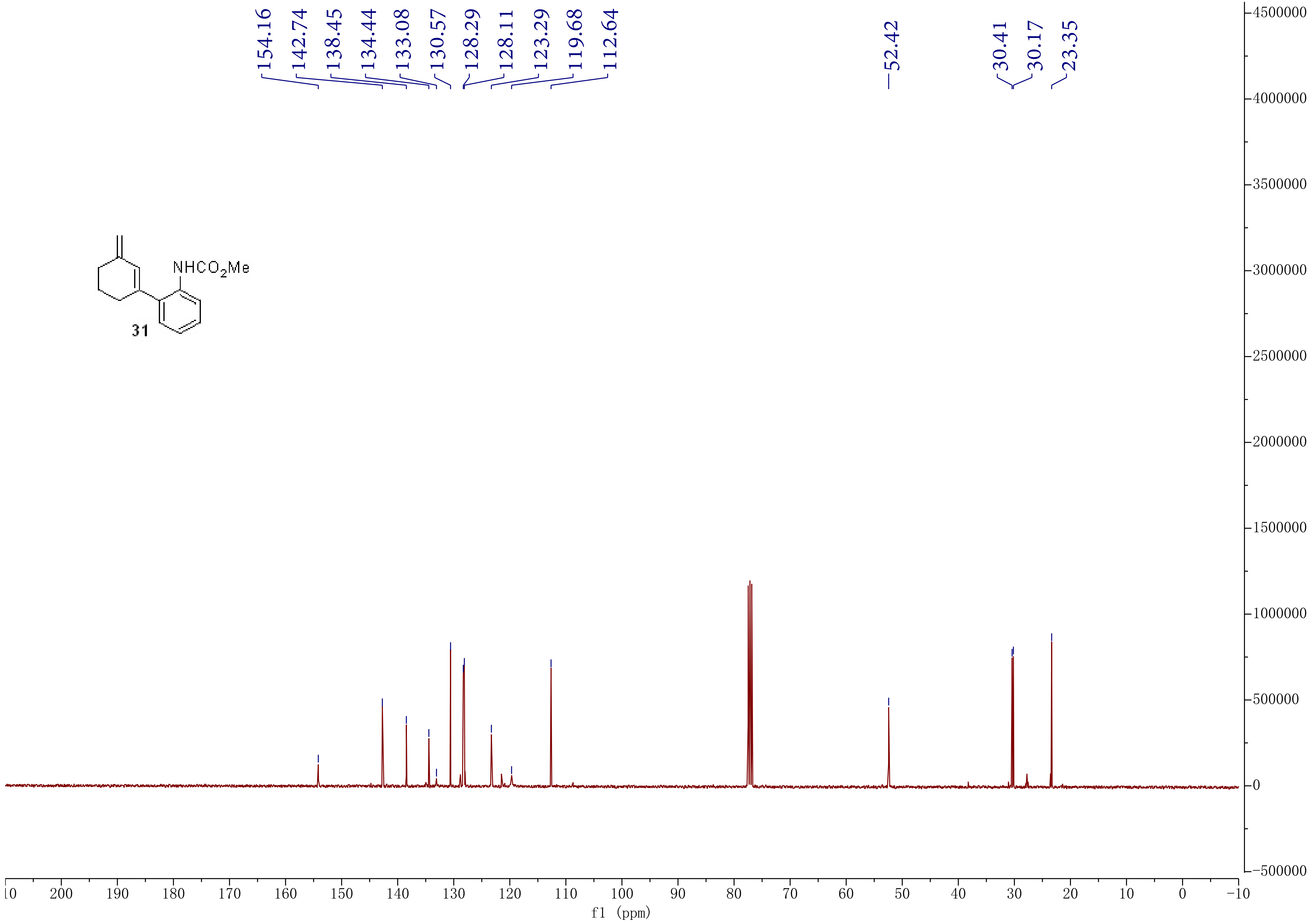

154.16
142.74
138.45
134.44
133.08
130.57
128.29
128.11
123.29
119.68
112.64
52.42
30.41
30.17
23.35
NHCO2Me
31
4500000
4000000
3500000
3000000
2500000
2000000
1500000
1000000
500000
0
−500000
200
190
180
170
160
150
140
130
120
110
100
90
80
70
60
50
40
30
20
10
0
−10
f1 (ppm)

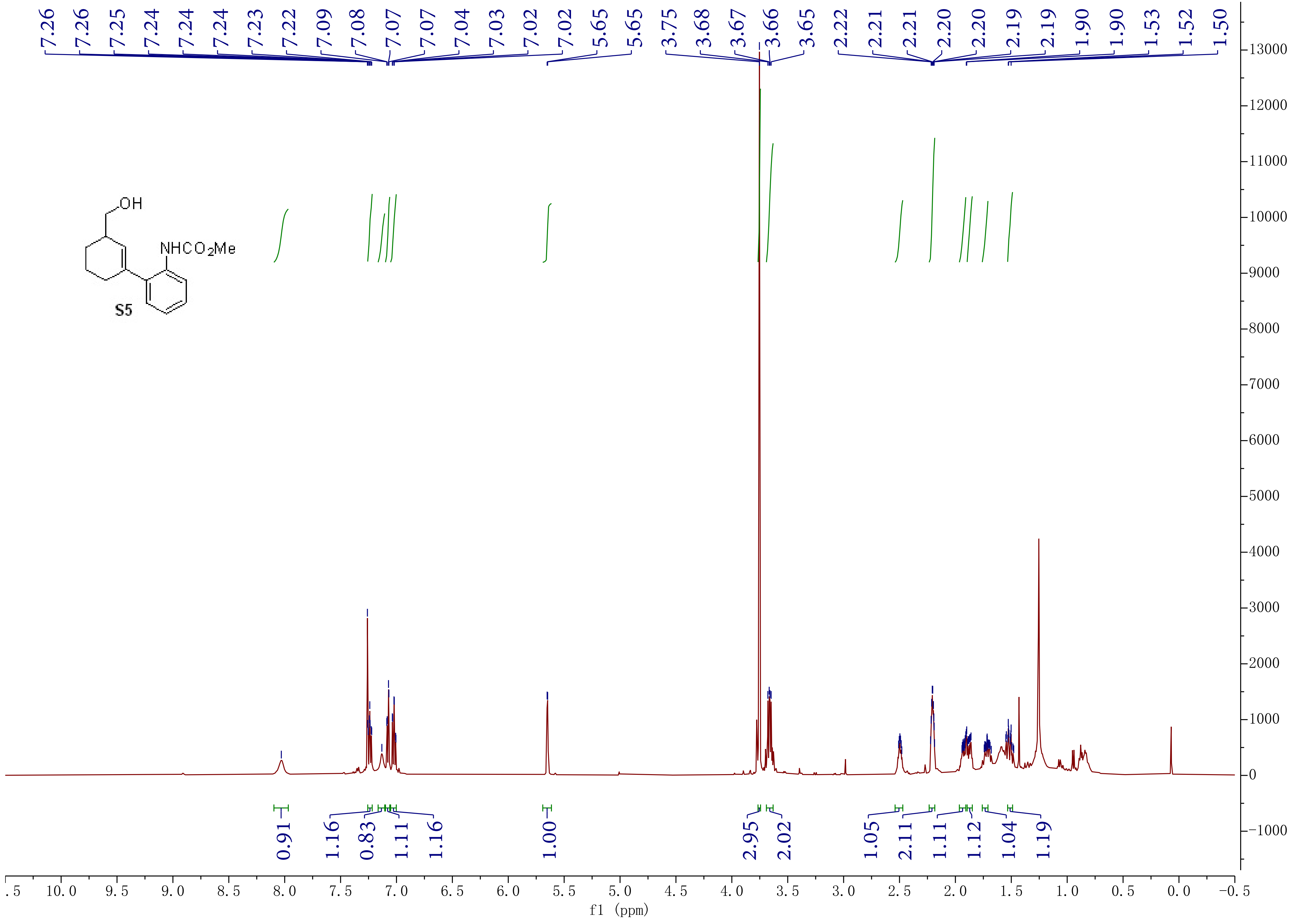

7.26 7.26 7.25 7.24 7.24 7.24 7.23 7.22 7.09 7.08 7.07 7.07 7.04 7.03 7.02 7.02 5.65 5.65 3.75 3.68 3.67 3.66 3.65 2.22 2.21 2.21 2.20 2.20 2.19 2.19 1.90 1.90 1.53 1.52 1.50
OH
NHCO2Me
S5
0.91 1.16 0.83 1.11 1.16 1.00 2.95 2.02 1.05 2.11 1.11 1.12 1.04 1.19
f1 (ppm)

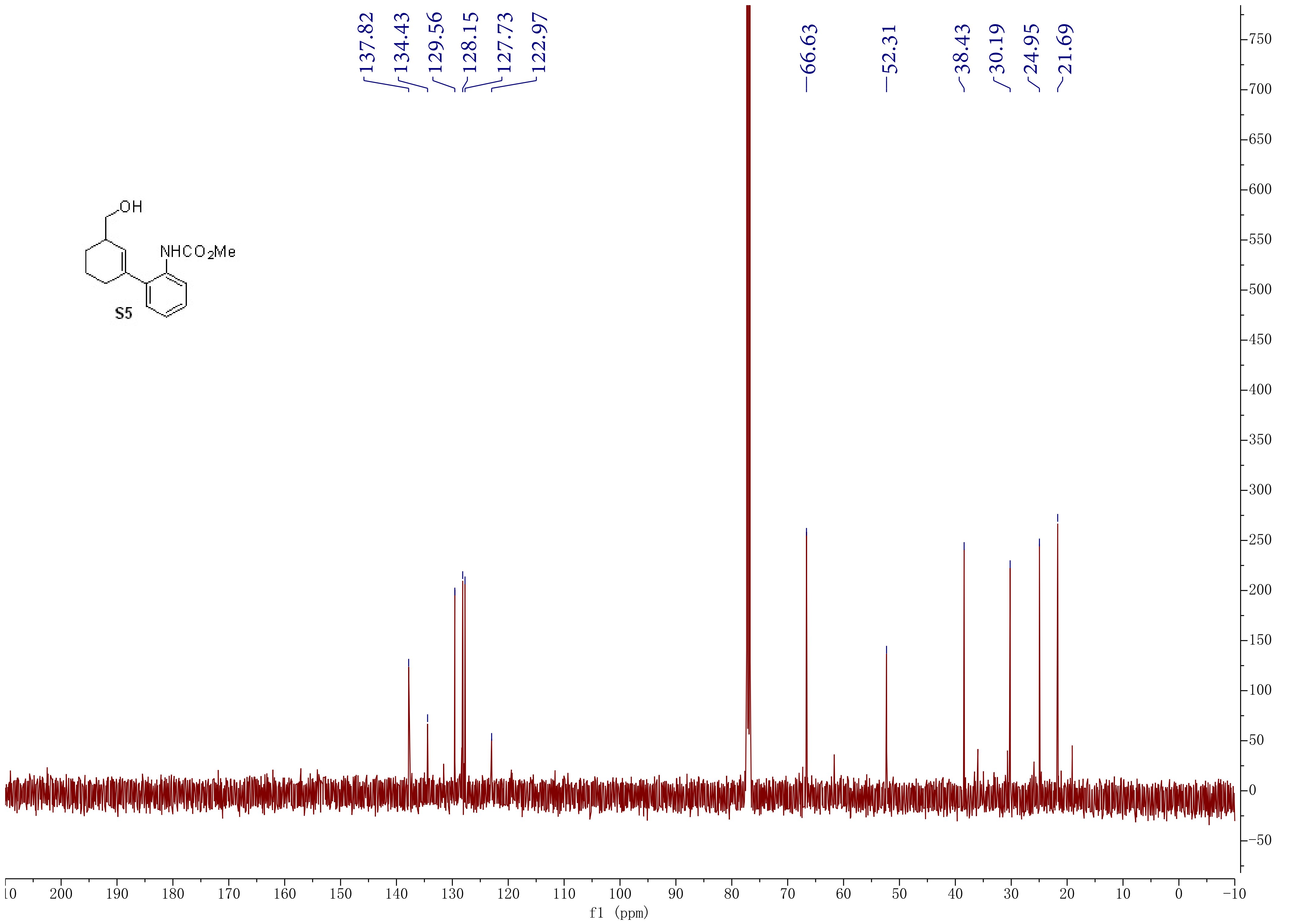

137.82
134.43
129.56
128.15
127.73
122.97
66.63
52.31
38.43
30.19
24.95
21.69
OH
NHCO2Me
S5
f1 (ppm)

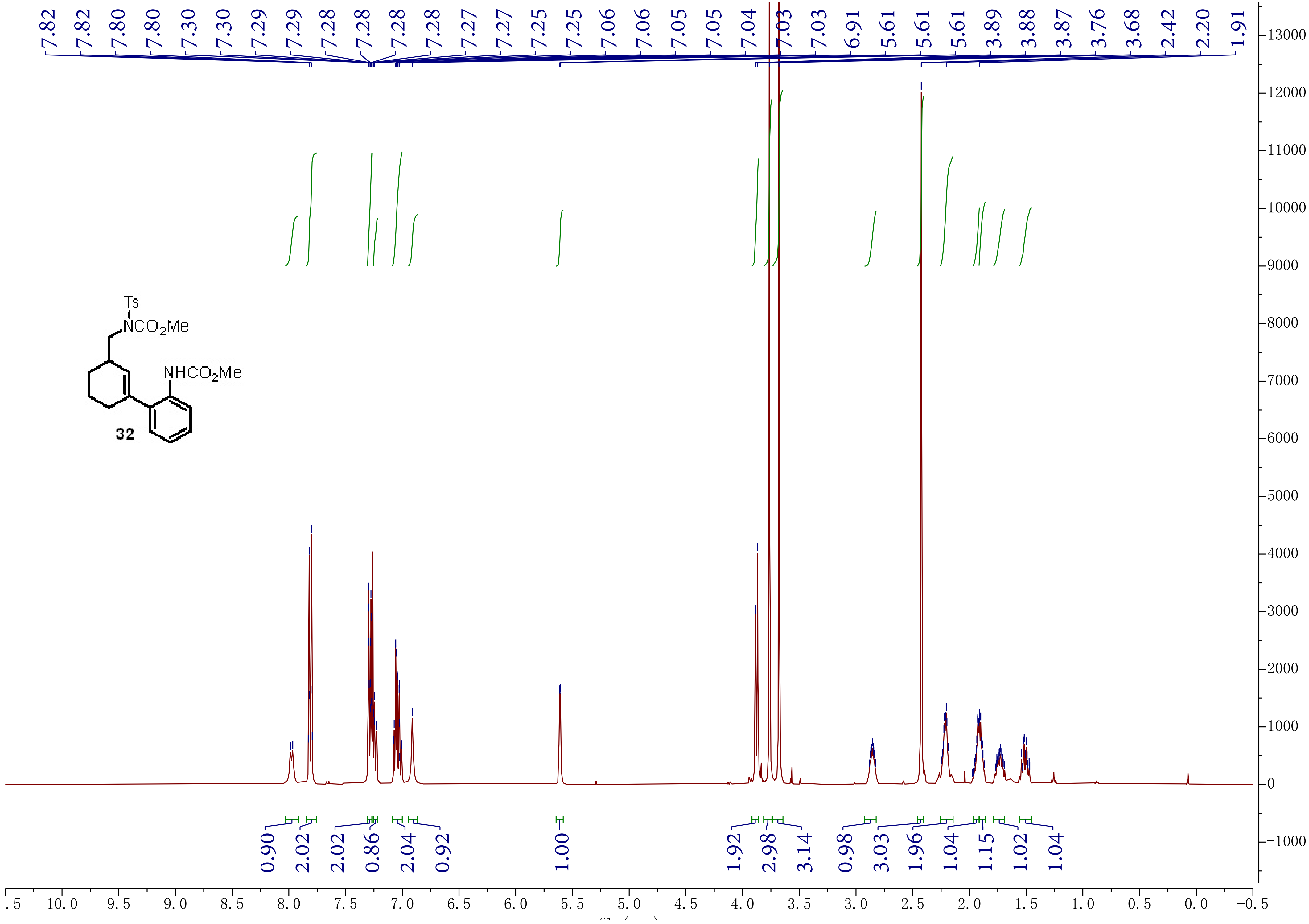
7.82
7.82
7.80
7.80
7.30
7.30
7.29
7.29
7.28
7.28
7.28
7.28
7.27
7.27
7.25
7.25
7.06
7.06
7.05
7.05
7.04
7.03
7.03
6.91
5.61
5.61
5.61
3.89
3.88
3.87
3.76
3.68
2.42
2.20
1.91
Ts
NCO2Me
NHCO2Me
32
0.90
2.02
2.02
0.86
2.04
0.92
1.00
1.92
2.98
3.14
0.98
3.03
1.96
1.04
1.15
1.02
1.04
13000
12000
11000
10000
9000
8000
7000
6000
5000
4000
3000
2000
1000
0
-1000
.5
10.0
9.5
9.0
8.5
8.0
7.5
7.0
6.5
6.0
5.5
5.0
4.5
4.0
3.5
3.0
2.5
2.0
1.5
1.0
0.5
0.0
-0.5

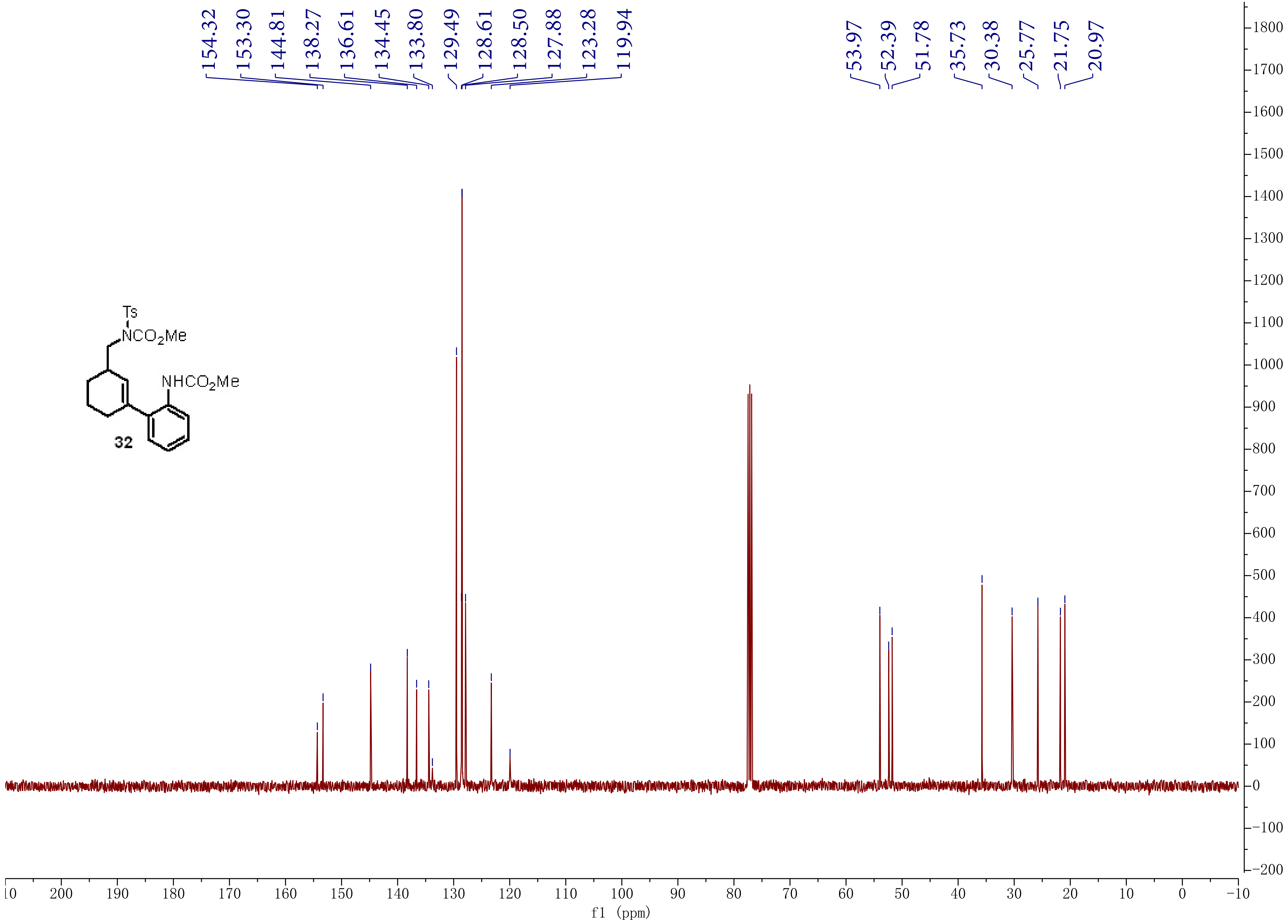
154.32
153.30
144.81
138.27
136.61
134.45
133.80
129.49
128.61
128.50
127.88
123.28
119.94
53.97
52.39
51.78
35.73
30.38
25.77
21.75
20.97
Ts
NCO2Me
NHCO2Me
32
f1 (ppm)

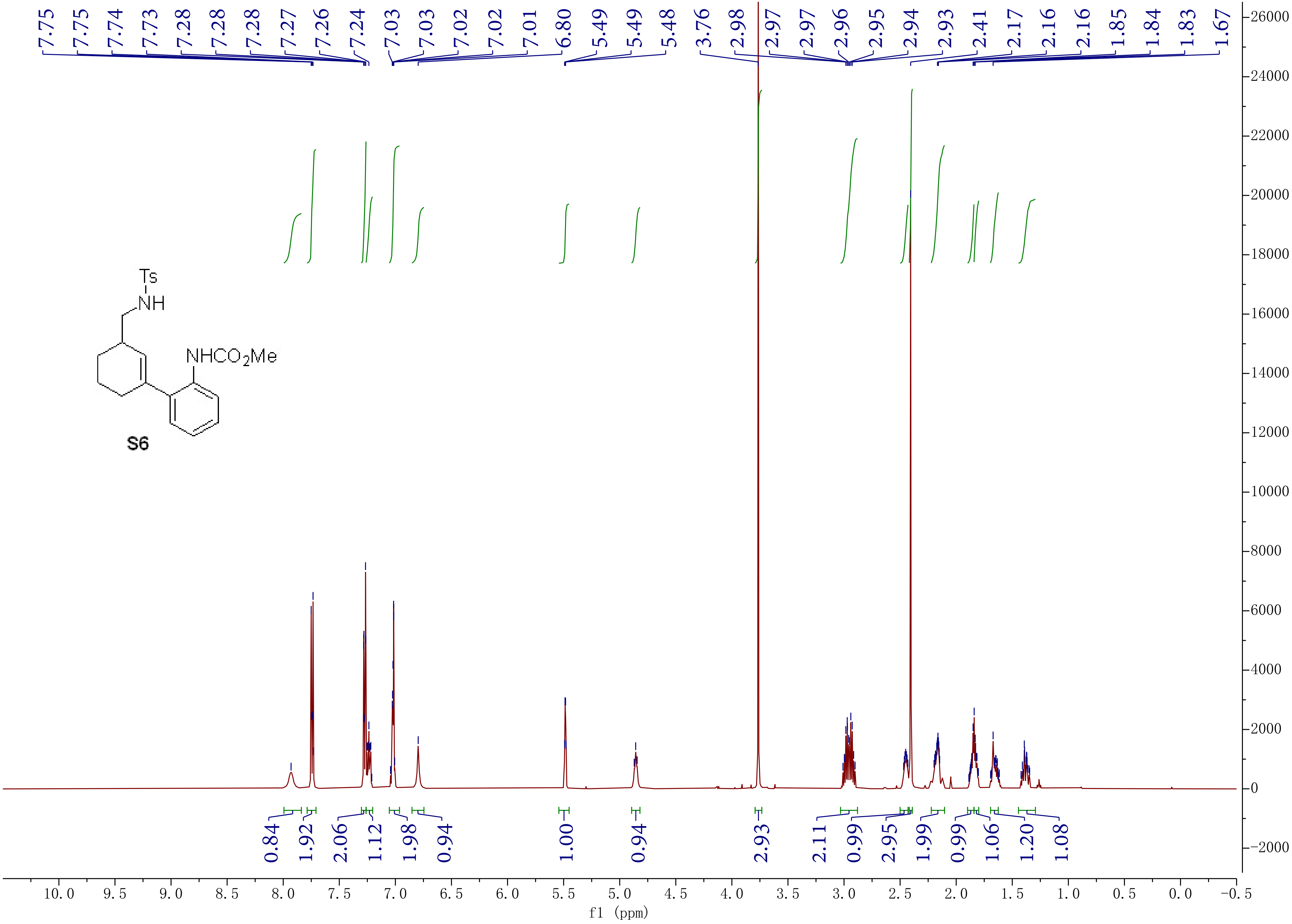
7.75
7.75
7.74
7.73
7.28
7.28
7.28
7.27
7.26
7.24
7.03
7.03
7.02
7.02
7.01
6.80
5.49
5.49
5.48
3.76
2.98
2.97
2.97
2.96
2.95
2.94
2.93
2.41
2.17
2.16
2.16
1.85
1.84
1.83
1.67
Ts
NH
NHCO2Me
S6
0.84
1.92
2.06
1.12
1.98
0.94
1.00
0.94
2.93
2.11
0.99
2.95
1.99
0.99
1.06
1.20
1.08
f1 (ppm)

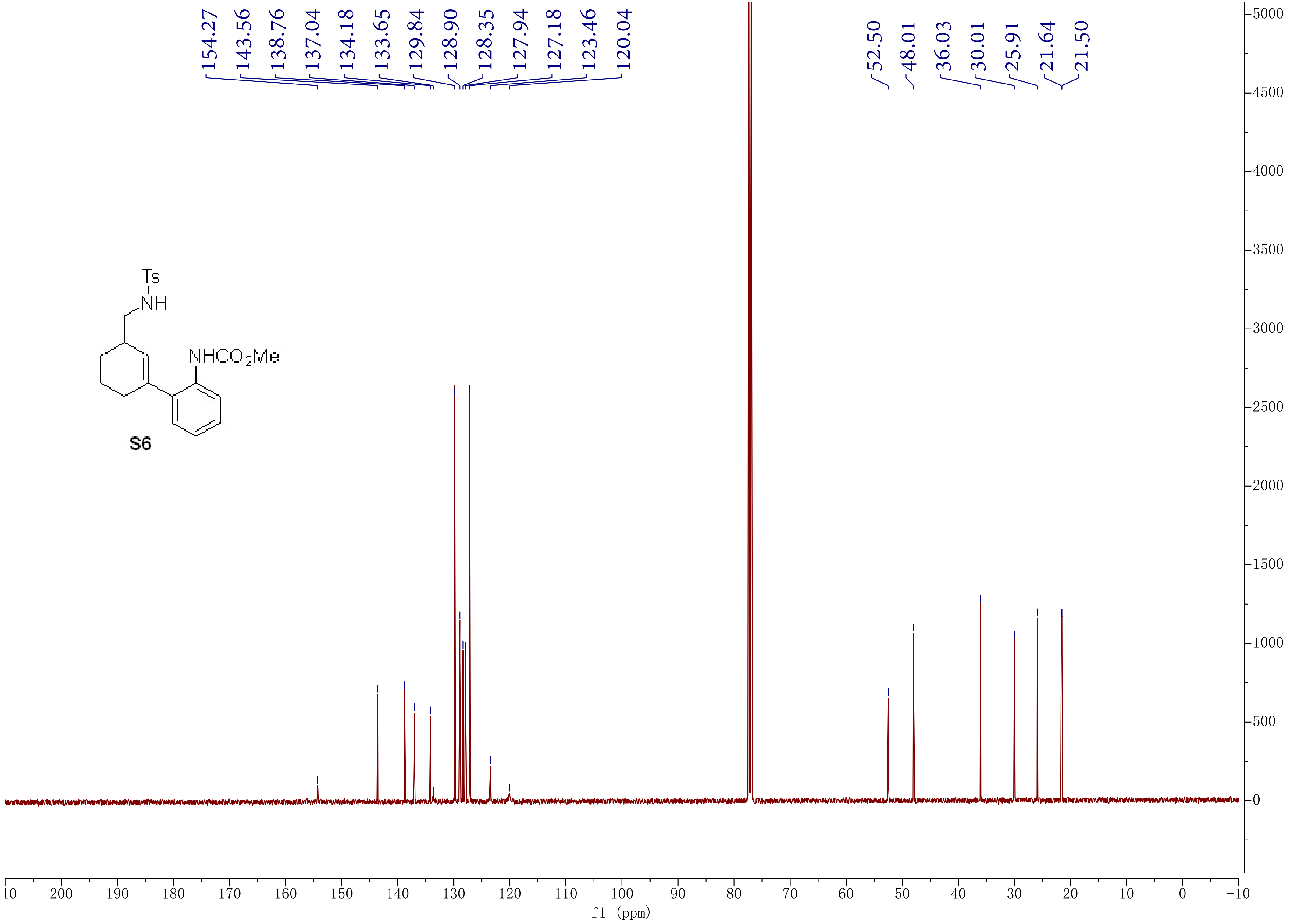
154.27
143.56
138.76
137.04
134.18
133.65
129.84
128.90
128.35
127.94
127.18
123.46
120.04
52.50
48.01
36.03
30.01
25.91
21.64
21.50
Ts
NH
NHCO2Me
S6
f1 (ppm)

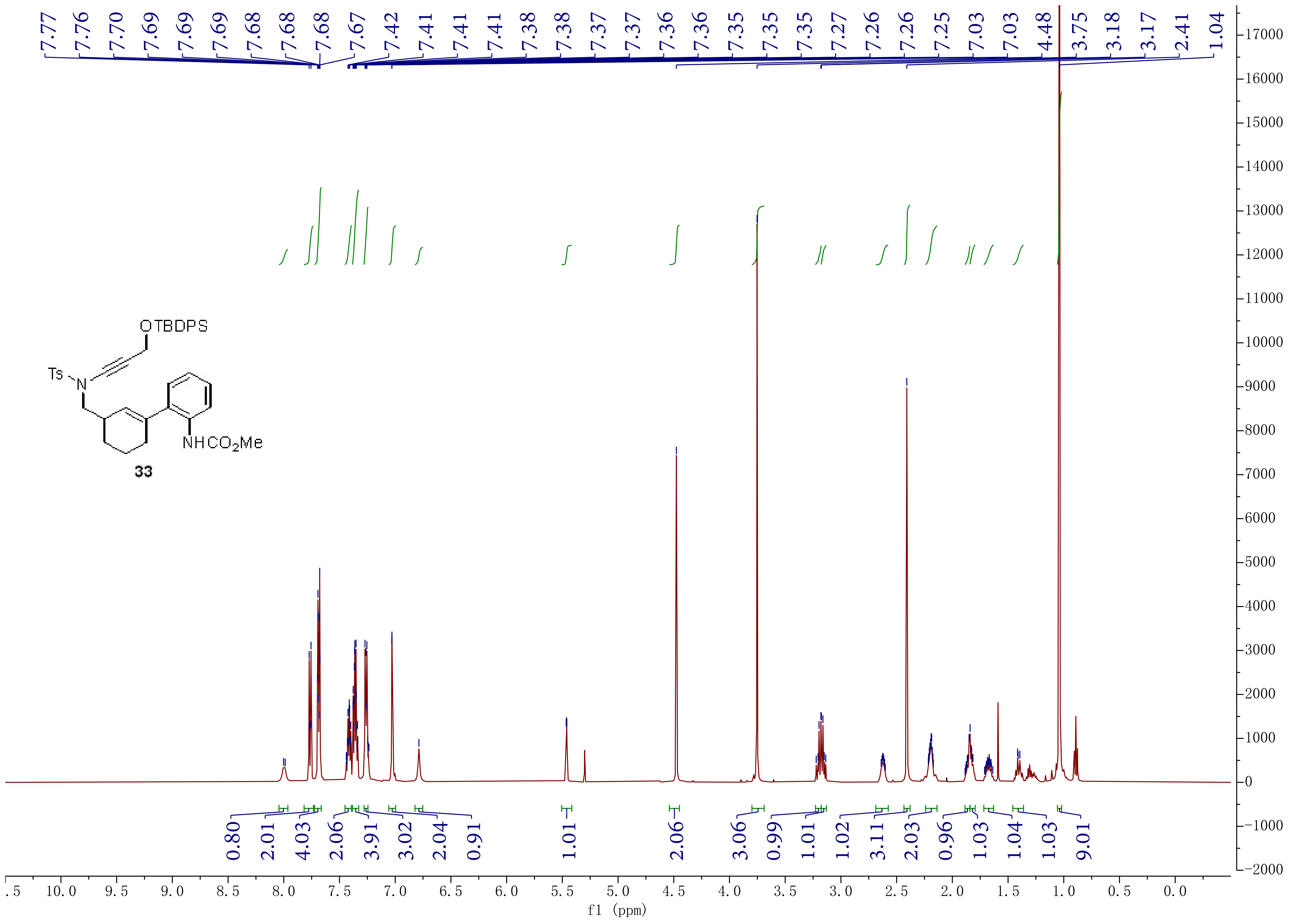
7.77 7.76 7.70 7.69 7.69 7.69 7.68 7.68 7.68 7.67 7.42 7.41 7.41 7.41 7.38 7.38 7.37 7.37 7.36 7.36 7.35 7.35 7.35 7.27 7.26 7.26 7.25 7.03 7.03 4.48 3.75 3.18 3.17 2.41 1.04
OTBDPS
Ts
N
NHCO2Me
33
0.80 2.01 4.03 2.06 3.91 3.02 2.04 0.91 1.01 2.06 3.06 0.99 1.01 1.02 3.11 2.03 0.96 1.03 1.04 1.03 9.01
f1 (ppm)

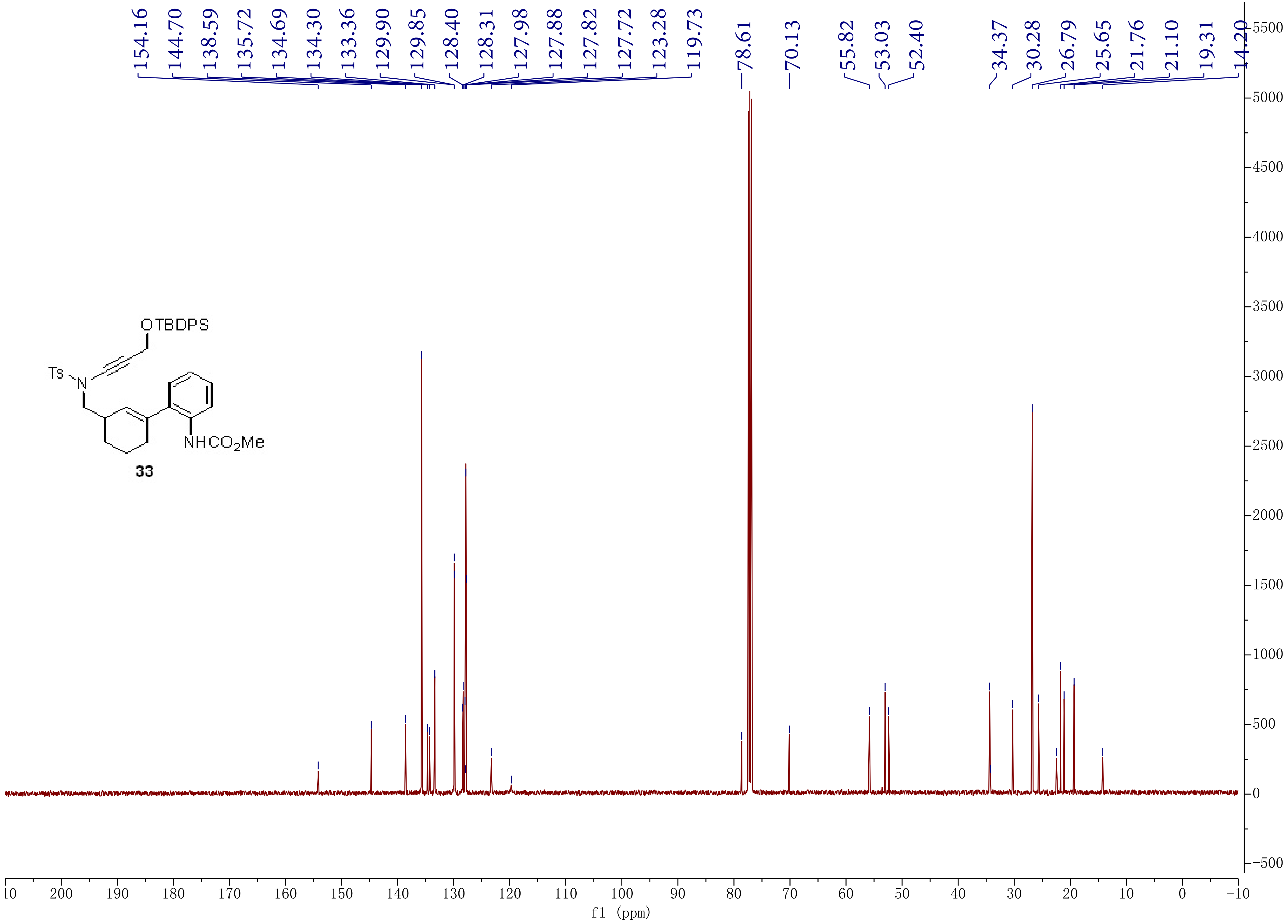
154.16
144.70
138.59
135.72
134.69
134.30
133.36
129.90
129.85
128.40
128.31
127.98
127.88
127.82
127.72
123.28
119.73
78.61
70.13
55.82
53.03
52.40
34.37
30.28
26.79
25.65
21.76
21.10
19.31
14.20
OTBDPS
Ts
N
NHCO2Me
33
f1 (ppm)

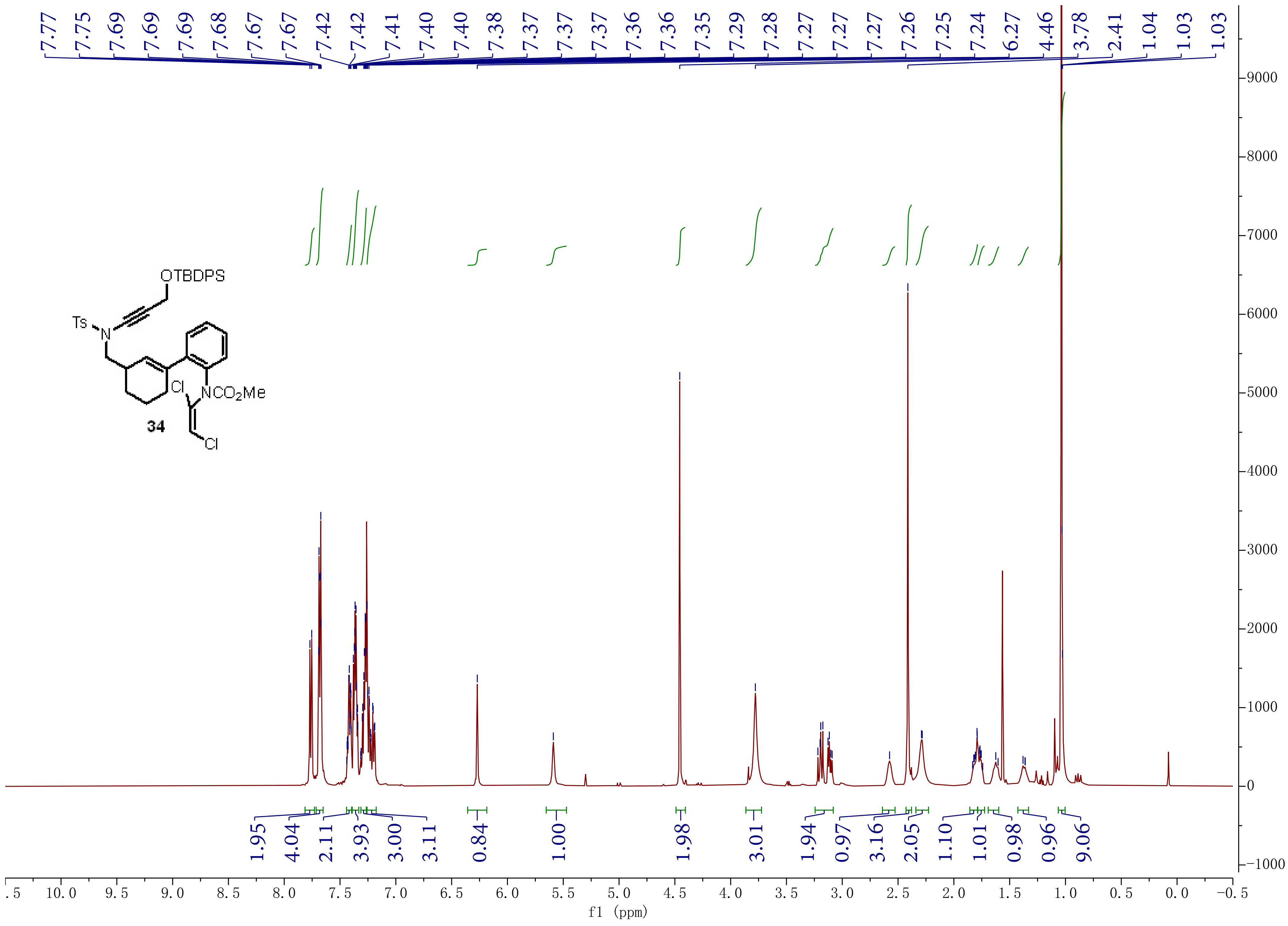

7.77
7.75
7.69
7.69
7.69
7.68
7.67
7.67
7.42
7.42
7.41
7.40
7.40
7.38
7.37
7.37
7.37
7.36
7.36
7.35
7.29
7.28
7.27
7.27
7.27
7.26
7.25
7.24
6.27
4.46
3.78
2.41
1.04
1.03
1.03
OTBDPS
Ts
N
Cl
NCO2Me
Cl
34
1.95
4.04
2.11
3.93
3.00
3.11
0.84
1.00
1.98
3.01
1.94
0.97
3.16
2.05
1.10
1.01
0.98
0.96
9.06
f1 (ppm)

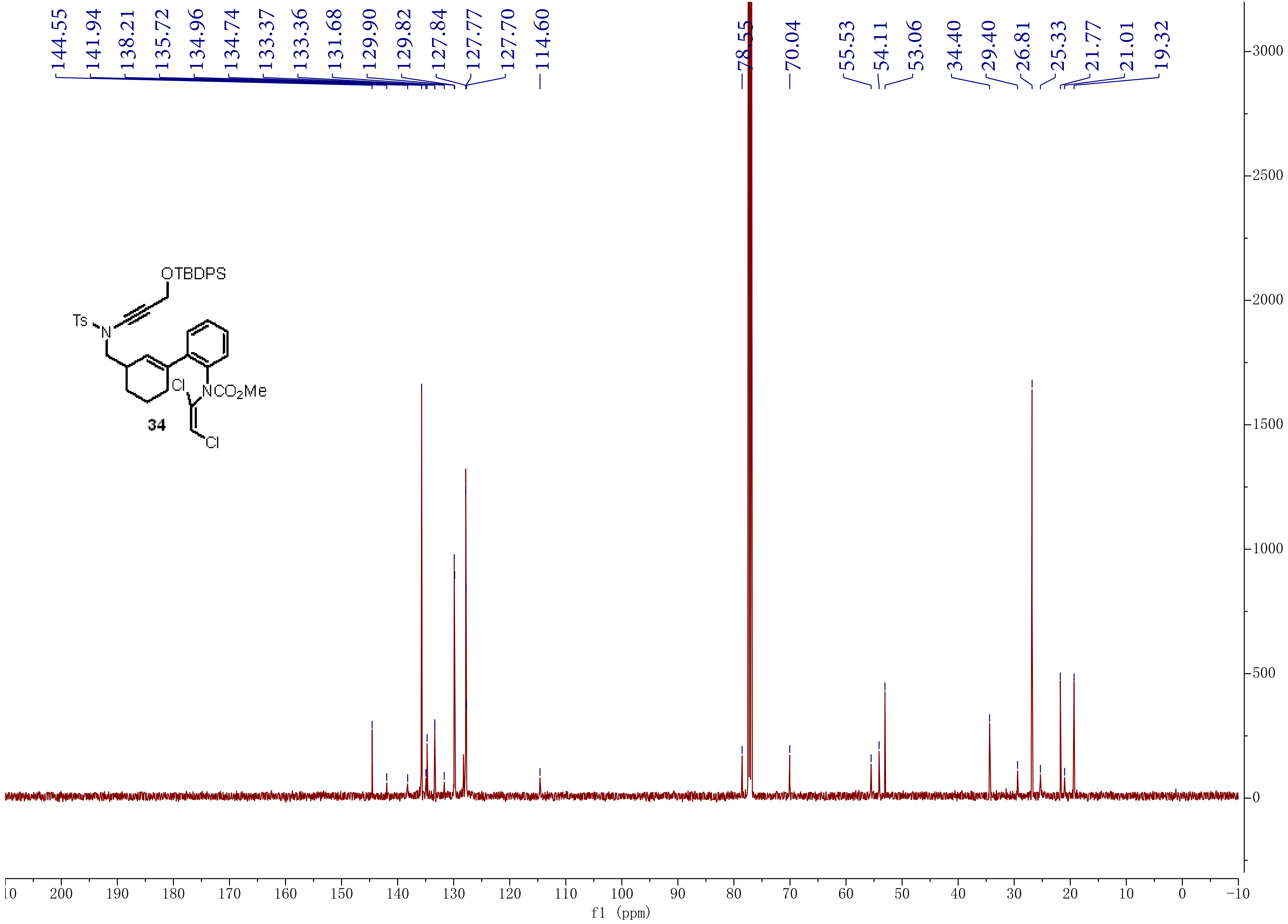
144.55
141.94
138.21
135.72
134.96
134.74
133.37
133.36
131.68
129.90
129.82
127.84
127.77
127.70
114.60
78.55
70.04
55.53
54.11
53.06
34.40
29.40
26.81
25.33
21.77
21.01
19.32
OTBDPS
Ts
N
Cl
NCO2Me
Cl
34
f1 (ppm)

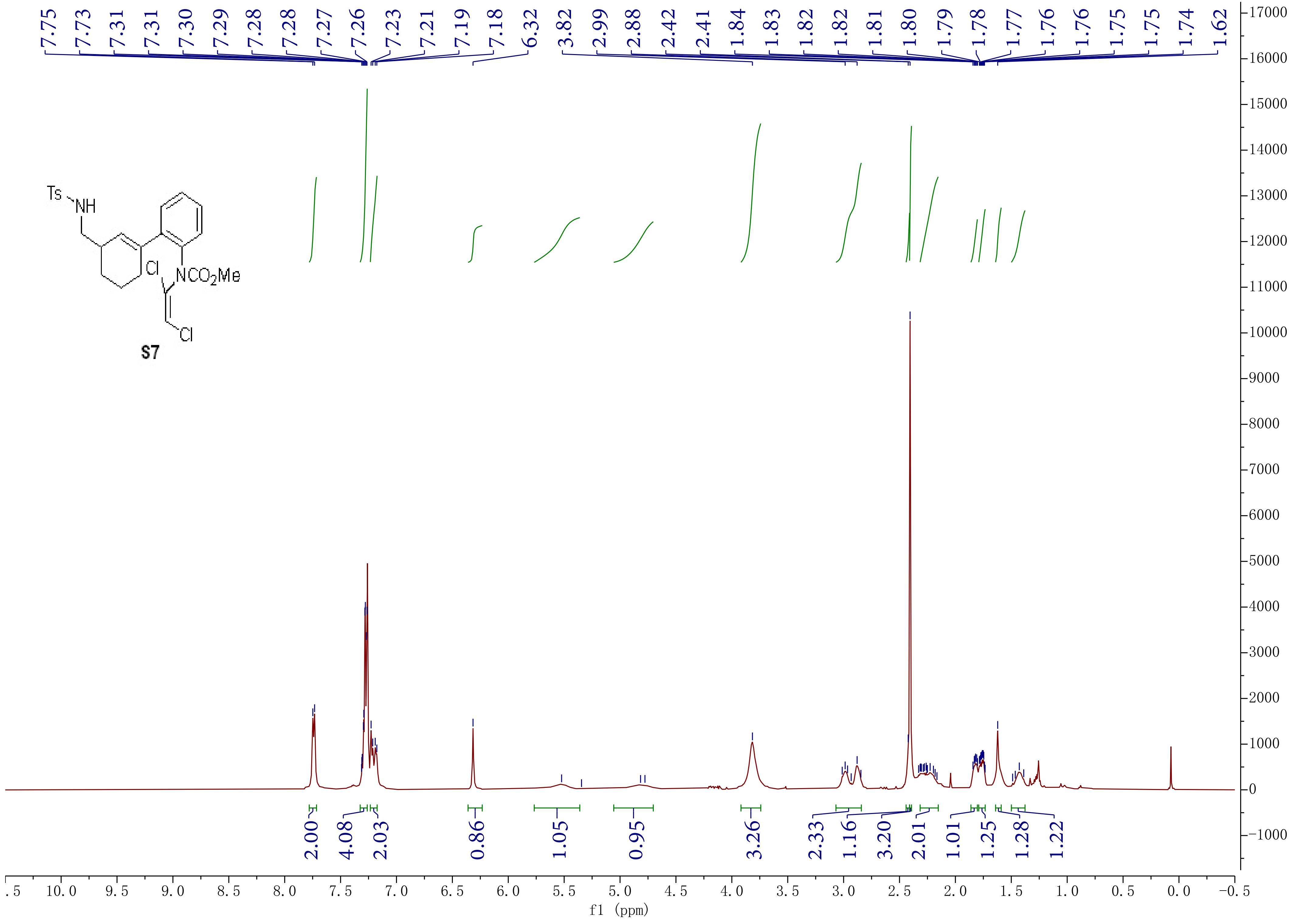
7.75
7.73
7.31
7.31
7.30
7.29
7.28
7.28
7.27
7.26
7.23
7.21
7.19
7.18
6.32
3.82
2.99
2.88
2.42
2.41
1.84
1.83
1.82
1.82
1.81
1.80
1.79
1.78
1.77
1.76
1.76
1.75
1.75
1.74
1.62
Ts
NH
Cl
NCO2Me
Cl
S7
2.00
4.08
2.03
0.86
1.05
0.95
3.26
2.33
1.16
3.20
2.01
1.01
1.25
1.28
1.22
f1 (ppm)

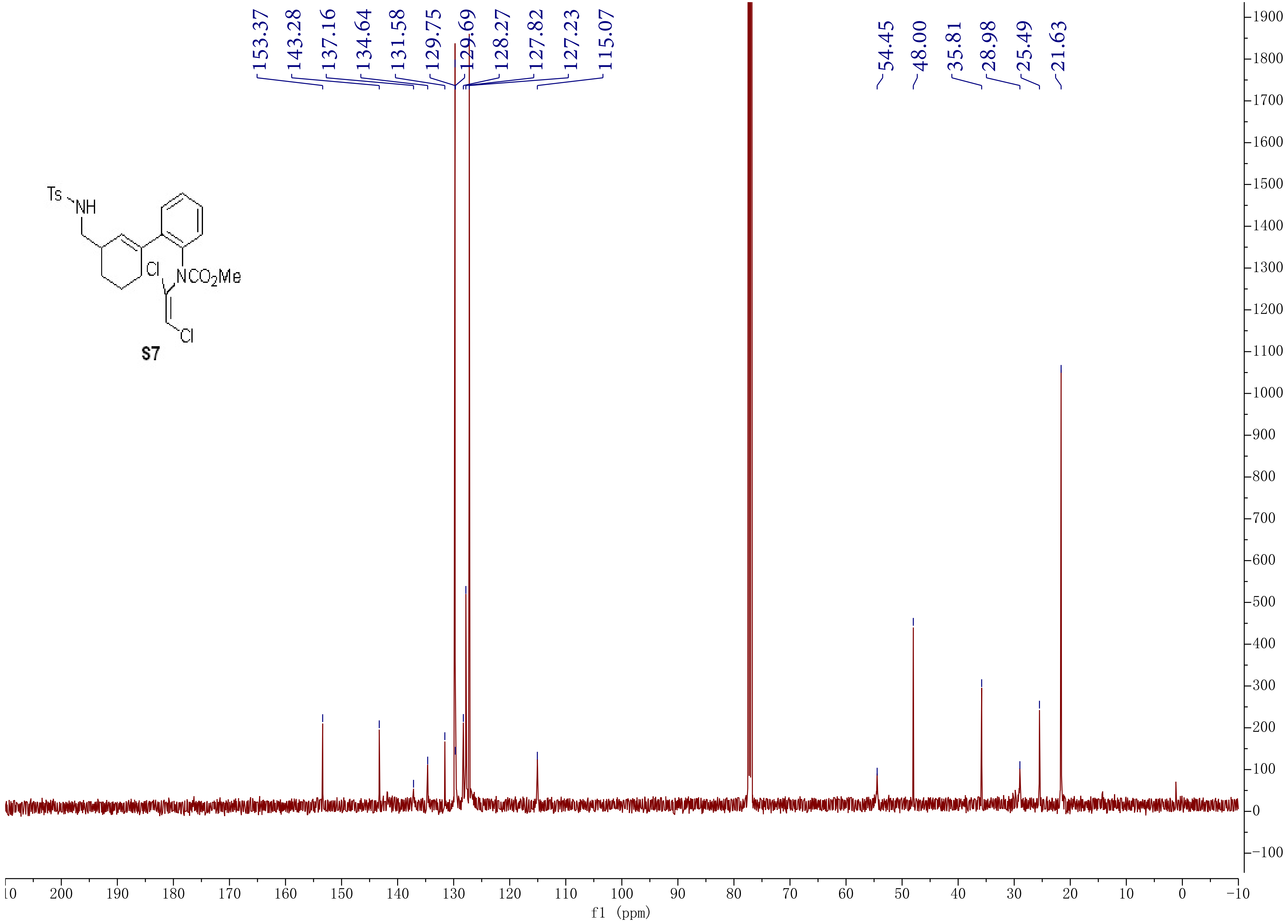
153.37
143.28
137.16
134.64
131.58
129.75
129.69
128.27
127.82
127.23
115.07
54.45
48.00
35.81
28.98
25.49
21.63
Ts
NH
Cl
NCO2Me
Cl
S7
f1 (ppm)

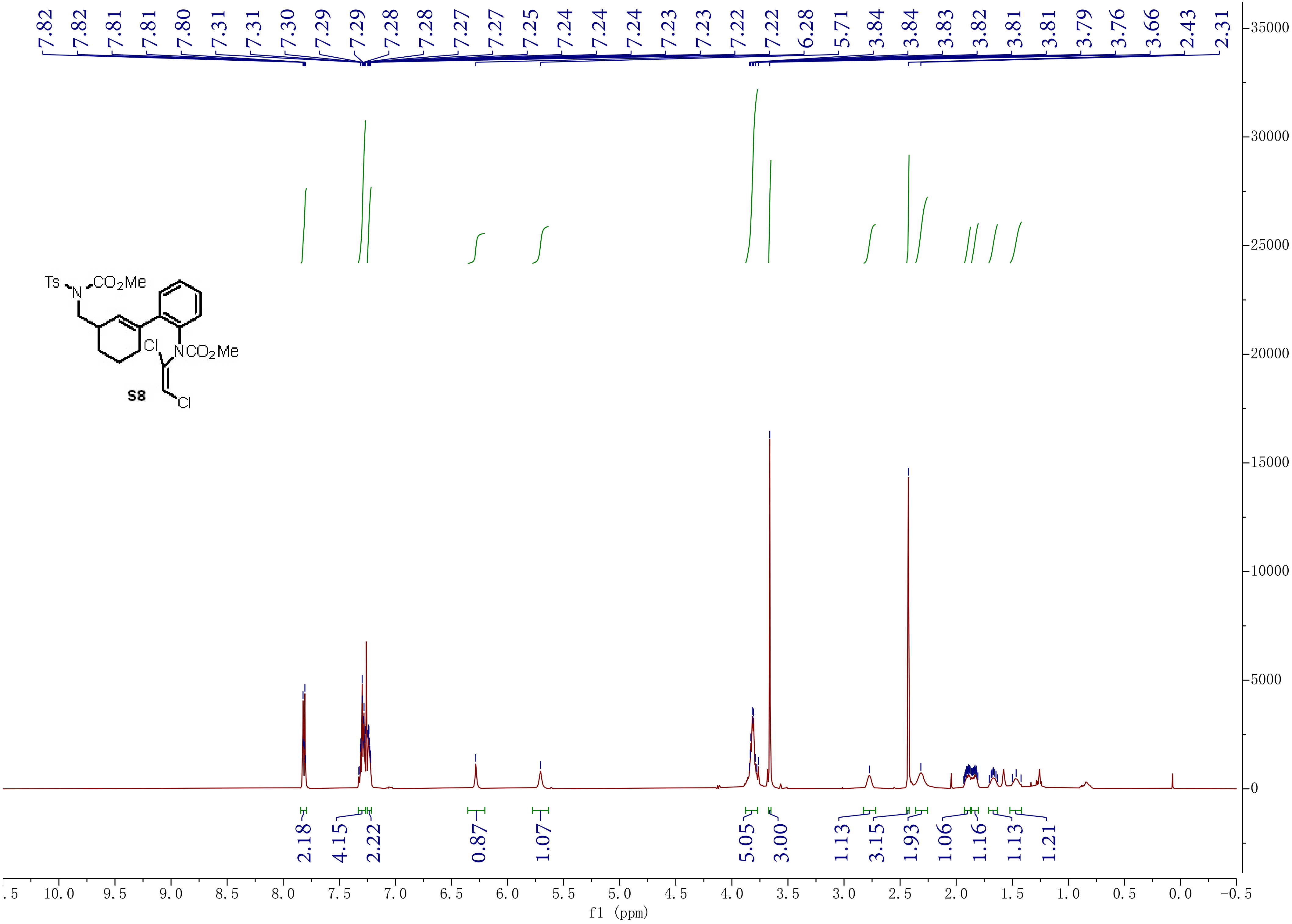
7.82 7.82 7.81 7.81 7.80 7.31 7.31 7.30 7.29 7.29 7.28 7.28 7.27 7.27 7.25 7.24 7.24 7.24 7.23 7.23 7.22 7.22 6.28 5.71 3.84 3.84 3.83 3.82 3.81 3.81 3.79 3.76 3.66 2.43 2.31
Ts
N
CO2Me
Cl
NCO2Me
Cl
S8
2.18 4.15 2.22 0.87 1.07 5.05 3.00 1.13 3.15 1.93 1.06 1.16 1.13 1.21
35000 30000 25000 20000 15000 10000 5000 0
.5 10.0 9.5 9.0 8.5 8.0 7.5 7.0 6.5 6.0 5.5 5.0 4.5 4.0 3.5 3.0 2.5 2.0 1.5 1.0 0.5 0.0 -0.5
f1 (ppm)

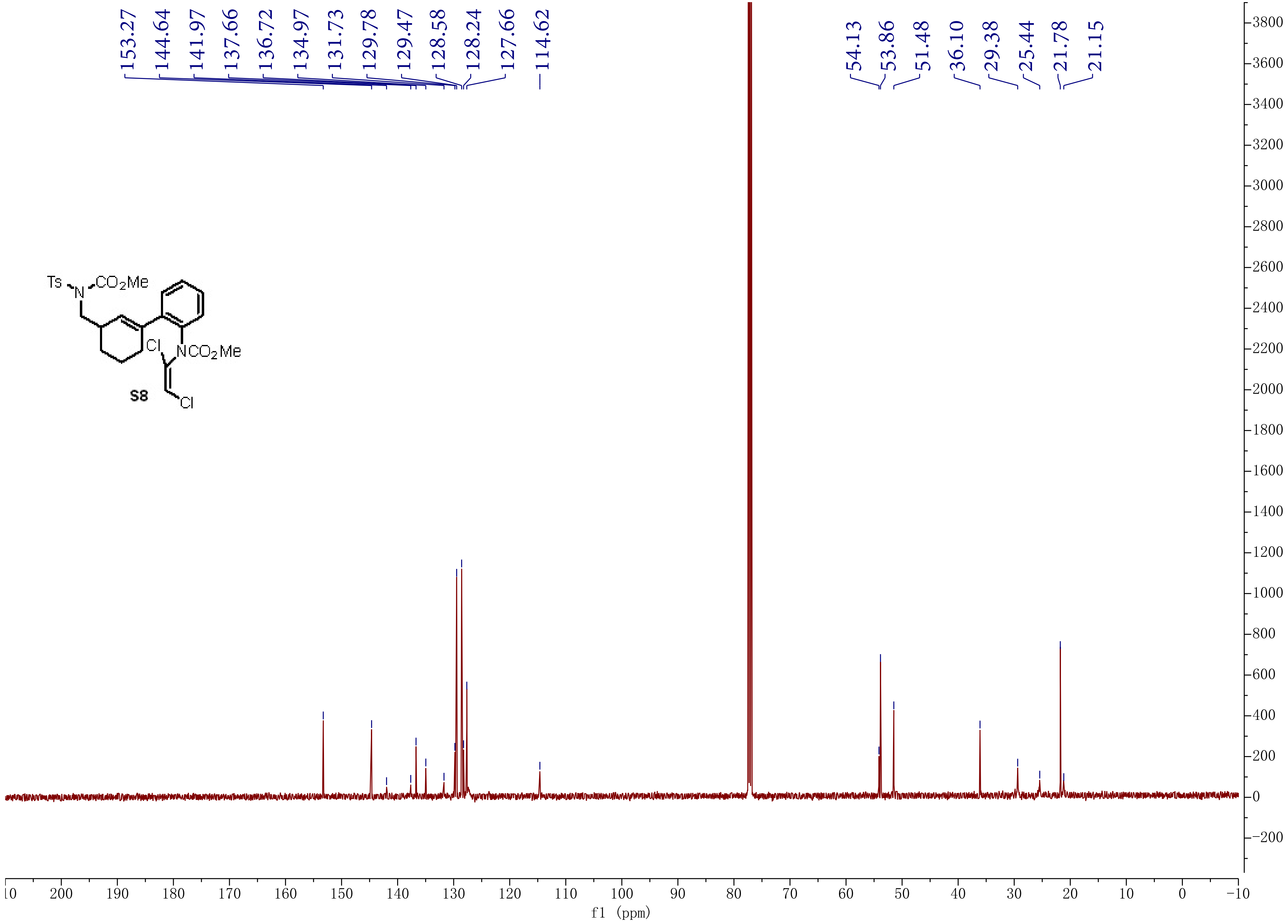
153.27
144.64
141.97
137.66
136.72
134.97
131.73
129.78
129.47
128.58
128.24
127.66
114.62
54.13
53.86
51.48
36.10
29.38
25.44
21.78
21.15
Ts
N
CO_2Me
Cl
NCO_2Me
Cl
S8
f1 (ppm)

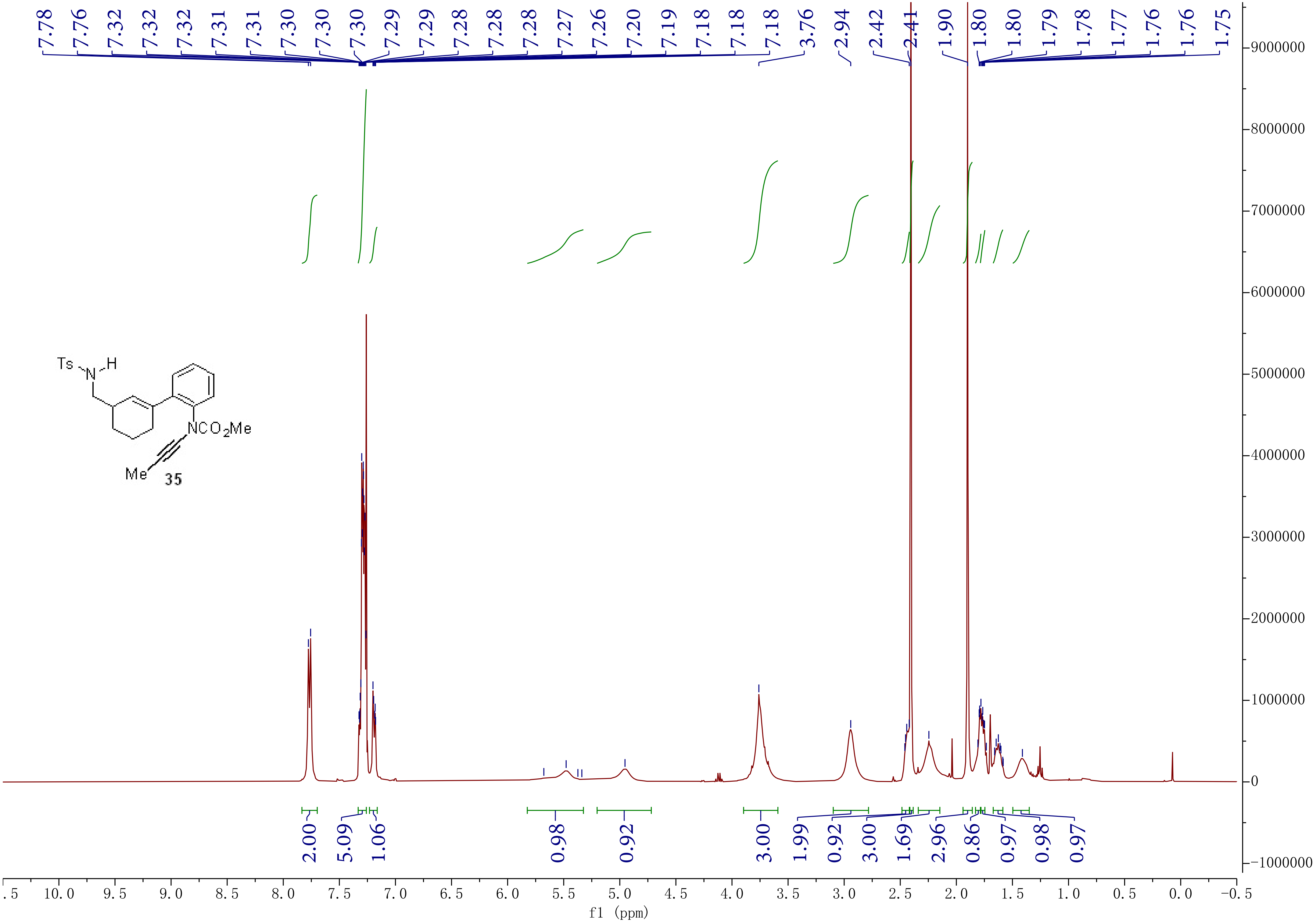

7.78
7.76
7.32
7.32
7.32
7.31
7.31
7.30
7.30
7.30
7.29
7.29
7.28
7.28
7.28
7.27
7.26
7.20
7.19
7.18
7.18
7.18
3.76
2.94
2.42
2.41
1.90
1.80
1.80
1.79
1.78
1.77
1.76
1.76
1.75
Ts
N
H
NCO2Me
Me
35
2.00
5.09
1.06
0.98
0.92
3.00
1.99
0.92
3.00
1.69
2.96
0.86
0.97
0.98
0.97
f1 (ppm)

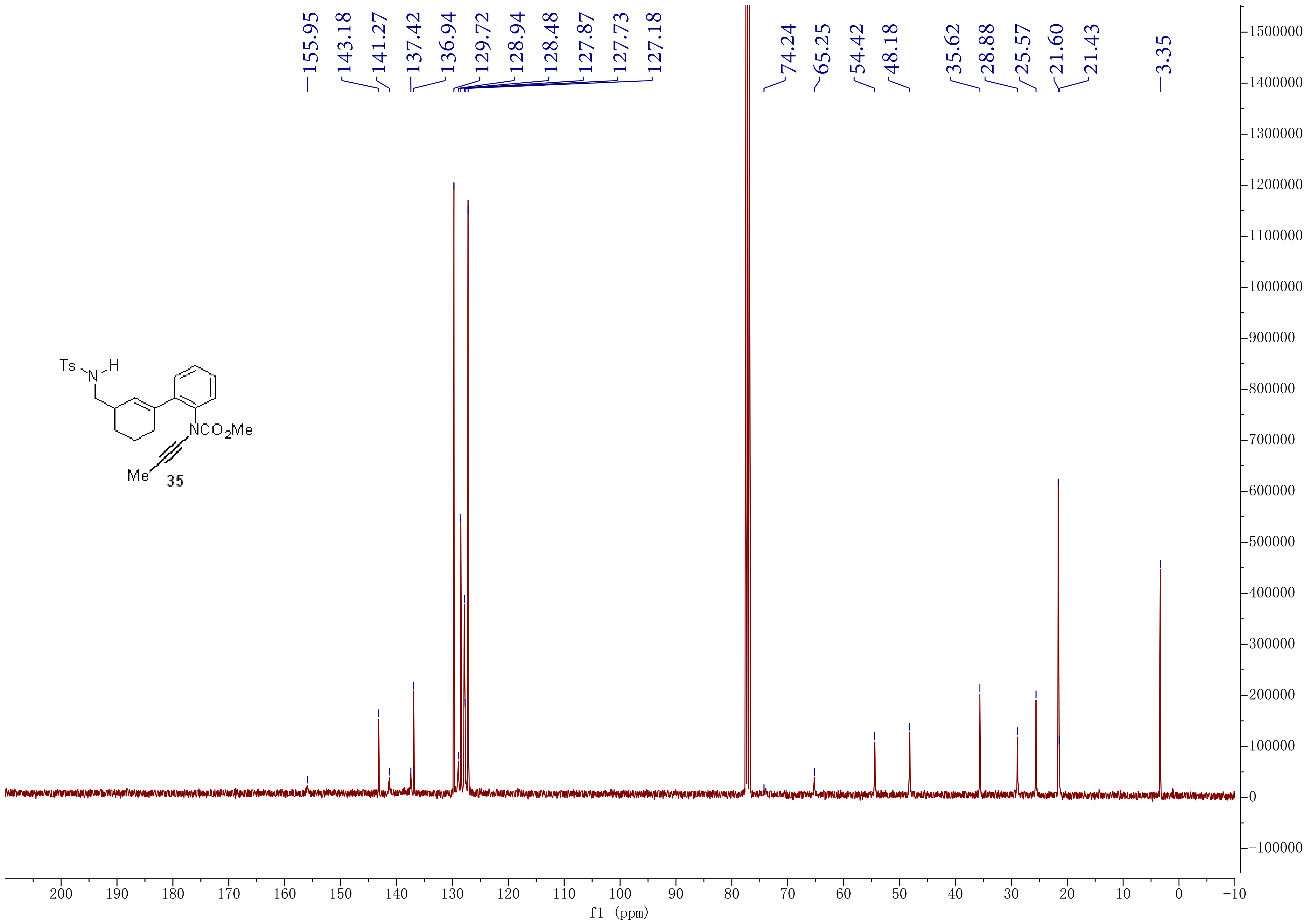
155.95
143.18
141.27
137.42
136.94
129.72
128.94
128.48
127.87
127.73
127.18
74.24
65.25
54.42
48.18
35.62
28.88
25.57
21.60
21.43
3.35
Ts
N
H
NCO$_2$Me
Me
35
f1 (ppm)

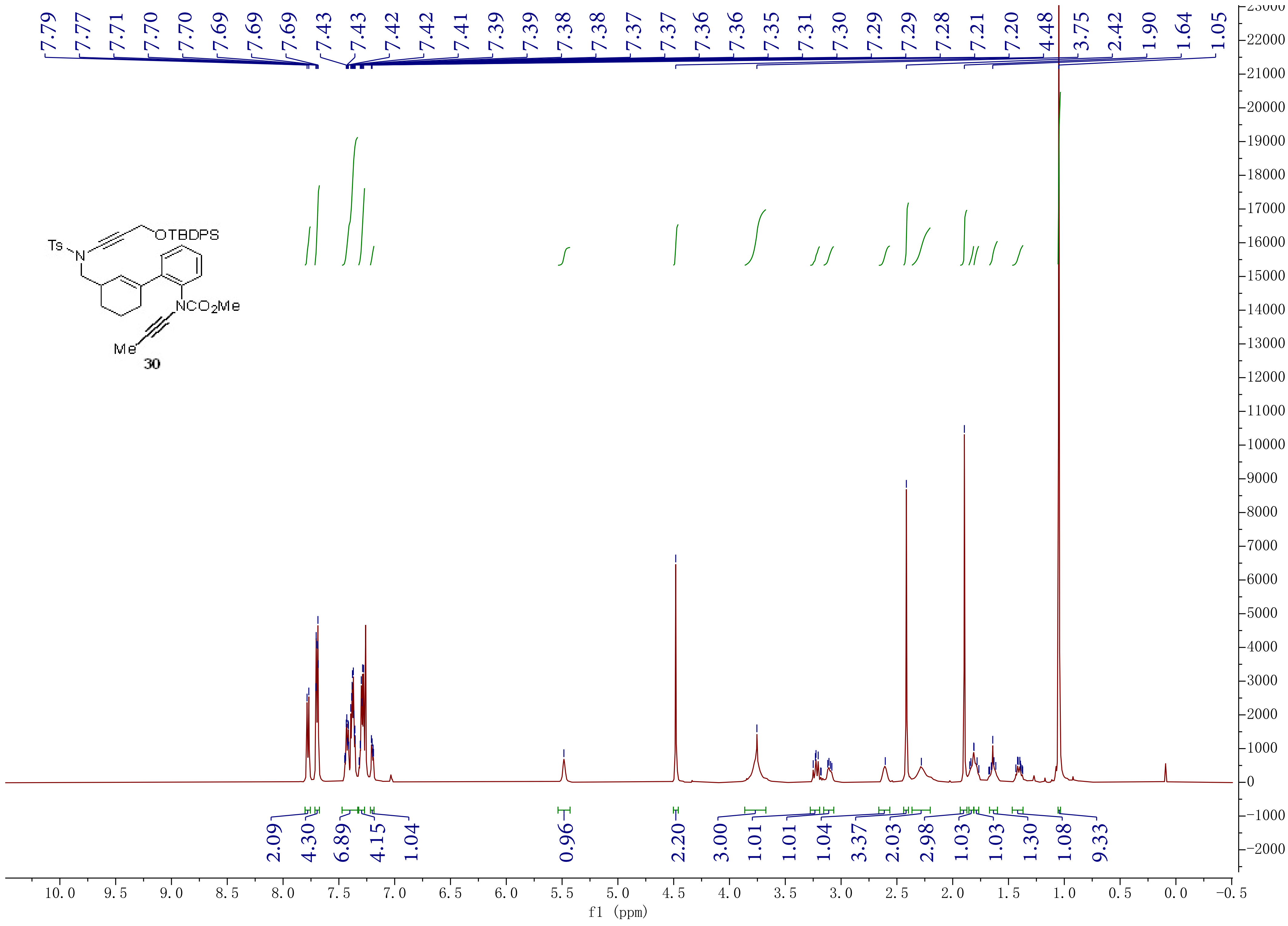

7.79 7.77 7.71 7.70 7.70 7.69 7.69 7.69 7.43 7.43 7.42 7.42 7.41 7.39 7.39 7.38 7.38 7.37 7.37 7.36 7.36 7.35 7.31 7.30 7.29 7.29 7.28 7.21 7.20 4.48 3.75 2.42 1.90 1.64 1.05
Ts
N
OTBDPS
NCO2Me
Me
30
2.09 4.30 6.89 4.15 1.04 0.96 2.20 3.00 1.01 1.01 1.04 3.37 2.03 2.98 1.03 1.03 1.30 1.08 9.33
10.0 9.5 9.0 8.5 8.0 7.5 7.0 6.5 6.0 5.5 5.0 4.5 4.0 3.5 3.0 2.5 2.0 1.5 1.0 0.5 0.0 -0.5
f1 (ppm)

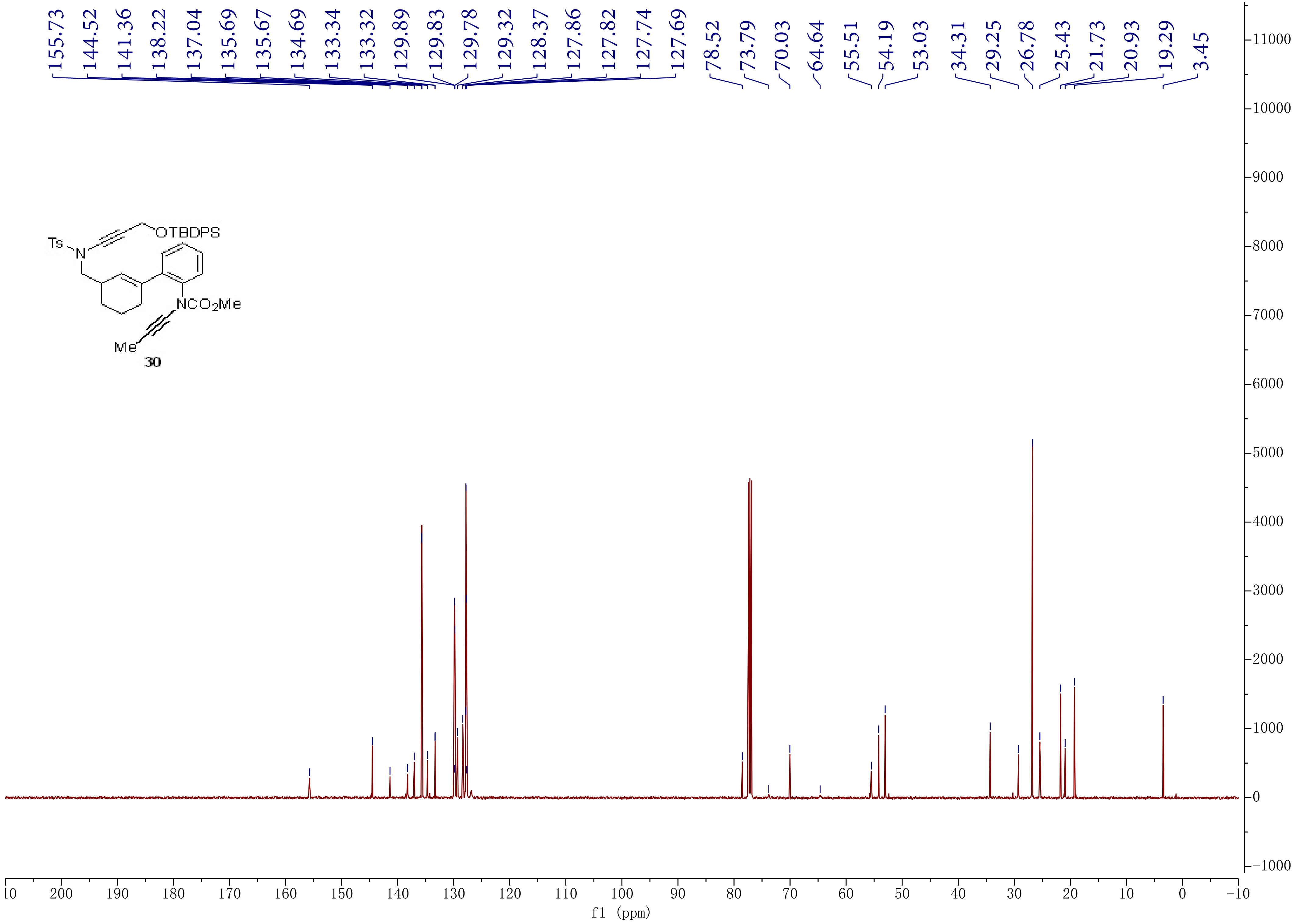

155.73
144.52
141.36
138.22
137.04
135.69
135.67
134.69
133.34
133.32
129.89
129.83
129.78
129.32
128.37
127.86
127.82
127.74
127.69
78.52
73.79
70.03
64.64
55.51
54.19
53.03
34.31
29.25
26.78
25.43
21.73
20.93
19.29
3.45
Ts
N
OTBDPS
NCO2Me
Me
30
11000
10000
9000
8000
7000
6000
5000
4000
3000
2000
1000
0
−1000
10
200
190
180
170
160
150
140
130
120
110
100
90
80
70
60
50
40
30
20
10
0
−10
f1 (ppm)

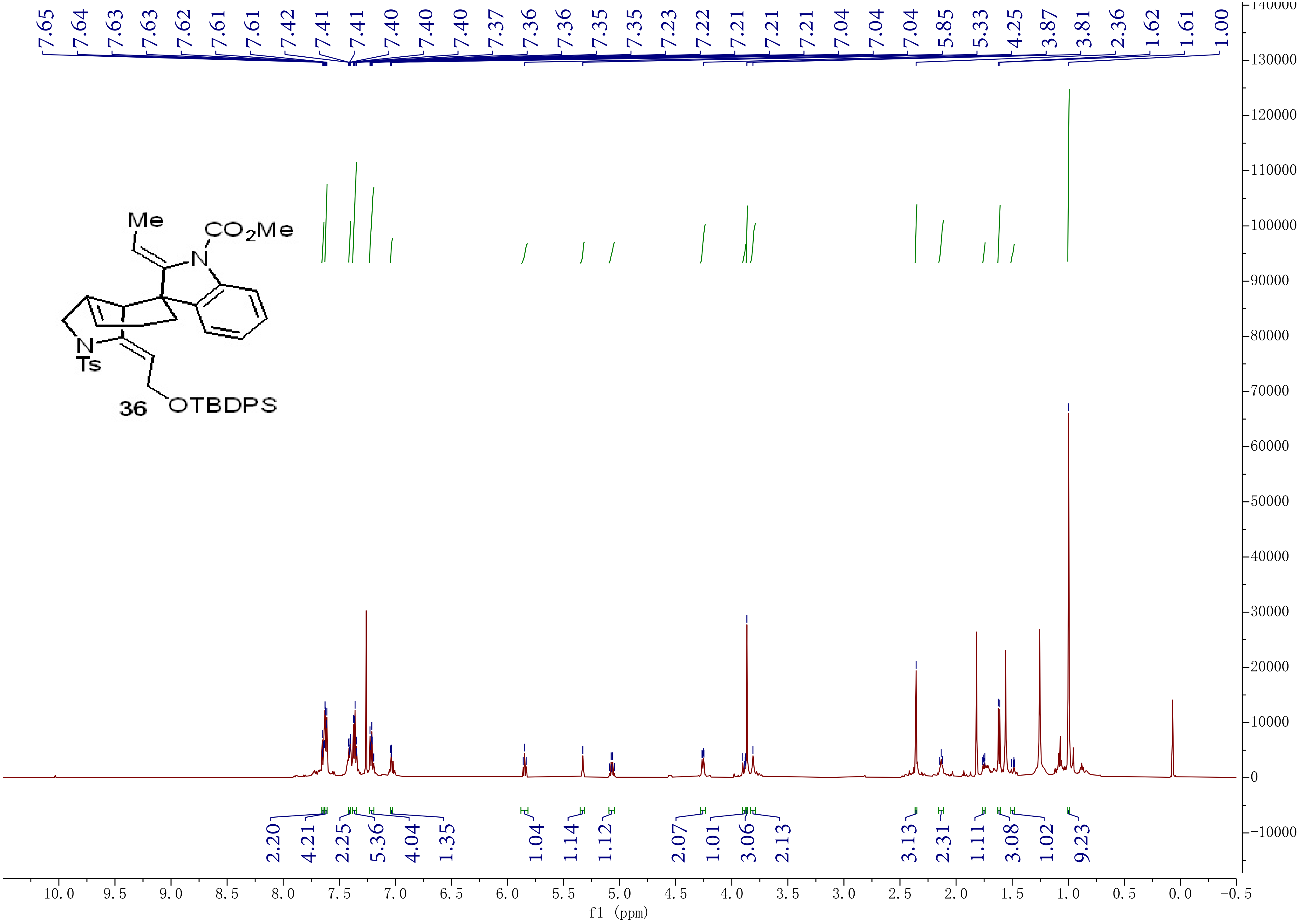
7.65 7.64 7.63 7.63 7.62 7.61 7.61 7.42 7.41 7.41 7.40 7.40 7.40 7.37 7.36 7.36 7.35 7.35 7.23 7.22 7.21 7.21 7.21 7.04 7.04 7.04 5.85 5.33 4.25 3.87 3.81 2.36 1.62 1.61 1.00
Me
CO_2Me
N
N
Ts
OTBDPS
36
2.20 4.21 2.25 5.36 4.04 1.35 1.04 1.14 1.12 2.07 1.01 3.06 2.13 3.13 2.31 1.11 3.08 1.02 9.23
10.0 9.5 9.0 8.5 8.0 7.5 7.0 6.5 6.0 5.5 5.0 4.5 4.0 3.5 3.0 2.5 2.0 1.5 1.0 0.5 0.0 -0.5
f1 (ppm)
140000 130000 120000 110000 100000 90000 80000 70000 60000 50000 40000 30000 20000 10000 0 -10000

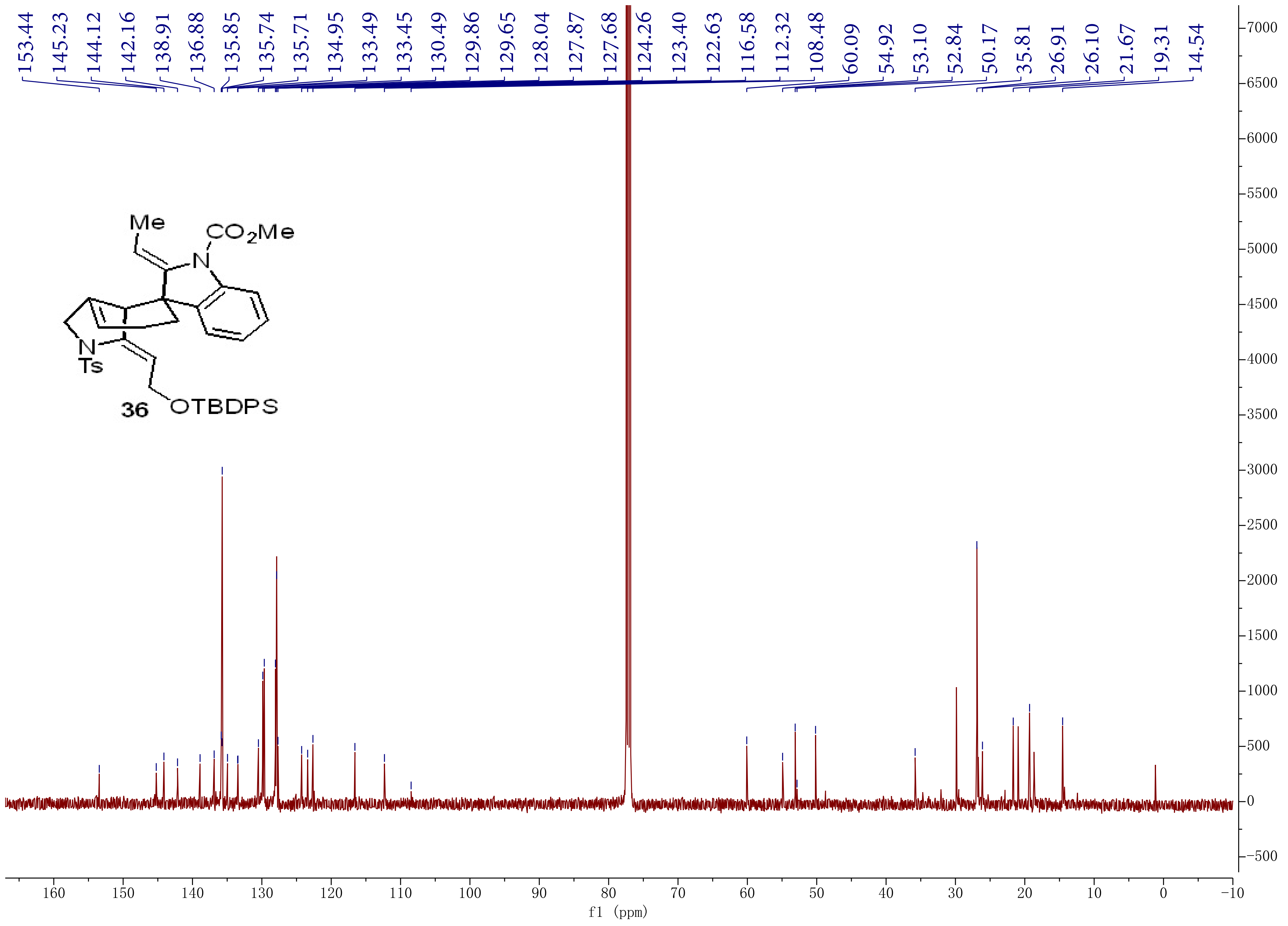
153.44
145.23
144.12
142.16
138.91
136.88
135.85
135.74
135.71
134.95
133.49
133.45
130.49
129.86
129.65
128.04
127.87
127.68
124.26
123.40
122.63
116.58
112.32
108.48
60.09
54.92
53.10
52.84
50.17
35.81
26.91
26.10
21.67
19.31
14.54
Me
CO_2Me
N
N
Ts
36
OTBDPS
f1 (ppm)

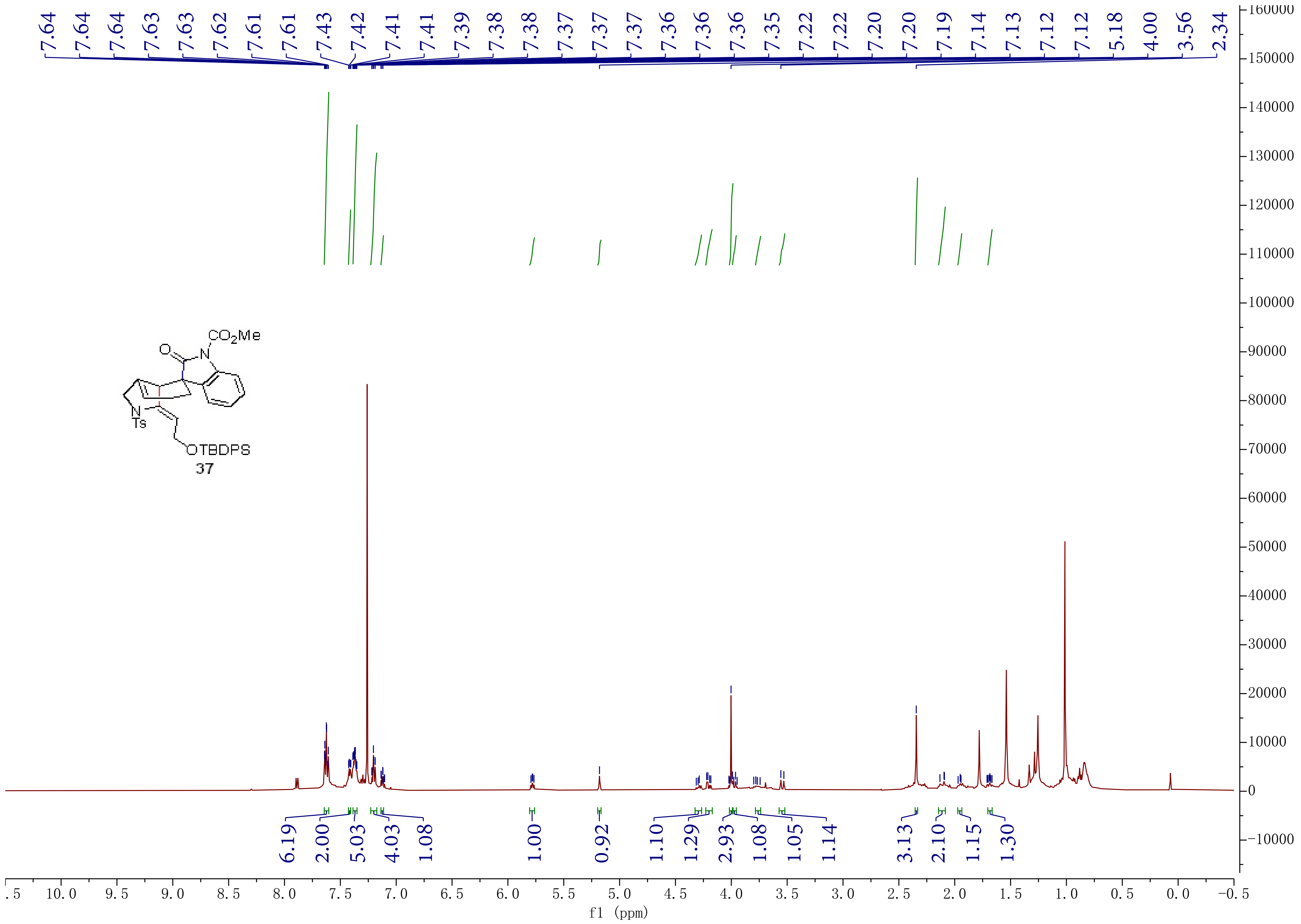
7.64 7.64 7.64 7.63 7.63 7.62 7.61 7.61 7.43 7.42 7.41 7.41 7.39 7.38 7.38 7.37 7.37 7.37 7.36 7.36 7.36 7.35 7.22 7.22 7.20 7.20 7.19 7.14 7.13 7.12 7.12 5.18 4.00 3.56 2.34
CO2Me
O
N
N
Ts
OTBDPS
37
6.19 2.00 5.03 4.03 1.08 1.00 0.92 1.10 1.29 2.93 1.08 1.05 1.14 3.13 2.10 1.15 1.30
f1 (ppm)

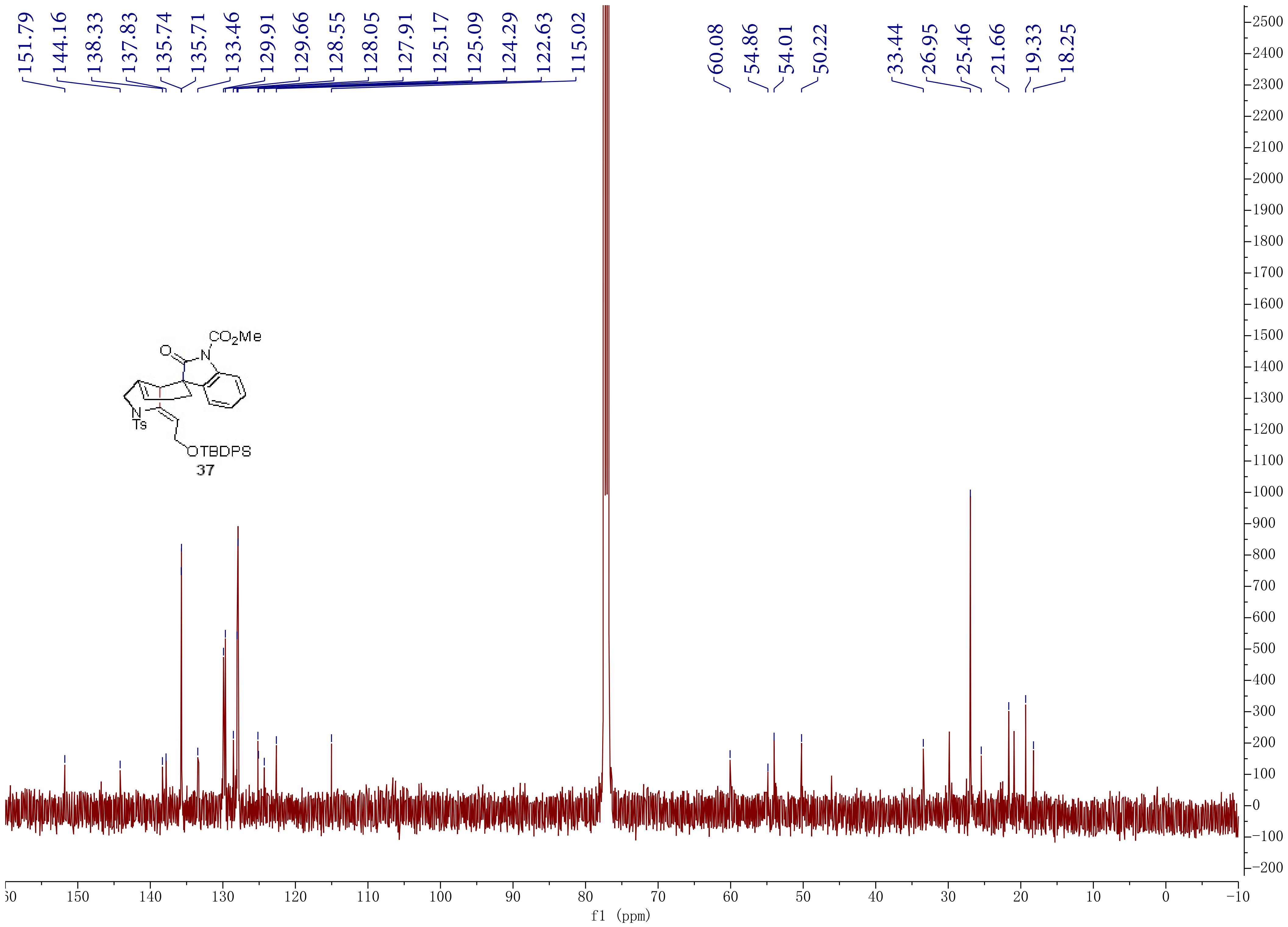
151.79
144.16
138.33
137.83
135.74
135.71
133.46
129.91
129.66
128.55
128.05
127.91
125.17
125.09
124.29
122.63
115.02
60.08
54.86
54.01
50.22
33.44
26.95
25.46
21.66
19.33
18.25
CO2Me
O
N
N
Ts
OTBDPS
37
f1 (ppm)

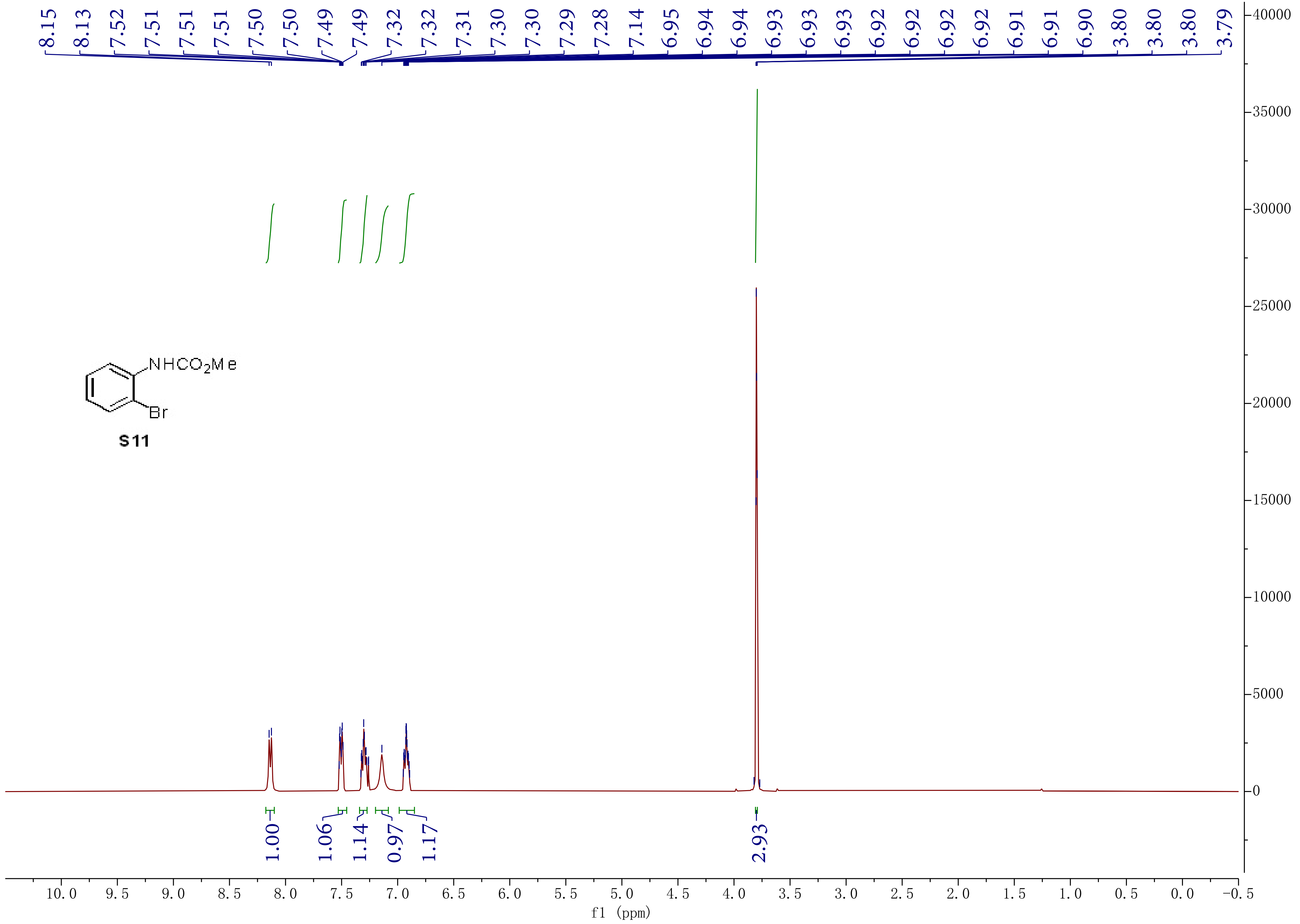
8.15
8.13
7.52
7.51
7.51
7.51
7.50
7.50
7.49
7.49
7.32
7.32
7.31
7.30
7.30
7.29
7.28
7.14
6.95
6.94
6.94
6.93
6.93
6.93
6.92
6.92
6.92
6.92
6.91
6.91
6.90
3.80
3.80
3.80
3.79
NHCO2Me
Br
S11
1.00
1.06
1.14
0.97
1.17
2.93
f1 (ppm)

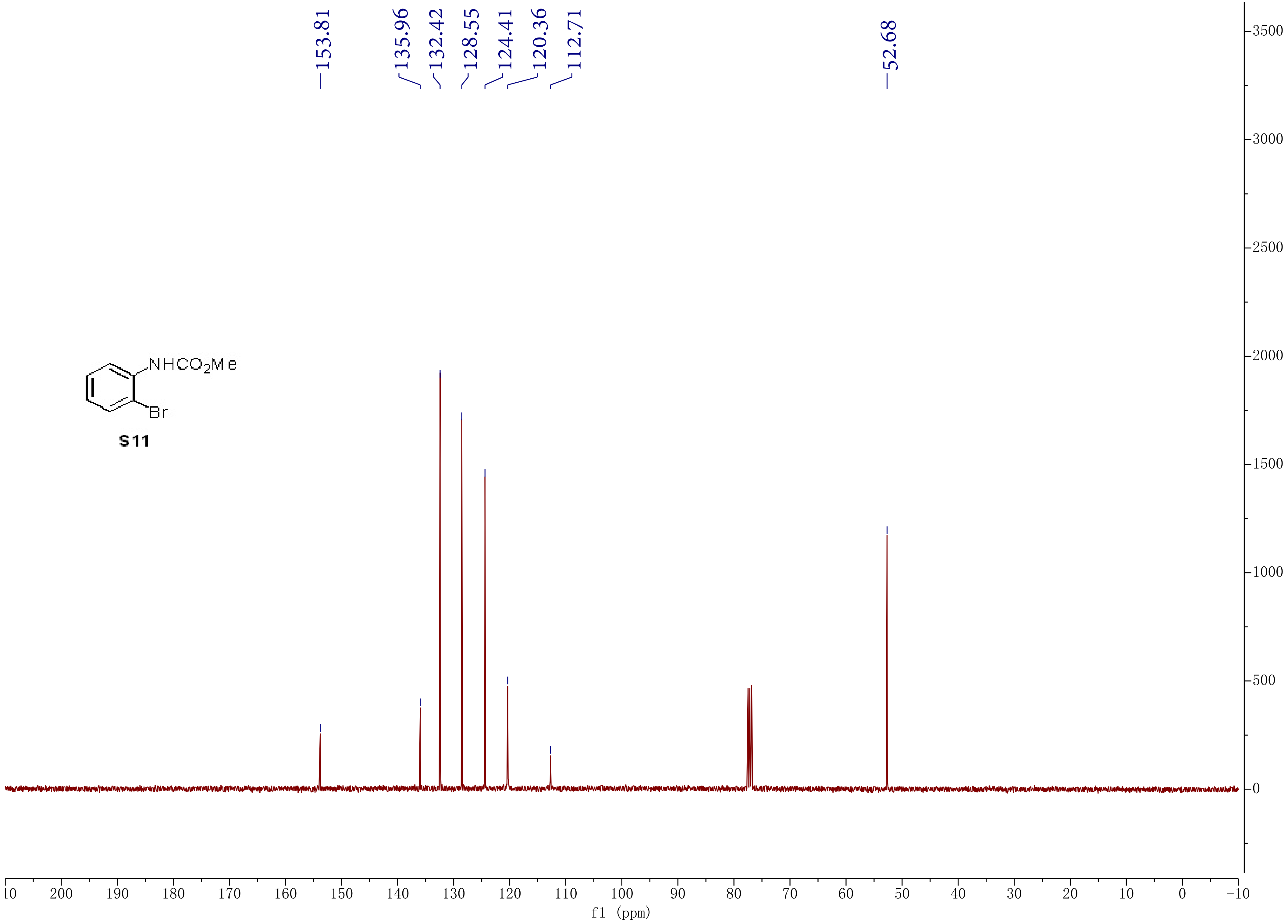
153.81
135.96
132.42
128.55
124.41
120.36
112.71
52.68
NHCO2Me
Br
S11
f1 (ppm)

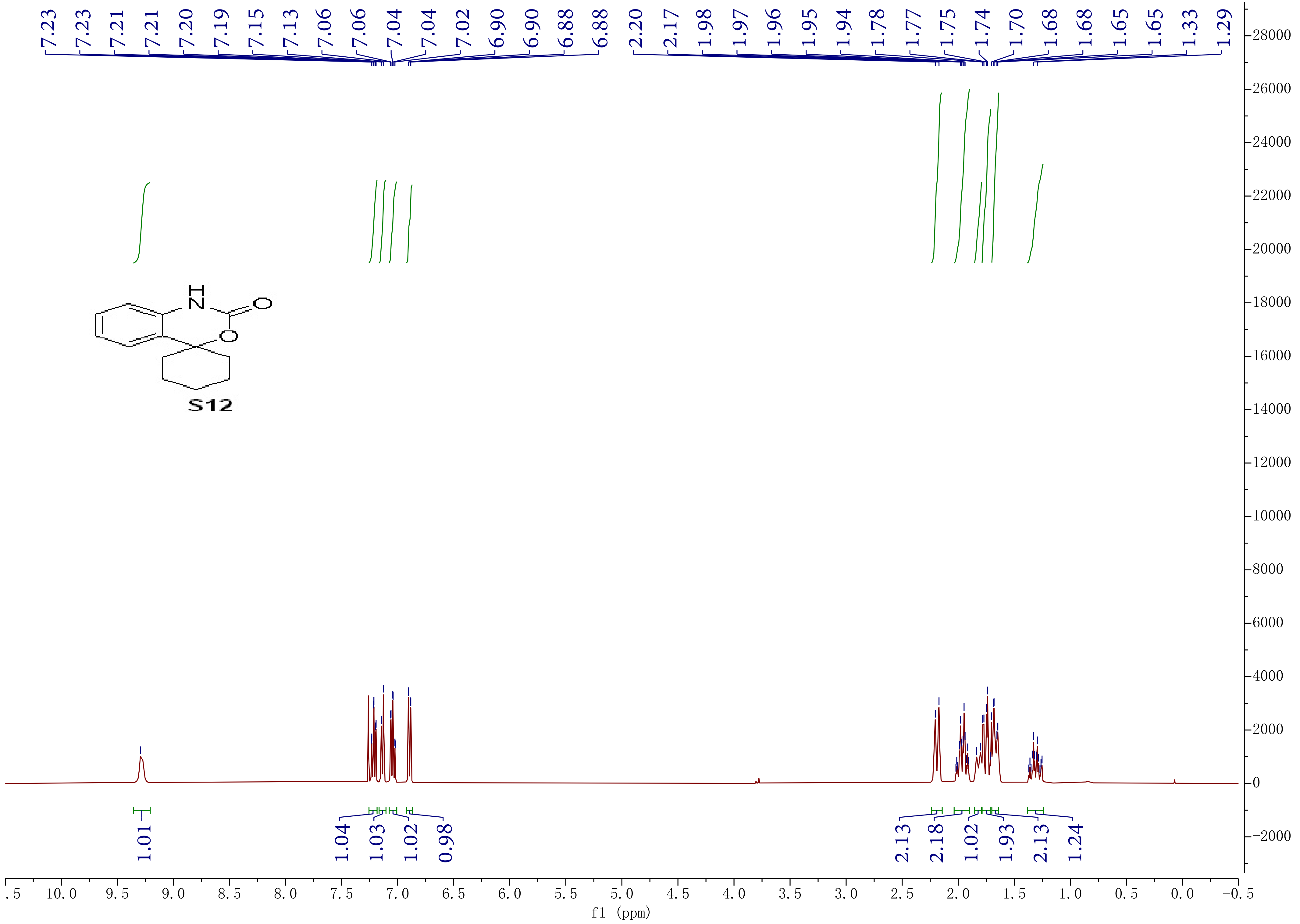

7.23 7.23 7.21 7.21 7.20 7.19 7.15 7.13 7.06 7.06 7.04 7.04 7.02 6.90 6.90 6.88 6.88
2.20 2.17 1.98 1.97 1.96 1.95 1.94 1.78 1.77 1.75 1.74 1.70 1.68 1.68 1.65 1.65 1.33 1.29
S12
1.01
1.04 1.03 1.02 0.98
2.13 2.18 1.02 1.93 2.13 1.24
f1 (ppm)

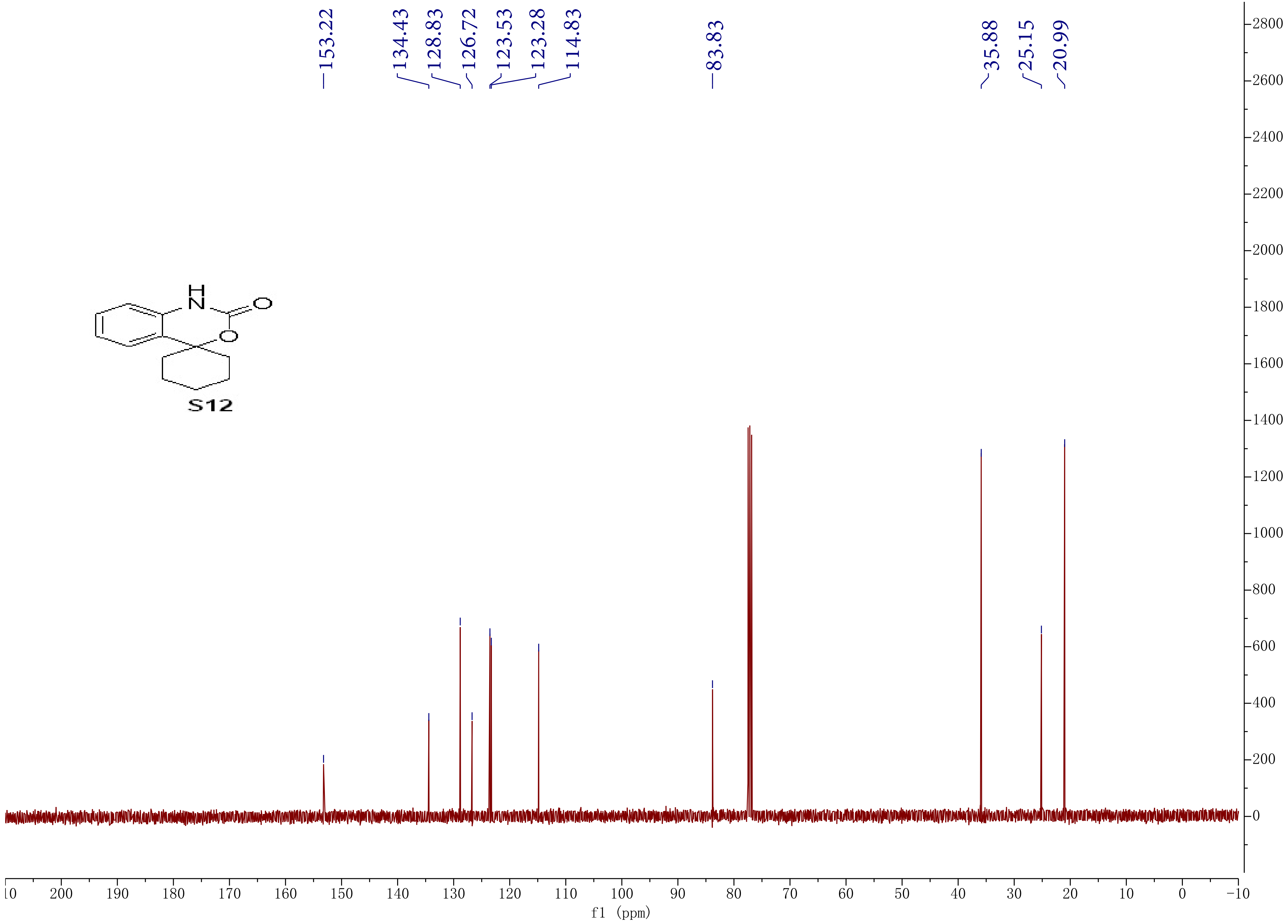
153.22
134.43
128.83
126.72
123.53
123.28
114.83
83.83
35.88
25.15
20.99
S12
f1 (ppm)
